# Aldehyde–Olefin Couplings Via Sulfoxylate-Mediated Oxidative Generation of Ketyl Radical Anions

**DOI:** 10.1021/jacs.4c10093

**Published:** 2024-09-20

**Authors:** Zhihang Li, Joseph A. Tate, Adam Noble

**Affiliations:** † School of Chemistry, University of Bristol, Bristol BS8 1TS, U.K.; ‡ Syngenta, Jealott’s Hill International Research Centre, Bracknell RG42 6EY, U.K.

## Abstract

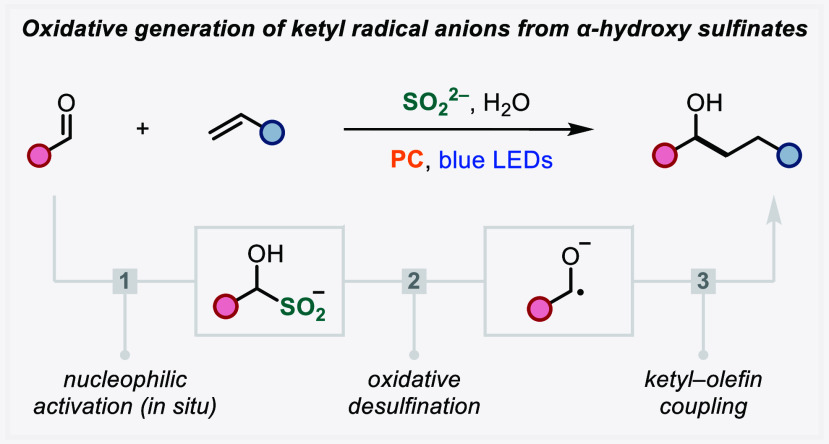

Ketyl radicals are valuable reactive intermediates because they allow carbonyl chemistry to be extended beyond traditional electrophilic reactivity through simple single-electron reduction to a nucleophilic radical. However, this pathway is challenging due to the large negative reduction potentials of carbonyls, thus requiring highly reducing conditions. Herein, we describe the development of an alternative strategy to access ketyl radicals from aldehydes, which avoids the reduction pathway by instead proceeding via single-electron oxidation and desulfination of α-hydroxy sulfinates. These redox-active aldehyde adducts are generated *in situ* through the addition of sulfoxylate (SO_2_^2–^) to aldehydes and possess low oxidation potentials, thereby facilitating ketyl radical formation and circumventing the need for strongly reducing conditions. We demonstrate the application of this sulfoxylate-mediated ketyl radical formation in ketyl–olefin coupling reactions.

The transformation of carbonyls into ketyl radicals is a classic approach to achieving umpolung reactivity by converting an electrophilic C=O bond into a nucleophilic radical.^1^ By reversing the polarity of the carbonyl and switching from a two-electron to a single-electron pathway, the range of alcohol products that can be accessed is dramatically expanded, hence ketyl radical-mediated reactions have found widespread application in organic synthesis.^1,2^ Mechanistically, the most direct route to ketyl radicals from carbonyls is by single-electron reduction (Scheme 1A). However, this pathway is susceptible to practical challenges due to the large negative reduction potentials of aldehydes and ketones,^3^ which often necessitate the use of superstoichiometric quantities of strong metal reductants,^4^ most commonly SmI_2_,^2^ or other forcing conditions, including UV irradiation or electrochemistry at deeply reducing potentials.^5,6^

**Scheme 1:**
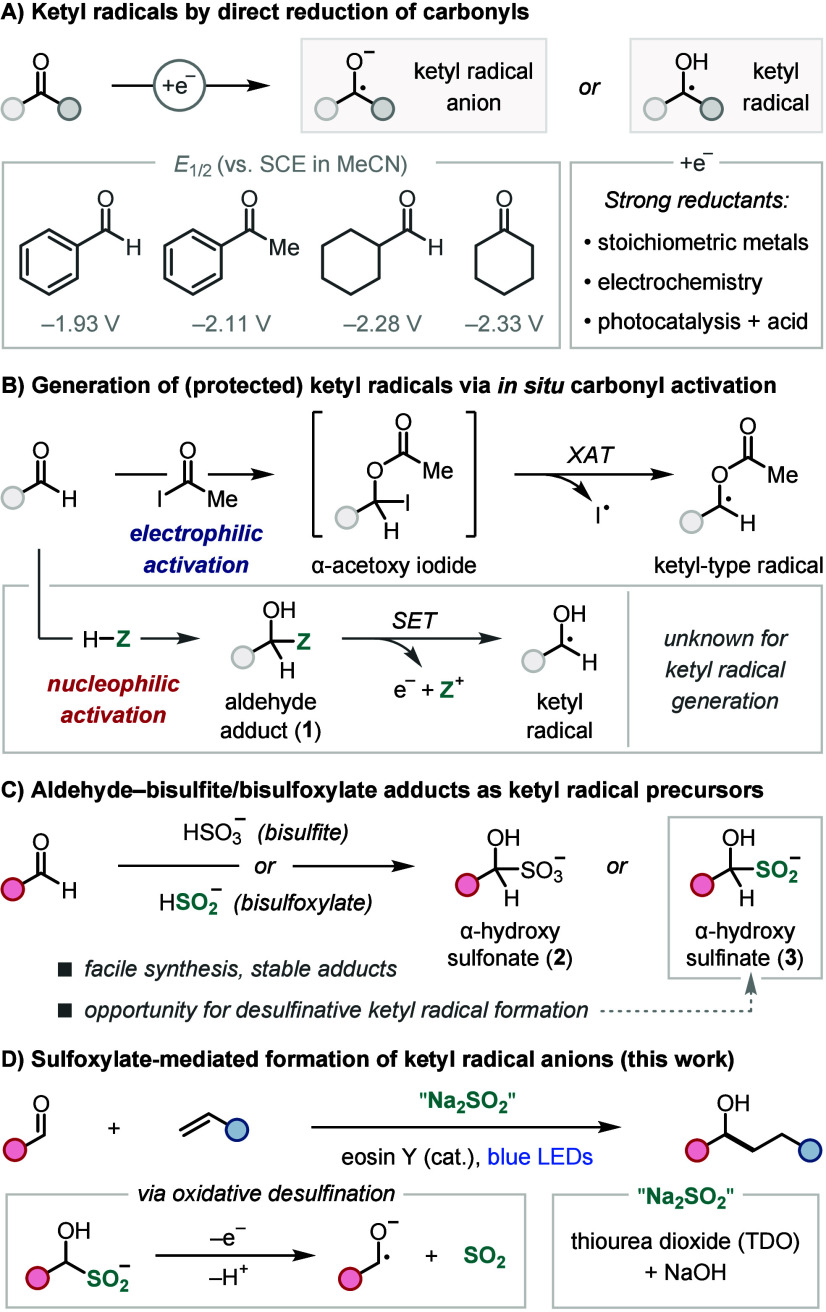
Ketyl Radicals Via Single-Electron Reduction and *In Situ* Aldehyde Activation

The use of visible-light photocatalysis has recently led to significant progress in the catalytic generation of ketyl radicals.^7^ For example, aromatic aldehydes and ketones can be reduced to ketyl radicals in the presence of Brønsted or Lewis acids, which facilitate the challenging single-electron transfer (SET) through proton-coupled electron transfer (PCET)^8^ or carbonyl–metal complexation.^9^ In addition, highly reducing excited state organic radicals have been shown to promote ketyl radical formation from aliphatic carbonyls.^10^ Despite these advances, this single-electron reduction approach to ketyl radicals still suffers from limitations because of the requirement for highly reducing conditions, inevitably leading to issues with functional group compatibility.

An alternative approach to ketyl radicals from aldehydes that avoids the challenging single-electron reduction is via *in situ* activation, wherein aldehydes are initially converted to adducts that are more amenable to catalytic radical formation (Scheme 1B).^11^ For example, Nagib and co-workers showed that α-acetoxy iodides, formed by electrophilic activation of aldehydes with acetyl iodide, undergo efficient photoinduced halogen-atom transfer (XAT) to generate α-acetoxy radicals,^11^ which are protected ketyl (or ketyl-type) radicals (Scheme 1B, top).^12^ However, the instability of α-hydroxy halides means this XAT process is only applicable to the synthesis of ketyl-type radicals,^13^ whereas there are no reports of related *in situ* carbonyl activation to generate unprotected ketyl radicals or ketyl radical anions.^14^ Nonetheless, such a strategy would expand the scope of ketyl radical chemistry by avoiding the strongly reducing conditions of the single-electron reduction pathway and circumventing the use of the highly electrophilic activators required for XAT-mediated formation of ketyl-type radicals.

We envisioned an alternative *in situ* activation strategy to generate unprotected ketyl radicals from aldehydes using a nucleophilic redox activator, HZ (Scheme 1B, bottom). Here, initial addition of HZ provides redox-active aldehyde adduct **1**, which could undergo SET-mediated C–Z bond cleavage to generate a ketyl radical. Inspiration for the nucleophilic redox activator came from the established reactivity of aldehydes with bisulfite salts to form stable aldehyde–bisulfite adducts **2** (Scheme 1C).^15^ However, given the lack of precedent for radical desulfonation of alkyl sulfonates, we instead turned to the less well-known α-hydroxy sulfinates **3**. Importantly, these adducts can easily be prepared by analogous reactions of aldehydes with bisulfoxylate salts,^16^ and alkyl sulfinates possess low oxidation potentials, enabling facile oxidative desulfination,^17^ which makes them attractive ketyl radical precursors. Herein, we report the successful development of this sulfoxylate strategy for ketyl radical formation from aldehydes and demonstrate its application in photoredox-catalyzed ketyl–olefin coupling reactions (Scheme 1D).^8e−8g^ It is important to note that this novel approach utilizes inexpensive reagents, a cheap organic photocatalyst, and avoids the strongly reducing conditions needed for traditional reductive ketyl radical formation.

We began our studies by investigating the photoredox-catalyzed ketyl–olefin coupling reaction of benzaldehyde (**4**) with 4-vinylpyridine (**5**) in the presence of sodium sulfoxylate (Na_2_SO_2_), which was generated *in situ* through NaOH-mediated hydrolysis of thiourea dioxide (TDO) (Table 1).^18^ Analysis of this mixture in D_2_O/MeCN-*d*_3_ by ^1^H NMR showed that **4** was quantitatively converted to the corresponding α-hydroxy sulfinate within 60 min.^19^ To our delight, when α-hydroxy sulfinate formation was performed in the presence of the water-soluble organic photocatalyst eosin Y, subsequent irradiation with blue LEDs (Kessil Tuna Blue) led to a 74% yield of the ketyl–olefin coupling product as a 4:1 mixture of **6a** and the SO_2_-incorporated product **6b** (entry 1).^19^ While sulfinate **6b** was observed in small amounts during our optimization studies, it was readily converted to **6a** by silica-mediated protodesulfination. Control experiments demonstrated that photocatalyst, light, and TDO are all essential for this transformation (entries 2–4). The reaction was also successfully catalyzed by Ru(bpy)_3_Cl_2_, although with a lower yield of 61% (entry 5). A high yield was also obtained using only water as the solvent (entry 6); however, we found that using MeCN as a cosolvent was beneficial for substrates with poor water solubility. Lowering the amount of NaOH reduced the yield of **6a**/**6b** to 56% (entry 7). Finally, we investigated the impact of the prestirring procedure, which was routinely carried out before photoirradiation to preform the α-hydroxy sulfinate; however, a similar yield was achieved without (entry 8).

**Table 1:**
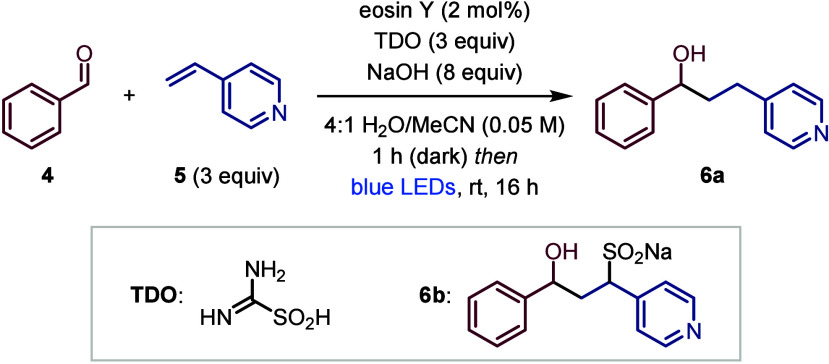
Optimization Studies^a^

Entry	Variation from above conditions	Yield (%)^b^
1	none	74
2	without eosin Y	0
3	without light	0
4	without TDO	0
5	Ru(bpy)_3_Cl_2_ instead of eosin Y	61
6	H_2_O as the solvent	70
7	4.0 equiv NaOH	56
8	without prestirring	73

a Reactions performed using 0.1 mmol of **4**.

b Yields are the sum of **6a** and **6b** and were determined by ^1^H NMR analysis using an internal standard.

With the optimized conditions in hand, we proceeded to investigate the scope of aldehydes in the reaction with **5** (Scheme 2). In addition to benzaldehyde (**6a**), extended aromatic systems, including biphenyl (**7**) and 2-naphthyl (**8**) could be used, despite their reduced water solubility. The synthesis of **6a** was reproduced on a larger scale (2 mmol) without significant reduction in yield. A broad range of benzaldehydes were successfully coupled, including those functionalized with ethers (**9**-**10**), amides (**11**-**12**), amines (**13**-**15**), halides (**16**-**18**), and perfluoroalkyl groups (**19**-**20**). Of note, aldehydes with strongly electron-donating *para* tertiary amines, such as piperidine (**13**), morpholine (**14**) and Boc-protected piperazine (**15**), which would be challenging to reduce to ketyl radicals via a reduction pathway, reacted efficiently under our oxidative desulfination conditions. Synthetically useful oxygen-containing functional groups, including benzylic alcohols (**21**), phenols (**22**), and carboxylic acids (**23**) were also tolerated. Heteroaromatic aldehydes could also be used, giving furan **24** and pyridine **25** in good yields. Finally, extension to ketones was demonstrated through coupling of acetophenone, which provided tertiary alcohol **26** in 27% yield. The lower yield for this substrate is likely due to a less favorable addition of the sulfoxylate anion to the more sterically hindered ketone. While aliphatic aldehydes were found to react with Na_2_SO_2_ to generate α-hydroxy sulfinates, they failed to undergo productive ketyl–olefin coupling.

**Scheme 2:**
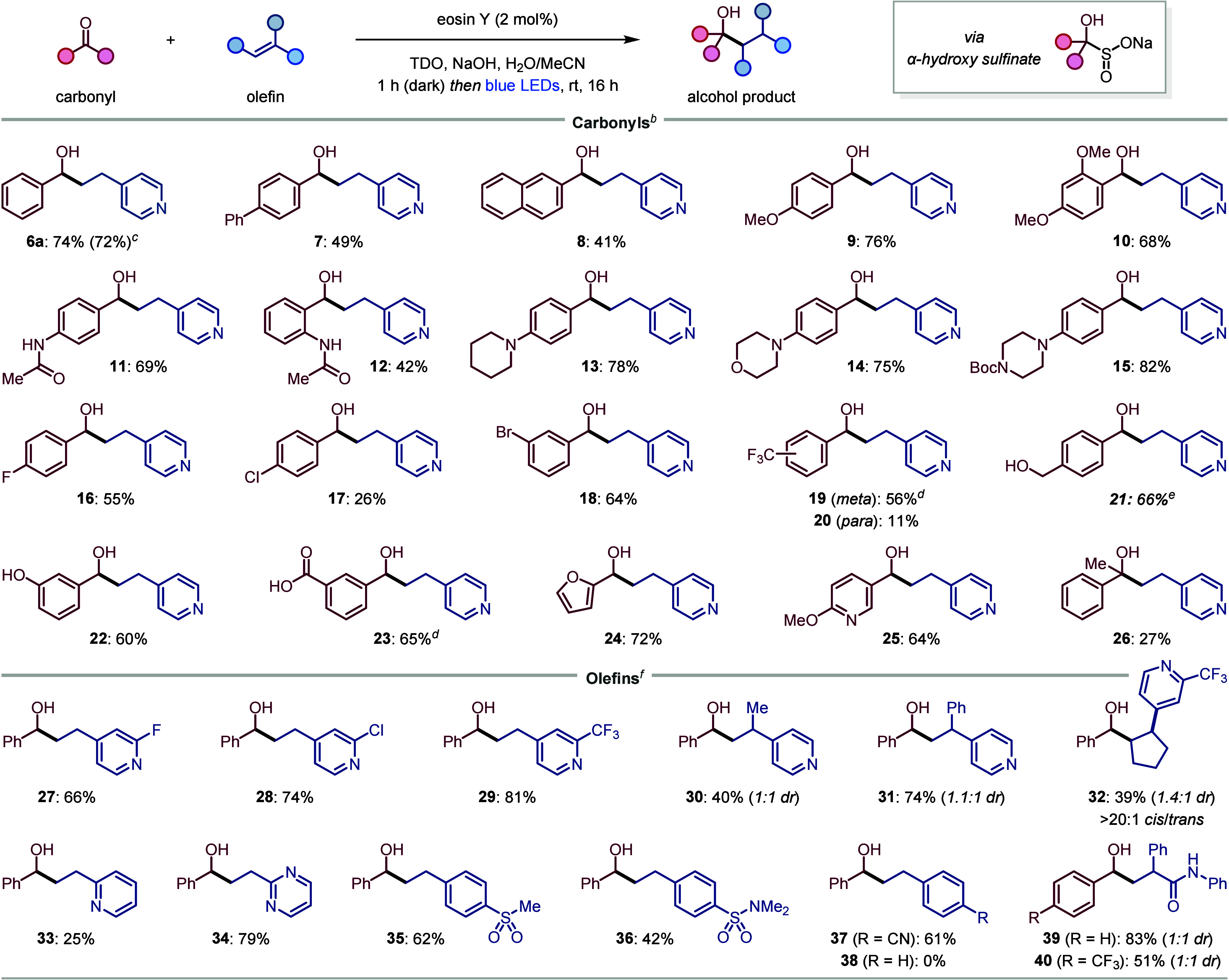
Ketyl–Olefin Coupling Scope Reactions performed using the carbonyl (0.25 mmol), olefin (1.5–3 equiv), TDO (1.5–3 equiv), and NaOH (4–8 equiv) in H_2_O/MeCN (0.05 M). Yields are of isolated products after purification. Using olefin (3 equiv), TDO (3 equiv), and NaOH (8 equiv) in H_2_O/MeCN (4:1, 0.05 M). Using 2.0 mmol of aldehyde. Using sodium dithionite (Na_2_S_2_O_4_) instead of TDO and THF instead of MeCN. Using THF instead of MeCN. Using olefin (1.5 equiv), TDO (1.5 equiv), and NaOH (4 equiv) in H_2_O/MeCN (1:1, 0.05 M).

Next, the olefin scope was investigated with **4** as the ketyl radical precursor and using slightly modified conditions with a 1:1 ratio H_2_O/MeCN to ensure dissolution of the less water-soluble olefins (Scheme 2). 4-Vinylpyridines bearing a range of substituents gave moderate to high yields of the alcohol products, including halogen (**27**-**28**), trifluoromethyl (**29**), α-methyl (**30**), and α-phenyl (**31**) groups. A cyclopentenylpyridine demonstrated the coupling could be extended to trisubstituted olefins, affording the *cis*-disubstituted cyclopentane **32** in moderate yield. A low yield was obtained with 2-vinylpyridine (**33**), which we attribute to the 2-pyridyl group being less electron-withdrawing compared to 4-pyridyl.^20^ Nonetheless, the successful formation of **33** is notable considering that all previous reports of ketyl radical reactions with 2-vinylpyridine required additional Lewis or Brønsted acid activation of the pyridine group.^8f,8g^ Pleasingly, 2-vinyl pyrimidine was an excellent coupling partner, providing **34** in 79% yield. Finally, styrenes were also compatible coupling partners, although electron-withdrawing substituents on the aromatic ring (**35**-**37**) or the alkene (**39**) were found to be essential, with simple styrene (**38**) failing to couple, which highlights the significant influence the electrophilicity of the olefin has on the reaction. In addition, the nucleophilicity of the ketyl radical was also found to be important, as demonstrated by the inefficient coupling of 4-trifluoromethyl benzaldehyde with 4-vinylpyridine (**20**), which reflects the diminished nucleophilicity caused by the electron-withdrawing CF_3_ group. However, we found that this lower reactivity could be counteracted by using the more electrophilic olefin *N*,2-diphenylacrylamide, giving alcohol **40** in 51% yield.

We subsequently explored intramolecular ketyl–olefin couplings of olefin-tethered aldehydes (Scheme 3).^21^ Although our initial attempts proved unsuccessful due to the poor solubility of the substrates in MeCN/H_2_O, we found using tetrabutylammonium hydroxide in place of NaOH ensured dissolution and allowed the reactions to proceed. The intramolecular coupling was applied to the synthesis of *N*-acetyl tetrahydroquinolines **41** and **42**, and indanes **43** and **44**, the latter of which were generated in high diastereoselectivities.^13b,21a^ While the intermolecular reactions were unsuccessful with unsubstituted styrenes (see **38**, Scheme 2), the intramolecular couplings did not require additional electron-withdrawing groups (**41**, **43**) due to the faster rate of addition of the ketyl radical anion to the olefin. Interestingly, the cyclization also proceeded with an unprotected secondary amine linkage, however, tetrahydroquinoline **45** was not observed due to unexpected oxidation to quinoline **46** under the reaction conditions.

**Scheme 3:**
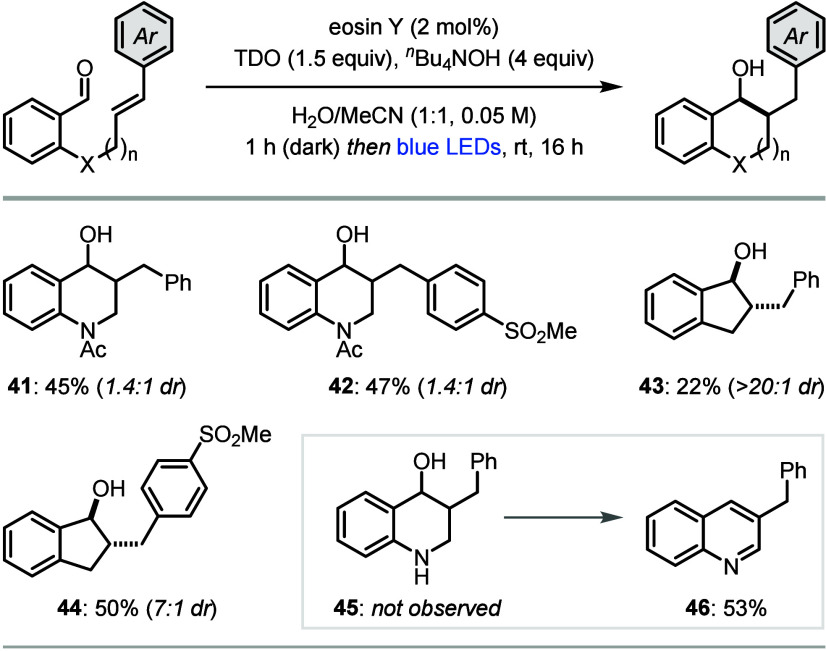
Intramolecular Ketyl–Olefin Couplings Reactions performed on a 0.25 mmol scale. Yields are of isolated products after purification.

To provide support for our proposed sulfoxylate-mediated ketyl radical formation, we isolated benzaldehyde-derived α-hydroxy sulfinate **3a** (Scheme 4a) and performed electrochemical and fluorescence quenching studies. Cyclic voltammetry confirmed that **3a** has a low oxidation potential of (*E*_p/2_ = 0.33 V vs SCE in H_2_O/MeCN) and, therefore, can undergo exergonic SET with the excited state disodium salt of eosin Y (*E*_1/2_ [Na_2_EY*/Na_2_EY^•–^] = 0.83 V in H_2_O/MeCN).^22^ Unfortunately, fluorescence quenching studies with Na_2_EY and **3a** were complicated by an unexpected ground state sulfoxylate transfer reaction.^19^ Therefore, we used Ru(bpy)_3_Cl_2_ in place of Na_2_EY, since it was also a competent photocatalyst in the ketyl–olefin coupling reaction but displayed no ground state reactivity with **3a**. With this catalyst, we observed efficient quenching with **3a** under the basic reaction conditions (Scheme 4B). Interestingly, no quenching occurred in the absence of NaOH, which suggests that PCET, or initial deprotonation of **3a** before SET, could be involved, leading to the formation of a ketyl radical anion rather than a neutral ketyl radical.^23^ Using a mixture of benzaldehyde, TDO, and NaOH led to similar levels of quenching, which provides further support for *in situ* formation of **3a**. Crucially, no quenching was observed with benzaldehyde (**4**), 4-vinylpyridine (**5**), or Na_2_SO_2_ (TDO/NaOH), confirming the key role of the α-hydroxy sulfinate in the photoredox catalysis.

**Scheme 4:**
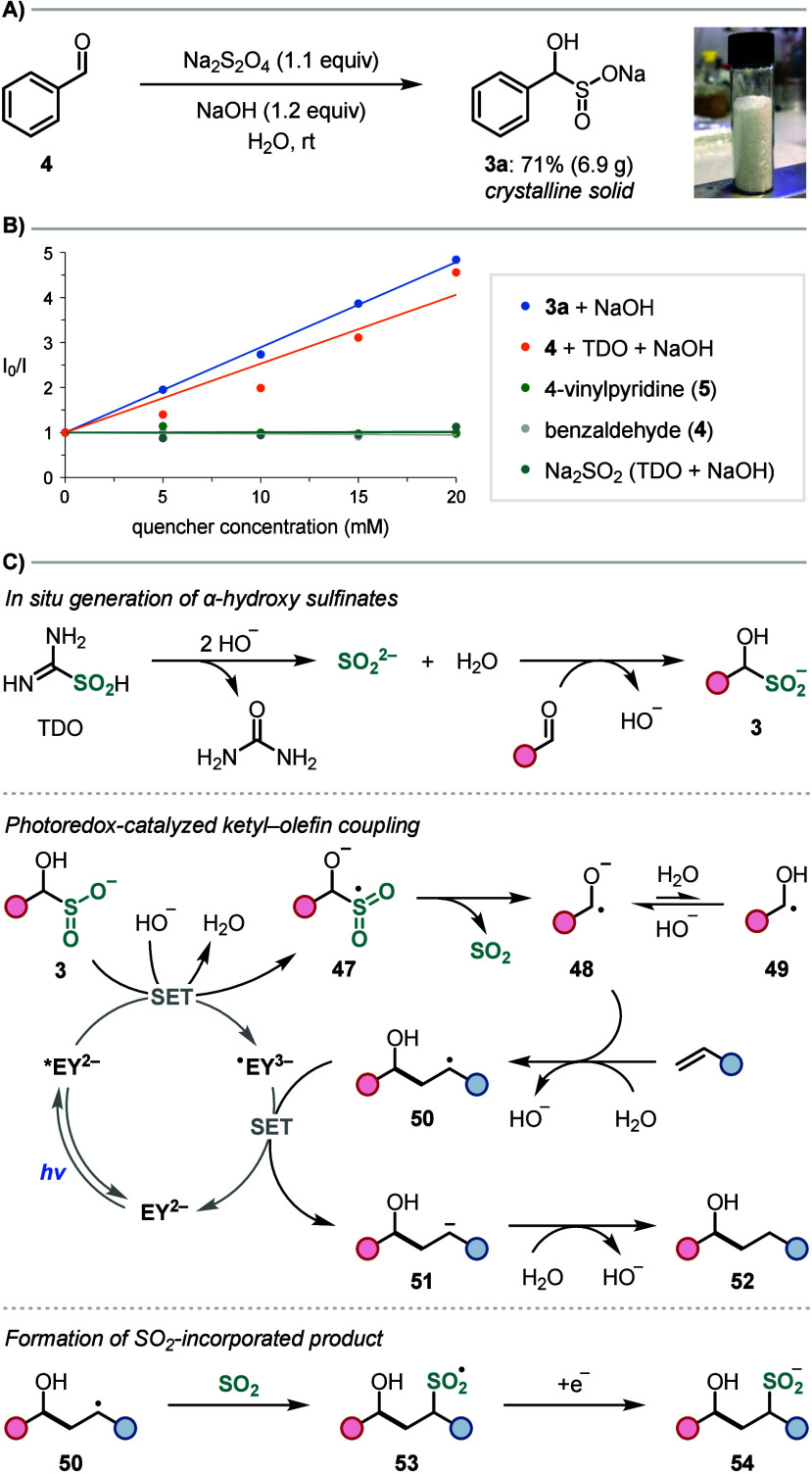
Mechanistic Studies and Proposed Mechanism

Based on the above, we propose the mechanism shown in Scheme 4C. Reaction of TDO with hydroxide provides SO_2_^2–^, which adds to the aldehyde to generate α-hydroxy sulfinate **3**. This redox-active adduct is oxidized by photoexcited eosin Y (*EY^**2–**^) in the presence of hydroxide to afford sulfonyl radical **47**, which undergoes SO_2_ extrusion to generate ketyl radical anion **48**. Based on the relatively high acidity of ketyl radicals (p*K*_a_ ∼ 8),^23^ the basic reaction conditions should favor radical anion **48** over neutral ketyl radical **49**. Addition of **48** to the olefin and alkoxide protonation gives alkyl radical **50**, which is reduced to carbanion **51** to turnover the photocatalyst (*E*_1/2_ [Na_2_EY/Na_2_EY^•–^] = −1.06 V in H_2_O/MeCN),^22^ before protonation provides coupled product **52**. The formation of small amounts of SO_2_-incorporated product **54** (see **6b**, Table 1) suggests that an alternative pathway involving sulfination of **50** followed by single-electron reduction of sulfonyl radical **53** is also operative.^24^

In conclusion, we have developed a mechanistically novel method for the generation of ketyl radicals from aldehydes that uses sulfoxylate as a traceless redox activator. Through facile *in situ* formation and subsequent photoredox-catalyzed oxidative desulfination of α-hydroxy sulfinates, ketyl radical anions can be generated without the strongly reducing conditions that are commonly required. This strategy, which proceeds under mild conditions and uses inexpensive reagents, was successfully applied to both inter- and intramolecular ketyl–olefin couplings of a diverse range of aromatic aldehydes, thus demonstrating its potential as a valuable alternative to traditional reductive ketyl radical formation.

## Supporting Information Available

Experimental procedures, characterization data, and spectra (PDF)
