# Supplementary material for: Aldehyde–Olefin Couplings Via Sulfoxylate-Mediated Oxidative Generation of Ketyl Radical Anions
Source: J Am Chem Soc. 2024 Sep 20;146(39):26616–21. doi: 10.1021/jacs.4c10093 (PMC11450749; doi:10.1021/jacs.4c10093)
Supplement: Supplementary file 1 — ja4c10093_si_001.pdf [file ja4c10093_si_001.pdf]

SUPPORTING INFORMATION

Aldehyde–Olefin Couplings via Sulfoxylate-Mediated Oxidative Generation of Ketyl Radical Anions

Zhihang Li,[a] Joseph A. Tate,[b] and Adam Noble[a]*

[a] *School of Chemistry, University of Bristol, Cantock's Close, Bristol, BS8 1TS, United Kingdom*

[b] *Syngenta, Jealott's Hill International Research Centre, Bracknell, RG42 6EY, United Kingdom*

*e-mail: a.noble@bristol.ac.uk

TABLE OF CONTENTS

LIST OF SUPPLEMENTARY SCHEMES, FIGURES AND TABLES 2

LIST OF CHARACTERISED PRODUCTS 2

1. MATERIALS AND GENERAL METHODS 4
   1.1. Glassware, Solvents and Reagents 4
   1.2. Chromatography and Instrumentation 4
   1.3. Naming of Compounds 5
   1.4. Photochemical Equipment and Setup 5
      1.4.1. Small Scale Setup 5
      1.4.2. Larger Scale Setup 6

2. EXPERIMENTAL DATA 7
   2.1. General Procedures 7
      2.1.1. General Procedure A: Aldehyde Scope 7
      2.1.2. General Procedure B: Olefin Scope 7
      2.1.3. General Procedure C: Intramolecular Cyclization Scope 7
      2.1.4. Work-Up Procedure 8
   2.2. Reaction Optimization 9
      2.2.1. Isolated α-Hydroxy Sulfinate 9
      2.2.2. *In Situ* Generated α-Hydroxy Sulfinate 11
   2.3. Synthesis of Starting Materials 14
      2.3.1. Synthesis of α-Hydroxy Sulfinates 14
      2.3.2. Synthesis of Aldehydes 14
      2.3.3. Synthesis of Olefins 15
      2.3.4. Synthesis of Starting Materials for Intramolecular Scope 22
   2.4. Substrate Scope 28
      2.4.1. Aldehyde Scope 28
      2.4.2. Olefin Scope 41
      2.4.3. Intramolecular Cyclization Scope 49
      2.4.4. Scale-up Reaction (2.0 mmol) 53
      2.4.5. Unsuccessful and Low Yielding Substrates 54

3. MECHANISTIC STUDIES 55
   3.1. Cyclic Voltammetry 55
   3.2. Fluorescence Quenching Studies 56
      3.2.1. Quenching Studies with $Ru(bpy)_3Cl_2$ 56
      3.2.2. Quenching Studies with Na_2EY 61

4. SPECTROSCOPIC DATA 65

5. REFERENCES 133

LIST OF SUPPLEMENTARY SCHEMES, FIGURES AND TABLES

Figure S1. Photochemical reaction setup (0.25 mmol scale) 5
Figure S2. Photochemical reaction setup (2 mmol scale) 6
Table S1: Photocatalyst screening 9
Table S2. Base equivalents screening 10
Table S3. Co-solvent screening 10
Scheme S1. *In situ* generation of α-hydroxy sulfinate **3a** 11
Scheme S2. One-pot transformation of *in situ* generated α-hydroxy sulfinate **3a** 12
Table S4: Reaction optimization with *in situ* generated α-hydroxy sulfinate 13
Figure S3. Cyclic voltammogram of α-hydroxy sulfinate **3a** 55
Figure S4. Stern-Volmer plot of fluorescence quenching of $Ru(bpy)_3Cl_2$ by isolated α-hydroxy sulfinate **3a** without NaOH 56
Figure S5. Stern-Volmer plot of fluorescence quenching of $Ru(bpy)_3Cl_2$ by α-hydroxy sulfinate **3a** (10 mM) with increasing equivalents of NaOH 57
Figure S6. Stern-Volmer plot of fluorescence quenching of $Ru(bpy)_3Cl_2$ by α-hydroxy sulfinate **3a** with NaOH (50 mM) 57
Figure S7. Stern-Volmer plot of fluorescence quenching of $Ru(bpy)_3Cl_2$ by *in situ* generated α-hydroxy sulfinate with NaOH (50 mM) 58
Figure S8. Stern-Volmer plot of fluorescence quenching of $Ru(bpy)_3Cl_2$ by benzaldehyde 59
Figure S9. Stern-Volmer plot of fluorescence quenching of $Ru(bpy)_3Cl_2$ by 4-vinylpyridine 59
Figure S10. Stern-Volmer plot of fluorescence quenching of $Ru(bpy)_3Cl_2$ by TDO with NaOH (equiv NaOH) . 60
Figure S11. Stern-Volmer plot of fluorescence quenching of $Ru(bpy)_3Cl_2$ by TDO (10 mM) with increasing equivalents of NaOH 60
Figure S12. Stern-Volmer plot of fluorescence quenching of Na_2EY by α-hydroxy sulfinate **3a** 61
Figure S13. UV-Vis absorption spectra of Na_2EY with added α-hydroxy sulfinate **3a** 62
Scheme S3. Sulfoxylate transfer reaction between Na_2EY and **3a** 62
Scheme S4. Addition of Na_2SO_2 to Na_2EY 63
Figure S14. ^1H NMR spectra of Na_2EY with and without Na_2SO_2 63
Figure S15. Stern-Volmer plot of fluorescence quenching of Na_2EY by benzaldehyde 64
Figure S16. Stern-Volmer plot of fluorescence quenching of Na_2EY by 4-vinylpyridine 64

LIST OF CHARACTERISED PRODUCTS

Hydroxy(phenyl)methanesulfinate (**3a**) 14
N-(2-Formylphenyl) acetamide 14
2-Fluoro-4-vinylpyridine 15
2-Chloro-4-vinylpyridine 16
2-(Trifluoromethyl)-4-vinylpyridine 16
4-(Prop-1-en-2-yl)pyridine 17
4-(1-Phenylvinyl)pyridine 18
4-(Cyclopent-1-en-1-yl)-2-(trifluoromethyl)pyridine (**S1**) 18
2-Vinylpyrimidine 19
1-(Methylsulfonyl)-4-vinylbenzene 20
N,*N*-Dimethyl-4-vinylbenzenesulfonamide 20
N,2-Diphenylacrylamide 21

N-Cinnamyl-*N*-(2-formylphenyl) acetamide (**S2**) 22
(*E*)-1-(3-Bromoprop-1-en-1-yl)-4-(methylsulfonyl)benzene (**S5**) 23
(*E*)-*N*-(2-Formylphenyl)-*N*-(3-(4-(methylsulfonyl)phenyl)allyl)acetamide (**S6**) 24
2-Cinnamylbenzaldehyde (**S7**) 25
(*E*)-2-(3-(4-(Methylsulfonyl)phenyl)allyl)benzaldehyde (**S8**) 26
2-(Cinnamylamino)benzaldehyde (**S10**) 27
1-Phenyl-3-(pyridin-4-yl)propan-1-ol (**6a**) 28
1-([1,1'-Biphenyl]-4-yl)-3-(pyridin-4-yl)propan-1-ol (**7**) 28
1-(Naphthalen-2-yl)-3-(pyridin-4-yl)propan-1-ol (**8**) 29
1-(4-Methoxyphenyl)-3-(pyridin-4-yl)propan-1-ol (**9**) 30
1-(2,4-Dimethoxyphenyl)-3-(pyridin-4-yl)propan-1-ol (**10**) 30
N-(4-(1-Hydroxy-3-(pyridin-4-yl)propyl)phenyl)acetamide (**11**) 31
N-(2-(1-Hydroxy-3-(pyridin-4-yl)propyl)phenyl)acetamide (**12**) 31
1-(4-(Piperidin-1-yl)phenyl)-3-(pyridin-4-yl)propan-1-ol (**13**) 32
1-(4-Morpholinophenyl)-3-(pyridin-4-yl)propan-1-ol (**14**) 33
tert-Butyl 4-(4-(1-hydroxy-3-(pyridin-4-yl)propyl)phenyl)piperazine-1-carboxylate (**15**) 33
1-(4-Fluorophenyl)-3-(pyridin-4-yl)propan-1-ol (**16**) 34
1-(4-Chlorophenyl)-3-(pyridin-4-yl)propan-1-ol (**17**) 34
1-(3-Bromophenyl)-3-(pyridin-4-yl)propan-1-ol (**18**) 35
3-(Pyridin-4-yl)-1-(3-(trifluoromethyl)phenyl)propan-1-ol (**19**) 36
3-(Pyridin-4-yl)-1-(4-(trifluoromethyl)phenyl)propan-1-ol (**20**) 36
1-(4-(Hydroxymethyl)phenyl)-3-(pyridin-4-yl)propan-1-ol (**21**) 37
3-(1-Hydroxy-3-(pyridin-4-yl)propyl)phenol (**22**) 38
3-(1-Hydroxy-3-(pyridin-4-yl)propyl)benzoic acid (**23**) 38
1-(Furan-2-yl)-3-(pyridin-4-yl)propan-1-ol (**24**) 39
1-(6-Methoxypyridin-3-yl)-3-(pyridin-4-yl)propan-1-ol (**25**) 39
2-Phenyl-4-(pyridin-4-yl)butan-2-ol (**26**) 40
3-(2-Fluoropyridin-4-yl)-1-phenylpropan-1-ol (**27**) 41
3-(2-Chloropyridin-4-yl)-1-phenylpropan-1-ol (**28**) 41
1-Phenyl-3-(2-(trifluoromethyl)pyridin-4-yl)propan-1-ol (**29**) 42
1-Phenyl-3-(pyridin-4-yl)butan-1-ol (**30**) 43
1,3-Diphenyl-3-(pyridin-4-yl)propan-1-ol (**31**) 43
Phenyl(2-(2-(trifluoromethyl)pyridin-4-yl)cyclopentyl)methanol (**32**) 44
1-Phenyl-3-(pyridin-2-yl)propan-1-ol (**33**) 45
1-Phenyl-3-(pyrimidin-2-yl)propan-1-ol (**34**) 45
3-(4-(Methylsulfonyl)phenyl)-1-phenylpropan-1-ol (**35**) 46
4-(3-Hydroxy-3-phenylpropyl)-*N*,*N*-dimethylbenzenesulfonamide (**36**) 46
4-(3-Hydroxy-3-phenylpropyl)benzonitrile (**37**) 47
4-Hydroxy-*N*,2,4-triphenylbutanamide (**39**) 47
4-Hydroxy-*N*,2-diphenyl-4-(4-(trifluoromethyl)phenyl)butanamide (**40**) 48
1-(3-Benzyl-4-hydroxy-3,4-dihydroquinolin-1(2*H*)-yl)ethan-1-one (**41**) 49
1-(4-Hydroxy-3-(4-(methylsulfonyl) benzyl)-3,4-dihydroquinolin-1(2*H*)-yl)ethan-1-one (**42**) 49
(1*S**,2*R**)-2-Benzyl-2,3-dihydro-1*H*-inden-1-ol (**43**) 50
(1*S**,2*R**)-2-(4-(Methylsulfonyl)benzyl)-2,3-dihydro-1*H*-inden-1-ol (**44**) 51
3-Benzylquinoline (**46**) 51

1. MATERIALS AND GENERAL METHODS

1.1. Glassware, Solvents and Reagents

All anhydrous solvents were supplied by an Anhydrous Engineering alumina column drying system [tetrahydrofuran (THF), Et_2O, dichloromethane (DCM)] and stored over 4 Å molecular sieves. All reagents were purchased from commercial sources [Fluorochem Ltd, Sigma Aldrich (Merck), Fischer, TCI, etc.] and were used as received. Water (HPLC grade), MeCN and THF [Sigma Aldrich (Merck)] were used for the photoredox reactions.

Photoredox reactions were performed in glass vials (7 mL) sealed with B10 Suba-Seals.

1.2. Chromatography and Instrumentation

Thin layer chromatography (TLC) was performed using Merck Kieselgel 60 F254 fluorescent treated silica, which was visualised under UV light, or by staining with aqueous basic potassium permanganate followed by heating, or by staining with Hanessian's stain (CAM stain) followed by heating.

Flash column chromatography (FCC) was carried out using Sigma-Aldrich silica gel (60 Å, 230–400 mesh, 40–63 µm).

NMR spectra were recorded at various field strengths, as indicated, using Bruker 400 MHz, Varian VNMR 400 MHz, Varian VNMR 500 MHz for ^{1}H, ^{13}C and ^{19}F acquisitions. All NMR spectra were recorder at 25 °C unless otherwise stated. Chemical shifts (δ) are reported in parts per million (ppm) and referenced to $CDCl_3$ (^{1}H: 7.26 ppm; ^{13}C: 77.0 ppm) or CD_3OD (^{1}H: 3.31 ppm; ^{13}C: 49.00 ppm) or D_2O (^{1}H: 4.79 ppm). Coupling constants (*J*) are given in Hertz (Hz) and refer to apparent multiplicities (s = singlet, d = doublet, t = triplet, q = quartet, p = pentet, hex = hextet, h = heptet, m = multiplet, br = broad signal, dd = doublet of doublets, etc.). The ^{1}H NMR spectra are reported as follows: chemical shift (multiplicity, coupling constants, number of protons).

High resolution mass spectra (HRMS) were recorded on a Bruker Daltonics MicrOTOF II by Electrospray Ionisation (ESI); a Thermo Scientific QExactive by Electron Ionisation (EI); a Thermo Scientific Orbitrap Elite by ESI or Atmospheric Pressure Chemical Ionisation (APCI); or a Bruker UltrafleXtreme by Matrix-assisted Laser Desorption/Ionisation (MALDI).

IR spectra were recorded neat as a thin film on a Perkin Elmer Spectrum One FT-IR. Selected absorption maxima (ν_{max}) are reported in wavenumbers (cm^{-1}).

Cyclic voltametric (CV) experiments were performed at room temperature using MultiPalmSens 4. CV analysis was conducted with a working electrode (glassy carbon), a counter electrode (platinum wire) and a reference electrode ($Ag/AgNO_3$ (0.1 M)).

Luminescence Quenching Studies were performed at room temperature (25 °C) using FluoroMax spectrometer.

UV-Vis absorption spectra were recorded using an Agilent Technologies Cary 300 UV/Vis spectrophotometer, in quartz cuvettes with a path length of 10 mm.

1.3. Naming of Compounds

Compound names are those generated by ChemDraw Professional 20.0 software (PerkinElmer), following the IUPAC nomenclature.

1.4. Photochemical Equipment and Setup

1.4.1. Small Scale Setup

A 40 W Kessil A160WE Tuna Blue LED lamp was used for all the photoredox reactions, with the color dial turned fully anticlockwise, and the intensity dial turned fully clockwise.

The holder for reaction vials was adapted from Leonori and co-workers (Figure S1).[1] All photoredox reactions were carried out at room temperature (rt, 25-30 °C) with assistance of fan cooling. The stirring rate was set to 1200 rpm. The distance between the lamp and the vial was approximately 5 cm.

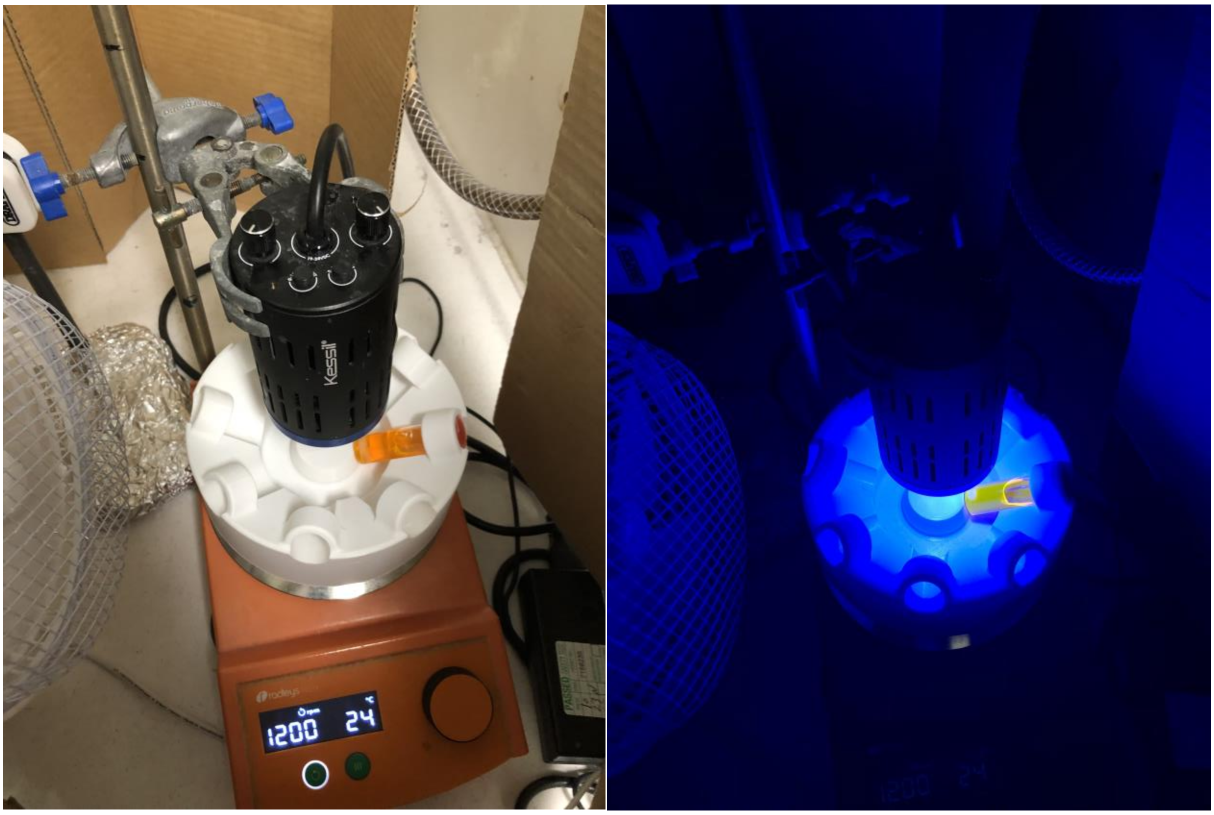

Figure S1. Photochemical reaction setup (0.25 mmol scale)

1.4.2. Larger Scale Setup

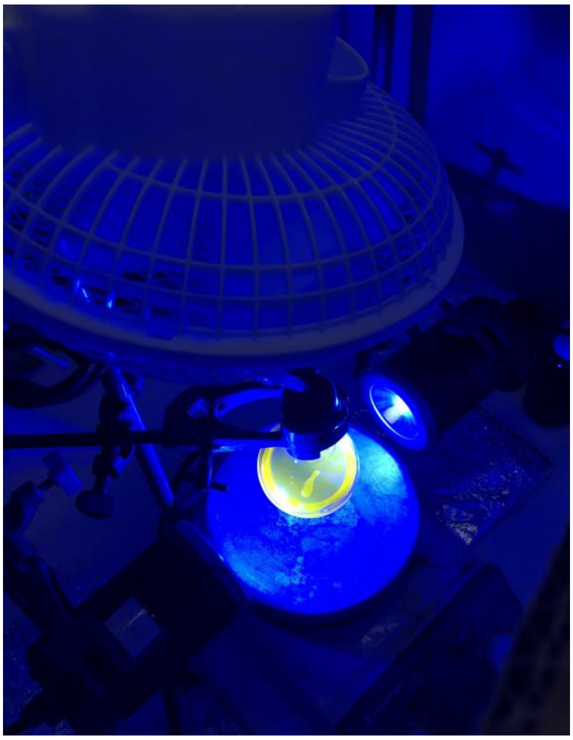

Figure S2. Photochemical reaction setup (2 mmol scale)

Two 40 W Kessil A160WE Tuna Blue LED lamps were used for all the photoredox reactions, with the color dial turned fully anticlockwise, and the intensity dial turned fully clockwise.

The reaction was carried out at room temperature (rt, 25-30 °C) with assistance of fan cooling. The stirring rate was set to 400 rpm. The distance between the lamp and the round bottom flask was 5 cm.

2. EXPERIMENTAL DATA

2.1. General Procedures

2.1.1. General Procedure A: Aldehyde Scope

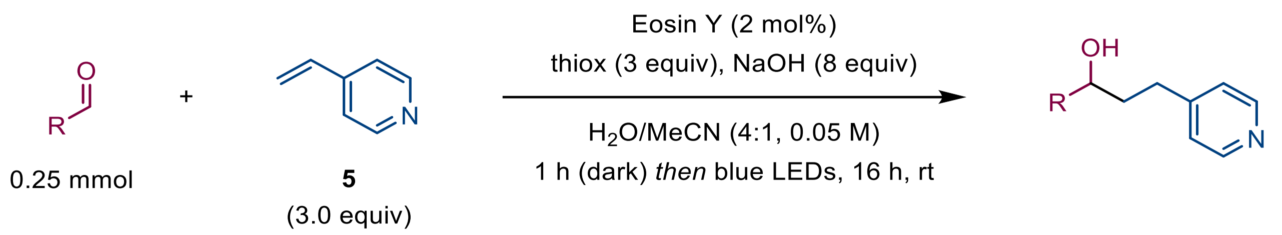

A 7 mL vial was charged with eosin Y (3.2 mg, 5.0 µmol, 2.0 mol%), thiourea dioxide (TDO, 81 mg, 0.75 mmol, 3.0 equiv) and aldehyde (if solid, 0.25 mmol, 1.0 equiv). MeCN (1.0 mL), H_2O (3.5 mL) and NaOH (4 M in H_2O, 0.50 mL, 2.0 mmol, 8.0 equiv) were then added to the vial. Subsequently, the solution was degassed by sparging with N_2 for 5 min before addition of aldehyde (if liquid) and 4-vinylpyridine (80 µL, 0.75 mmol, 3.0 equiv). The mixture was then stirred in the dark for 1 h before irradiation with blue LEDs at room temperature for 16 h.

2.1.2. General Procedure B: Olefin Scope

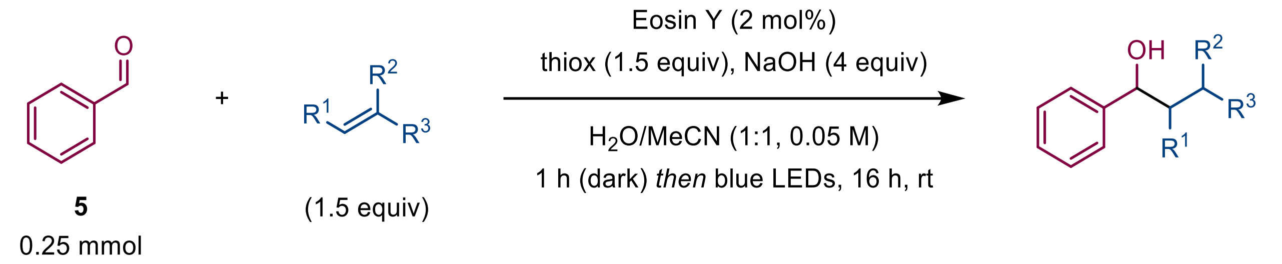

A 7 mL vial was charged with eosin Y (3.2 mg, 5.0 µmol, 2.0 mol%), TDO (41 mg, 0.38 mmol, 1.5 equiv) and olefin (if solid, 0.38 mmol, 1.5 equiv). MeCN (2.5 mL), H_2O (2.25 mL) and NaOH (4 M in H_2O, 0.25 mL, 1.0 mmol, 4.0 equiv) were then added to the vial. Subsequently, the solution was degassed by sparging with N_2 for 5 min before addition of benzaldehyde (25.5 µL, 0.25 mmol, 1.0 equiv) and olefin (if liquid). The mixture was then stirred in the dark for 1 h before irradiation with blue LEDs at room temperature for 16 h.

2.1.3. General Procedure C: Intramolecular Cyclization Scope

A 7 mL vial was charged with eosin Y (3.2 mg, 5.0 µmol, 2.0 mol%), TDO (41 mg, 0.38 mmol, 1.5 equiv) and substrate (0.25 mmol, 1 equiv). MeCN (2.5 mL), H_2O (1.87 mL) and tetra-*n*-butylammonium hydroxide (^{n}Bu$_4$NOH, 1.6 M in H_2O, 0.63 mL, 1.0 mmol, 4.0 equiv) were then added to the vial. Subsequently, the solution was degassed by sparging with N_2 for 5 min and the mixture was then stirred in the dark for 1 h before irradiation with blue LEDs at room temperature for 16 h. The solvent was removed *in vacuo* to afford the crude product, which was purified by flash column chromatography.

2.1.4. Work-Up Procedure

The reaction mixture was diluted with MeOH and transferred to a round bottom flask before the solvent was removed *in vacuo* at 40 °C. The crude mixture was then dissolved in 5 mL of MeOH/DCM (20:80), and the solution was passed through silica (sintered funnel with diameter = 5 cm, silica depth = 5 cm), eluting with 300 mL MeOH/DCM (20:80). Subsequently, the solvent was removed *in vacuo* to give the crude product, which was purified by flash column chromatography.

2.2. Reaction Optimization

2.2.1. Isolated α-Hydroxy Sulfinate

Procedure: A 7 mL vial was charged with photocatalyst (2.0 µmol, 2.0 mol%), α-hydroxy sulfinate **3a** (19 mg, 0.10 mmol, 1.0 equiv), H_2O (1.0 mL minus the volume of co-solvent and NaOH solution), and NaOH (4 M in H_2O). Subsequently, the solution was degassed by sparging with N_2 for 5 min before addition of 4-vinylpyridine (**5**, 11 µL, 0.10 mmol, 1.0 equiv). The mixture was then irradiated with blue LEDs at room temperature for 16 h. The solvent was then removed *in vacuo* with the aid of MeOH. The yields of **6a** and **6b** were subsequently determined by ^1H NMR analysis using an internal standard. Low yields of benzyl alcohol (**3b**) were observed due to α-hydroxy sulfinate reduction.

<u>Photocatalyst screening</u>

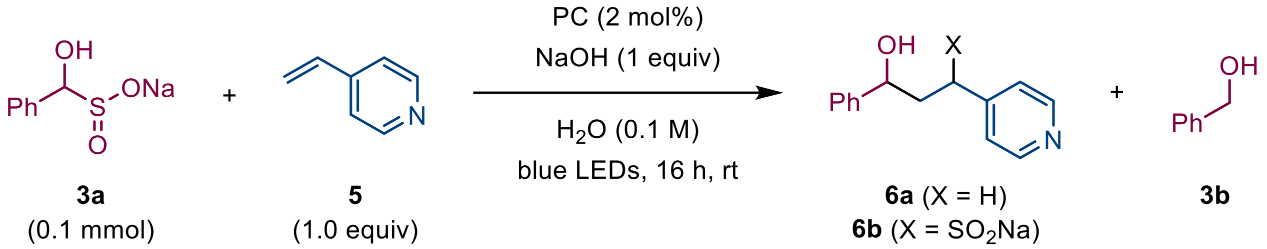

3a (0.1 mmol) **5** (1.0 equiv) **6a** (X = H) **6b** (X = SO_2Na) **3b**

Entry	Photocatalyst (PC)	Yield of **6a** (%)[a]	Yield of **6b** (%)[a]	Overall yield: **6a** + **6b** (%)[a]	Yield of **3b** (%)[a]
1	$Ru(bpy)_3Cl_2 \cdot 6H_2O$	18	12	30	4
2	Eosin Y	**41**	**4**	**45**	**3**
3	Rose bengal	13	2	15	2
4	Rhodamine G	2	0	2	1
5	Methylene blue	0	0	0	0
6	Fluorescein	4	6	10	1
7	Riboflavin	0	0	0	0

Table S1: Photocatalyst screening

[a] Yields were determined by ^1H NMR analysis using 1,4-dioxane as an internal standard.

Base equivalents screening

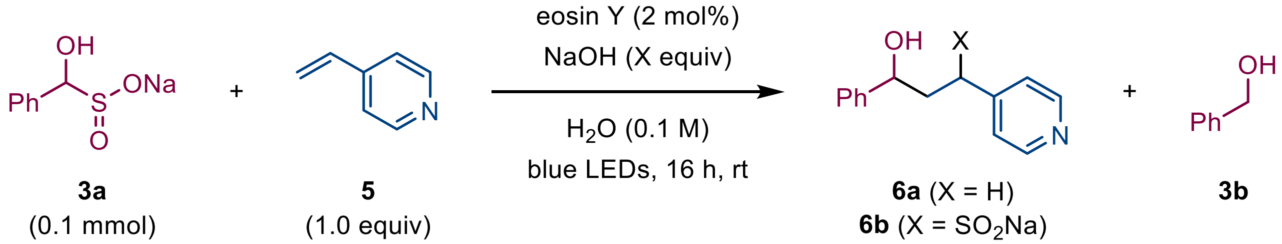

3a (0.1 mmol) | **5** (1.0 equiv) | **6a** (X = H), **6b** (X = SO_2Na) | **3b**

Entry	NaOH equivalents	Yield of **6a** (%)^a	Yield of **6b** (%)^a	Overall yield: **6a** + **6b** (%)^a	Yield of **3b** (%)^a
1	0	18	0	18	2
2	1	41	4	45	2
3	2	39	8	47	3
4	4	**46**	**2**	**48**	**1**

Table S2. Base equivalents screening

^{a} Yields were determined by ^{1}H NMR analysis using 1,4-dioxane as an internal standard.

Co-solvent screening

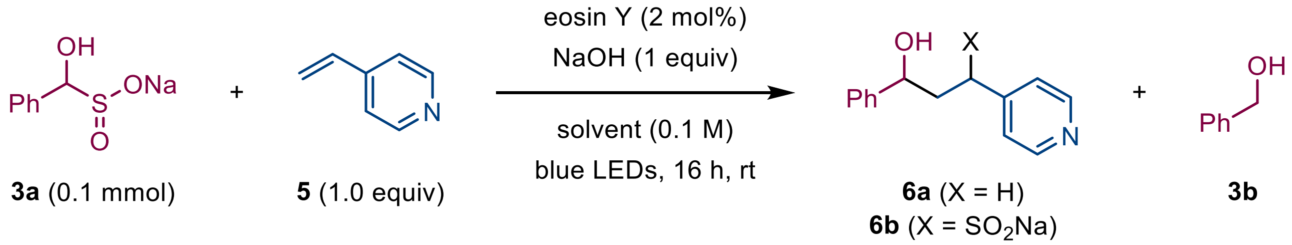

3a (0.1 mmol) | **5** (1.0 equiv) | **6a** (X = H), **6b** (X = SO_2Na) | **3b**

Entry	Solvent	Yield of **6a** (%)^a	Yield of **6b** (%)^a	Overall yield: **6a** + **6b** (%)^a	Yield of **3b** (%)^a
1	H_2O	41	4	45	2
2	H_2O/DMF (4:1)	21	0	21	0
3	H_2O/1,4-dioxane (4:1)	30^b	16^b	46^b	1
4	H_2O/MeCN (4:1)	**40**	**8**	**48**	**1**

Table S3. Co-solvent screening

^{a} Yields were determined by ^{1}H NMR analysis using 1,4-dioxane as an internal standard. ^{b} Yields were determined by ^{1}H NMR analysis using CH_2Br_2 as an internal standard.

2.2.2. *In Situ* Generated α-Hydroxy Sulfinate

Formation of α-hydroxy sulfinate **3a**

Procedure: A 7 mL vial was charged with thiourea dioxide (TDO, 16.2 mg, 0.15 mmol, 1.5 equiv). D_2O (0.80 mL) and NaOH (4 M in D_2O, 0.20 mL, 0.80 mmol, 8.0 equiv) were then added to the vial. Subsequently, the solution was degassed by sparging with N_2 for 5 min before addition of benzaldehyde (**4**, 10 µL, 10.6 mg, 0.10 mmol, 1.0 equiv). The solution was stirred for 1 h before addition of 1,4-dioxane (8.5 µL, 0.10 mmol, 1.0 equiv) as the internal standard, and the yield of **3a-*D*** was determined by ^{1}H NMR analysis. Complete conversion was observed (Scheme S1a).

Complete conversion was also observed when the same reaction was performed in D_2O/MeCN-d_3 (4:1, 0.1 M) and with 3 equivalents of NaOH (Scheme S1b)

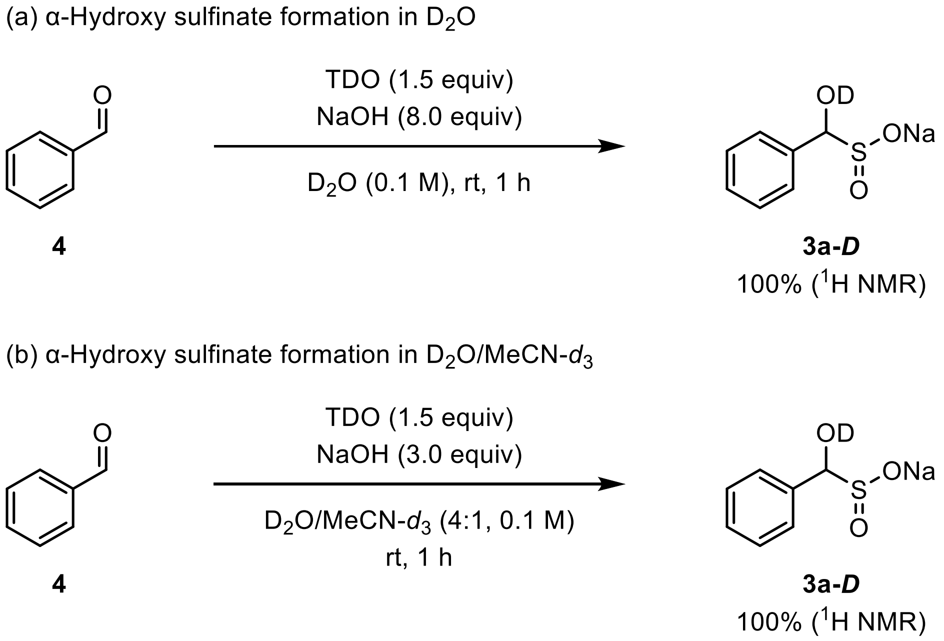

Scheme S1. *In situ* generation of α-hydroxy sulfinate 3a

One-pot α-hydroxy sulfinate formation/ketyl–olefin coupling reaction

Since α-hydroxy sulfinate **3a** could be generated *in situ* with 100% yield, the reaction was repeated with H_2O as the solvent in the presence of eosin Y (1.3 mg, 2.0 µmol, 2 mol%). After 1 h stirring, 4-vinylpyridine (16 µL, 0.15 mmol, 1.5 equiv) was added and the mixture was irradiated with blue LEDs at room temperature for 16 h. The solvent was then removed *in vacuo* with the aid of MeOH. The yield was subsequently determined by ^{1}H NMR analysis to be 54% overall NMR yield was obtained.

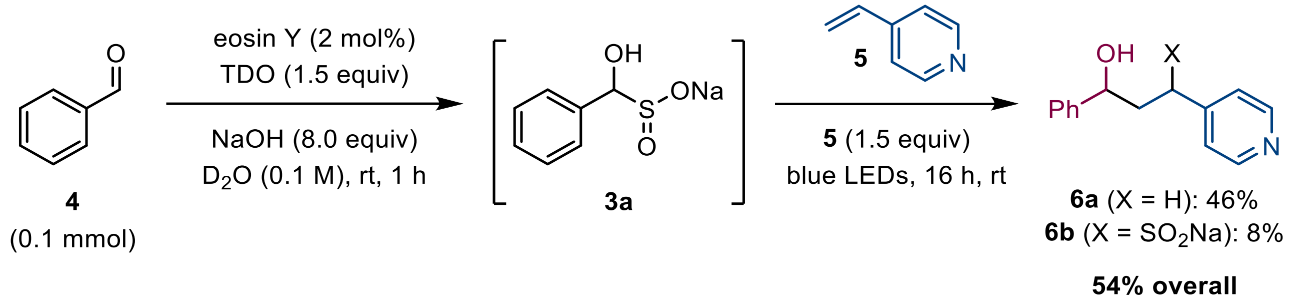

Scheme S2. One-pot transformation of *in situ* generated α-hydroxy sulfinate 3a

Optimization of the *in situ* activation procedure

Procedure: A 7 mL vial was charged with eosin Y (1.3 mg, 2.0 µmol, 2.0 mol%) and thiourea dioxide (TDO, 32 mg, 0.30 mmol, 3.0 equiv). MeCN (0.40 mL), H_2O (1.4 mL) and NaOH (4 M in H_2O, 0.20 mL, 0.8 mmol, 8.0 equiv) were then added to the vial. Subsequently, the solution was degassed by sparging with N_2 for 5 min before addition of benzaldehyde (**4**, 10 µL, 10.6 mg, 0.10 mmol, 1.0 equiv) and 4-vinylpyridine (**5**, 32 µL, 0.30 mmol, 3.0 equiv). The mixture was then stirred in the dark for 1 h before irradiation with blue LEDs at room temperature for 16 h. The solvent was then removed *in vacuo* and the yields of **6a** and **6b** were determined by ^{1}H NMR analysis using 1,4-dioxane as an internal standard. Benzyl alcohol (**3b**) was also observed due to α-hydroxy sulfinate reduction.

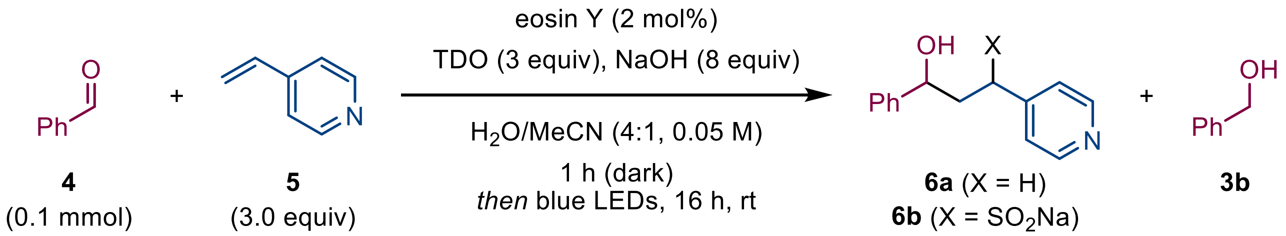

Entry	Variation from the above conditions	Yield of **6a** (%)[a]	Yield of **6b** (%)[a]	Overall yield: **6a** + **6b** (%)[a]	Yield of **3b** (%)[a]
1	none	60	14	74	6
2	without eosin Y	0	0	0	16
3	without light	0	0	0	15
4	without TDO	0	0	0	0
5	$Ru(bpy)_3Cl_2$ instead of eosin Y	33	28	61	2
6	H_2O (0.05 M) as the solvent	50	20	70	4
7	4:1 H_2O/THF (0.05 M) as the solvent	62	11	73	3
8	4.0 equiv NaOH	56	0	56	2
9	without pre-stirring	62	11	73	4

Table S4: Reaction optimization with *in situ* generated α-hydroxy sulfinate

[a] Yields were determined by ^{1}H NMR analysis using 1,4-dioxane as an internal standard.

2.3. Synthesis of Starting Materials

2.3.1. Synthesis of α-Hydroxy Sulfinates

Hydroxy(phenyl)methanesulfinate (3a)

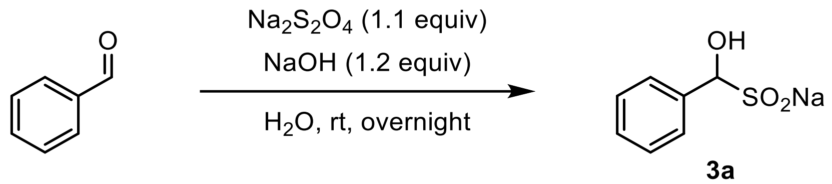

Prepared following a method reported by Mulliez and Naudy.[2]

Sodium dithionite (9.58 g, 55.0 mmol, 1.10 equiv) was added to a solution of NaOH (2.4 g, 60 mmol, 1.2 equiv) in H_2O (50 mL, 1.1 M), and the solution was stirred and degassed by sparging with N_2 for 15 min. Subsequently, benzaldehyde (5.1 mL, 50 mmol, 1.0 equiv) was added dropwise, and the mixture was stirred under N_2 at room temperature overnight to give a white suspension. The solid was collected by filtration, washed with acetone, and then dried *in vacuo* to give sodium hydroxy(phenyl)methanesulfinate (**3a**) as a white solid (6.9 g, 71%).

NMR Spectroscopy (*see spectra*):

^{1}H NMR (400 MHz, D_2O): δ_H 7.50 – 7.40 (m, 5H), 4.58 (s, 1H) ppm.

^{13}C NMR (101 MHz, D_2O): δ_C 135.43, 128.53, 128.44, 127.27, 94.26 ppm.

All recorded spectroscopic data matched those previously reported in the literature.[2]

2.3.2. Synthesis of Aldehydes

***N*-(2-Formylphenyl) acetamide**

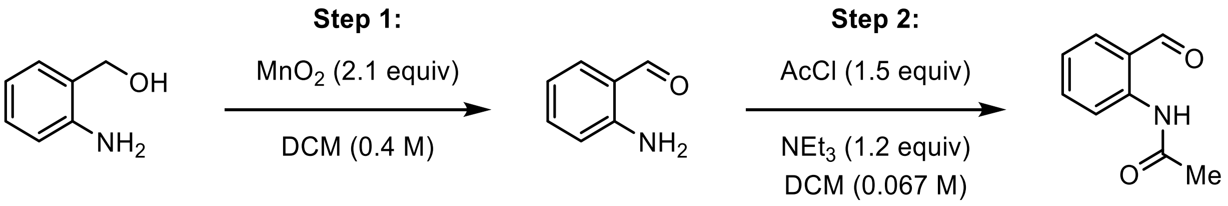

Step 1: Prepared following a method reported by Willis.[3]

2-Aminobenzyl alcohol (1.23 g, 10.0 mmol) was dissolved in DCM (25 mL). After addition of MnO_2 (1.82 g, 21.0 mmol), the solution was stirred under N_2 at room temperature overnight. MnO_2 was then removed by filtration and the resulting filtrate was concentrated *in vacuo*. Purification by flash column chromatography (20:80 EtOAc/petroleum ether) gave 2-aminobenzaldehyde as a yellow oil (970 mg, 80%).

R_f = 0.56 (20:80 EtOAc/petroleum ether, $KMnO_4$).

Step 2: 2-Aminobenzaldehyde (570 mg, 4.71 mmol, 1.00 equiv) was dissolved in DCM (15 mL). After addition of triethylamine (0.79 mL, 5.6 mmol, 1.2 equiv) and acetyl chloride (0.50 mL, 7.1 mmol, 1.5 equiv), the solution

was stirred at room temperature for 1 h. The reaction mixture was then washed with 1 M aqueous HCl and saturated aqueous $NaHCO_3$, dried with $MgSO_4$, filtered, and concentrated *in vacuo*. Purification by flash column chromatography (20:80 EtOAc/petroleum ether) gave *N*-(2-formylphenyl) acetamide white solid (407 mg, 53%).

R_f = 0.45 (20:80 EtOAc/petroleum ether, $KMnO_4$).

NMR Spectroscopy (*see spectra*):

^{1}H NMR (400 MHz, $CDCl_3$): δ_H 11.12 (s, 1H), 9.91 (s, 1H), 8.72 (d, *J* = 8.5 Hz, 1H), 7.66 (dd, *J* = 7.6, 1.7 Hz, 1H), 7.60 (ddd, *J* = 8.7, 7.3, 1.7 Hz, 1H), 7.21 (td, *J* = 7.5, 1.1 Hz, 1H), 2.25 (s, 3H) ppm;

^{13}C NMR (101 MHz, $CDCl_3$): δ_C 195.71, 169.76, 141.12, 136.36, 136.18, 122.99, 121.63, 119.96, 25.54 ppm.

All recorded spectroscopic data matched those previously reported in the literature.[4]

2.3.3. Synthesis of Olefins

2-Fluoro-4-vinylpyridine

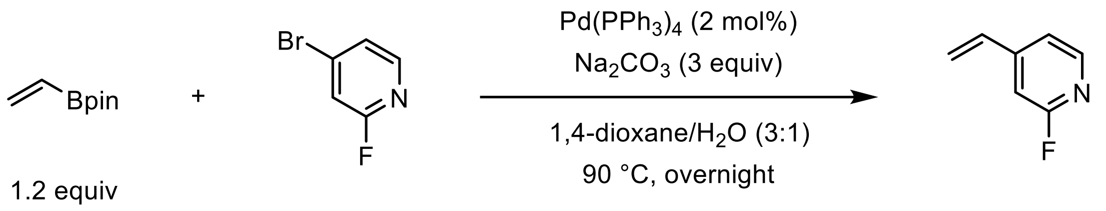

To a round bottom flask was added tetrakis(triphenylphosphine)palladium (185 mg, 0.160 mmol, 2.00 mol%) and Na_2CO_3 (2.54 g, 24.0 mmol, 3.00 equiv). 1,4-Dioxane (22 mL) and H_2O (7 mL) were added to the flask before vinyl-Bpin (1.63 mL, 9.60 mmol, 1.20 equiv) and 4-bromo-2-fluoro-pyridine (822 µL, 8.00 mmol, 1.00 equiv) were added. The reaction mixture was heated to 90 °C under N_2 overnight before it was cooled to room temperature and diluted with H_2O. The mixture was extracted three times with Et_2O and the combined organic phases were dried over $MgSO_4$, filtered, and concentrated *in vacuo*. Purification by flash column chromatography (10:90 Et_2O/petroleum ether) gave 2-fluoro-4-vinylpyridine as a yellow oil (454 mg, 46%).

R_f = 0.36 (10:90 Et_2O/petroleum ether, $KMnO_4$).

NMR Spectroscopy (*see spectra*):

^{1}H NMR (400 MHz, $CDCl_3$): δ_H 8.15 (d, *J* = 5.2 Hz, 1H), 7.16 (dt, *J* = 5.2, 1.7 Hz, 1H), 6.88 (s, 1H), 6.66 (dd, *J* = 17.6, 10.8 Hz, 1H), 5.98 (d, *J* = 17.6 Hz, 1H), 5.55 (d, *J* = 10.9 Hz, 1H) ppm.

^{13}C NMR (101 MHz, $CDCl_3$): δ_C 164.63 (d, *J* = 237.6 Hz), 150.57 (d, *J* = 8.2 Hz), 147.95 (d, *J* = 15.5 Hz), 133.91 (d, *J* = 3.7 Hz), 120.20, 118.67 (d, *J* = 3.9 Hz), 106.46 (d, *J* = 37.9 Hz) ppm.

^{19}F NMR (377 MHz, $CDCl_3$): δ_F −68.58 (s, 1F) ppm.

All recorded spectroscopic data matched those previously reported in the literature.[5]

2-Chloro-4-vinylpyridine

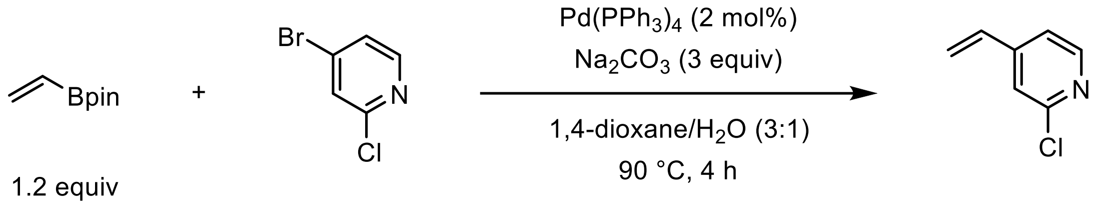

To a round bottom flask was added tetrakis(triphenylphosphine)palladium (92 mg, 0.080 mmol, 2.0 mol%) and Na_2CO_3 (1.27 g, 12.0 mmol, 3.00 equiv). 1,4-Dioxane (11 mL) and H_2O (3.5 mL) were added to the flask before vinyl-Bpin (815 µL, 4.80 mmol, 1.20 equiv) and 4-bromo-2-chloro-pyridine (450 µL, 4.00 mmol, 1.00 equiv) were added. The reaction mixture was heated to 90 °C under N_2 for 4 h before it was cooled to room temperature and diluted with H_2O. The mixture was extracted three times with Et_2O and the combined organic phases were dried over $MgSO_4$, filtered, and concentrated *in vacuo*. Purification by flash column chromatography (10:90 Et_2O/petroleum ether) gave 2-chloro-4-vinylpyridine as a yellow oil (494 mg, 89%).

$\mathbf{R_f}$ = 0.29 (10:90 Et_2O/petroleum ether).

NMR Spectroscopy (*see spectra*):

^{1}**H NMR** (400 MHz, $CDCl_3$): δ_H 8.31 (dd, *J* = 5.2, 0.7 Hz, 1H), 7.29 – 7.28 (m, 1H), 7.18 (dd, *J* = 5.2, 1.5 Hz, 1H), 6.61 (dd, *J* = 17.5, 10.8 Hz, 1H), 5.97 (d, *J* = 17.5 Hz, 1H), 5.54 (d, *J* = 10.8 Hz, 1H) ppm.

^{13}**C NMR** (101 MHz, $CDCl_3$): δ_C 152.28, 150.02, 148.04, 133.72, 121.42, 120.33, 119.62 ppm.

All recorded spectroscopic data matched those previously reported in the literature.[6]

2-(Trifluoromethyl)-4-vinylpyridine

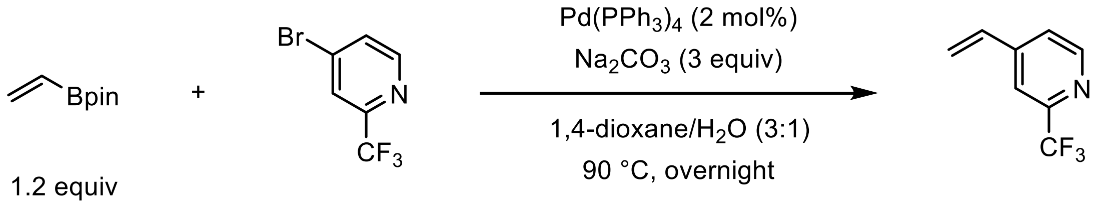

To a round bottom flask was added tetrakis(triphenylphosphine)palladium (170 mg, 0.147 mmol, 2.00 mol%) and Na_2CO_3 (2.32 g, 21.9 mmol, 3.00 equiv). 1,4-Dioxane (20 mL) and H_2O (6.5 mL) were added to the flask before vinyl-Bpin (1.50 mL, 8.76 mmol, 1.20 equiv) and 4-bromo-2-(trifluoromethyl)pyridine (1.00 mL, 7.30 mmol, 1.00 equiv) were added. The reaction mixture was heated to 90 °C under N_2 overnight before it was cooled to room temperature and diluted with H_2O. The mixture was extracted three times with Et_2O and the combined organic phases were dried over $MgSO_4$, filtered, and concentrated *in vacuo*. Purification by flash column chromatography (10:90 Et_2O/petroleum ether) gave 2-(trifluoromethyl)-4-vinylpyridine as a yellow oil (688 mg, 54%)

$\mathbf{R_f}$ = 0.24 (10:90 Et_2O/petroleum ether).

NMR Spectroscopy (*see spectra*):

^{1}H NMR (400 MHz, $CDCl_3$): δ_H 8.66 (d, J = 5.1 Hz, 1H), 7.65 (s, 1H), 7.44 (d, J = 4.6 Hz, 1H), 6.72 (dd, J = 17.6, 10.9 Hz, 1H), 6.06 (d, J = 17.6 Hz, 1H), 5.61 (d, J = 10.9 Hz, 1H) ppm.

^{13}C NMR (101 MHz, $CDCl_3$): δ_C 150.54, 148.90 (q, J = 34.4 Hz), 146.67, 133.83, 123.46, 121.75 (q, J = 270.10 Hz), 120.73, 117.74 – 117.40 (m) ppm.

^{19}F NMR (377 MHz, $CDCl_3$): δ_F −68.06 (s, 3F) ppm.

All recorded spectroscopic data matched those previously reported in the literature.[7]

4-(Prop-1-en-2-yl)pyridine

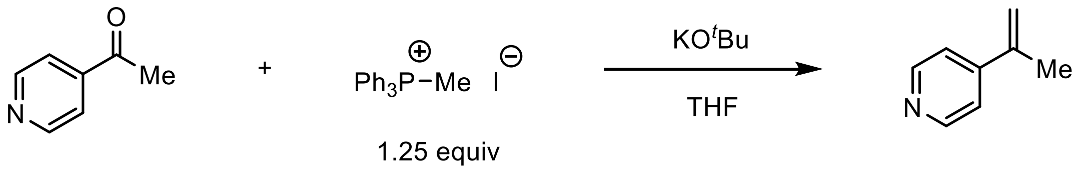

Prepared following a method reported by Vaquero.[8]

Anhydrous THF (20 mL) was added to methyl-triphenyl-phosphonium iodide (4.0 g, 9.9 mmol, 1.5 equiv) under N_2. The suspension was cooled down to 0 °C before KO^{t}Bu (1.0 M in THF, 9.9 mL, 9.9 mmol, 1.5 equiv) was added slowly. The mixture was then stirred for 1 h at room temperature. Subsequently, 4-acetylpyridine (0.74 mL, 6.6 mmol, 1.0 equiv) was added and the mixture was stirred overnight at room temperature. H_2O (40 mL) was added, and the mixture was extracted with diethyl ether (3 × 20 mL). The combined organic phases were dried over $MgSO_4$, filtered, and evaporated *in vacuo*. Purification by flash column chromatography (5:95 MeOH/DCM) gave 4-(prop-1-en-2-yl)pyridine as a yellow oil (537 mg, 68%).

R_f = 0.28 (5:95 MeOH/DCM, $KMnO_4$).

NMR Spectroscopy (*see spectra*):

^{1}H NMR (400 MHz, $CDCl_3$): δ_H (d, J = 5.3 Hz, 2H), 7.32 (d, J = 5.2 Hz, 2H), 5.56 (s, 1H), 5.25 (s, 1H), 2.14 (s, 3H) ppm.

^{13}C NMR (101 MHz, $CDCl_3$): δ_C 150.09, 148.41, 141.20, 120.23, 115.95, 21.01 ppm.

All recorded spectroscopic data matched those previously reported in the literature.[9]

4-(1-Phenylvinyl)pyridine

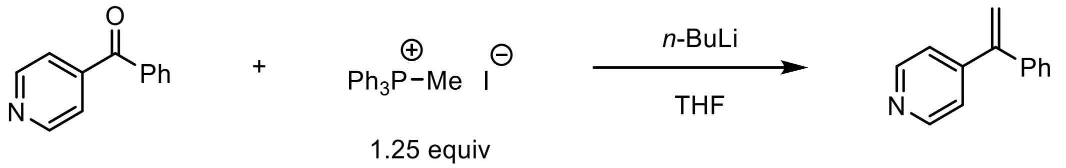

Prepared following a method reported by Hyster.[10]

A flame dried round bottom flask under an N_2 atmosphere was charged with methyl triphenyl phosphonium iodide (1.23 g, 3.05 mmol, 1.25 equiv) and THF (10 mL). The resulting suspension was cooled to 0 °C before *n*-BuLi (1.6 M solution in hexane, 1.9 mL, 3.05 mmol, 1.25 equiv) was added dropwise. The orange solution was stirred for 10 min and cooled to −78 °C before a solution of 4-benzoylpyridine (447 mg, 2.44 mmol, 1.00 equiv) in THF (10 mL) was added. The mixture was then warmed to room temperature and stirred overnight. The reaction was quenched with saturated aqueous NH_4Cl, the phases were separated, and the organic phase was extracted three times with Et_2O. The combined organic phases were dried over $MgSO_4$, filtered, and concentrated *in vacuo*. Purification by flash column chromatography (20:80 EtOAc/petroleum ether) gave 4-(1-phenylvinyl)pyridine as a yellow oil (227 mg, 51%).

R_f = 0.27 (20:80 EtOAc/petroleum ether, $KMnO_4$).

NMR Spectroscopy (*see spectra*):

^{1}H NMR (400 MHz, $CDCl_3$): δ_H 8.62 – 8.53 (m, 1H), 7.39 – 7.33 (m, 1H), 7.33 – 7.27 (m, 1H), 7.25 – 7.22 (m, 1H), 5.63 – 5.57 (m, 1H) ppm.

^{13}C NMR (101 MHz, $CDCl_3$): δ_C 150.04, 148.93, 148.10, 139.92, 128.58, 128.37, 128.28, 122.91, 117.05 ppm.

All recorded spectroscopic data matched those previously reported in the literature.[11]

4-(Cyclopent-1-en-1-yl)-2-(trifluoromethyl)pyridine (S1)

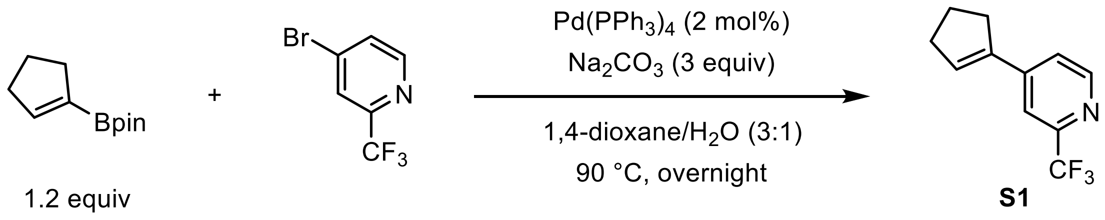

To a round bottom flask was added tetrakis(triphenylphosphine)palladium (116 mg, 0.100 mmol, 2.00 mol%), Na_2CO_3 (1.59 g, 15.0 mmol, 3.00 equiv). 1,4-Dioxane (20 mL) and H_2O (7 mL) were added to the flask before 2-(cyclopent-2-enyl)-4,4,5,5-tetramethyl-1,3,2-dioxaborolane (1.3 mL, 6.0 mmol, 1.2 equiv) and 4-bromo-2-trifluoromethyl-pyridine (685 µL, 5.00 mmol, 1.00 equiv) were added. The reaction mixture was heated to 90 °C under N_2 overnight before it was cooled to room temperature and diluted with H_2O. The mixture was extracted three times with Et_2O and the combined organic phases were dried over $MgSO_4$, filtered, and concentrated *in*

vacuo. Purification by flash column chromatography (10:90 Et_2O/petroleum ether) gave **S1** as a yellow oil (905 mg, 85%).

R_f = 0.16 (10:90 Et_2O/petroleum ether).

NMR Spectroscopy (*see spectra*):

^{1}H NMR (400 MHz, $CDCl_3$): δ_H 8.62 (d, *J* = 5.1 Hz, 1H), 7.65 (d, *J* = 1.6 Hz, 1H), 7.42 (dd, *J* = 5.2, 1.6 Hz, 1H), 6.55 (td, *J* = 2.7, 1.3 Hz, 1H), 2.72 (ddq, *J* = 8.2, 6.7, 2.3 Hz, 2H), 2.60 (tq, *J* = 7.8, 2.6 Hz, 2H), 2.08 (p, *J* = 7.6 Hz, 2H) ppm.

^{13}C NMR (101 MHz, $CDCl_3$): δ_C 150.25, 148.56 (q, *J* = 33.7 Hz), 145.72, 139.91, 133.69, 122.65, 121.86 (q, *J* = 271.5 Hz), 117.01 (q, *J* = 2.5 Hz), 33.80, 32.63, 23.23 ppm.

^{19}F NMR (377 MHz, $CDCl_3$): δ_F −68.11 (s, 3F) ppm.

All recorded spectroscopic data matched those previously reported in the literature.[12]

2-Vinylpyrimidine

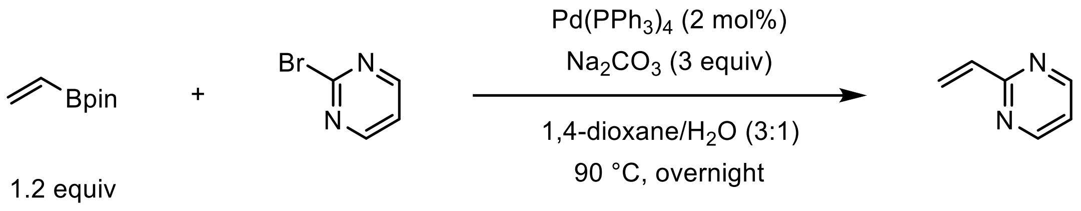

To a round bottom flask was added tetrakis(triphenylphosphine)palladium (185 mg, 0.160 mmol, 2.00 mol%), Na_2CO_3 (2.54 g, 24.0 mmol, 3.00 equiv) and 2-bromopyrimidine (1.27 g, 8.00 mmol, 1.00 equiv). 1,4-Dioxane (22 mL) and H_2O (7 mL) were added to the flask before vinyl-Bpin (1.63 mL, 9.60 mmol, 1.20 equiv) was added. The reaction mixture was heated to 90 °C under N_2 overnight before it was cooled to room temperature and diluted with H_2O. The mixture was extracted three times with Et_2O and the combined organic phases were dried over $MgSO_4$, filtered, and concentrated *in vacuo*. Purification by flash column chromatography (50:50 Et_2O/petroleum ether) gave 2-vinylpyrimidine as a yellow oil (492 mg, 58%).

R_f = 0.22 (50:50 Et_2O/petroleum ether).

NMR Spectroscopy (*see spectra*):

^{1}H NMR (400 MHz, $CDCl_3$): δ_H 8.68 (d, *J* = 4.9 Hz, 2H), 7.10 (t, *J* = 4.9 Hz, 1H), 6.86 (dd, *J* = 17.4, 10.5 Hz, 1H), 6.60 (dd, *J* = 17.4, 1.7 Hz, 1H), 5.71 (dd, *J* = 10.5, 1.7 Hz, 1H) ppm.

^{13}C NMR (101 MHz, $CDCl_3$): δ_C 164.51, 157.13, 136.63, 123.97, 119.25 ppm.

All recorded spectroscopic data matched those previously reported in the literature.[9]

1-(Methylsulfonyl)-4-vinylbenzene

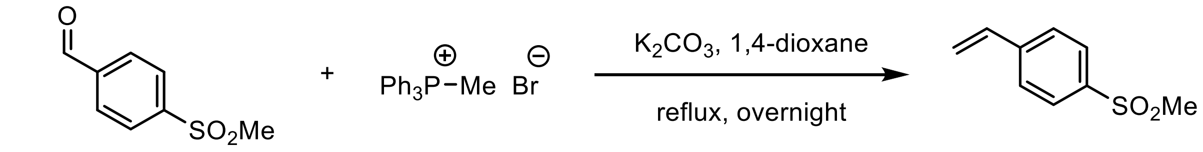

Prepared following a method reported by You.[13]

4-(Methylsulfonyl) benzaldehyde (921 mg, 5.00 mmol, 1.00 equiv), methyl triphenylphosphonium bromide (2.14 g, 6.00 mmol, 1.20 equiv), K_2CO_3 (1.1 g, 8.0 mmol, 1.6 equiv), and 1,4-dioxane (10 mL) were added to a round bottom flask. The mixture was then heated to reflux overnight before allowing to cool to room temperature. Subsequently, H_2O was added, the phases were separated, and the organic phase was extracted three times with DCM. The combined organic phases were dried over $MgSO_4$, filtered, and concentrated *in vacuo*. Purification by flash column chromatography (1:1 Et_2O/petroleum ether to 100% Et_2O) gave 1-(methylsulfonyl)-4-vinylbenzene as a yellow oil. The product was then crystallized from petroleum ether as a yellow solid (817 mg, 90%).

R_f = 0.20 (1:2 Et_2O/petroleum ether).

NMR Spectroscopy (*see spectra*):

^{1}H NMR (400 MHz, $CDCl_3$): δ_H 7.93 – 7.85 (m, 2H), 7.61 – 7.54 (m, 2H), 6.77 (dd, *J* = 17.6, 10.9 Hz, 1H), 5.91 (d, *J* = 17.6 Hz, 1H), 5.46 (d, *J* = 10.9 Hz, 1H), 3.05 (s, 3H) ppm.

^{13}C NMR (101 MHz, $CDCl_3$): δ_C 143.01, 139.45, 135.33, 127.88, 127.09, 118.16, 44.70 ppm.

All recorded spectroscopic data matched those previously reported in the literature.[14]

***N*,*N*-Dimethyl-4-vinylbenzenesulfonamide**

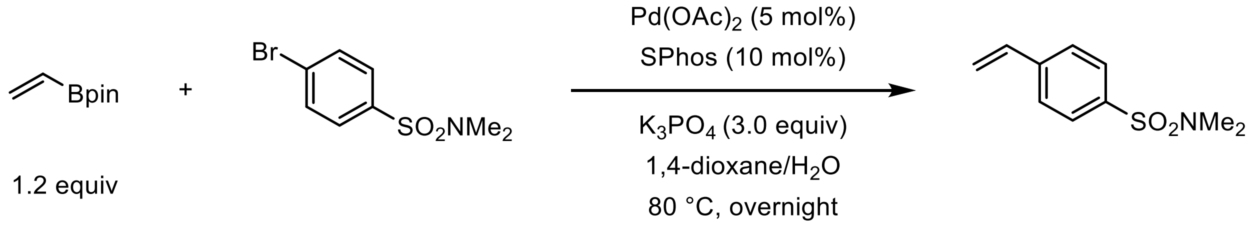

Prepared following a method reported by Gilmour.[15]

To a round bottom flask was added 1-bromo-4-(*N*,*N*-dimethylsulfonamide)benzene (792 mg, 3.00 mmol, 1.00 equiv), SPhos (123 mg, 0.300 mmol, 10.0 mol%), K_3PO_4 (1.91 g, 9.00 mmol, 3.00 equiv) and $Pd(OAc)_2$ (34 mg, 0.15 mmol, 5.0 mol%). 1,4-Dioxane (19 mL) and H_2O (4 mL) were added to the flask before vinyl-Bpin (0.61 mL, 3.6 mmol, 1.2 equiv) was added. The reaction mixture was heated to 80 °C under N_2 overnight before it was cooled to room temperature and extracted three times with EtOAc. The combined organic phases were dried over $MgSO_4$, filtered, and concentrated *in vacuo*. Purification by flash column chromatography (1:2 Et_2O/petroleum ether) gave *N*,*N*-dimethyl-4-vinylbenzenesulfonamide as a pale yellow solid (458 mg, 72%).

R$_f$ = 0.39 (1:2 Et_2O/petroleum ether).

NMR Spectroscopy (see spectra):

^{1}H NMR (400 MHz, $CDCl_3$): δ_H 7.73 (d, *J* = 8.2 Hz, 2H), 7.55 (d, *J* = 8.2 Hz, 2H), 6.76 (dd, *J* = 17.6, 10.9 Hz, 1H), 5.89 (d, *J* = 17.6 Hz, 1H), 5.43 (d, *J* = 10.9 Hz, 1H), 2.70 (s, 6H) ppm.

^{13}C NMR (101 MHz, $CDCl_3$): δ_C 141.96, 135.47, 134.39, 128.22, 126.77, 117.54, 38.07 ppm.

All recorded spectroscopic data matched those previously reported in the literature.[16]

N,2-Diphenylacrylamide

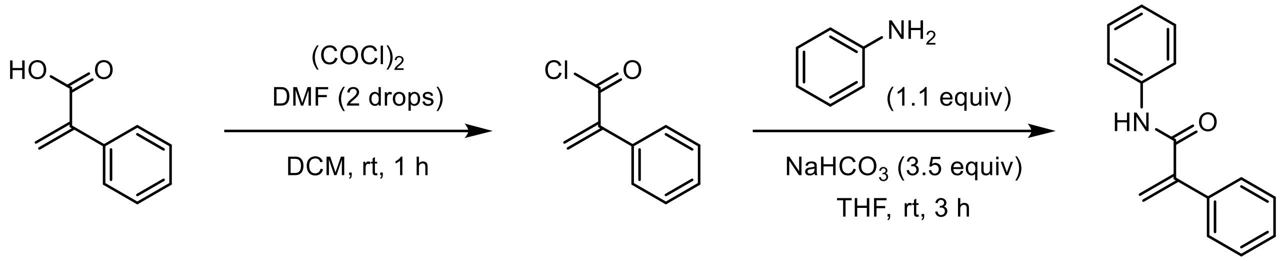

Prepared following a method reported by Li and Lu.[17]

To 2-phenylacrylic acid (1.0 g, 6.8 mmol, 1.0 equiv) was added DCM (10 mL) and oxalyl chloride (2 M in DCM, 3.7 mL, 7.4 mmol, 1.1 equiv), followed by 2 drops of DMF at room temperature. The reaction was stirred at room temperature for 1 h before concentration *in vacuo* gave the crude acyl chloride.

Subsequently, $NaHCO_3$ (2.00 g, 23.8 mmol, 3.50 equiv) and dry THF (40 mL) were added to the crude acyl chloride. Aniline (670 µL, 690 mg, 7.4 mmol, 1.1 equiv) was then added and the mixture was stirred at room temperature for 3 h. Then, H_2O (15 mL) and EtOAc (30 mL) were added and the phases were separated. The aqueous phase was extracted with EtOAc (20 mL) and the combined organic phases were washed with 1 M aqueous HCl (15 mL), dried over $MgSO_4$, filtered, and concentrated *in vacuo*. Purification by flash column chromatography (10:90 EtOAc/petroleum ether) gave *N*,2-diphenylacrylamide as a white solid (1.1 g, 73%).

R$_f$ = 0.27 (10:90 EtOAc/petroleum ether).

NMR Spectroscopy (see spectra):

^{1}H NMR (400 MHz, $CDCl_3$): δ_H 7.52 (d, *J* = 7.9 Hz, 2H), 7.49 – 7.40 (m, 5H), 7.39 (br. s, 1H), 7.32 (t, *J* = 7.8 Hz, 2H), 7.12 (t, *J* = 7.4 Hz, 1H), 6.30 (s, 1H), 5.73 (s, 1H) ppm.

^{13}C NMR (101 MHz, $CDCl_3$): δ_C 165.29, 145.23, 137.77, 136.81, 129.15, 129.11, 129.01, 128.43, 124.76, 123.53, 120.05 ppm.

All recorded spectroscopic data matched those previously reported in the literature.[18]

2.3.4. Synthesis of Starting Materials for Intramolecular Scope

N-Cinnamyl-*N*-(2-formylphenyl) acetamide (S2)

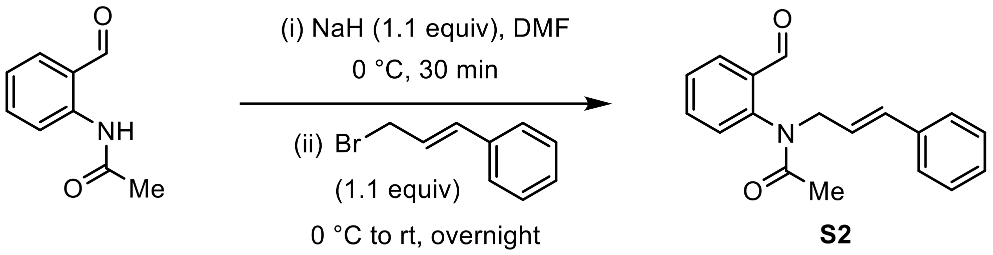

Prepared following a method reported by Lambert.[19]

N-(2-Formylphenyl) acetamide (163 mg, 1.0 mmol, 1.0 equiv) and NaH (60 wt. % dispersion in mineral oil, 44 mg, 1.1 mmol, 1.1 equiv) were added to a round bottom flask. The flask was cooled to 0 °C before the addition of DMF (2.5 mL) and the reaction mixture was stirred at 0 °C for 30 min. Subsequently, cinnamyl bromide (217 mg, 1.1 mmol, 1.1 equiv) was added before the solution was warmed to room temperature and stirred overnight. Then H_2O was added, and the mixture was extracted with Et_2O. The combined organic phases were washed with H_2O, dried over $MgSO_4$, filtered, and concentrated *in vacuo*. Purification by flash column chromatography (50:50 EtOAc/petroleum ether) gave **S2** as an orange oil (195 mg, 70%).

R_f = 0.49 (50:50 EtOAc/petroleum ether, $KMnO_4$).

NMR Spectroscopy (*see spectra*):

> **^{1}H NMR** (400 MHz, $CDCl_3$): δ_H 10.15 (s, 1H), 7.96 (dd, *J* = 7.7, 1.7 Hz, 1H), 7.66 (td, *J* = 7.6, 1.7 Hz, 1H), 7.56 – 7.49 (m, 1H), 7.32 – 7.18 (m, 6H), 6.36 – 6.17 (m, 2H), 4.64 (dd, *J* = 14.3, 6.3 Hz, 1H), 4.29 (dd, *J* = 14.3, 6.9 Hz, 1H), 1.82 (s, 3H) ppm.
>
> **^{13}C NMR** (101 MHz, $CDCl_3$): δ_C 189.71, 170.10, 144.55, 136.42, 135.61, 134.92, 133.07, 130.36, 130.34, 129.19, 128.73, 128.10, 126.62, 123.11, 52.46, 22.89 ppm.

IR (film): ν_{max} 3027, 2855, 2745, 1694, 1663, 1595, 1484, 1392, 1264, 1192 cm^{-1}.

HRMS (ESI$^+$): calc'd for $[M+Na]^+$, 302.1152; found, 302.1140.

(*E*)-1-(3-Bromoprop-1-en-1-yl)-4-(methylsulfonyl)benzene (S5)

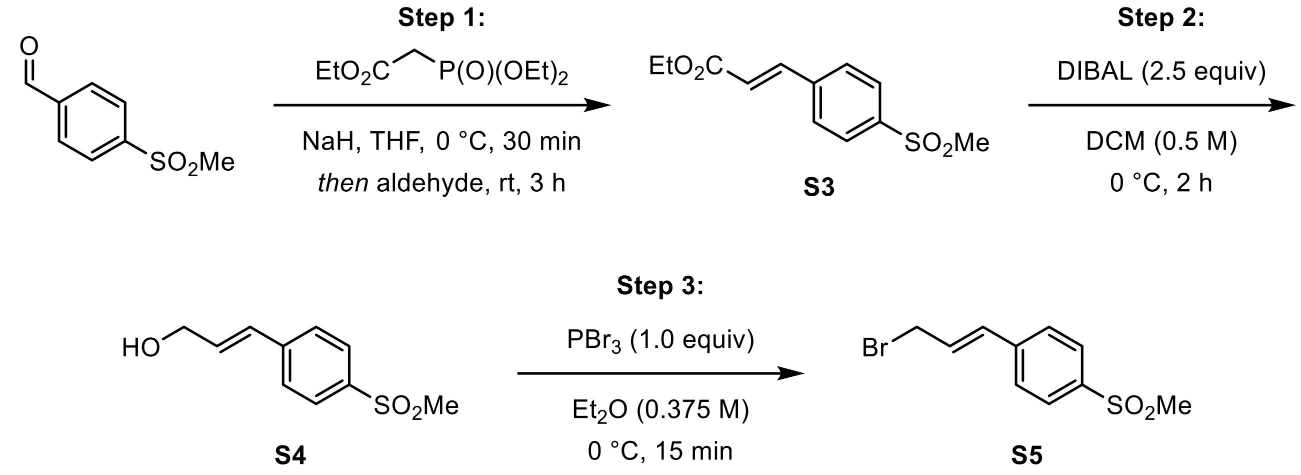

Prepared following a method reported by Shin.[20]

<u>**Step 1**</u>**:** To a flame-dried flask was added sodium hydride (60 wt.% dispersion in mineral oil, 1.25 g, 31.2 mmol, 1.30 equiv) and anhydrous THF (120 mL) and the mixture was cooled to 0 °C. Triethyl phosphonoacetate (6.19 mL, 6.99 g, 31.2 mmol, 1.3 equiv) was added and the mixture was stirred at 0 °C for 30 min before adding 4-methylsulfonylbenzaldehyde (4.4 g, 24 mmol, 1.0 equiv) at 0 °C. The mixture was then allowed to warm to room temperature and stirred for 3 h before being quenched with saturated aqueous NH_4Cl (30 mL) and H_2O (30 mL). The phases were separated, the aqueous phase was extracted with Et_2O (2 × 60 mL), and the combined organic phases were dried over $MgSO_4$, filtered, and concentrated *in vacuo*. Purification by flash column chromatography (30:70 EtOAc/petroleum ether) gave **S3** as a white solid (4.10 g, 67%).

$\mathbf{R_f}$ = 0.25 (30:70 EtOAc/petroleum ether, $KMnO_4$).

NMR Spectroscopy of S3 (*see spectra*):

> ^{1}**H NMR** (400 MHz, $CDCl_3$): δ_H 8.00 – 7.89 (m, 2H), 7.74 – 7.64 (m, 3H), 6.54 (d, *J* = 16.0 Hz, 1H), 4.28 (q, *J* = 7.1 Hz, 2H), 3.06 (s, 3H), 1.34 (t, *J* = 7.2 Hz, 3H) ppm.

<u>**Step 2**</u>**:** To a flame-dried flask was added **S3** (4.10 g, 16.1 mmol, 1.00 equiv) and DCM (32 mL) and the mixture was cooled to 0 °C. Diisobutylaluminum hydride (1.0 M in DCM, 40.3 mL, 40.3 mmol, 2.50 equiv) was added and the mixture was stirred at 0 °C for 2 h before being quenched with MeOH (12 mL) and a saturated aqueous solution of Rochelle's salt (80 mL). The phases were separated, the aqueous phase was extracted with DCM, and the combined organic phases were dried over $MgSO_4$, filtered, and concentrated *in vacuo*. Purification by flash column chromatography (1:19 MeOH/DCM) gave **S4** as a white solid (2.64 g, 77%).

$\mathbf{R_f}$ = 0.26 (1:19 MeOH/DCM, $KMnO_4$).

NMR Spectroscopy of S4 (*see spectra*):

> ^{1}**H NMR** (400 MHz, $CDCl_3$): δ_H 7.91 – 7.83 (m, 2H), 7.57 – 7.49 (m, 2H), 6.68 (dt, *J* = 16.0, 1.7 Hz, 1H), 6.51 (dt, *J* = 16.0, 5.1 Hz, 1H), 4.39 (dd, *J* = 5.1, 1.7 Hz, 2H), 3.05 (s, 3H) ppm.

<u>**Step 3**</u>**:** To a flame-dried flask was added **S4** (2.61 g, 12.3 mmol, 1.00 equiv) and DCM (30 mL) and the solution was cooled to 0 °C. Phosphorous tribromide (1.16 mL, 12.3 mmol, 1.00 equiv) was added and the mixture was

stirred at 0 °C for 30 min before being quenched with saturated aqueous $NaHCO_3$ (40 mL). The phases were separated, the aqueous phase was extracted with Et_2O (2 × 40 mL), and the combined organic phases were dried over $MgSO_4$, filtered, and concentrated *in vacuo*. Purification by flash column chromatography (100% DCM) gave **S5** as a white solid (2.70 g, 80%).

R_f = 0.33 (DCM, $KMnO_4$).

NMR Spectroscopy of S5 (*see spectra*):

^{1}**H NMR** (400 MHz, $CDCl_3$): δ_H 7.95 – 7.85 (m, 2H), 7.61 – 7.51 (m, 2H), 6.69 (d, *J* = 15.7 Hz, 1H), 6.54 (dt, *J* = 15.5, 7.5 Hz, 1H), 4.15 (dd, *J* = 7.6, 1.0 Hz, 2H), 3.05 (s, 3H) ppm.

^{13}**C NMR** (101 MHz, $CDCl_3$): δ_C 141.39, 139.91, 132.50, 129.46, 127.98, 127.56, 44.67, 32.12 ppm.

IR (film): v_{max} 3020, 2926, 1594, 1405, 1299, 1148, 1090 cm^{-1}.

HRMS (ESI$^+$): calc'd for $[M+H]^+$, 274.9736; found 274.9740.

(*E*)-*N*-(2-Formylphenyl)-*N*-(3-(4-(methylsulfonyl)phenyl)allyl)acetamide (S6)

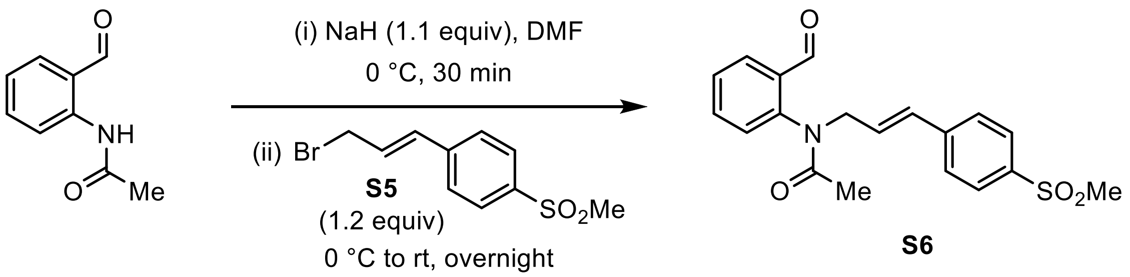

Prepared following a method reported by Lambert.[19]

N-(2-Formylphenyl) acetamide (170 mg, 1.04 mmol, 1.00 equiv) and sodium hydride (60 wt.% dispersion in mineral oil, 46 mg, 1.1 mmol, 1.1 equiv) were added to a round bottom flask. The flask was cooled to 0 °C before the addition of DMF (2.5 mL) and the reaction mixture was stirred at 0 °C for 30 min. Subsequently, (*E*)-1-(3-bromoprop-1-en-1-yl)-4-(methylsulfonyl)benzene (**S5**, 343 mg, 1.25 mmol, 1.20 equiv) was added before the solution was warmed to room temperature and stirred overnight. Then H_2O was added, and the mixture was extracted with EtOAc. The combined organic phases were washed with water, dried over $MgSO_4$, filtered, and concentrated *in vacuo*. Purification by flash column chromatography (1:100 to 1:20 MeOH/DCM) gave **S6** as a light-yellow solid (253 mg, 68%).

R_f = 0.46 (5:95 MeOH/DCM).

NMR Spectroscopy (*see spectra*):

^{1}**H NMR** (400 MHz, $CDCl_3$): δ_H 10.13 (s, 1H), 7.96 (dd, *J* = 7.7, 1.7 Hz, 1H), 7.89 – 7.79 (m, 2H), 7.69 (td, *J* = 7.6, 1.7 Hz, 1H), 7.59 – 7.51 (m, 1H), 7.48 – 7.40 (m, 2H), 7.27 – 7.24 (m, 1H), 6.44 – 6.38 (m, 2H), 4.63 (dd, *J* = 14.5, 5.3 Hz, 1H), 4.33 (dd, *J* = 14.5, 5.6 Hz, 1H), 3.02 (s, 3H), 1.82 (s, 3H) ppm.

^{13}C NMR (101 MHz, $CDCl_3$): δ_C 189.65, 170.21, 144.18, 141.80, 139.53, 135.76, 132.91, 132.70, 130.97, 130.25, 129.40, 127.94, 127.68, 127.29, 52.29, 44.67, 22.80 ppm.

IR (film): v_{max} 3009, 2926, 2853, 1694, 1660, 1595, 1393, 1304, 1148 cm^{-1}.

HRMS (ESI$^+$): calc'd for $[M+H]^+$, 358.1108; found, 358.1095.

2-Cinnamylbenzaldehyde (S7)

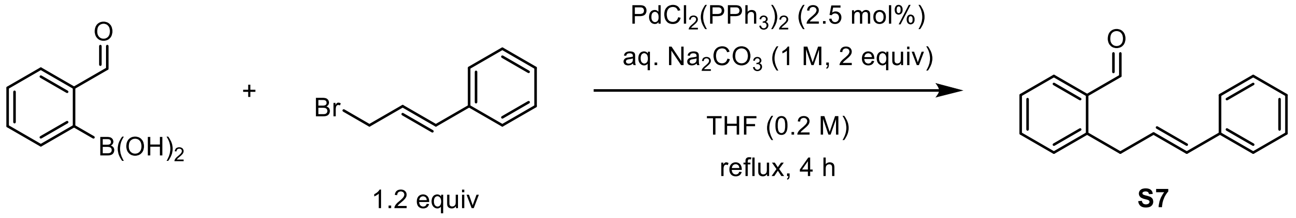

Prepared following a method reported by Youn.[21]

To a solution of the 2-formylphenylboronic acid (1.2 g, 8.0 mmol, 1.0 equiv), cinnamyl bromide (1.89 g, 9.60 mmol, 1.20 equiv) and $PdCl_2(PPh_3)_2$ (140 mg, 0.200 mmol, 2.50 mol%) in THF (40 mL, 0.2 M) was added an aqueous solution of Na_2CO_3 (1 M, 16 mL, 2.0 equiv). The reaction mixture was then heated to reflux overnight. After allowing to cool to room temperature, the reaction mixture was quenched with H_2O and extracted with DCM. The combined organic phases were dried over $MgSO_4$, filtered, and concentrated *in vacuo*. Purification by flash column chromatography (5:95 EtOAc/petroleum ether) gave **S7** as yellow oil (0.96 g, 54%).

R_f = 0.49 (5:95 EtOAc/petroleum ether).

NMR Spectroscopy (*see spectra*):

^{1}H NMR (400 MHz, $CDCl_3$): δ_H 10.30 (s, 1H), 7.87 (dd, J = 7.7, 1.5 Hz, 1H), 7.55 (td, J = 7.5, 1.5 Hz, 1H), 7.43 (td, J = 7.5, 1.3 Hz, 1H), 7.39 – 7.25 (m, 5H), 7.24 – 7.16 (m, 1H), 6.43 – 6.37 (m, 2H), 4.02 – 3.96 (m, 2H) ppm.

^{13}C NMR (101 MHz, $CDCl_3$): δ_C 192.63, 142.61, 137.32, 134.17, 133.96, 132.29, 131.70, 131.25, 128.73, 128.64, 127.41, 127.14, 126.28, 35.95 ppm.

All recorded spectroscopic data matched those previously reported in the literature.[21]

(*E*)-2-(3-(4-(Methylsulfonyl)phenyl)allyl)benzaldehyde (S8)

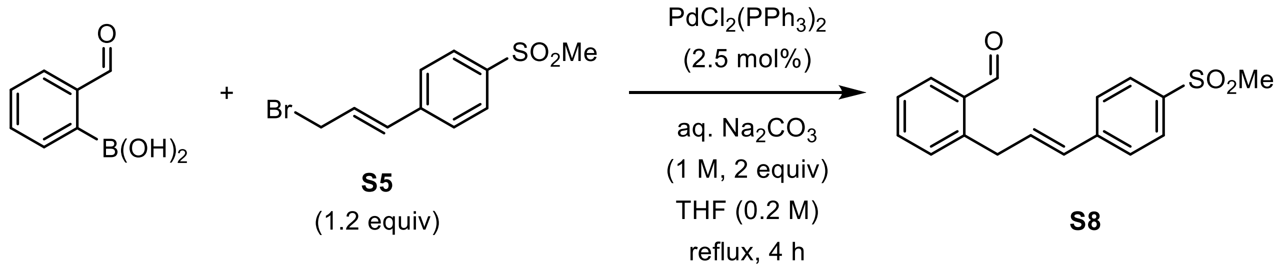

Prepared following a method reported by Youn.[21]

To a solution of the 2-formylphenylboronic acid (300 mg, 2.00 mmol, 1.00 equiv), (*E*)-1-(3-bromoprop-1-en-1-yl)-4-(methylsulfonyl)benzene (**S5**, 606 mg, 2.20 mmol, 1.20 equiv) and $PdCl_2(PPh_3)$ (35 mg, 0.050 mmol, 2.5 mol%) in THF (10 mL, 0.2 M) was added an aqueous solution of Na_2CO_3 (1.0 M, 4.0 mL, 2.0 equiv). The reaction mixture was then heated to reflux overnight. After allowing to cool to room temperature, the reaction mixture was quenched with H_2O and extracted with DCM. The combined organic phases were dried over $MgSO_4$, filtered, and concentrated *in vacuo*. Purification by flash column chromatography (30:70 EtOAc/petroleum ether) gave **S8** as a yellow oil (204 mg, 34%).

R_f = 0.18 (30:70 EtOAc/petroleum ether, $KMnO_4$).

NMR Spectroscopy (*see spectra*):

^{1}H NMR (400 MHz, $CDCl_3$): δ_H 10.23 (s, 1H), 7.89 – 7.80 (m, 3H), 7.57 (td, *J* = 7.5, 1.5 Hz, 1H), 7.52 – 7.42 (m, 3H), 7.35 (dd, *J* = 7.5, 1.2 Hz, 1H), 6.59 (dt, *J* = 15.9, 6.5 Hz, 1H), 6.42 (dt, *J* = 15.8, 1.6 Hz, 1H), 4.03 (dd, *J* = 6.6, 1.5 Hz, 2H), 3.02 (s, 3H) ppm.

^{13}C NMR (101 MHz, $CDCl_3$): δ_C 192.83, 142.90, 141.45, 138.77, 134.24, 133.96, 133.53, 133.36, 131.43, 129.85, 127.84, 127.46, 126.95, 44.72, 36.28 ppm.

IR (film): ν_{max} 3025, 2926, 2857, 2743, 1692, 1595, 1298, 1146, 1090 cm^{-1}.

HRMS (ESI$^+$): calc'd for $[M+Na]^+$, 323.0712; found, 323.0698.

2-(Cinnamylamino)benzaldehyde (S10)

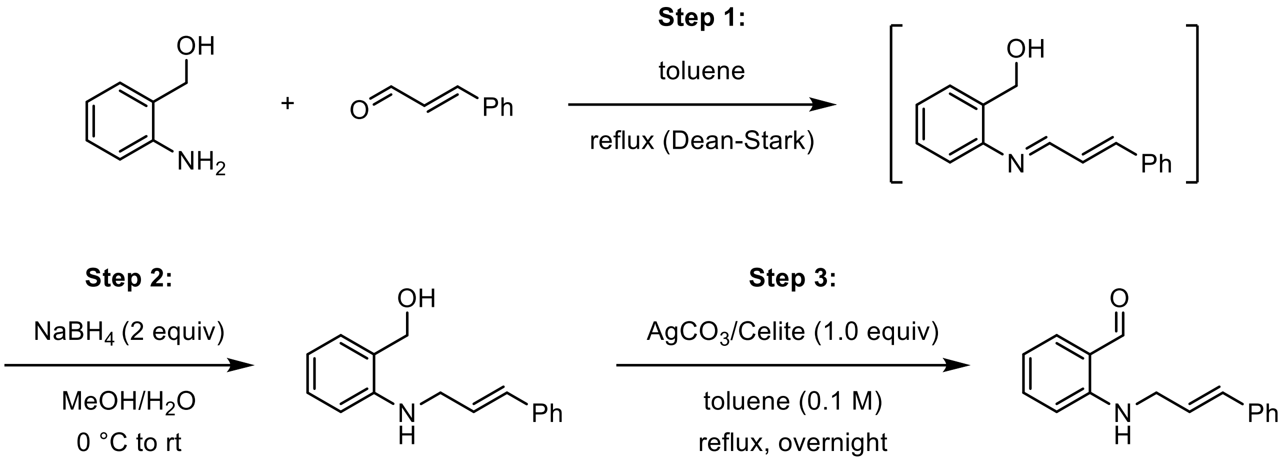

S9 **S10**

<u>Steps 1-2</u>: Prepared following a method reported by Nicewicz.[22]

To a flame dried round bottom flask was added 2-aminobenzyl alcohol (1.23 g, 10.0 mmol, 1.00 equiv) and cinnamaldehyde (1.25 mL, 10.0 mmol, 1.00 equiv) in anhydrous toluene (6.5 mL). A Dean-Stark apparatus was attached, and reaction was refluxed for 2 h. The H_2O generated was removed from the reaction with the aid of a heat gun. The reaction was then cooled to room temperature and solvent was removed *in vacuo*.

The resulting residue was resuspended in MeOH (13 mL) and cooled to 0 °C. Subsequently, a solution of sodium borohydride (750 mg, 20.0 mmol, 2.00 equiv) in H_2O (15 mL) was added over 30 min. The solution was then warmed to room temperature and stirred overnight. The reaction was quenched with saturated aqueous NH_4Cl, and the MeOH was removed *in vacuo*. The aqueous phase was extracted with DCM, and the combined organic phases were dried over $MgSO_4$, filtered, and concentrated *in vacuo*. Purification by flash column chromatography (20% EtOAc/petroleum ether) gave **S9** as a yellow oil (1.58 g, 66% over 2 steps).

<u>Step 3</u>: Prepared following a method reported by Hailes.[23]

To a solution of **S9** (1.2 g, 5.0 mmol, 1.0 equiv) in anhydrous toluene (40 mL) was added Fetizon's reagent (50 wt. % Ag_2CO_3/Celite, 3.3 g, 6.0 mmol, 1.2 equiv). The reaction mixture was refluxed under N_2 overnight. The insoluble materials were then removed by filtration and the filtrate was concentrated *in vacuo*. Purification by flash column chromatography (5:95 EtOAc/petroleum ether) gave **S10** as a yellow solid (0.98 g, 82%).

R_f = 0.53 (10:90 EtOAc/petroleum ether).

NMR Spectroscopy (*see spectra*):

> **^{1}H NMR** (400 MHz, $CDCl_3$): δ_H 9.86 (s, 1H), 8.54 (t, *J* = 6.0 Hz, 1H), 7.50 (dd, *J* = 7.7, 1.7 Hz, 1H), 7.43 – 7.35 (m, 3H), 7.35 – 7.28 (m, 2H), 7.27 – 7.21 (m, 1H), 6.78 – 6.68 (m, 2H), 6.61 (dt, *J* = 15.9, 1.8 Hz, 1H), 6.30 (dt, *J* = 15.9, 5.5 Hz, 1H), 4.08 (td, *J* = 6.0, 1.7 Hz, 2H) ppm.

> **^{13}C NMR** (101 MHz, $CDCl_3$): δ_C 194.23, 150.71, 136.83, 136.77, 135.98, 131.72, 128.70, 127.75, 126.51, 125.82, 118.72, 115.30, 111.35, 44.65 ppm.

All recorded spectroscopic data matched those previously reported in the literature.[23]

2.4. Substrate Scope

2.4.1. Aldehyde Scope

1-Phenyl-3-(pyridin-4-yl)propan-1-ol (6a)

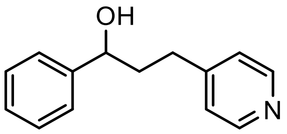

6a

Prepared following **General Procedure A** and the Work-Up Procedure, using benzaldehyde (25.5 µL). Purification by flash column chromatography (10:90 MeOH/DCM) gave **6a** (39.5 mg, 74%) as a white solid.

R$_f$ = 0.43 (10:90 MeOH/DCM, CAM).

NMR Spectroscopy (*see spectra*):

^{1}**H NMR** (400 MHz, $CDCl_3$): δ_H 8.46 – 8.39 (m, 2H), 7.38 – 7.27 (m, 5H), 7.13 – 7.07 (m, 2H), 4.68 (dd, *J* = 7.9, 5.2 Hz, 1H), 2.76 (ddd, *J* = 13.9, 9.9, 5.7 Hz, 1H), 2.67 (ddd, *J* = 13.9, 9.6, 6.5 Hz, 1H), 2.13 (dddd, *J* = 13.6, 9.6, 7.9, 5.7 Hz, 1H), 2.01 (dddd, *J* = 13.6, 9.9, 6.5, 5.2 Hz, 1H) ppm.

^{13}**C NMR** (101 MHz, $CDCl_3$): δ_C 151.20, 149.68, 144.49, 128.74, 127.94, 125.98, 124.09, 73.62, 39.40, 31.50 ppm.

HRMS (ESI$^+$): calc'd for $[M+H]^+$, 214.1226; found, 214.1221.

All recorded spectroscopic data matched those previously reported in the literature.[24]

1-([1,1'-Biphenyl]-4-yl)-3-(pyridin-4-yl)propan-1-ol (7)

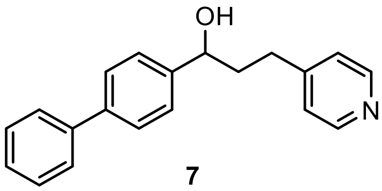

7

Prepared following **General Procedure A** and the Work-Up Procedure, using 4-phenylbenzaldehyde (45.6 mg). Purification by flash column chromatography (5:95 MeOH/DCM) gave **7** (35.2 mg, 49%) as a white solid.

R$_f$ = 0.33 (5:95 MeOH/DCM, CAM).

NMR Spectroscopy (*see spectra*):

^{1}**H NMR** (400 MHz, $CDCl_3$): δ_H 8.45 (d, *J* = 5.1 Hz, 2H), 7.62 – 7.56 (m, 4H), 7.49 – 7.39 (m, 4H), 7.39 – 7.31 (m, 1H), 7.13 (d, *J* = 5.8 Hz, 2H), 4.73 (dd, *J* = 7.9, 5.2 Hz, 1H), 2.79 (ddd, *J* = 15.3, 9.9, 5.7 Hz, 1H), 2.71 (ddd, *J* = 15.3, 9.6, 6.4 Hz, 1H), 2.47 (s, 1H), 2.25 – 2.11 (m, 1H), 2.06 (dddd, *J* = 13.8, 9.9, 6.4, 5.2 Hz, 1H) ppm.

^{13}C NMR (101 MHz, $CDCl_3$): δ_C 151.08, 149.78, 143.43, 140.90, 140.82, 128.95, 127.52, 127.48, 127.20, 126.45, 124.10, 73.40, 39.36, 31.51 ppm.

IR (film): v_{max} 3206, 3027, 2970, 2906, 1606, 1365, 1217, 1067, 1004 cm^{-1}.

HRMS (ESI$^+$): calc'd for [M+H]$^+$, 290.1539; found 290.1529.

1-(Naphthalen-2-yl)-3-(pyridin-4-yl)propan-1-ol (8)

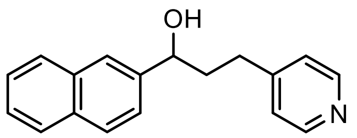

8

Prepared following **General Procedure A** and the Work-Up Procedure, using 2-naphthaldehyde (39.0 mg). Purification by flash column chromatography (5:95 MeOH/DCM) gave **8** (29.6 mg, 45%) as a colorless oil.

R$_f$ = 0.19 (5:95 MeOH/DCM, CAM).

NMR Spectroscopy (*see spectra*):

^{1}H NMR (500 MHz, $CDCl_3$): δ_H 8.45 – 8.40 (m, 2H), 7.87 – 7.80 (m, 3H), 7.77 (s, 1H), 7.53 – 7.40 (m, 3H), 7.13 – 7.08 (m, 2H), 4.85 (dd, *J* = 7.7, 5.3 Hz, 1H), 2.77 (ddd, *J* = 15.3, 9.9, 5.7 Hz, 1H), 2.70 (ddd, *J* = 15.3, 9.7, 6.5 Hz, 1H), 2.21 (dddd, *J* = 13.6, 9.7, 7.8, 5.7 Hz, 1H), 2.15 – 2.05 (m, 1H) ppm.

^{13}C NMR (126 MHz, $CDCl_3$): δ_C 151.17, 149.68, 141.79, 133.39, 133.19, 128.64, 128.03, 127.85, 126.43, 126.12, 124.79, 124.09, 124.00, 73.71, 39.26, 31.47 ppm.

IR (film): v_{max} 3201, 3053, 2922, 2856, 1934, 1602, 1417, 1067 cm^{-1}.

HRMS (ESI$^+$): calc'd for [M+H]$^+$,264.1383; found 264.1380.

Note: For 2-naphthaldehyde, the formation of the pinacol coupling product, 1,2-di(naphthalen-2-yl)ethane-1,2-diol,[25] was observed (48% yield, 2:1 d.r., determined by ^{1}H NMR analysis). We postulate that the extended π-system provides greater stabilization to the ketyl radical anion, which makes it less reactive towards 4-vinylpyridine and leads to competitive pinacol coupling.

1-(4-Methoxyphenyl)-3-(pyridin-4-yl)propan-1-ol (9)

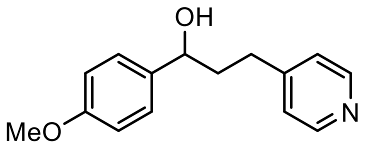

9

Prepared following **General Procedure A** and the Work-Up Procedure, using *p*-anisaldehyde (30.5 µL). Purification by flash column chromatography (5:95 MeOH/DCM) gave **9** (46.0 mg, 76%) as a white solid.

R$_f$ = 0.18 (5:95 MeOH/DCM, CAM).

NMR Spectroscopy (*see spectra*):

^{1}H NMR (400 MHz, $CDCl_3$): δ_H 8.43 (d, *J* = 5.1 Hz, 2H), 7.27 – 7.23 (m, 2H), 7.12 (d, *J* = 5.1 Hz, 2H), 6.91 – 6.80 (m, 2H), 4.61 (dd, *J* = 7.8, 5.4 Hz, 1H), 3.79 (s, 3H), 2.73 (ddd, *J* = 15.4, 9.9, 5.7 Hz, 1H), 2.71 (br. s, 1H), 2.64 (ddd, *J* = 15.4, 9.6, 6.4 Hz, 1H), 2.18 – 2.05 (m, 1H), 1.97 (dddd, *J* = 13.6, 9.9, 6.4, 5.4 Hz, 1H) ppm.

^{13}C NMR (101 MHz, $CDCl_3$): δ_C 159.36, 151.85, 149.19, 136.49, 127.25, 124.25, 114.10, 73.22, 55.44, 39.25, 31.63 ppm.

HRMS (ESI$^+$): calc'd for $[M+H]^+$, 244.1332; found, 244.1325.

All recorded spectroscopic data matched those previously reported in the literature.[26]

1-(2,4-Dimethoxyphenyl)-3-(pyridin-4-yl)propan-1-ol (10)

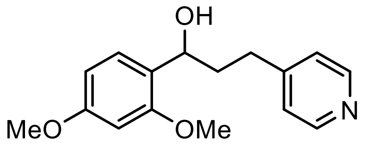

10

Prepared following **General Procedure A** and the Work-Up Procedure, using 2,4-dimethoxybenzaldehyde (41.5 mg). Purification by flash column chromatography (5:95 MeOH/DCM) gave **10** (46.4 mg, 68%) as a yellow oil.

R$_f$ = 0.20 (5:95 MeOH/DCM, CAM).

NMR Spectroscopy (*see spectra*):

^{1}H NMR (400 MHz, $CDCl_3$): δ_H 8.43 (d, *J* = 5.1 Hz, 2H), 7.19 (d, *J* = 8.2 Hz, 1H), 7.11 (d, *J* = 5.1 Hz, 2H), 6.49 – 6.41 (m, 2H), 4.81 (dd, *J* = 8.1, 5.1 Hz, 1H), 3.78 (s, 6H), 2.78 (ddd, *J* = 15.1, 9.9, 5.6 Hz, 1H), 2.75 (br. s, 1H), 2.66 (ddd, *J* = 15.1, 9.6, 6.6 Hz, 1H), 2.18 – 1.96 (m, 2H) ppm.

^{13}C NMR (101 MHz, $CDCl_3$): δ_C 160.33, 157.70, 151.57, 149.58, 127.59, 124.65, 124.14, 104.25, 98.81, 69.68, 55.51, 55.39, 37.58, 31.78 ppm.

HRMS (ESI⁺): calc'd for $[M+H]^+$, 274.1438; found, 274.1427.

All recorded spectroscopic data matched those previously reported in the literature.[27]

N-(4-(1-Hydroxy-3-(pyridin-4-yl)propyl)phenyl)acetamide (11)

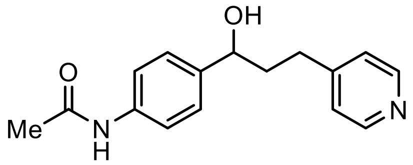

11

Prepared following **General Procedure A** and the Work-Up Procedure, using 4-acetamidobenzaldehyde (40.8 mg). Purification by flash column chromatography (5:95 to 10:90 MeOH/DCM) gave **11** (46.6 mg, 69%) as a yellow oil.

R_f = 0.30 (10:90 MeOH/DCM, CAM).

NMR Spectroscopy (*see spectra*):

> **^{1}H NMR** (400 MHz, $CDCl_3$): δ_H 8.44 – 8.37 (m, 2H), 7.61 (s, 1H), 7.49 – 7.41 (m, 2H), 7.30 – 7.22 (m, 2H), 7.11 – 7.04 (m, 2H), 4.61 (dd, *J* = 7.8, 5.3 Hz, 1H), 2.71 (ddd, *J* = 15.3, 9.8, 5.8 Hz, 1H), 2.66 (br.s, 1H), 2.63 (ddd, *J* = 15.3, 9.5, 6.5 Hz, 1H), 2.14 (s, 3H), 2.12 – 2.02 (m, 1H), 1.96 (dddd, *J* = 13.6, 9.7, 6.5, 5.3 Hz, 1H) ppm.
>
> **^{13}C NMR** (101 MHz, $CDCl_3$): δ_C 168.66, 151.18, 149.69, 140.35, 137.61, 126.67, 124.13, 120.22, 73.14, 39.30, 31.45, 24.65 ppm.

IR (film): ν_{max} 3260, 3190, 3121, 3056, 2926, 2853, 1667, 1604, 1540, 1520, 1416, 1316, 1265, 1003 cm^{-1}.

HRMS (ESI⁺): calc'd for $[M+H]^+$,271.1441; found 271.1439.

N-(2-(1-Hydroxy-3-(pyridin-4-yl)propyl)phenyl)acetamide (12)

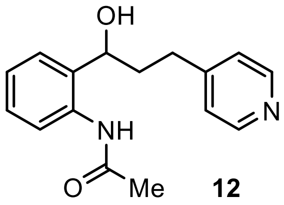

12

Prepared following **General Procedure A** and the Work-Up Procedure, using *N*-(2-formylphenyl) acetamide (40.8 mg). Purification by flash column chromatography (5:95 to 10:90 MeOH/DCM) gave **12** (28.2 mg, 42%) as a colorless oil.

R_f = 0.20 (5:95 MeOH/DCM, $KMnO_4$).

NMR Spectroscopy (*see spectra*):

^{1}**H NMR** (400 MHz, $CDCl_3$): δ_H 9.17 (s, 1H), 8.40 – 8.24 (m, 2H), 8.02 (d, *J* = 8.1 Hz, 1H), 7.28 – 7.21 (m, 1H), 7.11 – 7.05 (m, 2H), 7.05 – 6.99 (m, 2H), 4.66 (dd, *J* = 8.2, 5.9 Hz, 1H), 4.31 (s, 1H), 2.73 (ddd, *J* = 14.8, 9.3, 5.7 Hz, 1H), 2.62 (ddd, *J* = 14.8, 9.1, 6.8 Hz, 1H), 2.22 (dtd, *J* = 14.3, 8.7, 5.9 Hz, 1H), 2.09 (s, 3H), 2.08-1.98 (m, 1H) ppm.

^{13}**C NMR** (126 MHz, $CDCl_3$): δ_C 168.83, 151.32, 149.27, 136.75, 132.36, 128.65, 127.57, 124.38, 124.24, 123.18, 73.88, 36.56, 31.78, 24.84 ppm.

IR (film): v_{max} 3268, 3067, 3031, 2928, 1667, 1606, 1526, 1448, 1370, 1304, 1066 cm^{-1}.

HRMS (ESI$^+$): calc'd for $[M+H]^+$, 271.1441; found 271.1429.

1-(4-(Piperidin-1-yl)phenyl)-3-(pyridin-4-yl)propan-1-ol (13)

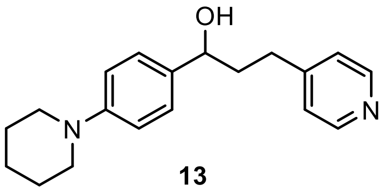

13

Prepared following **General Procedure A** and the Work-Up Procedure, using 4-(piperidin-1-yl) benzaldehyde (47.3 mg). Purification by flash column chromatography (5:95 MeOH/DCM) gave **13** (57.8 mg, 78%) as a yellow oil.

R$_f$ = 0.17 (5:95 MeOH/DCM, CAM).

NMR Spectroscopy (*see spectra*):

^{1}**H NMR** (400 MHz, $CDCl_3$): δ_H 8.50 – 8.40 (m, 2H), 7.24 – 7.16 (m, 2H), 7.16 – 7.06 (m, 2H), 6.94 – 6.86 (m, 2H), 4.56 (dd, *J* = 7.7, 5.6 Hz, 1H), 3.17 – 3.10 (m, 4H), 2.71 (ddd, *J* = 14.1, 9.9, 5.8 Hz, 1H), 2.62 (ddd, *J* = 14.1, 9.7, 6.4 Hz, 1H), 2.18 – 2.04 (m, 1H), 1.97 (ddt, *J* = 13.6, 9.9, 6.4 Hz, 1H), 1.74 – 1.64 (m, 4H), 1.61 – 1.51 (m, 2H) ppm.

^{13}**C NMR** (101 MHz, $CDCl_3$): δ_C 152.01, 151.33, 149.67, 134.64, 126.89, 124.10, 116.50, 73.41, 50.68, 39.05, 31.63, 25.90, 24.38 ppm.

IR (film): v_{max} 3155, 2932, 2851, 2812, 2227, 1609, 1514, 1236, 1129, 1004 cm^{-1}.

HRMS (ESI$^+$): calc'd for $[M+H]^+$,297.1961; found 297.1956.

1-(4-Morpholinophenyl)-3-(pyridin-4-yl)propan-1-ol (14)

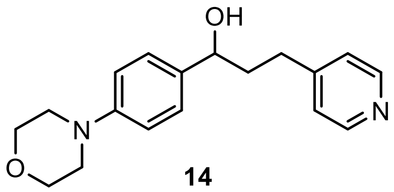

14

Prepared following **General Procedure A** and the Work-Up Procedure, using 4-(4-morpholinyl) benzaldehyde (47.8 mg). Purification by flash column chromatography (5:95 MeOH/DCM) gave **14** (55.8 mg, 75%) as an off-white solid.

$\mathbf{R_f}$ = 0.17 (5:95 MeOH/DCM, CAM).

NMR Spectroscopy (*see spectra*):

^{1}**H NMR** (400 MHz, $CDCl_3$): δ_H 8.47 – 8.41 (m, 2H), 7.28 – 7.20 (m, 2H), 7.12 – 7.06 (m, 2H), 6.93 – 6.83 (m, 2H), 4.59 (dd, *J* = 7.8, 5.5 Hz, 1H), 3.88 – 3.81 (m, 4H), 3.17 – 3.11 (m, 4H), 2.72 (ddd, *J* = 14.0, 9.9, 5.8 Hz, 1H), 2.63 (ddd, *J* = 14.0, 9.6, 6.4 Hz, 1H), 2.14 (br. s, 1H), 2.12 (dddd, *J* = 13.6, 9.6, 7.8, 5.8 Hz, 1H), 1.98 (dddd, *J* = 13.6, 9.9, 6.4, 5.5 Hz, 1H) ppm.

^{13}**C NMR** (101 MHz, $CDCl_3$): δ_C 151.13, 151.10, 149.80, 135.68, 127.03, 124.06, 115.77, 73.31, 67.00, 49.39, 39.13, 31.59 ppm.

IR (film): ν_{max} 3202, 2953, 2910, 2855, 1606, 1513, 1422, 1230, 1119, 1068 cm^{-1}.

HRMS (ESI$^+$): calc'd for $[M+H]^+$,299.1754; found 299.1747.

***tert*-Butyl 4-(4-(1-hydroxy-3-(pyridin-4-yl)propyl)phenyl)piperazine-1-carboxylate (15)**

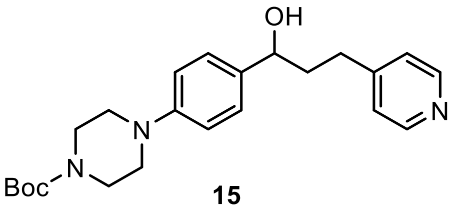

15

Prepared following **General Procedure A** and the Work-Up Procedure, using *tert*-butyl 4-(4-formylphenyl)piperazine-1-carboxylate (72.6 mg). Purification by flash column chromatography (5:95 MeOH/DCM) gave **15** (83.6 mg, 82%) as a yellow oil.

$\mathbf{R_f}$ = 0.21 (5:95 MeOH/DCM, CAM).

NMR Spectroscopy (*see spectra*):

^{1}**H NMR** (500 MHz, $CDCl_3$): δ_H 8.48 – 8.43 (m, 2H), 7.28 – 7.22 (m, 2H), 7.13 – 7.09 (m, 2H), 6.95 – 6.88 (m, 2H), 4.61 (dd, *J* = 7.8, 5.5 Hz, 1H), 3.61 – 3.55 (m, 4H), 3.13 (t, *J* = 5.2 Hz, 4H), 2.74 (ddd, *J* = 15.3, 9.9, 5.7 Hz, 1H), 2.65 (ddd, *J* = 15.3, 9.7, 6.4 Hz, 1H), 2.28 (s, 1H), 2.13 (dddd, *J* = 13.5, 9.7, 7.8, 5.7 Hz, 1H), 2.00 (ddt, *J* = 13.5, 9.9, 6.4 Hz, 1H), 1.49 (s, 9H) ppm.

^{13}C NMR (126 MHz, $CDCl_3$): δ_C 154.85, 151.14, 151.06, 149.78, 135.94, 127.02, 124.05, 116.66, 80.09, 73.28, 49.47, 39.16, 31.59, 28.56 ppm.

IR (film): v_{max} 3207, 2971, 2922, 2854, 2824, 2244, 2227, 1694, 1674, 1606, 1515, 1418, 1366, 1229, 1170, 1125, 1067 cm^{-1}.

HRMS (ESI$^+$): calc'd for $[M+H]^+$, 398.2438; found 398.2430.

1-(4-Fluorophenyl)-3-(pyridin-4-yl)propan-1-ol (16)

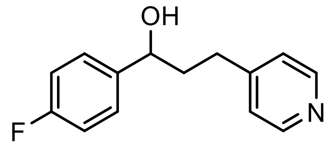

16

Prepared following **General Procedure A** and the Work-Up Procedure, using 4-fluorobenzaldehyde (27 µL). Purification by flash column chromatography (5:95 MeOH/DCM) gave **16** (31.8 mg, 55%) as a white solid.

R$_f$ = 0.12 (5:95 MeOH/DCM, CAM).

NMR Spectroscopy (*see spectra*):

^{1}H NMR (400 MHz, $CDCl_3$): δ_H 8.45 – 8.39 (m, 2H), 7.35 – 7.25 (m, 2H), 7.12 – 7.07 (m, 2H), 7.07 – 6.97 (m, 2H), 4.66 (dd, J = 7.9, 5.2 Hz, 1H), 2.73 (ddd, J = 15.2, 9.9, 5.7 Hz, 1H), 2.65 (ddd, J = 15.2, 9.6, 6.5 Hz, 1H), 2.09 (dddd, J = 13.6, 9.6, 7.9, 5.7 Hz, 1H), 1.96 (dddd, J = 13.6, 9.9, 6.5, 5.2 Hz, 1H) ppm.

^{13}C NMR (101 MHz, $CDCl_3$): δ_C 162.41 (d, J = 245.7 Hz), 150.98, 149.75, 140.23 (d, J = 3.1 Hz), 127.62 (d, J = 8.2 Hz), 124.07, 115.56 (d, J = 21.3 Hz), 72.95, 39.51, 31.45 ppm.

^{19}F NMR (377 MHz, not ^{19}F{^{1}H} decoupled, $CDCl_3$): δ_F −114.53 (tt, J = 8.6, 5.3 Hz, 1F) ppm.

HRMS (ESI$^+$): calc'd for $[M+H]^+$, 232.1132; found 232.1123.

All recorded spectroscopic data matched those previously reported in the literature.[27]

1-(4-Chlorophenyl)-3-(pyridin-4-yl)propan-1-ol (17)

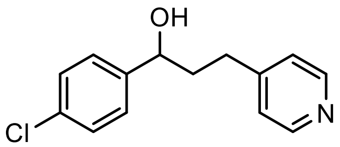

17

Prepared following **General Procedure A** and the Work-Up Procedure, using 4-chlorobenzaldehyde (35.1 mg). Purification by flash column chromatography (5:95 MeOH/DCM) gave **17** (16.0 mg, 26%) as a white solid.

R$_f$ = 0.17 (5:95 MeOH/DCM, CAM).

NMR Spectroscopy (*see spectra*):

^{1}H NMR (400 MHz, $CDCl_3$): δ_H 8.43 (d, *J* = 5.1 Hz, 2H), 7.32 (d, *J* = 8.6 Hz, 2H), 7.27 (d, *J* = 8.6 Hz, 2H), 7.11 (d, *J* = 5.1 Hz, 2H), 4.66 (dd, *J* = 7.9, 5.1 Hz, 1H), 2.78-2.63 (m, 2H), 2.09 (dddd, *J* = 13.9, 9.7, 7.9, 5.7 Hz, 1H), 2.03 – 1.89 (m, 1H) ppm.

^{13}C NMR (101 MHz, $CDCl_3$): δ_C 151.08, 149.61, 143.00, 133.53, 128.86, 127.35, 124.12, 72.83, 39.44, 31.37 ppm.

HRMS (ESI$^+$): calc'd for $[M+H]^+$,248.0837; found 248.0829.

All recorded spectroscopic data matched those previously reported in the literature.[27]

1-(3-Bromophenyl)-3-(pyridin-4-yl)propan-1-ol (18)

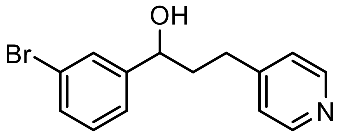

18

Prepared following **General Procedure A** and the Work-Up Procedure, using 3-bromobenzaldehyde (29 µL). Purification by flash column chromatography (5:95 MeOH/DCM) gave **18** (46.8 mg, 64%) as a colorless oil.

R_f = 0.25 (5:95 MeOH/DCM, CAM).

NMR Spectroscopy (*see spectra*):

^{1}H NMR (400 MHz, $CDCl_3$): δ_H 8.42 (d, *J* = 5.1 Hz, 2H), 7.50 (t, *J* = 1.8 Hz, 1H), 7.40 (dt, *J* = 7.5, 1.8 Hz, 1H), 7.37 – 7.16 (m, 2H), 7.13 – 7.07 (m, 2H), 4.64 (dd, *J* = 8.1, 4.9 Hz, 1H), 2.81 – 2.61 (m, 2H), 2.13 – 2.03 (m, 1H), 2.03 – 1.92 (m, 1H) ppm.

^{13}C NMR (101 MHz, $CDCl_3$): δ_C 150.96, 149.68, 146.93, 130.92, 130.31, 129.10, 124.56, 124.10, 122.86, 72.84, 39.42, 31.35 ppm.

HRMS (ESI$^+$): calc'd for $[M+H]^+$, 292.0332; found 292.0324.

All recorded spectroscopic data matched those previously reported in the literature.[27]

3-(Pyridin-4-yl)-1-(3-(trifluoromethyl)phenyl)propan-1-ol (19)

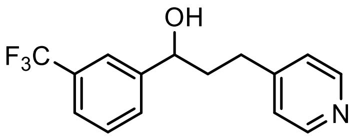

19

Prepared following a ***modified*** **General Procedure A**: A 7 mL vial was charged with eosin Y (3.2 mg, 5.0 µmol, 2.0 mol%), **sodium dithionite** (131 mg, 0.750 mmol, 3.00 equiv) and 3-(trifluoromethyl) benzaldehyde (43.5 mg, 33.5 µL, 0.250 mmol, 1.00 equiv). THF (1.0 mL), H_2O (3.5 mL) and NaOH (4 M in H_2O, 500 µL, 2.0 mmol, 8.0 equiv) were then added to the vial. Subsequently, the solution was degassed by sparing with N_2 for 5 min before addition of 4-vinylpyridine (80 µL, 0.75 mmol, 3.0 equiv). The mixture was then stirred in the dark for 1 h before irradiation with blue LEDs at room temperature for 16 h. The Work-Up Procedure was then followed before purification by flash column chromatography (5:95 MeOH/DCM) gave **19** (39.4 mg, 56%) as a colorless oil.

$\mathbf{R_f}$ = 0.32 (5:95 MeOH/DCM, CAM).

NMR Spectroscopy (*see spectra*):

> ^{1}**H NMR** (400 MHz, $CDCl_3$): δ_H 8.43 – 8.35 (m, 2H), 7.62 (s, 1H), 7.56 – 7.49 (m, 2H), 7.51 – 7.41 (m, 1H), 7.17 – 7.07 (m, 2H), 4.75 (dd, *J* = 8.2, 4.8 Hz, 1H), 3.24 (s, 1H), 2.84 – 2.64 (m, 2H), 2.17 – 2.07 (m, 1H), 2.07 – 1.94 (m, 1H) ppm.
>
> ^{13}**C NMR** (101 MHz, $CDCl_3$): δ_C 150.98, 149.62, 145.72, 131.01 (q, *J* = 32.2 Hz), 129.31, 129.15, 124.59 (q, *J* = 3.9 Hz), 124.22 (q, *J* = 272.4 Hz), 124.12, 122.76 (q, *J* = 3.9 Hz), 72.80, 39.55, 31.37 ppm.
>
> ^{19}**F NMR** (377 MHz, $CDCl_3$): δ_F −62.46 (s, 3F) ppm.

IR (film): ν_{max} 3179, 3074, 2932, 2865, 1606, 1419, 1327, 1162, 1121, 1070 cm^{-1}.

HRMS (ESI⁺): calc'd for $[M+H]^+$, 282.1100; found 282.1093.

3-(Pyridin-4-yl)-1-(4-(trifluoromethyl)phenyl)propan-1-ol (20)

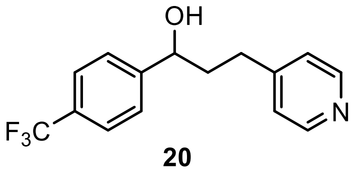

20

Prepared following **General Procedure A** and the Work-Up Procedure, using 4-trifluoromethylbenzaldehyde (43.5 mg). After work-up, the assay yield was determined by ^{1}H NMR analysis of the crude mixture to be 12% using 1,4-dioxane (8 µL, 0.094 mmol) as the internal standard. Purification by flash column chromatography (5:95 MeOH/DCM) gave **20** (7.9 mg, 11%) as a colorless oil containing a minor impurity.

$\mathbf{R_f}$ = 0.27 (5:95 MeOH/DCM, CAM).

NMR Spectroscopy (*see spectra*):

^{1}H NMR (400 MHz, $CDCl_3$): δ_H 8.44 (d, *J* = 6.3 Hz, 2H), 7.61 (d, *J* = 8.4 Hz, 2H), 7.47 (d, *J* = 8.3 Hz, 2H), 7.13 (d, *J* = 6.3 Hz, 2H), 4.76 (dd, *J* = 8.2, 4.8 Hz, 1H), 2.76 (s, 1H), 2.82-2.66 (m, 2H), 2.17 – 1.96 (m, 2H) ppm.

^{13}C{^{1}H, ^{19}F} NMR (101 MHz, $CDCl_3$): δ_C 151.32, 149.41, 148.52, 130.07, 126.24, 125.68, 124.21, 124.18, 72.84, 39.48, 31.33 ppm.

^{19}F NMR (377 MHz, $CDCl_3$): δ_F -62.36 (s, 3F) ppm.

IR (film): v_{max} 3216, 2925, 2850, 1606, 1417, 1325, 1162, 1121, 1067 cm^{-1}.

HRMS (ESI^{+}): calc'd for [M+H]$^{+}$, 282.1100 ; found 282.1095.

Note: For 4-trifluoromethylbenzaldehyde, the diminished nucleophilicity of the ketyl radical caused by the electron-withdrawing CF_3 group resulted in competitive formation of 1,2-bis(4-(trifluoromethyl)phenyl)ethane-1,2-diol through a pinacol coupling reaction (78% yield, 2:1 d.r., determined by ^{1}H NMR analysis).[25]

1-(4-(Hydroxymethyl)phenyl)-3-(pyridin-4-yl)propan-1-ol (21)

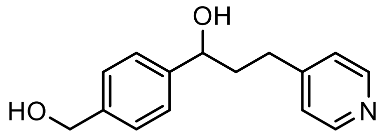

21

Prepared following a ***modified* General Procedure A** [THF (1 mL) was used instead of MeCN (1 mL)] and the Work-Up Procedure, using 4-(hydroxyl methyl) benzaldehyde (34.0 mg). Purification by flash column chromatography (5:95 to 10:90 MeOH/DCM) gave **21** (40.1 mg, 66%) as a colorless oil.

R_f = 0.29 (10:90 MeOH/DCM, CAM).

NMR Spectroscopy (*see spectra*):

^{1}H NMR (400 MHz, CD_3OD): δ_H 8.41 – 8.35 (m, 2H), 7.34 (s, 4H), 7.32 – 7.24 (m, 2H), 4.63 (dd, *J* = 7.7, 5.5 Hz, 1H), 4.59 (s, 2H), 2.77 (ddd, *J* = 13.9, 9.9, 5.8 Hz, 1H), 2.67 (ddd, *J* = 13.9, 9.7, 6.6 Hz, 1H), 2.14 – 1.93 (m, 2H) ppm.

^{13}C NMR (101 MHz, CD_3OD): δ_C 154.26, 149.86, 145.09, 141.89, 128.11, 127.06, 125.67, 73.95, 64.98, 40.77, 32.46 ppm.

IR (film): v_{max} 3254, 2919, 2861, 1606, 1419, 1220, 1042, 1004 cm^{-1}.

HRMS (ESI^{+}): calc'd for [M+H]$^{+}$,244.1332; found 244.1320.

3-(1-Hydroxy-3-(pyridin-4-yl)propyl)phenol (22)

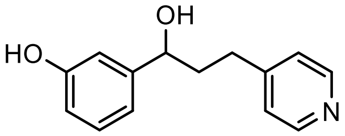

22

Prepared following **General Procedure A** and the Work-Up Procedure, using 3-hydroxyaldehyde (30.5 mg). Purification by flash column chromatography (5:95 to 10:90 MeOH/DCM) gave **22** (34.3 mg, 60%) as a white solid.

R_f = 0.43 (10:90 MeOH/DCM, CAM).

NMR Spectroscopy (*see spectra*):

> **^{1}H NMR** (400 MHz, CD_3OD): δ_H 8.39 – 8.33 (m, 2H), 7.27 – 7.21 (m, 2H), 7.10 (t, *J* = 8.1 Hz, 1H), 6.79 – 6.72 (m, 2H), 6.68 – 6.60 (m, 1H), 4.52 (dd, *J* = 7.5, 5.5 Hz, 1H), 2.73 (ddd, *J* = 13.8, 9.8, 5.5 Hz, 1H), 2.64 (ddd, *J* = 13.8, 9.6, 7.5 Hz, 1H), 2.09 – 1.88 (m, 2H) ppm.

> **^{13}C NMR** (101 MHz, CD_3OD): δ_C 158.60, 154.39, 149.78, 147.63, 130.41, 125.71, 118.21, 115.25, 113.81, 74.07, 40.65, 32.45 ppm.

IR (film): ν_{max} 3195, 3059, 2924, 2861, 2720, 1606, 1589, 1456, 1421, 1276, 1067, 1008 cm^{-1}.

HRMS (ESI$^+$): calc'd for $[M+H]^+$, 230.1176; found 230.1166.

3-(1-Hydroxy-3-(pyridin-4-yl)propyl)benzoic acid (23)

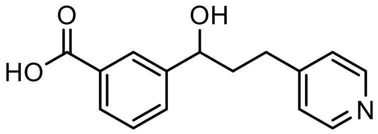

23

Prepared following a ***modified* General Procedure A**: A 7 mL vial was charged with eosin Y (3.2 mg, 5.0 µmol, 2.0 mol%), **sodium dithionite** (131 mg, 0.750 mmol, 3.00 equiv) and 3-carboxybenzaldehyde (37.5 mg, 0.250 mmol, 1.00 equiv). THF (1.0 mL), H_2O (3.5 mL) and NaOH (4 M in H_2O, 560 µL, 2.25 mmol, 9.00 equiv) were then added to the vial. Subsequently, the solution was degassed by sparging with N_2 for 5 min before addition of 4-vinylpyridine (80 µL, 0.75 mmol, 3.0 equiv). The mixture was then stirred in the dark for 1 h before irradiation with blue LEDs at room temperature for 16 h. The Work-Up Procedure was then followed before purification by flash column chromatography (5:95 to 15:85 MeOH/DCM) gave **23** (41.8 mg, 65%) as a light-yellow solid.

R_f = 0.32 (10:90 MeOH/DCM, CAM).

NMR Spectroscopy (*see spectra*):

^{1}**H NMR** (500 MHz, CD_3OD): δ_H 8.40 (d, *J* = 5.1 Hz, 2H), 8.03 (s, 1H), 7.92 (d, *J* = 7.7 Hz, 1H), 7.57 (d, *J* = 7.6 Hz, 1H), 7.43 (t, *J* = 7.7 Hz, 1H), 7.30 (d, *J* = 5.0 Hz, 2H), 4.71 (t, *J* = 6.7 Hz, 1H), 2.81 (ddd, *J* = 15.1, 9.5, 5.8 Hz, 1H), 2.72 (dt, *J* = 15.1, 7.8 Hz, 1H), 2.14 – 1.99 (m, 2H) ppm.

^{13}**C NMR** (126 MHz, CD_3OD): δ_C 171.32, 154.31, 149.74, 146.50, 133.73, 131.02, 129.63, 129.39, 128.24, 125.73, 73.71, 40.67, 32.41 ppm.

IR (film): ν_{max} 3371, 3070, 2926, 2858, 2616, 2504, 1923, 1693, 1608, 1558, 1392, 1266, 1202, 1055 cm^{-1}.

HRMS (ESI$^+$): calc'd for $[M+H]^+$,258.1125; found 258.1120.

1-(Furan-2-yl)-3-(pyridin-4-yl)propan-1-ol (24)

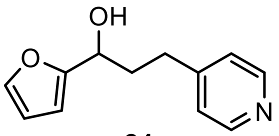

24

Prepared following **General Procedure A** and the Work-Up Procedure, using furfural (20.5 µL). Purification by flash column chromatography (5:95 MeOH/DCM) gave **24** (36.5 mg, 72%) as a yellow oil.

R$_f$ = 0.20 (5:95 MeOH/DCM, CAM).

NMR Spectroscopy (*see spectra*):

^{1}**H NMR** (400 MHz, $CDCl_3$): δ_H 8.47 – 8.38 (m, 2H), 7.37 – 7.32 (m, 1H), 7.14 – 7.07 (m, 2H), 6.31 (dd, *J* = 3.2, 1.8 Hz, 1H), 6.22 (d, *J* = 3.2 Hz, 1H), 4.66 (dd, *J* = 7.4, 6.1 Hz, 1H), 3.48 (s, 1H), 2.82 – 2.62 (m, 2H), 2.22-2.09 (m, 2H) ppm.

^{13}**C NMR** (101 MHz, $CDCl_3$): δ_C 156.56, 151.10, 149.53, 142.14, 124.16, 110.32, 106.18, 66.65, 35.98, 31.19 ppm.

HRMS (ESI$^+$): calc'd for $[M+H]^+$, 204.1019; found 204.1011.

All recorded spectroscopic data matched those previously reported in the literature.[27]

1-(6-Methoxypyridin-3-yl)-3-(pyridin-4-yl)propan-1-ol (25)

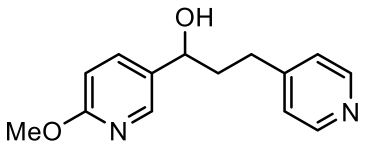

25

Prepared following **General Procedure A** and the Work-Up Procedure, using 6-methoxy-3-pyridinecarboxaldehyde (34.3 mg). Purification by flash column chromatography (5:95 MeOH/DCM) gave **25** (39.1 mg, 64%) as a white solid.

R_f = 0.28 (5:95 MeOH/DCM, CAM).

NMR Spectroscopy (<u>see spectra</u>):

^{1}H NMR (400 MHz, $CDCl_3$): δ_H 8.46 – 8.40 (m, 2H), 8.06 (d, *J* = 1.9 Hz, 1H), 7.59 (dd, *J* = 8.6, 2.5 Hz, 1H), 7.14 – 7.06 (m, 2H), 6.73 (d, *J* = 8.0 Hz, 1H), 4.63 (dd, *J* = 7.8, 5.5 Hz, 1H), 3.91 (s, 3H), 2.73 (ddd, *J* = 14.1, 9.9, 5.4 Hz, 1H), 2.63 (ddd, *J* = 14.1, 9.6, 6.4 Hz, 1H), 2.62 (br. s, 1H), 2.12 (dddd, *J* = 13.7, 9.6, 7.8, 5.4 Hz, 1H), 1.96 (dddd, *J* = 13.7, 9.9, 6.4, 5.5 Hz, 1H) ppm.

^{13}C NMR (101 MHz, $CDCl_3$): δ_C 164.17, 150.79, 149.82, 144.84, 136.66, 132.43, 124.05, 111.25, 71.06, 53.64, 39.11, 31.43 ppm.

IR (film): ν_{max} 3162, 3005, 2870, 2844, 1606, 1490, 1286, 1078, 1024 cm^{-1}.

HRMS (ESI$^+$): calc'd for $[M+H]^+$, 245.1285; found 245.1279.

2-Phenyl-4-(pyridin-4-yl)butan-2-ol (26)

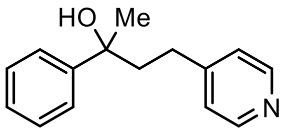

26

Prepared following **General Procedure A** and the Work-Up Procedure, using acetophenone (29 µL). Purification by flash column chromatography (5:95 MeOH/DCM) gave **26** (15.2 mg, 27%) as a yellow oil. The yield of recovered acetophenone was determined to be 70% by GC analysis.

R_f = 0.14 (5:95 MeOH/DCM, CAM).

NMR Spectroscopy (<u>see spectra</u>):

^{1}H NMR (500 MHz, $CDCl_3$): δ_H 8.42 (d, *J* = 5.4 Hz, 2H), 7.50 – 7.43 (m, 2H), 7.42 – 7.32 (m, 2H), 7.31 – 7.22 (m, 1H), 7.09 – 7.03 (m, 2H), 2.67 (ddd, *J* = 14.0, 10.0, 7.1 Hz, 1H), 2.42 (ddd, *J* = 14.0, 10.2, 6.6 Hz, 1H), 2.14 – 2.07 (m, 2H), 1.64 (s, 3H) ppm.

^{13}C NMR (126 MHz, $CDCl_3$): δ_C 152.36, 149.10, 147.27, 128.51, 127.00, 124.85, 124.14, 74.48, 44.90, 30.76, 30.05 ppm.

IR (film): ν_{max} 3210, 3059, 3026, 2973, 2929, 2966, 1603, 1445, 1418, 1066 cm^{-1}.

HRMS (ESI$^+$): calc'd for $[M+H]^+$, 228.1383; found 228.1373.

2.4.2. Olefin Scope

3-(2-Fluoropyridin-4-yl)-1-phenylpropan-1-ol (27)

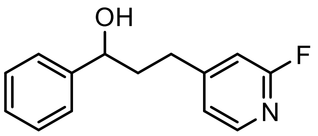

27

Prepared following **General Procedure B** and the Work-Up Procedure, using 2-fluoro-4-vinylpyridine (46.2 mg). Purification by flash column chromatography (5:95 MeOH/DCM) gave **27** (38.1 mg, 66%) as a yellow oil.

R_f = 0.41 (5:95 MeOH/DCM, CAM).

NMR Spectroscopy (*see spectra*):

^{1}H NMR (400 MHz, $CDCl_3$): δ_H 7.98 (d, J = 5.2 Hz, 1H), 7.31 – 7.17 (m, 5H), 6.91 (dt, J = 5.2, 1.8 Hz, 1H), 6.66 (s, 0H), 4.60 (ddd, J = 7.9, 5.1, 2.7 Hz, 1H), 2.72 (ddd, J = 14.1, 9.9, 5.7 Hz, 1H), 2.63 (ddd, J = 14.2, 9.6, 6.6 Hz, 1H), 2.11 (d, J = 3.1 Hz, 1H), 2.04 (dddd, J = 13.7, 9.7, 8.0, 5.7 Hz, 1H), 1.93 (dddd, J = 13.7, 9.9, 6.6, 5.1 Hz, 1H) ppm.

^{13}C NMR (101 MHz, $CDCl_3$): δ_C 164.21 (d, J = 238.4 Hz), 157.17 (d, J = 7.7 Hz), 147.43 (d, J = 15.0 Hz), 144.16, 128.80, 128.07, 125.92, 121.80 (d, J = 3.9 Hz), 109.29 (d, J = 6.6 Hz), 73.54, 39.10, 31.36 (d, J = 2.8 Hz) ppm.

^{19}F NMR (377 MHz, $CDCl_3$): δ_F = −68.98 (s, 1F) ppm

IR (film): ν_{max} 3334, 3062, 3028, 2943, 2855, 1613, 1559, 1411, 1278, 1148, 1060 cm^{-1}.

HRMS (ESI$^+$): calc'd for $[M+H]^+$, 232.1132; found 232.1124.

3-(2-Chloropyridin-4-yl)-1-phenylpropan-1-ol (28)

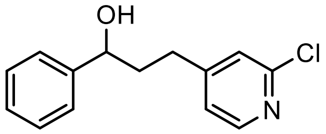

28

Prepared following **General Procedure B** and the Work-Up Procedure, using 2-chloro-4-vinylpyridine (52.3 mg). Purification by flash column chromatography (5:95 MeOH/DCM) gave **28** (45.8 mg, 74%) as a yellow oil.

R_f = 0.40 (5:95 MeOH/DCM, CAM).

NMR Spectroscopy (*see spectra*):

^{1}H NMR (400 MHz, $CDCl_3$): δ_H 8.23 (dd, J = 5.1, 0.8 Hz, 1H), 7.40 – 7.27 (m, 5H), 7.16 (dd, J = 1.5, 0.8 Hz, 1H), 7.04 (dd, J = 5.1, 1.5 Hz, 1H), 4.68 (dd, J = 8.0, 5.1 Hz, 1H), 2.76 (ddd, J = 14.1, 9.9, 5.6 Hz, 1H),

2.67 (ddd, J = 14.1, 9.6, 6.6 Hz, 1H), 2.17 – 2.06 (m, 1H), 2.10 (br. s, 1H), 2.00 (dddd, J = 13.7, 9.9, 6.6, 5.1 Hz, 1H) ppm.

^{13}C NMR (101 MHz, $CDCl_3$): δ_C 154.54, 151.78, 149.60, 144.14, 128.82, 128.10, 125.92, 124.37, 122.86, 73.57, 39.12, 31.22 ppm.

IR (film): ν_{max} 3333, 3061, 3029, 2925, 2854, 2240, 1593, 1546, 1387, 1360, 1202, 1085 cm^{-1}.

HRMS (ESI$^+$): calc'd for [M+H]$^+$, 248.0837; found 248.0828.

1-Phenyl-3-(2-(trifluoromethyl)pyridin-4-yl)propan-1-ol (29)

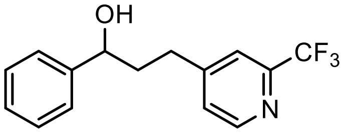

29

Prepared following **General Procedure B** and the Work-Up Procedure, using 2-(trifluoromethyl)-4-vinylpyridine (65 mg). Purification by flash column chromatography (5:95 MeOH/DCM) gave **29** (56.9 mg, 81%) as a yellow oil.

R$_f$ = 0.40 (5:95 MeOH/DCM, CAM).

NMR Spectroscopy (*see spectra*):

^{1}H NMR (400 MHz, $CDCl_3$): δ_H 8.58 (d, J = 5.0 Hz, 1H), 7.51 (d, J = 1.6 Hz, 1H), 7.41 – 7.27 (m, 6H), 4.70 (dd, J = 7.9, 5.1 Hz, 1H), 2.87 (ddd, J = 15.3, 10.1, 5.6 Hz, 1H), 2.77 (ddd, J = 14.3, 9.8, 6.5 Hz, 1H), 2.22 – 1.97 (m, 2H), 2.07 (br.s, 1H) ppm.

^{13}C NMR (101 MHz, $CDCl_3$): δ_C 153.17, 150.02, 148.40 (q, 34.1 Hz), 144.04, 128.86, 128.17, 126.61, 125.91, 121.78 (q, 273.9 Hz), 120.76 (q, 2.8 Hz), 73.62, 39.15, 31.55 ppm.

^{19}F NMR (377 MHz, $CDCl_3$): δ_F = −67.88 (s, 3F) ppm.

IR (film): ν_{max} 3360, 3063, 3031, 2924, 2868, 1610, 1431, 1330, 1178, 1133, 1085, 1061 cm^{-1}.

HRMS (ESI$^+$): calc'd for [M+H]$^+$,282.1100; found 282.1092.

1-Phenyl-3-(pyridin-4-yl)butan-1-ol (30)

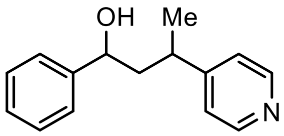

30

Prepared following **General Procedure B** and the Work-Up Procedure, using 4-(prop-1-en-2-yl) pyridine (45 mg). Purification by flash column chromatography (5:95 MeOH/DCM) gave **30** (22.7 mg, 40%, 1:1 d.r.) as a yellow oil.

R$_f$ = 0.32 (5:95 MeOH/DCM, CAM).

NMR Spectroscopy (*see spectra*):

^{1}**H NMR** (400 MHz, $CDCl_3$): δ_H 8.48 – 8.40 (m, 2H), 7.37 – 7.19 (m, 5H), 7.17 – 7.12 and 7.11 – 7.06 (m; m; 2H), 4.57 and 4.34 (dd, *J* = 7.7, 6.3 Hz; dd, *J* = 9.8, 3.8 Hz; 1H), 3.04 and 2.76 (dqd, *J* = 10.0, 7.0, 4.9 Hz; h, *J* = 7.1 Hz; 1H), 2.48 (br. s, 1H), 2.20 – 2.00 and 1.95 – 1.82 (m; m; 2H), 1.26 (dd, *J* = 6.9, 3.7 Hz, 3H) ppm.

^{13}**C NMR** (101 MHz, $CDCl_3$): δ_C 156.44, 155.99, 149.86, 145.02, 144.49, 128.76, 128.69, 128.02, 127.80, 126.20, 125.75, 122.96, 122.71, 72.40, 72.12, 46.98, 46.43, 36.30, 36.02, 22.32, 21.62 ppm.

IR (film): ν_{max} 3205, 3062, 3027, 2951, 2927, 2871, 1601, 1453, 1416, 1054 cm^{-1}.

HRMS (ESI$^+$): calc’d for $[M+H]^+$, 228.1383; found 228.1373.

1,3-Diphenyl-3-(pyridin-4-yl)propan-1-ol (31)

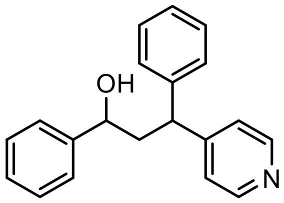

31

Prepared following **General Procedure B** and the Work-Up Procedure, using 4-(1-phenylvinyl) pyridine (68 mg). Purification by flash column chromatography (5:95 MeOH/DCM) gave **31** (53.4 mg, 74%, 1:1 d.r.) as a colorless oil.

R$_f$ = 0.2 (5:95 MeOH/DCM, CAM).

NMR Spectroscopy (*see spectra*):

^{1}**H NMR** (400 MHz, $CDCl_3$): δ_H 8.42 – 8.41 and 8.40 – 8.38 (m; m; 2H), 7.37 – 7.19 (m, 10H), 7.17 – 7.15 and 7.14 – 7.13 (m; m; 2H), 4.45 and 4.43 (dd, *J* = 9.7, 4.5 Hz; dd, *J* = 8.6, 5.3 Hz; 1H), 4.21 and 4.10 (dd, *J* = 9.8, 5.8 Hz; dd, *J* = 9.0, 6.7 Hz; 1H), 2.70 (br. s, 1H), 2.57 – 2.31 (m, 2H) ppm.

^{13}C NMR (101 MHz, $CDCl_3$): δ_C 154.01, 153.51, 149.90, 149.81, 144.73, 144.47, 142.89, 142.36, 128.99, 128.91, 128.78, 128.75, 128.29, 128.05, 127.97, 127.94, 127.08, 126.99, 126.10, 125.92, 123.53, 123.29, 72.11, 71.79, 47.09, 47.07, 44.19, 44.12 ppm.

IR (film): ν_{max} 3197, 3061, 3027, 2919, 1597, 1493, 1452, 1417, 1059 cm^{-1}.

HRMS (ESI$^+$): calc'd for $[M+H]^+$, 290.1539; found 290.1531.

Phenyl(2-(2-(trifluoromethyl)pyridin-4-yl)cyclopentyl)methanol (32)

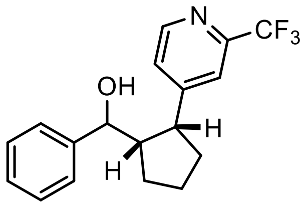

32

Prepared following **General Procedure B** and the Work-Up Procedure, using 4-(cyclopent-1-en-1-yl)-2-(trifluoromethyl) pyridine (**S1**) (80 mg). Purification by flash column chromatography (1:40 MeOH/DCM) gave **32** (31.3 mg, 39%, 1.4:1 d.r.) as a yellow oil.

R_f = 0.30 (1:40 MeOH/DCM, CAM).

NMR Spectroscopy (*see spectra*):

^{1}H NMR (500 MHz, $CDCl_3$): δ_H 8.54 (d, J = 5.0 Hz, 0.58H) and 8.46 (d, J = 5.0 Hz, 0.42H), 7.48 (d, J = 1.7 Hz, 0.58H), 7.34 – 7.25 (m, 4H), 7.21 – 7.19 (m, 1H), 7.19 – 7.15 (m, 1H), 7.09 (dd, J = 5.1, 1.6 Hz, 0.42H), 4.67 (dd, J = 7.4, 2.7 Hz, 0.58H) and 4.51 (dd, J = 6.8, 2.3 Hz, 0.42H), 3.21 (q, J = 8.2 Hz, 0.58H) and 2.90 (q, J = 9.0 Hz, 0.42H), 2.54 (p, J = 7.6 Hz, 0.58H) and 2.43 (dtd, J = 9.2, 8.0, 6.7 Hz, 0.42H), 2.25 – 2.13 (m, 1H), 2.09 – 2.00 (m, 1H), 1.98 – 1.69 (m, 4.42H), 1.63 – 1.55 (m, 0.58H) ppm.

^{13}C NMR (126 MHz, $CDCl_3$): δ_C 158.77, 157.30, 149.75, 149.71, 148.06 (q, J = 33.9 Hz), 148.00 (q, J = 34.2 Hz), 143.38, 143.09, 128.52, 128.47, 128.13, 127.98, 126.49, 126.39, 125.66, 125.45, 121.86 (q, J = 274.7 Hz), 121.71 (q, J = 275.9 Hz), 119.89 (q, J = 2.7 Hz), 119.80 (q, J = 2.7 Hz), 77.98, 76.69, 55.43, 54.55, 48.15, 48.13, 36.64, 36.38, 30.20, 28.84, 25.76, 25.20 ppm.

^{19}F NMR (377 MHz, $CDCl_3$): δ_F = −67.74 and −67.84 (s, 3F) ppm.

NOESY spectrum identified the *cis* configuration of the cyclopentane ring, which is consistent with the literature.[28]

IR (film): ν_{max} 3375, 3061, 3030, 2956, 2871, 1608, 1431, 1331, 1180, 1135, 1085 cm^{-1}.

HRMS (ESI$^+$): calc'd for $[M+H]^+$,322.1413; found 322.1398.

1-Phenyl-3-(pyridin-2-yl)propan-1-ol (33)

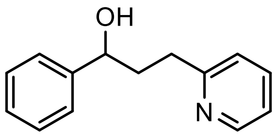

33

Prepared following **General Procedure B** and the Work-Up Procedure, using 2-vinyl pyridine (40.5 µL). Purification by flash column chromatography (5:95 MeOH/DCM) gave **33** (13.3 mg, 25%) as a yellow oil.

R$_f$ = 0.25 (5:95 MeOH/DCM, CAM).

NMR Spectroscopy (*see spectra*):

^{1}**H NMR** (400 MHz, $CDCl_3$): δ_H 8.51 (ddd, *J* = 5.0, 1.9, 0.9 Hz, 1H), 7.61 (td, *J* = 7.7, 1.8 Hz, 1H), 7.50 – 7.36 (m, 2H), 7.39 – 7.28 (m, 2H), 7.25 – 7.22 (m, 1H), 7.19 – 7.12 (m, 2H), 4.82 (dd, *J* = 7.8, 4.4 Hz, 1H), 2.99 (t, *J* = 6.7 Hz, 2H), 2.28 – 2.10 (m, 2H) ppm.

^{13}**C NMR** (101 MHz, $CDCl_3$): δ_C 161.49, 148.65, 145.36, 137.07, 128.41, 127.19, 125.97, 123.43, 121.37, 73.81, 38.13, 34.55 ppm.

HRMS (ESI$^+$): calc'd for $[M+H]^+$, 214.1226; found 214.1223.

All recorded spectroscopic data matched those previously reported in the literature.[27]

1-Phenyl-3-(pyrimidin-2-yl)propan-1-ol (34)

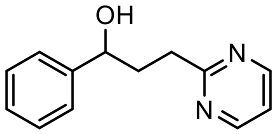

34

Prepared following **General Procedure B** and the Work-Up Procedure, using 2-vinylpyrimidine (40 mg). Purification by flash column chromatography (5:95 MeOH/DCM) gave **34** (42.4 mg, 79%) as a yellow oil.

R$_f$ = 0.30 (5:95 MeOH/DCM, CAM).

NMR Spectroscopy (*see spectra*):

^{1}**H NMR** (400 MHz, $CDCl_3$): δ_H 8.66 (d, *J* = 5.0 Hz, 2H), 7.42 – 7.36 (m, 2H), 7.36 – 7.28 (m, 2H), 7.29 – 7.20 (m, 1H), 7.15 (t, *J* = 4.9 Hz, 1H), 4.83 (dd, *J* = 7.4, 5.0 Hz, 1H), 4.21 (br. s, 1H), 3.16 (t, *J* = 6.9 Hz, 2H), 2.35 – 2.19 (m, 2H) ppm.

^{13}**C NMR** (101 MHz, $CDCl_3$): δ_C 171.12, 157.07, 144.93, 128.48, 127.36, 125.92, 118.71, 73.98, 37.07, 36.07 ppm.

IR (film): ν_{max} 3237, 3029, 2970, 2854, 1565, 1423, 1366, 1217, 1088 cm^{-1}.

HRMS (ESI$^+$): calc'd for $[M+H]^+$, 215.1179; found 215.1169.

3-(4-(Methylsulfonyl)phenyl)-1-phenylpropan-1-ol (35)

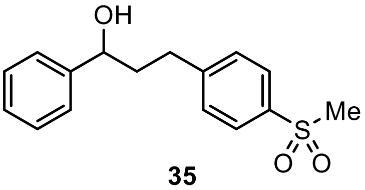

35

Prepared following **General Procedure B** and the Work-Up Procedure, using 1-(methyl sulfonyl)-4-vinylbenzene (68.4 mg). Purification by flash column chromatography (1:40 MeOH/DCM) gave **35** (45.0 mg, 62%) as a yellow oil.

R$_f$ = 0.17 (1:40 MeOH/DCM, CAM).

NMR Spectroscopy (*see spectra*):

^{1}H NMR (400 MHz, $CDCl_3$): δ_H 7.86 – 7.80 (m, 2H), 7.39 – 7.32 (m, 6H), 7.31 – 7.27 (m, 1H), 4.68 (dd, *J* = 7.9, 5.2 Hz, 1H), 3.02 (s, 3H), 2.85 (ddd, *J* = 13.9, 9.9, 5.5 Hz, 1H), 2.77 (ddd, *J* = 13.9, 9.7, 6.5 Hz, 1H), 2.14 (dddd, *J* = 13.6, 9.7, 7.9, 5.5 Hz, 1H), 2.07 (br.s, 1H), 2.02 (dddd, *J* = 13.6, 9.9, 6.5, 5.2 Hz, 1H) ppm.

^{13}C NMR (101 MHz, $CDCl_3$): δ_C 148.74, 144.31, 138.20, 129.54, 128.75, 127.98, 127.62, 125.96, 73.69, 44.68, 40.08, 32.12 ppm.

IR (film): v_{max} 3496, 3062, 3029, 2926, 2865, 2253, 1597, 1408, 1296, 1144, 1088 cm^{-1}.

HRMS (ESI$^+$): calc'd for [M+H]$^+$,291.1049; found 291.1051.

4-(3-Hydroxy-3-phenylpropyl)-*N*,*N*-dimethylbenzenesulfonamide (36)

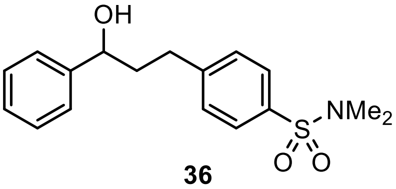

36

Prepared following **General Procedure B** and the Work-Up Procedure, using *N*,*N*-dimethyl-4-vinylbenzenesulfonamide (79.2 mg). Purification by flash column chromatography (5:95 MeOH/DCM) gave **36** (33.6 mg, 42%) as a yellow oil.

R$_f$ = 0.6 (5:95 MeOH/DCM, CAM).

NMR Spectroscopy (*see spectra*):

^{1}H NMR (400 MHz, $CDCl_3$): δ_H 7.70 – 7.64 (m, 2H), 7.40 – 7.27 (m, 7H), 4.69 (dd, *J* = 8.0, 5.3 Hz, 1H), 2.85 (ddd, *J* = 14.0, 10.0, 5.6 Hz, 1H), 2.75 (ddd, *J* = 14.0, 8.8, 6.4 Hz, 1H), 2.68 (s, 6H), 2.14 (dddd, *J* = 13.6, 9.9, 7.9, 5.7 Hz, 1H), 2.08 – 1.98 (m, 1H), 2.00 (br.s, 1H) ppm.

^{13}C NMR (126 MHz, $CDCl_3$): δ_C 147.49, 144.37, 133.09, 129.17, 128.76, 128.05, 127.98, 125.97, 73.81, 40.13, 38.07, 32.08 ppm.

IR (film): v_{max} 3517, 3062, 3028, 2922, 2894, 1597, 1455, 1337, 1158, 1090 cm^{-1}.

HRMS (ESI$^+$): calc'd for [M+H]$^+$, 320.1315; found 320.1304.

4-(3-Hydroxy-3-phenylpropyl)benzonitrile (37)

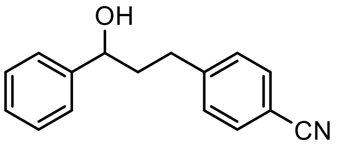

37

Prepared following **General Procedure B** and the Work-Up Procedure, using 4-cyanostyrene (48.4 mg). Purification by flash column chromatography (1:100 MeOH/DCM) gave **37** (36.0 mg, 61%) as a yellow oil.

R$_f$ = 0.27 (1:100 MeOH/DCM, CAM).

NMR Spectroscopy (*see spectra*):

> **^{1}H NMR** (400 MHz, CDCl$_3$): δ$_H$ 7.60 – 7.50 (m, 2H), 7.43 – 7.25 (m, 7H), 4.68 (ddd, *J* = 7.6, 5.1, 2.1 Hz, 1H), 2.82 (ddd, *J* = 14.0, 9.9, 5.7 Hz, 1H), 2.73 (ddd, *J* = 14.0, 9.6, 6.5 Hz, 1H), 2.12 (dddd, *J* = 13.6, 9.7, 7.9, 5.7 Hz, 1H), 2.06 – 1.97 (m, 1H), 1.96 (t, *J* = 2.1 Hz, 1H) ppm.

> **^{13}C NMR** (101 MHz, CDCl$_3$): δ$_C$ 147.74, 144.29, 132.35, 129.39, 128.78, 128.02, 125.94, 119.22, 109.86, 73.74, 39.98, 32.32 ppm.

IR (film): v_{max} 3431, 3062, 3031, 2931, 2864, 2227, 1606, 1453, 1059 cm^{-1}.

HRMS (ESI$^+$): calc'd for [M+H]$^+$, 238.1226; found 238.1224.

4-Hydroxy-*N*,2,4-triphenylbutanamide (39)

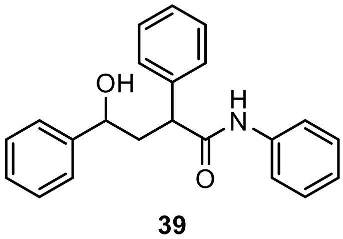

39

Prepared following **General Procedure B**, using *N*,2-diphenylacrylamide (83.7 mg). The solvent was removed *in vacuo*, and the crude product was purified without work-up. Purification by flash column chromatography (2:100 to 10:90 MeOH/DCM) gave **39** (68.5 mg, 83%, 1:1 d.r.) as a yellow solid.

R$_f$ = 0.25 (2:100 MeOH/DCM, CAM).

NMR Spectroscopy (*see spectra*):

^{1}H NMR (400 MHz, $CDCl_3$): δ_H 7.47 – 7.15 (m, 15H), 7.12 – 7.04 (m, 1H), 4.83 and 4.59 (dd, J = 8.4, 3.8 Hz; dt, J = 9.9, 3.8 Hz; 1H), 3.85 and 3.79 (t, J = 7.1 Hz; q, J = 4.3 Hz; 1H), 2.90 and 2.86 (d, J = 3.8 Hz; d, J = 3.8 Hz; 1H), 2.75 – 2.63 (m, 1H), 2.26 – 2.12 (m, 1H) ppm.

^{13}C NMR (126 MHz, $CDCl_3$): δ_C 172.28, 171.93, 144.65, 144.34, 139.73, 139.49, 137.83, 137.77, 129.41, 129.27, 129.09, 128.67, 128.40, 128.14, 127.96, 127.81, 127.77, 125.94, 124.61, 120.04, 120.01, 72.80, 72.24, 51.62, 50.49, 42.79, 42.75 ppm.

IR (film): v_{max} 3305, 3061, 3015, 2923, 1664, 1599, 1541, 1496, 1442, 1311, 1251, 1177, 1056, 1031 cm^{-1}.

HRMS (ESI$^+$): calc'd for $[M+H]^+$, 332.1645; found 332.1640.

4-Hydroxy-*N*,2-diphenyl-4-(4-(trifluoromethyl)phenyl)butanamide (40)

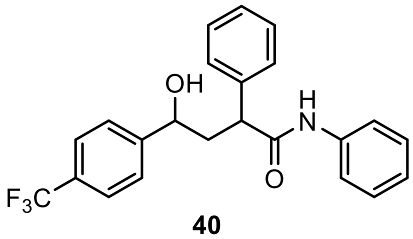

40

A 7 mL vial was charged with eosin Y (3.2 mg, 5.0 µmol, 2.0 mol%), TDO (41 mg, 0.38 mmol, 1.5 equiv) and *N*,2-diphenylacrylamide (83.7 mg, 0.38 mmol, 1.5 equiv). MeCN (2.5 mL), H_2O (2.25 mL) and NaOH (4 M in H_2O, 0.25 mL, 1.0 mmol, 4.0 equiv) were then added to the vial. Subsequently, the solution was degassed by sparging with N_2 for 5 min before addition of 4-trifluoromethyl benzaldehyde (34 µL, 0.25 mmol, 1.0 equiv). The mixture was then stirred in the dark for 1 h before irradiation with blue LEDs at room temperature for 16 h. The solvent was removed *in vacuo*, and the crude product was purified without work-up. Purification by flash column chromatography (2:100 to 5:95 MeOH/DCM) gave **40** (50.8 mg, 51%, 1:1 d.r.) as a colorless oil.

R_f = 0.50 (5:95 MeOH/DCM, CAM).

NMR Spectroscopy (*see spectra*):

^{1}H NMR (400 MHz, $CDCl_3$): δ_H 7.62 (t, J = 8.4 Hz, 2H), 7.56 – 7.29 (m, 12H), 7.15 (td, J = 7.4, 3.1 Hz, 1H), 4.97 and 4.71 (dd, J = 8.6, 3.6 Hz; dd, J = 10.1, 3.2 Hz; 1H), 3.95 and 3.87 (t, J = 6.9 Hz; dd, J = 9.9, 3.9 Hz; 1H), 3.64 (br. s, 1H), 2.76 – 2.66 (m, 1H), 2.28 – 2.13 (m, 1H) ppm.

^{13}C NMR (126 MHz, $CDCl_3$): δ_C 172.45, 172.06, 148.77, 148.51, 139.42, 139.27, 137.62, 137.51, 129.85 (q, J = 32.0 Hz), 125.76 (q, J = 32.7 Hz), 129.59, 129.40, 129.14, 129.11, 128.35, 128.19, 128.05, 127.94, 126.17, 125.55 (q, J = 3.7 Hz), 124.86, 124.82, 124.29 (q, J = 271.7 Hz), 124.26 (q, J = 272.1 Hz), 120.18, 120.13, 72.41, 71.48, 51.64, 50.30, 42.90, 42.76 ppm.

^{19}F NMR (377 MHz, $CDCl_3$): δ_F -62.27, -62.31 ppm.

IR (film): v_{max} 3316, 2985, 1734, 1539, 1373, 1325, 1234, 1044 cm^{-1}.

HRMS (ESI$^+$): calc'd for $[M+H]^+$, 400.1519; found 400.1500.

2.4.3. Intramolecular Cyclization Scope

1-(3-Benzyl-4-hydroxy-3,4-dihydroquinolin-1(2*H*)-yl)ethan-1-one (41)

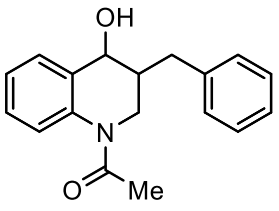

41

Prepared following **General Procedure C**, using *N*-cinnamyl-*N*-(2-formylphenyl) acetamide (**S2**) (69.8 mg). Purification by flash column chromatography (5:95 MeOH/DCM) gave **41** (31.6 mg, 45%, 1.4:1 d.r.) as a yellow oil.

R_f = 0.32 (5:95 MeOH/DCM, CAM).

NMR Spectroscopy (*see spectra*):

^{1}H NMR (400 MHz, $CDCl_3$): δ_H 7.50 – 7.08 (m, 9H), 4.56 and 4.50 (d, *J* = 2.9 Hz; d, *J* = 2.8 Hz; 1H), 4.00-3.84 and 3.81 (m; dd, *J* = 13.0, 4.6 Hz; 1H), 3.69 – 3.56 and 3.51 (m; dd, *J* = 13.0, 10.9 Hz; 1H), 2.94 and 2.87 (dd, *J* = 13.7, 8.2 Hz; dd, *J* = 13.9, 6.4 Hz; 1H), 2.74 and 2.56 (dd, *J* = 13.7, 7.3 Hz; dd, *J* = 14.0, 8.6 Hz; 1H), 2.40 – 2.20 (m, 1H), 2.18 and 2.10 (s and s; 3H) ppm.

^{13}C NMR (101 MHz, $CDCl_3$): δ_C 170.48, 170.36, 139.32, 138.96, 137.88, 129.18, 129.12, 128.85, 128.77, 128.57, 128.08, 126.69, 126.55, 125.30, 124.91, 124.44, 123.99, 70.60, 67.89, 45.84, 44.89, 44.10, 42.59, 36.87, 35.37, 23.79, 23.48 ppm.

IR (film): ν_{max} 3384, 3025, 2923, 2853, 1634, 1489, 1388, 1215, 1031 cm^{-1}.

HRMS (ESI$^+$): calc'd for $[M+H]^+$, 282.1489; found 282.1479.

1-(4-Hydroxy-3-(4-(methylsulfonyl) benzyl)-3,4-dihydroquinolin-1(2*H*)-yl)ethan-1-one (42)

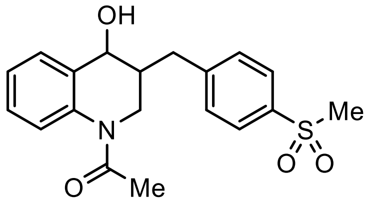

42

Prepared following **General Procedure C**, using (*E*)-*N*-(2-formylphenyl)-*N*-(3-(4-(methylsulfonyl)phenyl)allyl) acetamide (**S6**) (89.4 mg). Purification by flash column chromatography (5:95 MeOH/DCM) gave **42** (42.4 mg, 47%, 1.4:1 d.r.) as a pale-yellow solid.

R_f = 0.27 (5:95 MeOH/DCM, CAM).

NMR Spectroscopy (see spectra):

^{1}H NMR (400 MHz, $CDCl_3$): δ_H 7.88 and 7.87 (m, 2H), 7.48 – 7.39 (m, 3H), 7.32 – 7.11 (m, 3H), 4.48 and 4.46 (d, *J* = 5.6 Hz; d, *J* = 8.9 Hz; 1H), 4.04 – 3.96 and 3.78 (m; dd, *J* = 13.0, 5.0 Hz, 1H), 3.68 and 3.51 (dd, *J* = 13.0, 7.1 Hz, dd, *J* = 12.6, 10.7 Hz, 1H), 3.08 - 2.96 (m, 1H), 3.05 and 3.04 (s; s; 3H), 2.81 and 2.66 (dd, *J* = 13.6, 6.8 Hz; dd, *J* = 14.0, 8.6 Hz; 1H), 2.38 -2.19 (m, 1H), 2.35 (br. s, 1H), 2.22 and 2.17 (s; s; 3H) ppm.

^{13}C NMR (101 MHz, $CDCl_3$): δ_C 170.48, 170.31, 146.15, 145.83, 138.94, 138.81, 137.76, 130.28, 130.14, 128.78, 128.25, 127.89, 127.82, 125.63, 125.22, 124.44, 124.05, 70.33, 67.40, 45.43, 44.68, 44.19, 42.39, 36.94, 35.35, 23.74, 23.38 ppm.

IR (film): ν_{max} 3397, 3010, 2924, 1647, 1490, 1389, 1300, 1148, 1089 cm^{-1}.

HRMS (ESI^{+}): calc'd for $[M+H]^{+}$,360.1264; found 360.1252.

(1*S,2*R**)-2-Benzyl-2,3-dihydro-1*H*-inden-1-ol (43)**

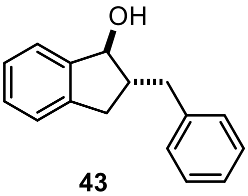

43

Prepared following **General Procedure C**, using 2-cinnamylbenzaldehyde (55.6 mg). ^{1}H NMR analysis of the crude product gave an assay yield of 24% (10:1 *trans*/*cis*). Purification by flash column chromatography (10:90 to 20:80 EtOAc/petroleum ether) followed by preparative TLC (10:90 EtOAc/petroleum ether) gave **43** (12.2 mg, 22%, >20:1 *trans*/*cis*) as a white solid.

R_f = 0.46 (20:80 EtOAc/petroleum ether, CAM).

NMR Spectroscopy (see spectra):

^{1}H NMR (400 MHz, $CDCl_3$): δ_H 7.40 – 7.29 (m, 3H), 7.31 – 7.21 (m, 5H), 7.20 – 7.15 (m, 1H), 4.95 (d, *J* = 6.4 Hz, 1H), 3.10 (dd, *J* = 13.5, 6.0 Hz, 1H), 3.01 (dd, *J* = 14.9, 6.9 Hz, 1H), 2.79 (dd, *J* = 13.5, 8.5 Hz, 1H), 2.61 – 2.48 (m, 2H) ppm.

^{13}C NMR (101 MHz, $CDCl_3$): δ_C 144.63, 141.68, 140.74, 129.05, 128.71, 128.33, 126.93, 126.33, 124.94, 124.08, 80.99, 52.44, 39.44, 35.92 ppm.

HRMS (EI): calc'd for $[M]^{+}$, 224.1196; found 224.1191.

All recorded spectroscopic data matched those previously reported in the literature.[29]

(1*S,2*R**)-2-(4-(Methylsulfonyl)benzyl)-2,3-dihydro-1*H*-inden-1-ol (44)**

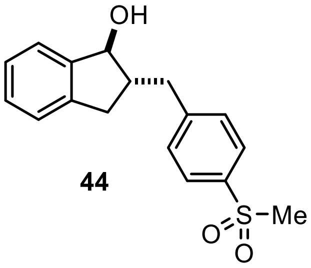

44

Prepared following **General Procedure C**, using (*E*)-2-(3-(4-(methylsulfonyl)phenyl)allyl)benzaldehyde (75.1 mg). Purification by flash column chromatography (5:95 MeOH/DCM) gave **44** (37.7 mg, 50%, 7:1 *trans/cis*) as a white solid.

R$_f$ = 0.32 (5:95 MeOH/DCM, CAM).

NMR Spectroscopy (*see spectra*):

> **^{1}H NMR** (400 MHz, $CDCl_3$): δ_H 7.88 (dq, *J* = 8.4, 2.0 Hz, 2H), 7.54 – 7.49 and 7.48 – 7.42 (m; m; 2H), 7.41 – 7.35 (m, 1H), 7.31 – 7.13 (m, 3H), 4.96 and 4.93 (d, *J* = 5.5 Hz; d, *J* = 5.2 Hz; 1H), 3.28 – 3.17 (m, 1H), 3.06 (s, 3H), 3.02 – 2.92 (m, 1H), 2.91 – 2.77 (m, 1H), 2.70 – 2.46 (m, 2H) ppm.

> **^{13}C NMR** (101 MHz, $CDCl_3$): δ_C (*chemical shifts in italics correspond to the minor diastereomer*) *148.34*, 147.49, *144.63*, 144.33, *143.17*, 141.21, 138.57, *138.32*, *130.09*, 130.00, *129.05*, 128.57, 127.77, *127.65*, 127.15, *127.12*, *125.22*, 125.01, *124.84*, 124.08, 80.85, *76.05*, 51.88, *46.89*, 44.70, 39.22, *35.99*, 35.63, *35.18* ppm.

IR (film): ν_{max} 3049, 3023, 2925, 2850, 1300, 1147, 1089 cm^{-1}.

HRMS (ESI$^+$): calc'd for $[M+Na]^+$, 325.0869 ; found 325.0864.

3-Benzylquinoline (46)

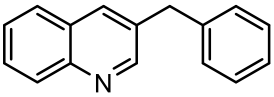

46

Prepared following **General Procedure C**, using 2-(cinnamylamino)benzaldehyde (59.3 mg). Purification by flash column chromatography (20:80 EtOAc/petroleum ether) gave **46** (29.3 mg, 53%) as a yellow oil.

R$_f$ = 0.30 (20:80 EtOAc/petroleum ether, CAM).

NMR Spectroscopy (*see spectra*):

> **^{1}H NMR** (400 MHz, $CDCl_3$): δ_H 8.83 (d, *J* = 2.2 Hz, 1H), 8.10 (d, *J* = 8.5 Hz, 1H), 7.89 (d, *J* = 2.2 Hz, 1H), 7.75 (dd, *J* = 8.1, 1.4 Hz, 1H), 7.68 (ddd, *J* = 8.4, 6.8, 1.5 Hz, 1H), 7.53 (ddd, *J* = 8.2, 6.8, 1.2 Hz, 1H), 7.41 – 7.28 (m, 2H), 7.26 (td, *J* = 6.6, 2.0 Hz, 3H), 4.18 (s, 2H) ppm.

^{13}C NMR (101 MHz, $CDCl_3$): δ_C 152.26, 147.03, 139.81, 134.96, 133.96, 129.32, 129.09, 128.97, 128.87, 128.24, 127.57, 126.79, 126.69, 39.36 ppm.

HRMS (EI$^+$): calc'd for $[M-H]^+$, 218.0964; found 218.0959.

All recorded spectroscopic data matched those previously reported in the literature.[30]

2.4.4. Scale-up Reaction (2.0 mmol)

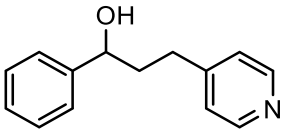

6a

To a round bottom flask was added NaOH (640 mg, 16 mmol, 8.0 equiv) and H_2O (32 mL). After dissolution of NaOH, MeCN (8.0 mL), eosin Y (26.0 mg, 0.04 mmol, 2.0 mmol%) and TDO (648 mg, 6.0 mmol, 3.0 equiv) were added to the flask. The solution was degassed by N_2 for 5 min before addition of benzaldehyde (212 mg, 2.0 mmol, 1.0 equiv) and 4-vinylpyridine (640 µL, 6.0 mmol, 3.0 equiv). The solution was further degassed by N_2 for 1 min, stirred at dark for 1 h before irradiation with two Kessi Tuna Blue lamps for 16 h at room temperature.

The solvent was removed under vacuum with added MeOH at 50 °C. The crude was dissolved in 20% MeOH/DCM, neutralized with acetic acid (680 µL, 6.0 equiv) and passed through silica with 20% MeOH/DCM (600 mL). The solution was concentrated in vacuo and the resulting crude was purified by silica gel column chromatography (5:95 MeOH/DCM) to afford the **6a** as a white solid (72%, 305.8 mg).

R_f = 0.32 (5:95 MeOH/DCM, CAM).

See small scale procedure for NMR data**.**

2.4.5. Unsuccessful and Low Yielding Substrates

No product or low yields were also observed for the following substrates. Yields were determined by ^{1}H NMR analysis using an internal standard.

Carbonyls (reaction with 4-vinylpyridine):

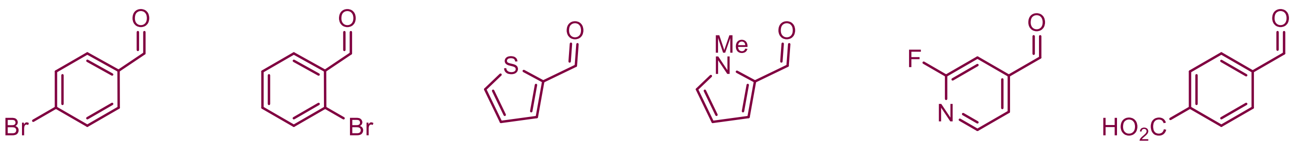

0% 0% 20% <40% 0% <10%

debromination observed *difficult to purify*

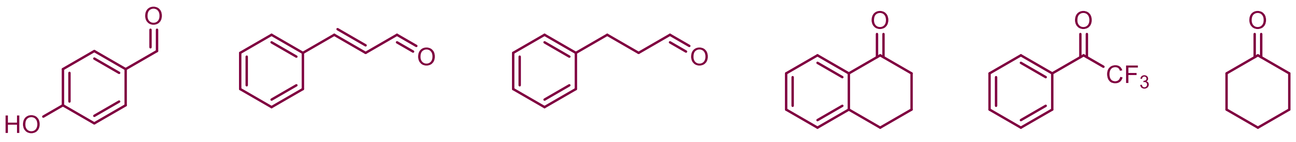

0% 0% 0% 0% 0% 0%

Olefins (reaction with benzaldehyde):

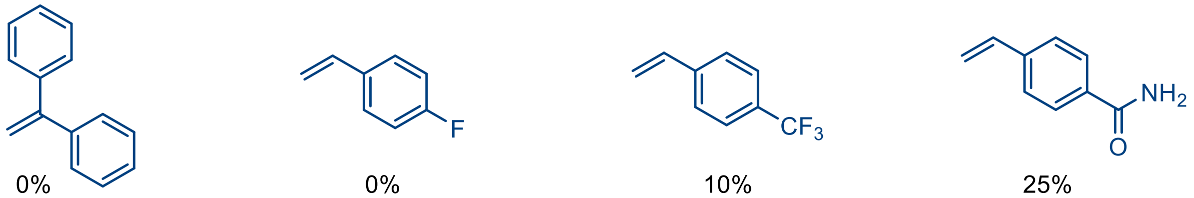

0% 0% 10% 25%

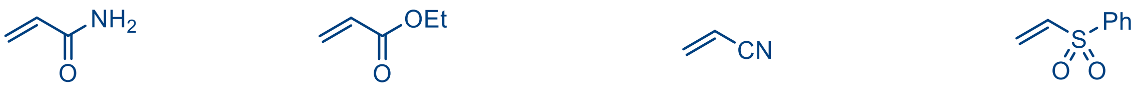

19% 0% 32% 0%

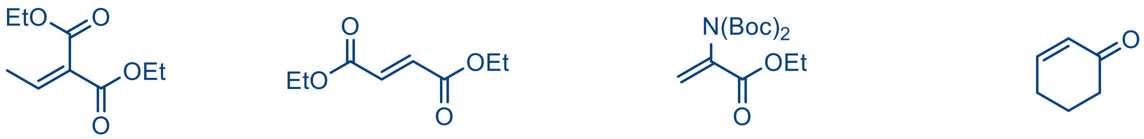

0% 0% 0% 0%

Intramolecular couplings:

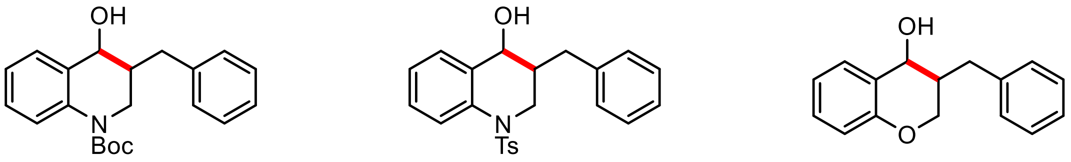

<20% 7% 20%

3. MECHANISTIC STUDIES

3.1. Cyclic Voltammetry

All measurements were conducted under air in a H_2O/MeCN (4:1) solution with 0.1 M $LiClO_4$ supporting electrolyte at 100 mV/s. The analyte was measured in a 20 mM concentration. 1 equivalent of ferrocene (Fc) was added and the Fc/Fc^+ redox couple was set to 0 V. Fc/Fc^+ was then converted to saturated calomel electrode (SCE) using the conversion: $E_{1/2}$ (Fc/Fc^+) = +0.38 V vs. SCE.[31]

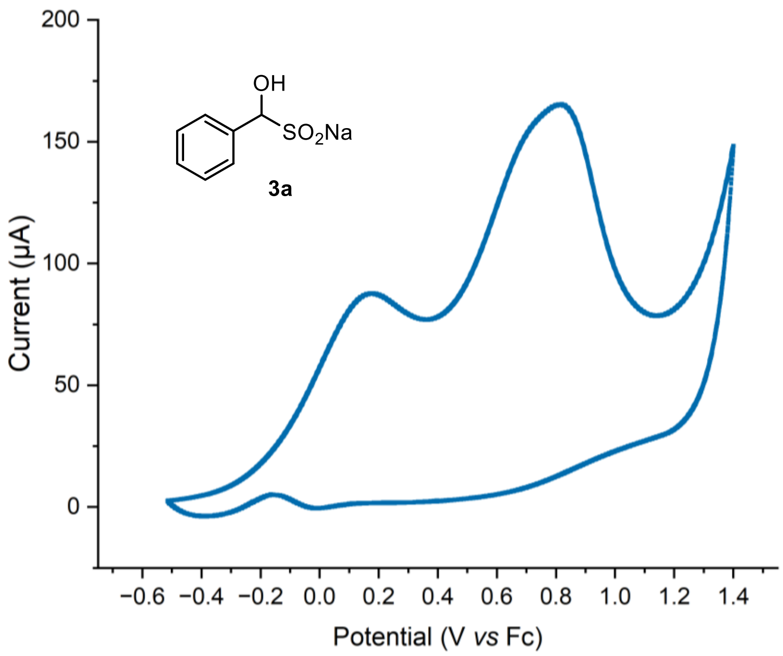

Figure S3. Cyclic voltammogram of α-hydroxy sulfinate 3a

Redox potentials:

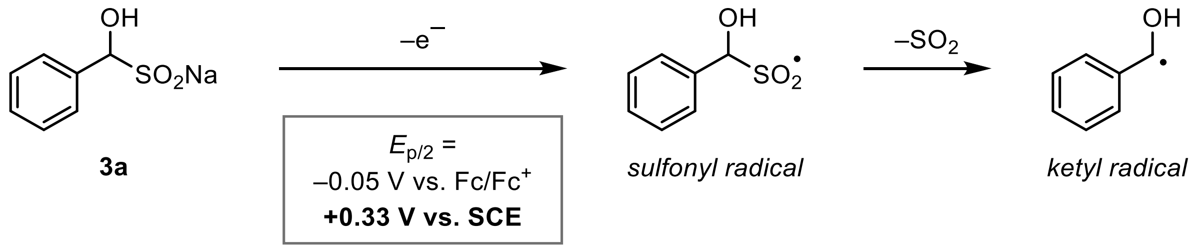

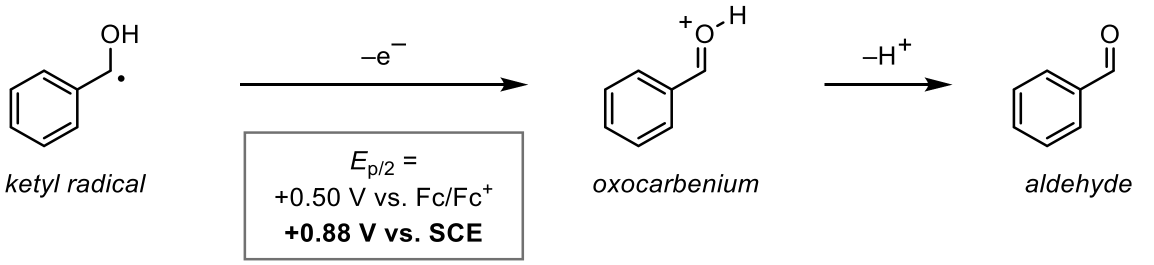

3.2. Fluorescence Quenching Studies

3.2.1. Quenching Studies with $Ru(bpy)_3Cl_2$

Stern-Volmer fluorescence quenching experiments were run with freshly prepared solutions of $Ru(bpy)_3Cl_2$ (2 µM, 1:4 MeCN/H_2O). After degassing by sparging with N_2, solutions were irradiated at 450 nm and fluorescence was measured from 470 nm to 800 nm. The emission intensities at the maximum emission wavelength (λ = 600 nm) were recorded for different concentrations of quencher and were used to draw the Stern-Volmer plots.

<u>Isolated α-hydroxy sulfinate</u>

No quenching was observed for the isolated α-hydroxy sulfinate **3a** *without* NaOH (**Figure S4**).

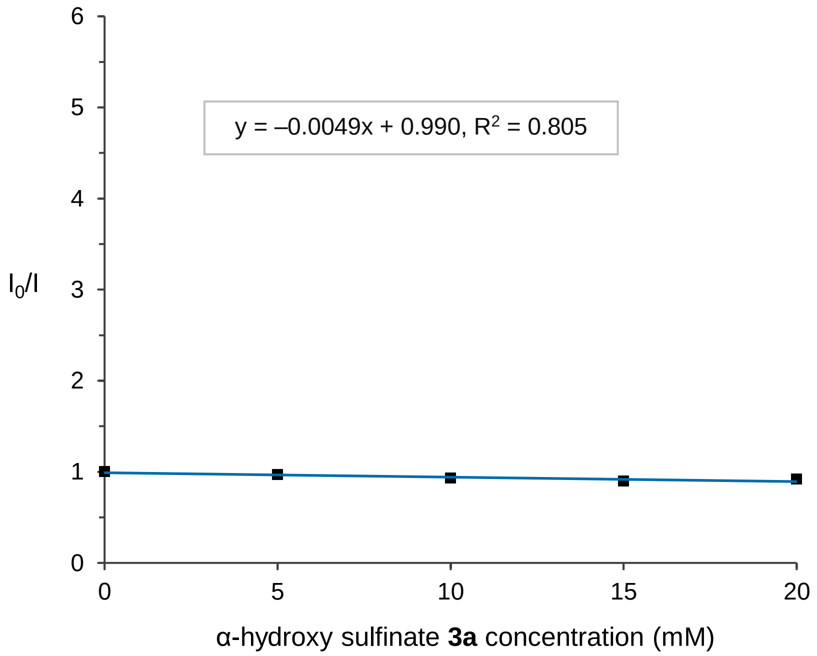

Figure S4. Stern-Volmer plot of fluorescence quenching of $Ru(bpy)_3Cl_2$ by isolated α-hydroxy sulfinate 3a without NaOH

Quenching was observed with α-hydroxy sulfinate **3a** *with* addition of 1-5 equivalents of NaOH (**Figure S5**). Higher quenching efficiency was observed with higher equivalents of NaOH.

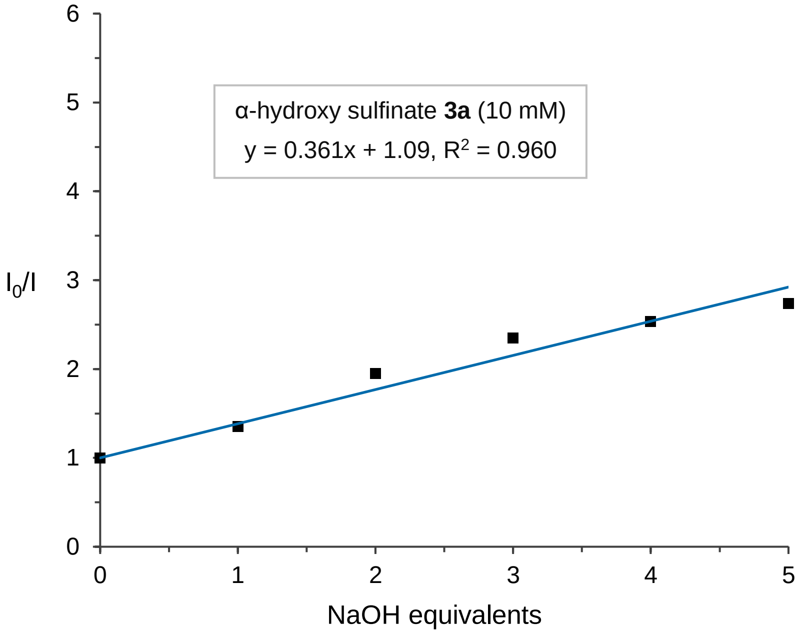

Figure S5. Stern-Volmer plot of fluorescence quenching of $Ru(bpy)_3Cl_2$ by α-hydroxy sulfinate 3a (10 mM) with increasing equivalents of NaOH

With the addition of NaOH (50 mM), increased quenching efficiency was observed with increasing concentration of isolated α-hydroxy sulfinate **3a** (**Figure S6**). The quencher rate coefficient was determined to be:

K_{SV} = 0.19

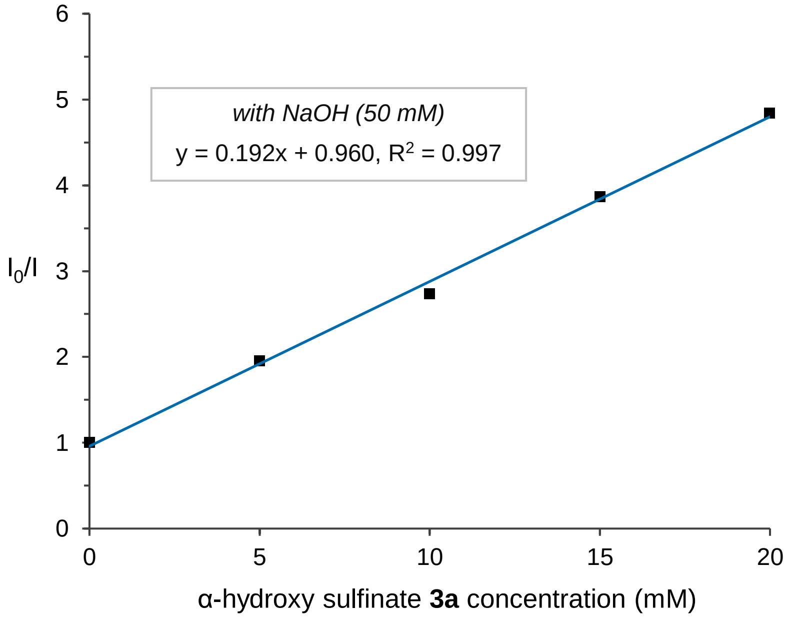

Figure S6. Stern-Volmer plot of fluorescence quenching of $Ru(bpy)_3Cl_2$ by α-hydroxy sulfinate 3a with NaOH (50 mM)

For the *in situ* generated α-hydroxy sulfinate, for each concentration respectively, benzaldehyde (5-20 mM), TDO (1 equiv), and NaOH (1 equiv + 50 mM) were added into prepared solutions of Ru(bpy)$_3$Cl$_2$. The solutions were stirred for 2 h before the fluorescence quenching experiments were carried out (**Figure S7**). The quencher rate coefficient was determined to be:

$$K_{SV} = 0.18$$

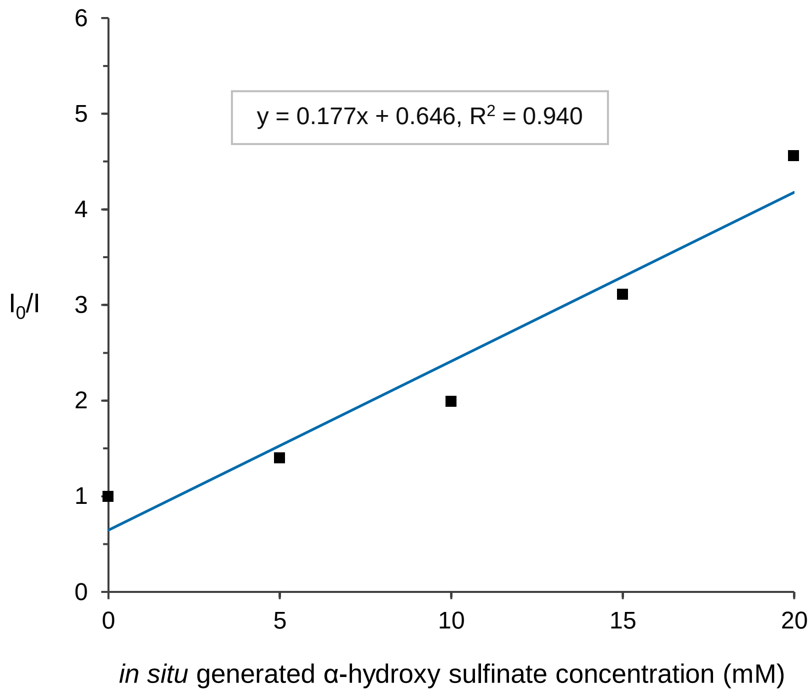

Figure S7. Stern-Volmer plot of fluorescence quenching of Ru(bpy)$_3$Cl$_2$ by *in situ* generated α-hydroxy sulfinate with NaOH (50 mM)

Benzaldehyde

No quenching was observed for benzaldehyde (5-20 mM) (**Figure S8**).

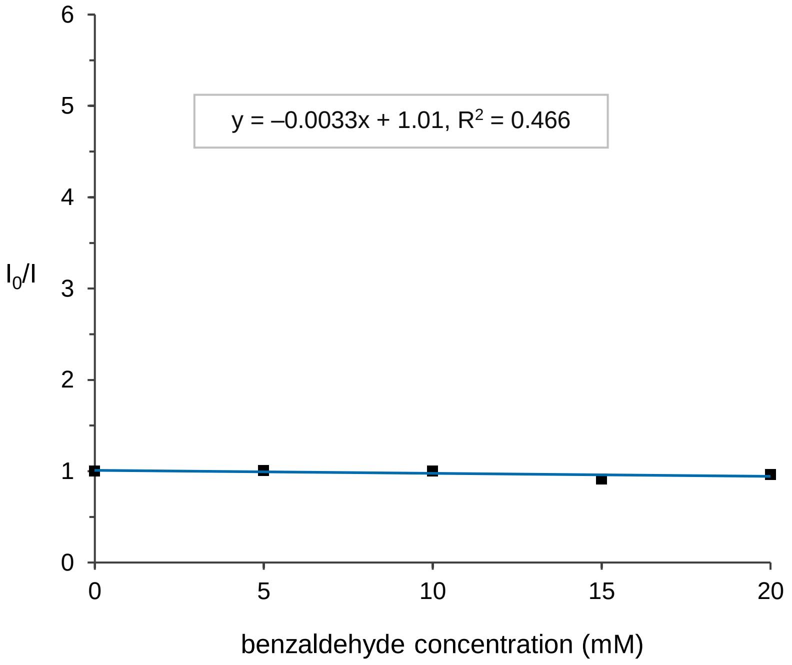

Figure S8. Stern-Volmer plot of fluorescence quenching of $Ru(bpy)_3Cl_2$ by benzaldehyde

4-Vinylpyridine

No quenching was observed for 4-vinylpyridine (5-20 mM) (**Figure S9**)

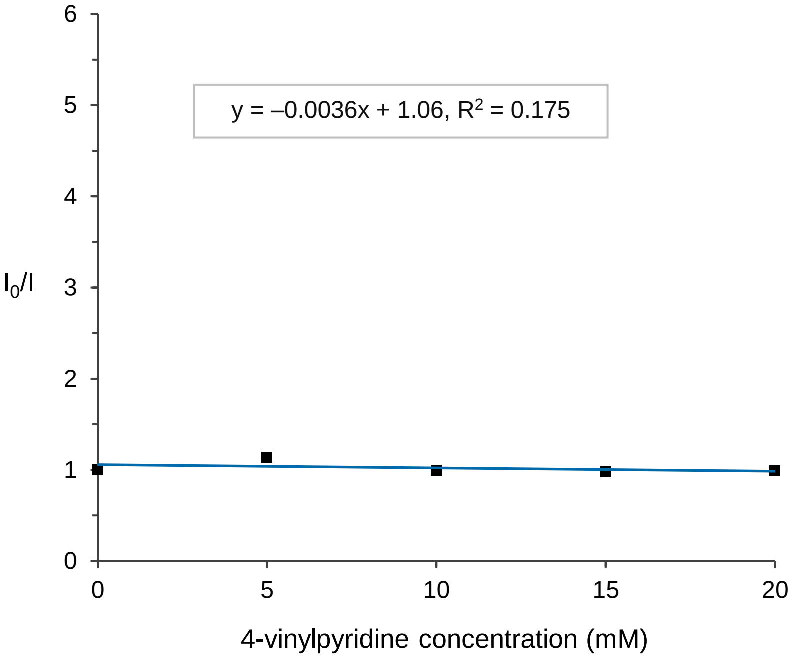

Figure S9. Stern-Volmer plot of fluorescence quenching of $Ru(bpy)_3Cl_2$ by 4-vinylpyridine

Thiourea dioxide (TDO)

No quenching was observed for TDO (5-20 mM) with 2 equiv NaOH (**Figure S10**).

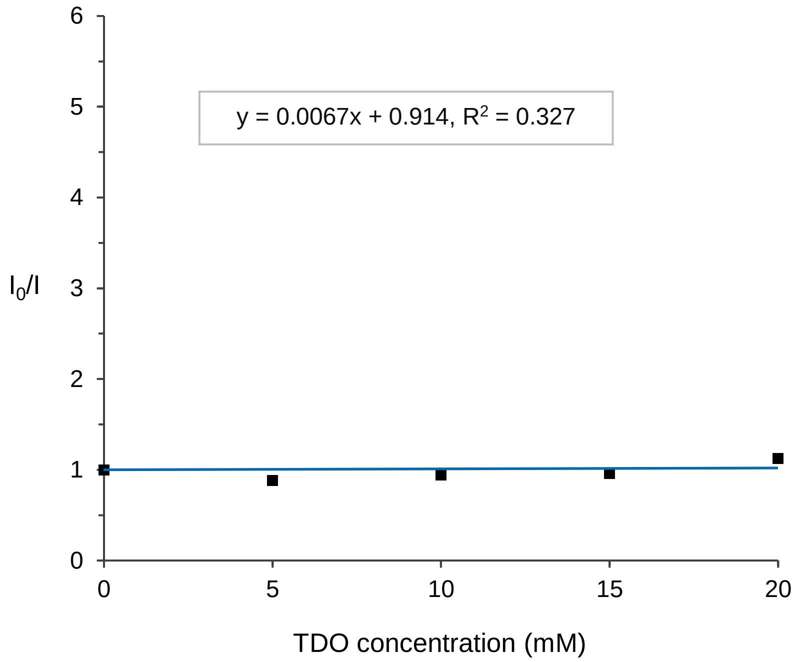

Figure S10. Stern-Volmer plot of fluorescence quenching of $Ru(bpy)_3Cl_2$ by TDO with NaOH (equiv NaOH)

No quenching was observed for TDO (10 mM) with NaOH (2-5 equiv) (**Figure S11**).

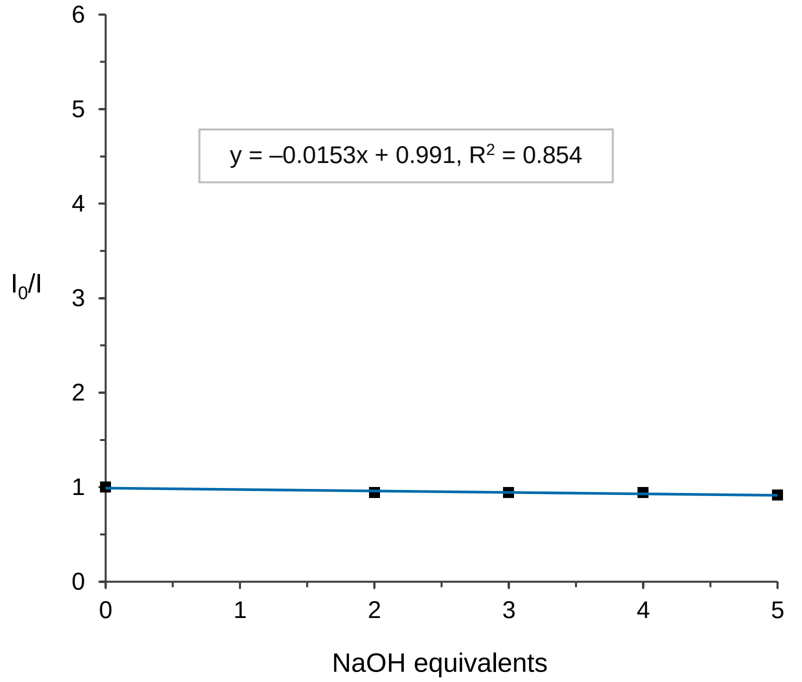

Figure S11. Stern-Volmer plot of fluorescence quenching of $Ru(bpy)_3Cl_2$ by TDO (10 mM) with increasing equivalents of NaOH

3.2.2. Quenching Studies with Na_2EY

Stern-Volmer fluorescence quenching experiments were run with freshly prepared solutions of disodium eosin Y (Na_2EY, 1 µM, 1:4 MeCN/H_2O). After degassing by sparging with N_2, the solutions were irradiated at 480 nm and fluorescence was measured from 500 nm to 800 nm. The emission intensities at the maximum emission wavelength (λ = 540 nm) were recorded for different concentrations of quencher and were used to draw the Stern-Volmer plots.

<u>Isolated α-hydroxy sulfinate</u>

Initial quenching experiments with Na_2EY and α-hydroxy sulfinate **3a** appeared to show effective quenching of the excited state photocatalyst by **3a** (**Figure S12**). However, subsequent UV/vis studies revealed a ground state interaction between Na_2EY and **3a** (**Figure S13**). Therefore, although apparent 'quenching' of excited-state Na_2EY was observed at various concentrations of **3a**, the results are inconclusive due to the ground-state interaction. The ground state interaction is likely to be a reversible sulfoxylate transfer reaction (**Scheme S3**), since a similar reaction was also observed when Na_2EY was treated with TDO and NaOH (**Scheme S4**).

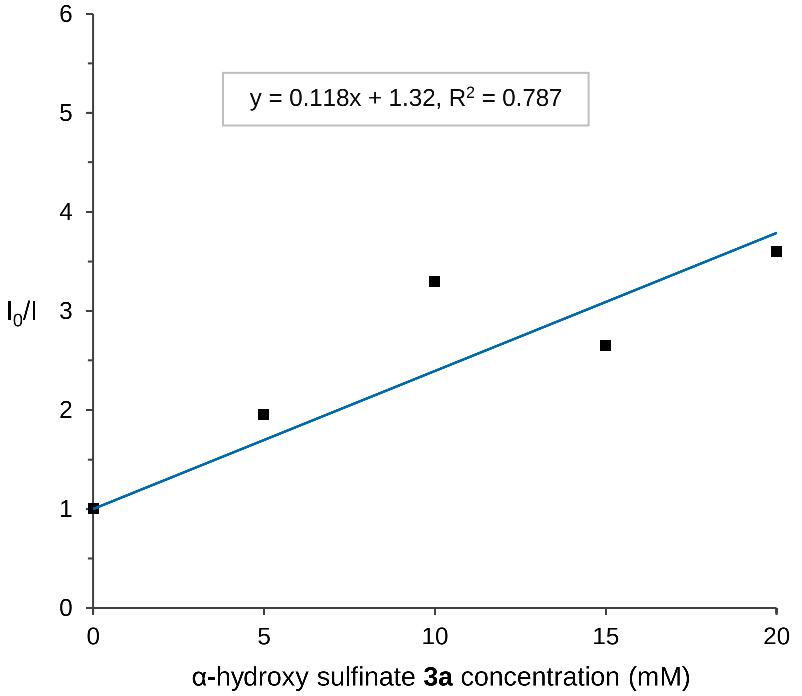

Figure S12. Stern-Volmer plot of fluorescence quenching of Na_2EY by α-hydroxy sulfinate 3a

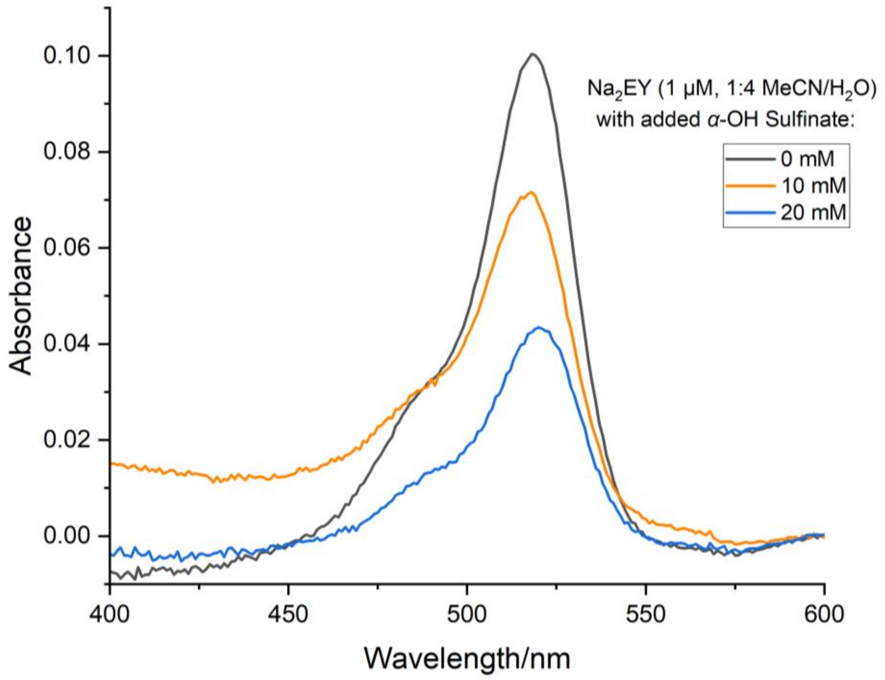

Figure S13. UV-Vis absorption spectra of Na_2EY with added α-hydroxy sulfinate 3a

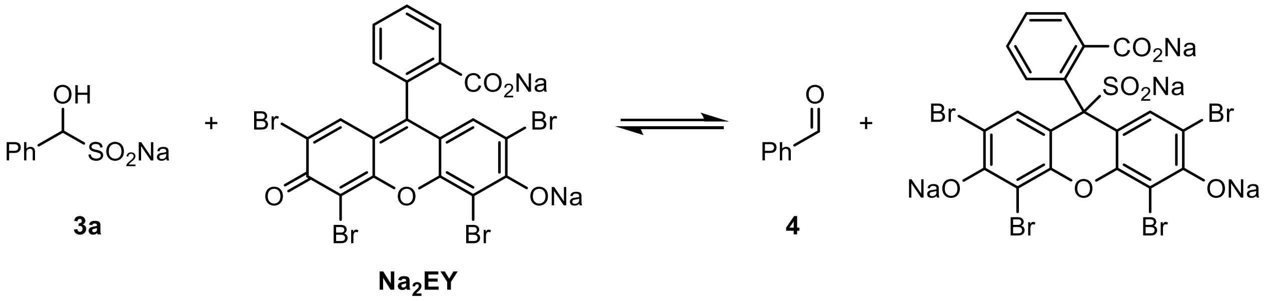

Scheme S3. Sulfoxylate transfer reaction between Na_2EY and 3a

Thiourea dioxide (TDO)

No fluorescence was observed with TDO (20 mM) and NaOH (3.0 equiv). However, this was found to be caused by the nucleophilic addition of Na_2SO_2 to ground-state Na_2EY instead of quenching of the excited state catalyst (**Scheme S4**). Confirmation of this ground state reactivity was provided by ^1H NMR studies (**Figure S14**).

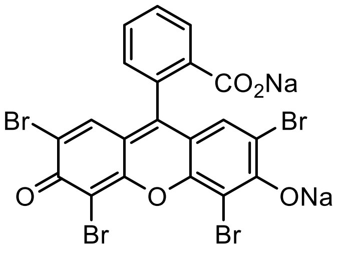

Scheme S4. Addition of Na_2SO_2 to Na_2EY

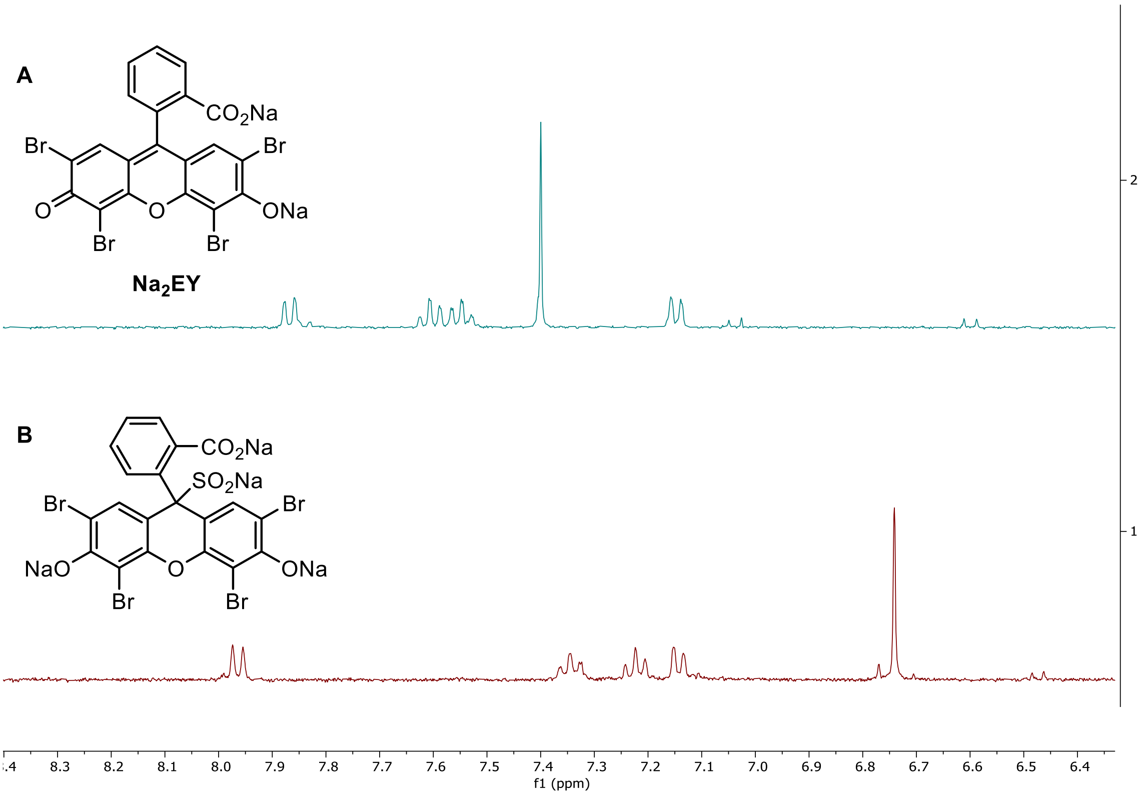

Figure S14. ^1H NMR spectra of Na_2EY with and without Na_2SO_2

(A) Eosin Y (1.3 mg, 2.0 µmol, 1.0 equiv, 2 mM), NaOH (160 equiv), D_2O/d^3-MeCN (4:1, 1 mL).
(B) Eosin Y (1.3 mg, 2.0 µmol, 1.0 equiv, 2 mM), NaOH (160 equiv), TDO (4.3 mg, 40 µmol, 20 equiv), D_2O/MeCN-d_3 (4:1, 1 mL).

Benzaldehyde

No quenching was observed for benzaldehyde (5-20 mM) (**Figure S15**).

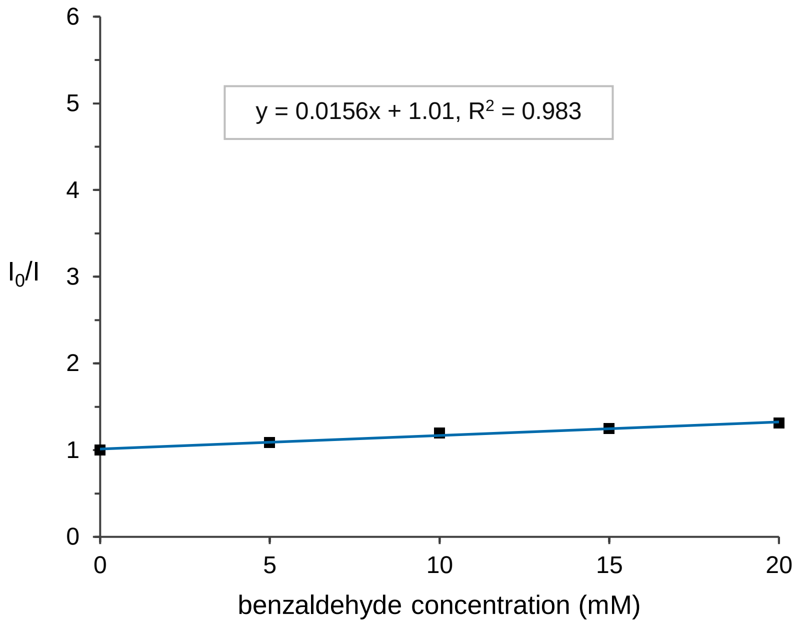

Figure S15. Stern-Volmer plot of fluorescence quenching of Na_2EY by benzaldehyde

4-VinylPyridine

No quenching was observed for 4-vinylpyridine (5-20 mM) (**Figure S16**).

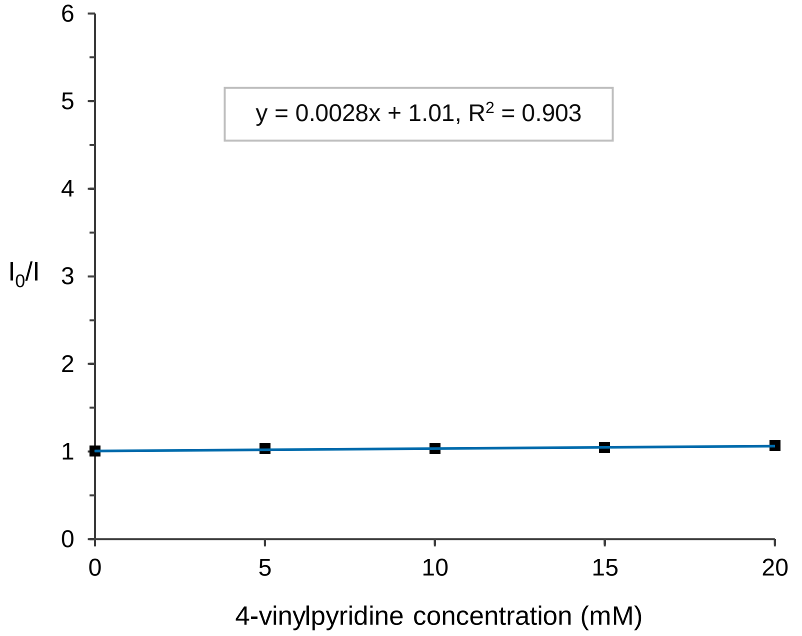

Figure S16. Stern-Volmer plot of fluorescence quenching of Na_2EY by 4-vinylpyridine

4. SPECTROSCOPIC DATA

^{1}H NMR (400 MHz, D_2O) of **3a** (*see procedure*)

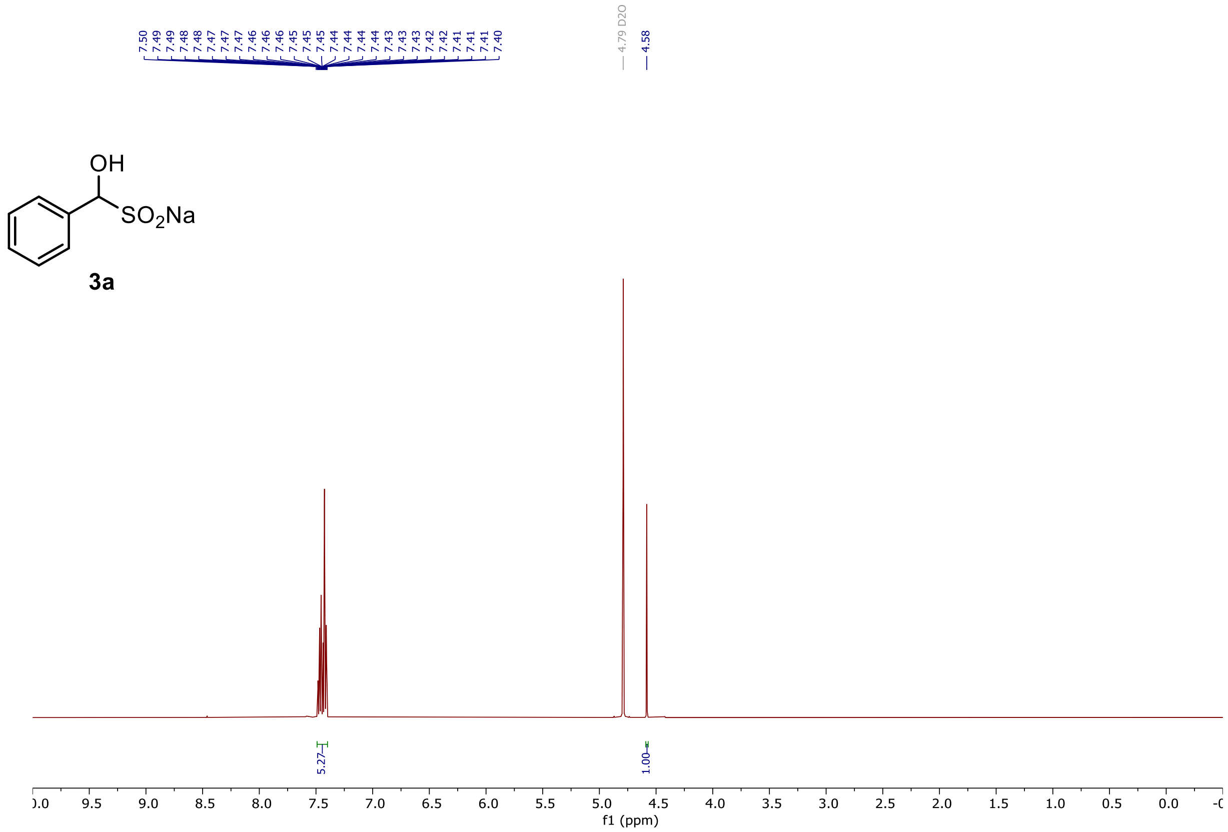

^{13}C NMR (101 MHz, D_2O) of **3a**

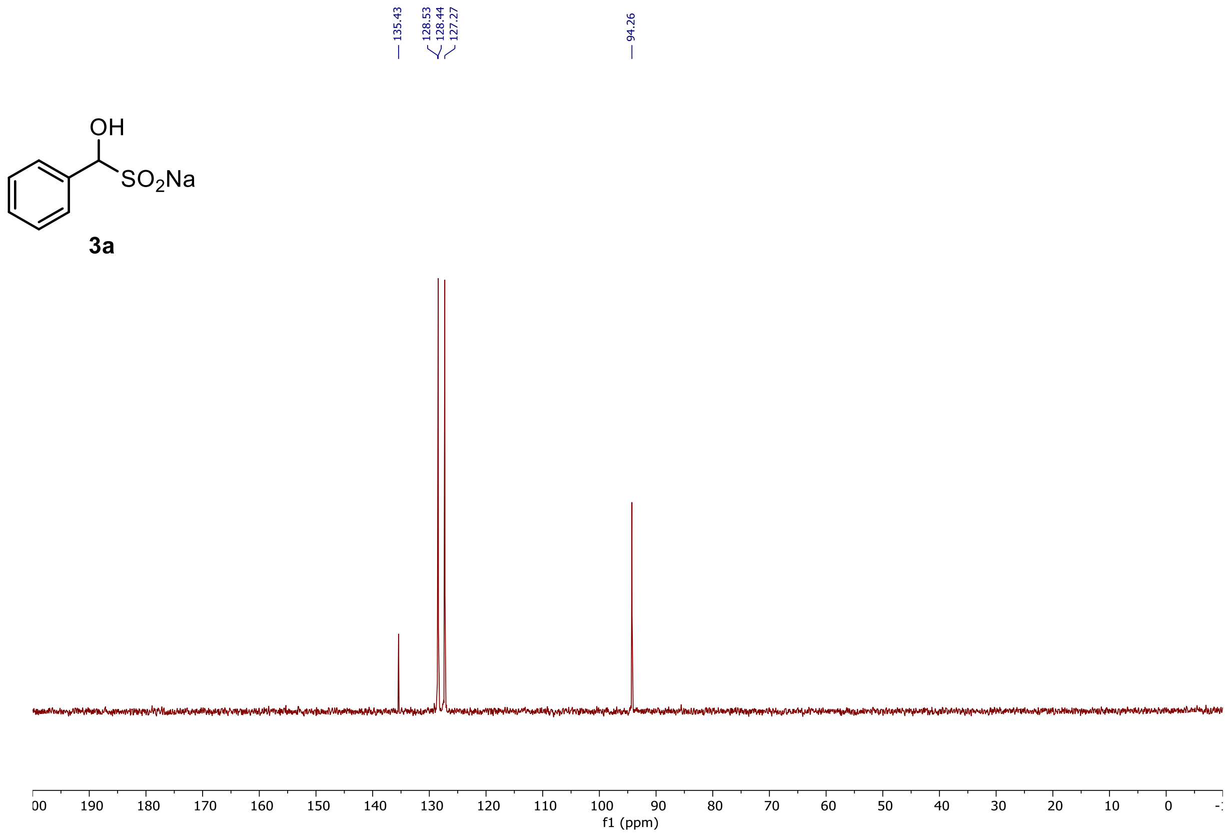

^{1}H NMR (400 MHz, $CDCl_3$) of *N*-(2-formylphenyl) acetamide (see procedure)

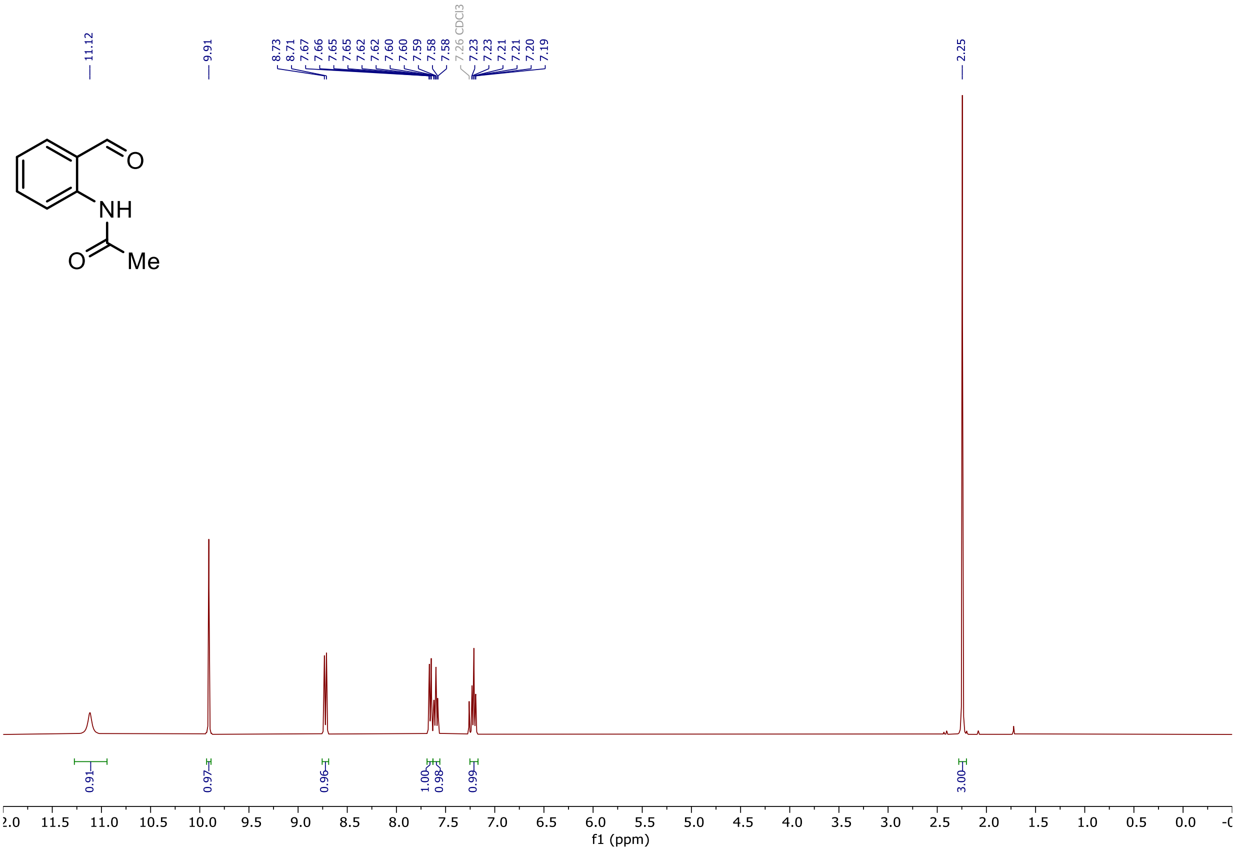

^{13}C NMR (101 MHz, $CDCl_3$) of *N*-(2-formylphenyl) acetamide

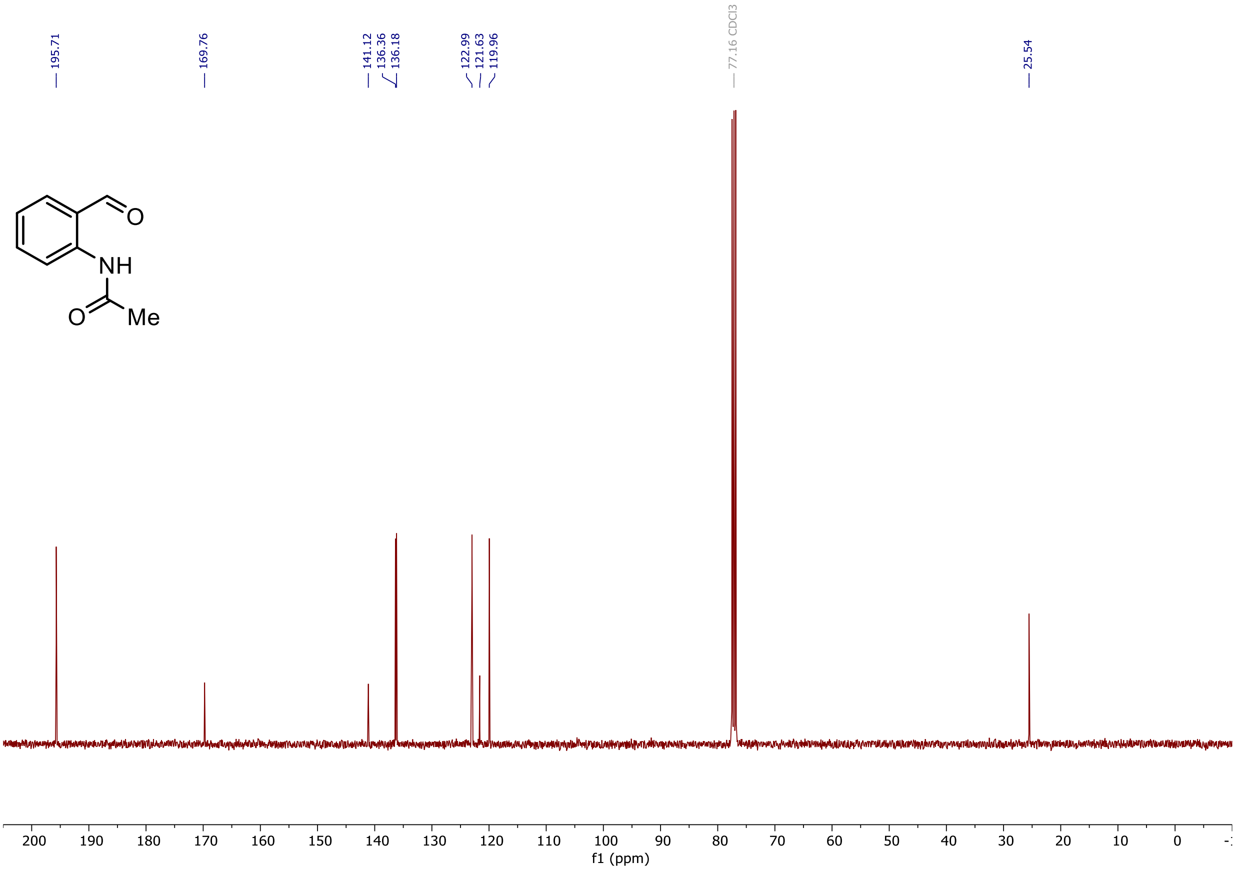

^{1}H NMR (400 MHz, $CDCl_3$) of 2-fluoro-4-vinylpyridine (see procedure)

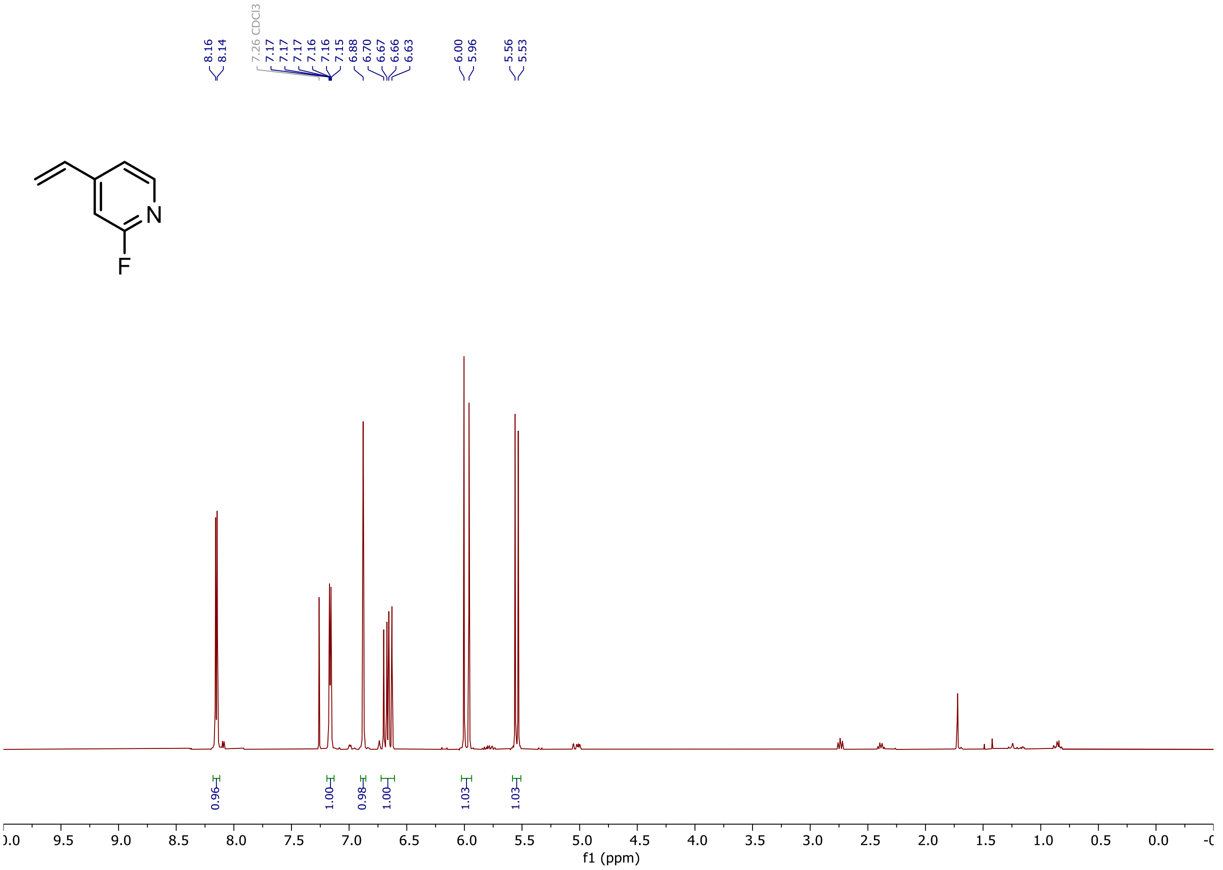

^{13}C NMR (101 MHz, $CDCl_3$) of 2-fluoro-4-vinylpyridine

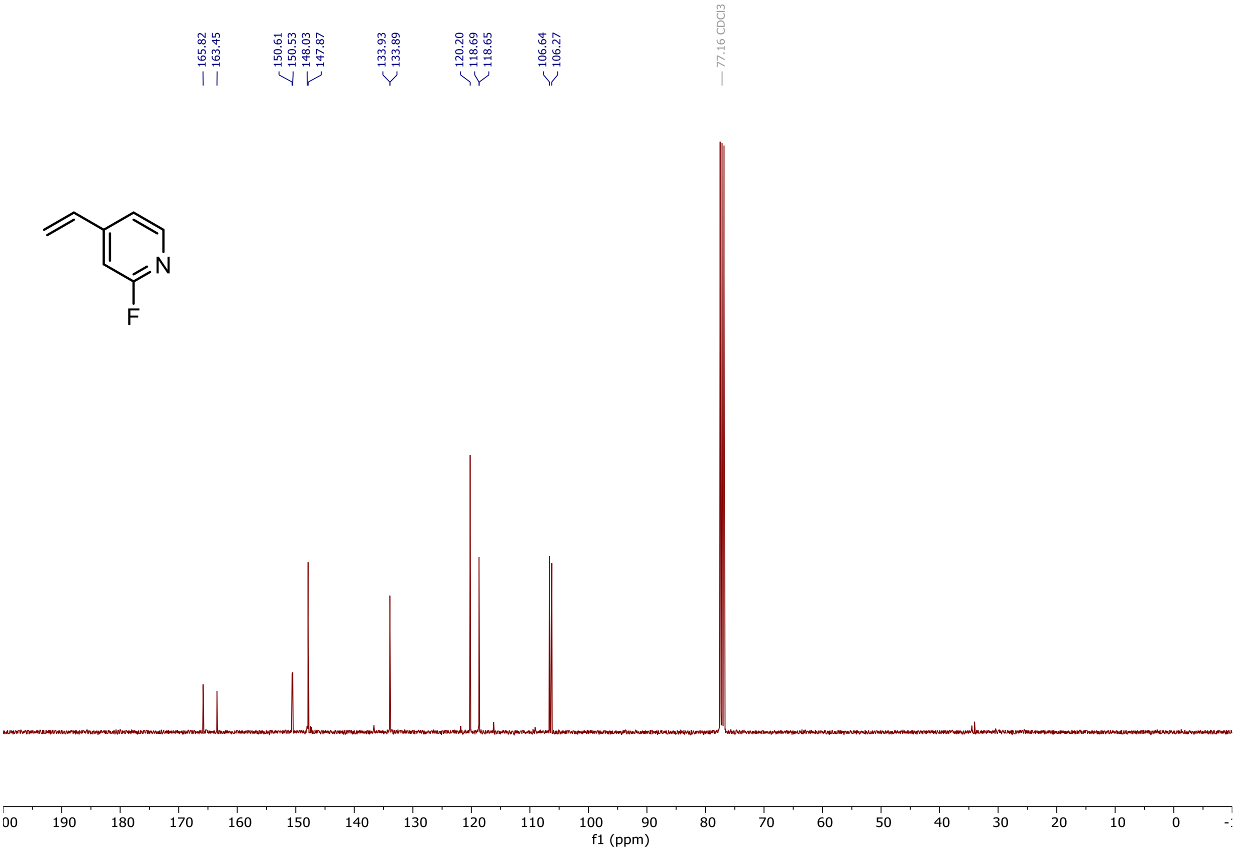

^{19}F NMR (377 MHz, $CDCl_3$) of 2-fluoro-4-vinylpyridine

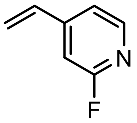

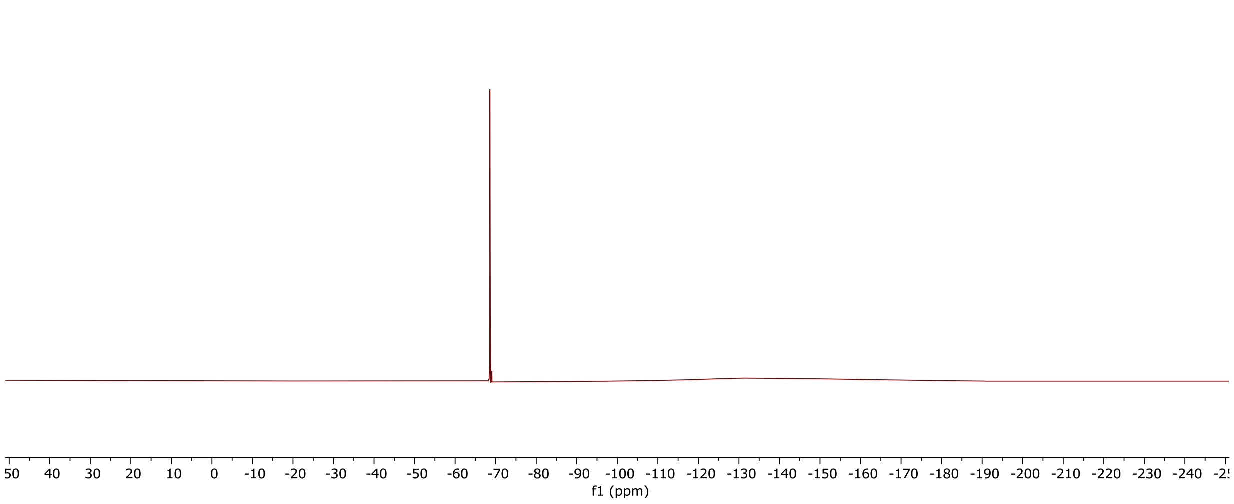

^{1}H NMR (400 MHz, $CDCl_3$) of 2-chloro-4-vinylpyridine (see procedure)

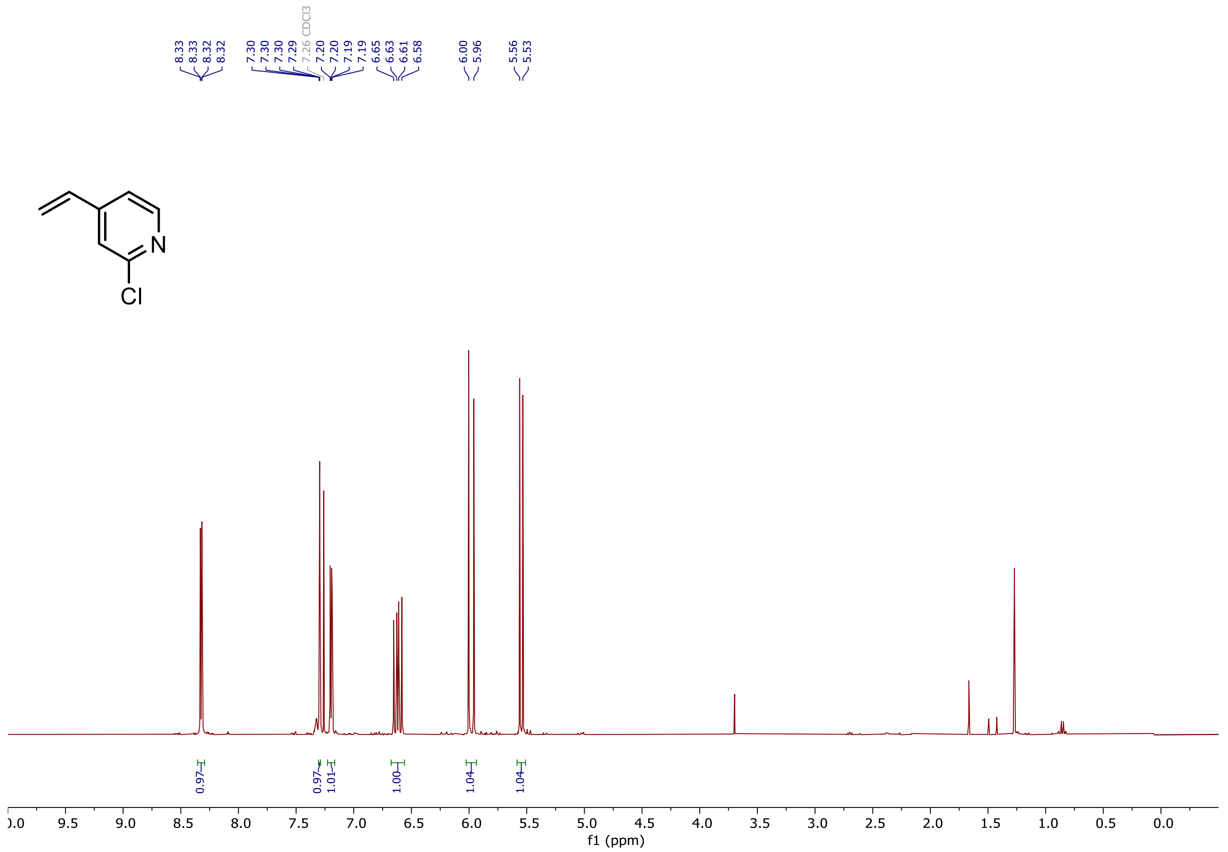

^{13}C NMR (101 MHz, $CDCl_3$) of 2-chloro-4-vinylpyridine

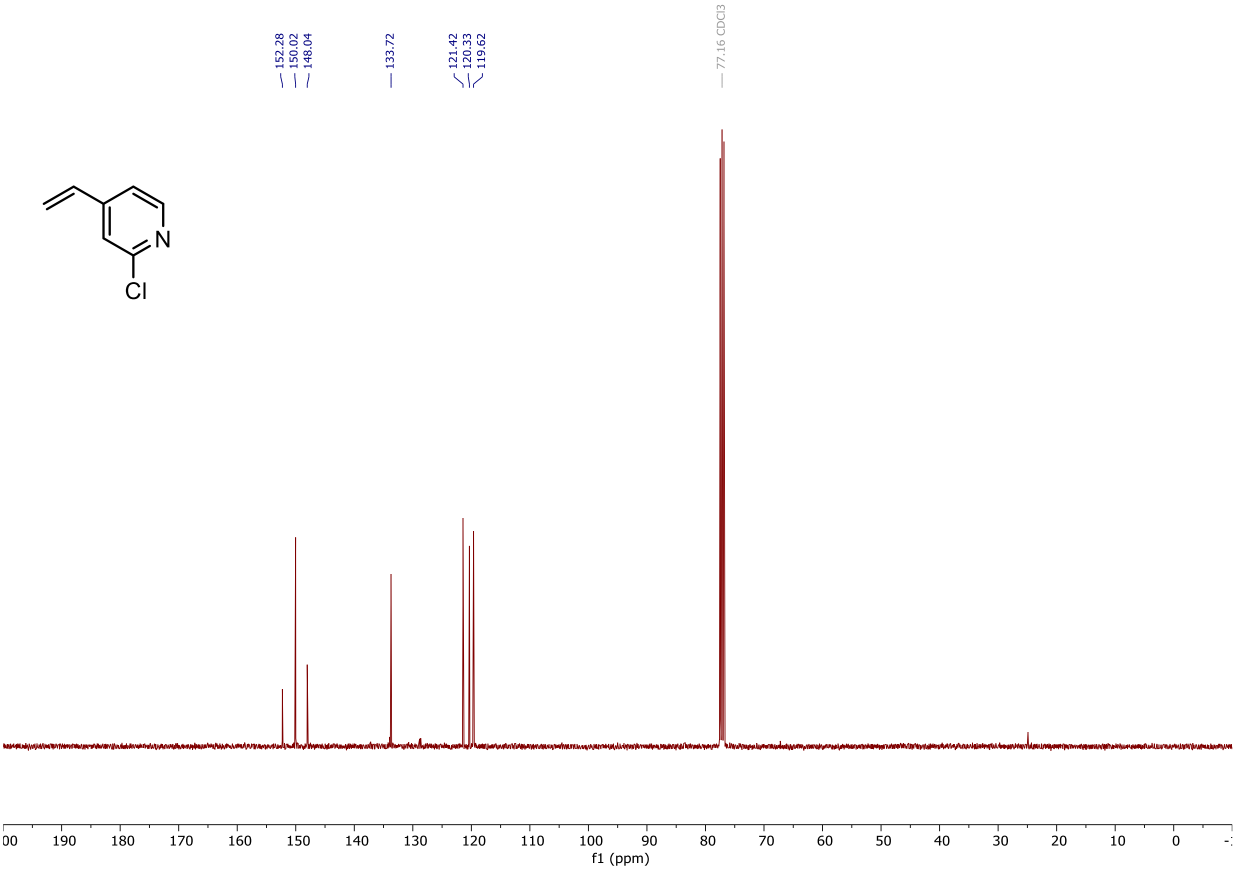

^{1}H NMR (400 MHz, $CDCl_3$) of 2-(trifluoromethyl)-4-vinylpyridine (see procedure)

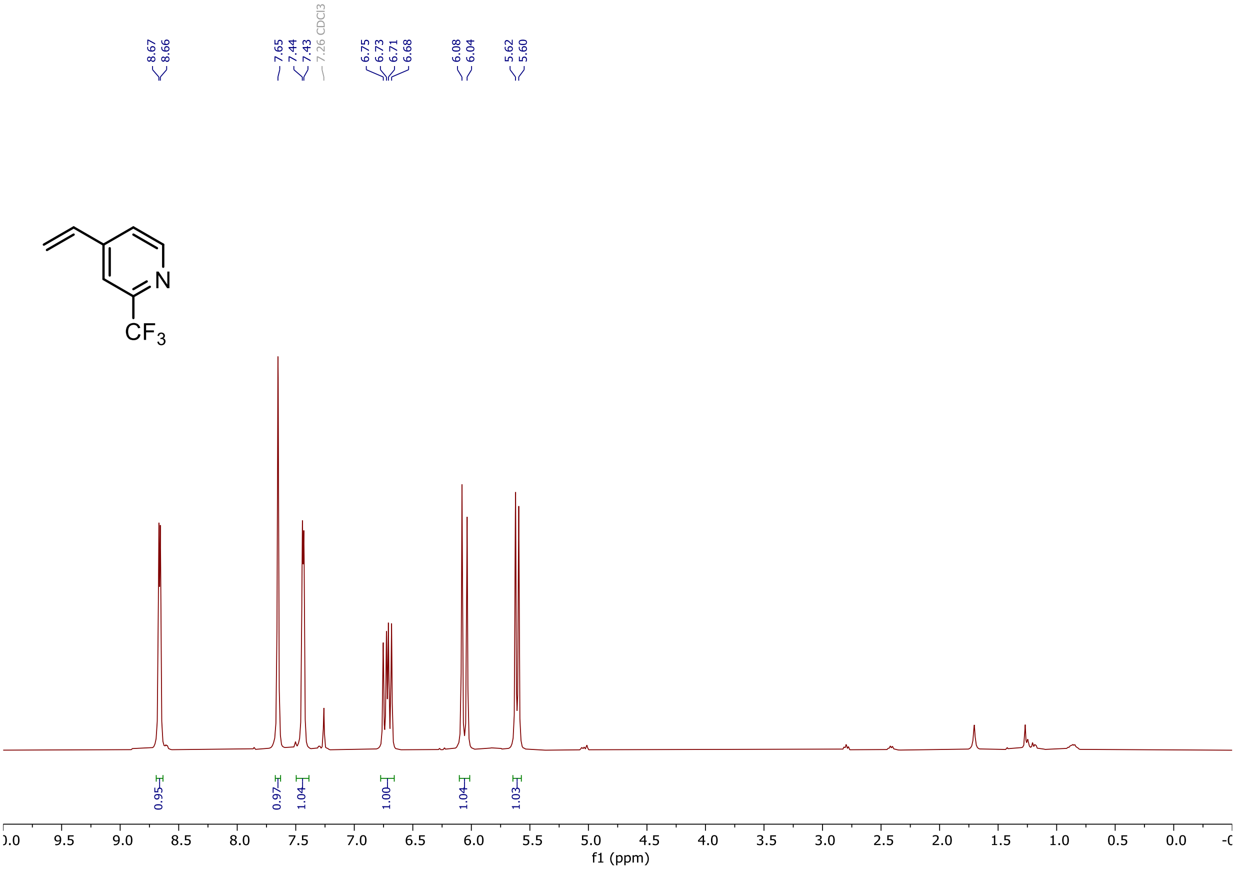

^{13}C NMR (101 MHz, $CDCl_3$) of 2-(trifluoromethyl)-4-vinylpyridine

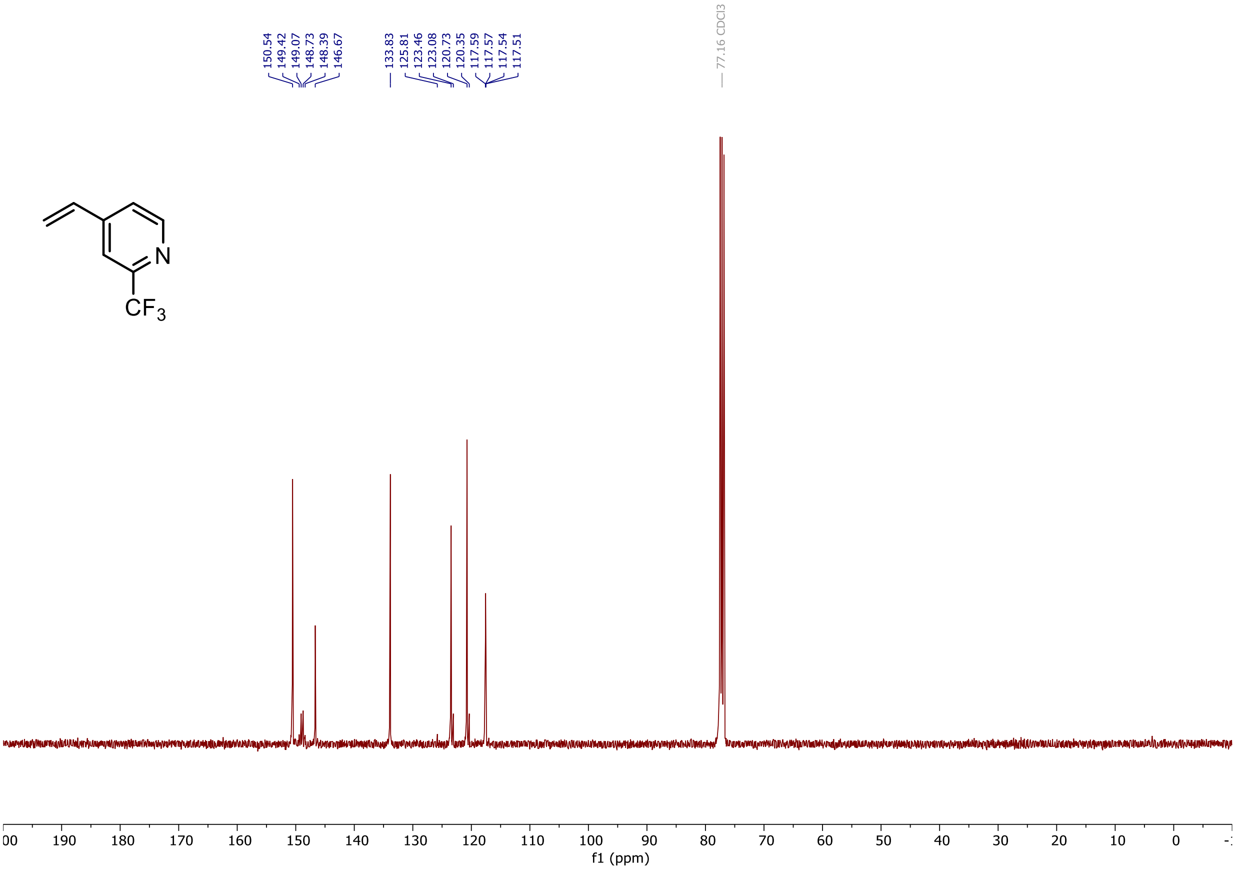

^{19}F NMR (377 MHz, $CDCl_3$) of 2-(trifluoromethyl)-4-vinylpyridine

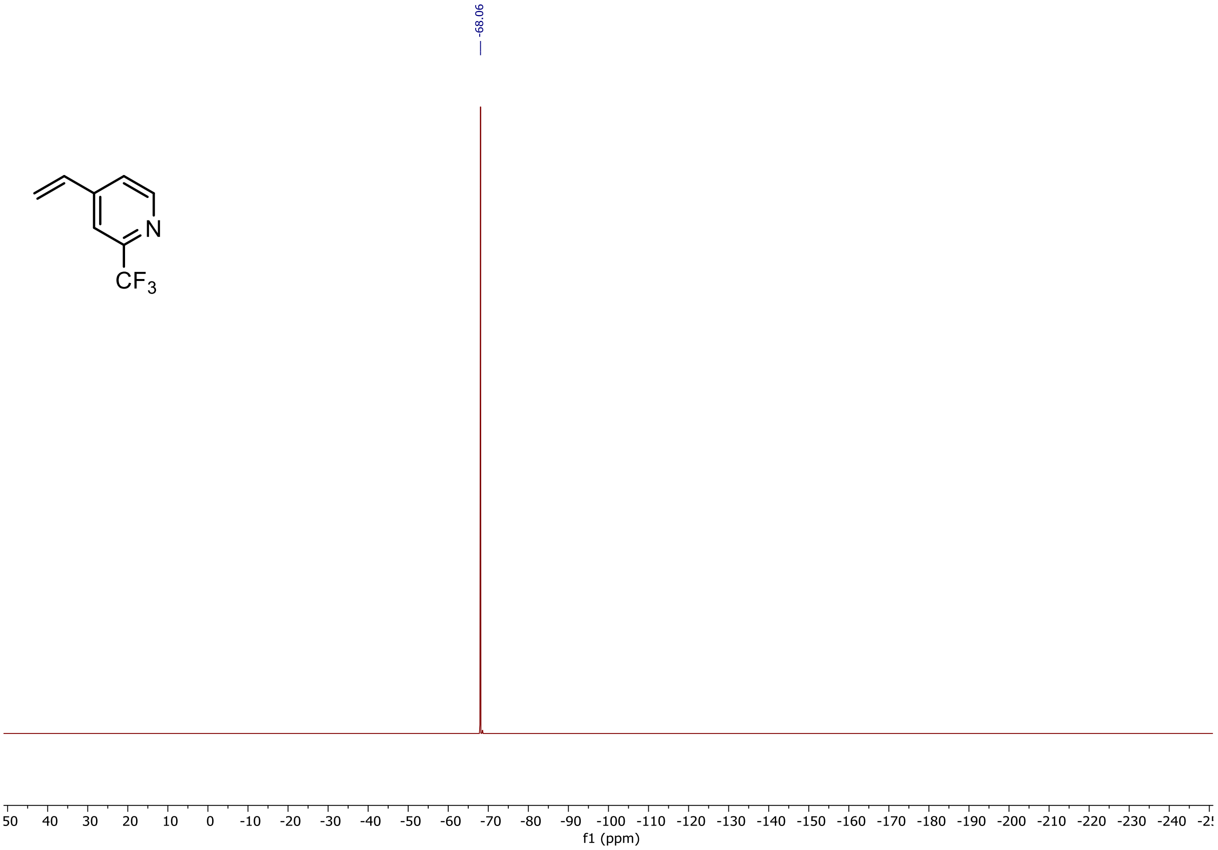

^{1}H NMR (400 MHz, $CDCl_3$) of 4-(prop-1-en-2-yl)pyridine (see procedure)

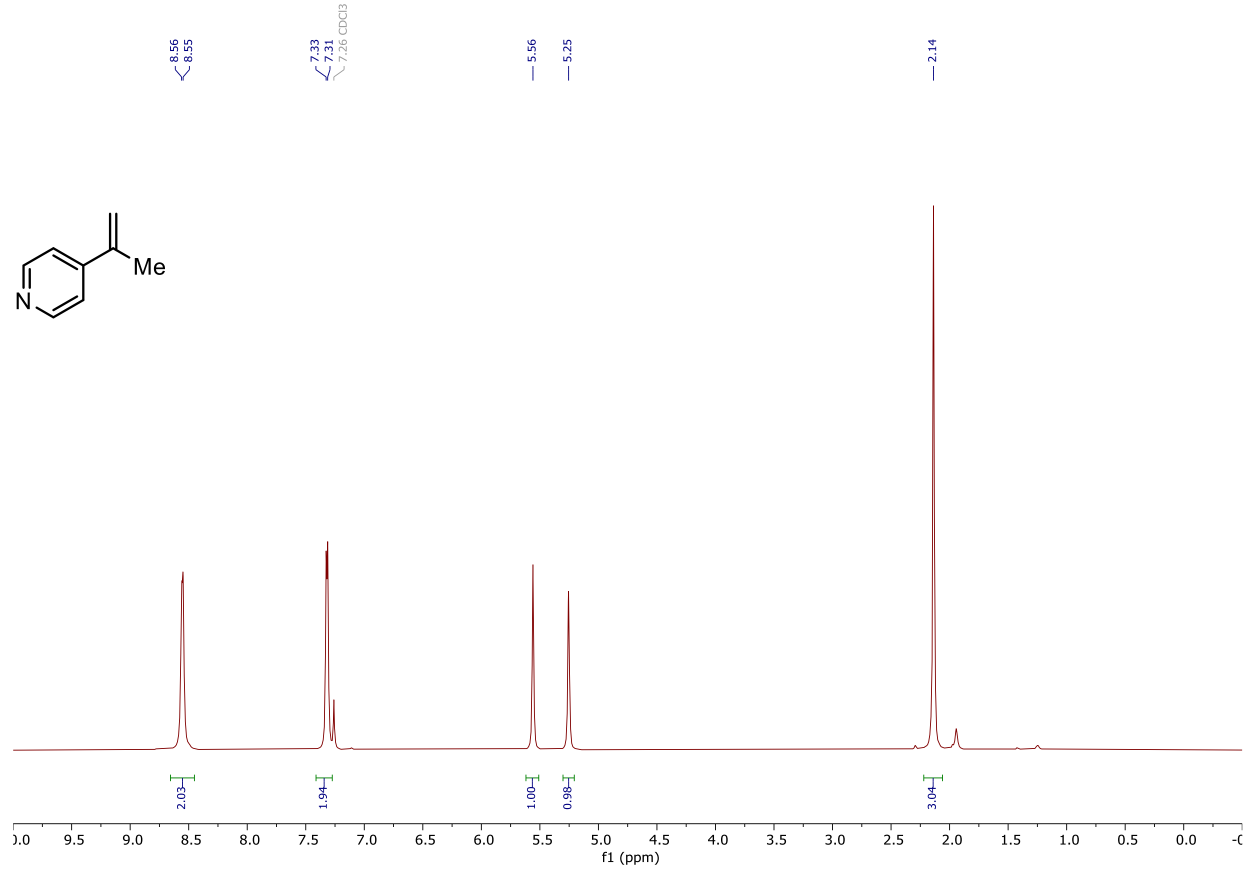

^{13}C NMR (101 MHz, $CDCl_3$) of 4-(prop-1-en-2-yl)pyridine

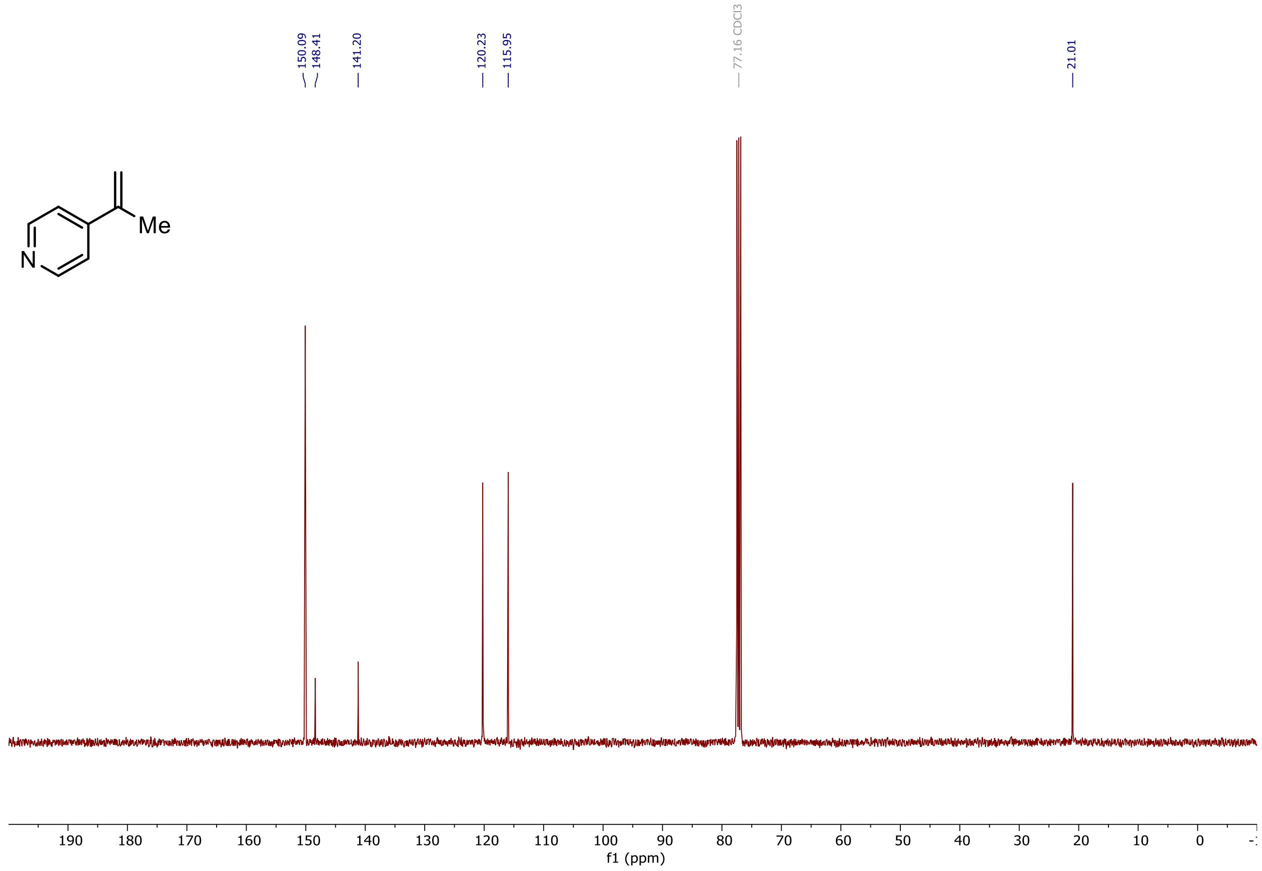

^1H NMR (400 MHz, $CDCl_3$) of 4-(1-phenylvinyl)pyridine (see procedure)

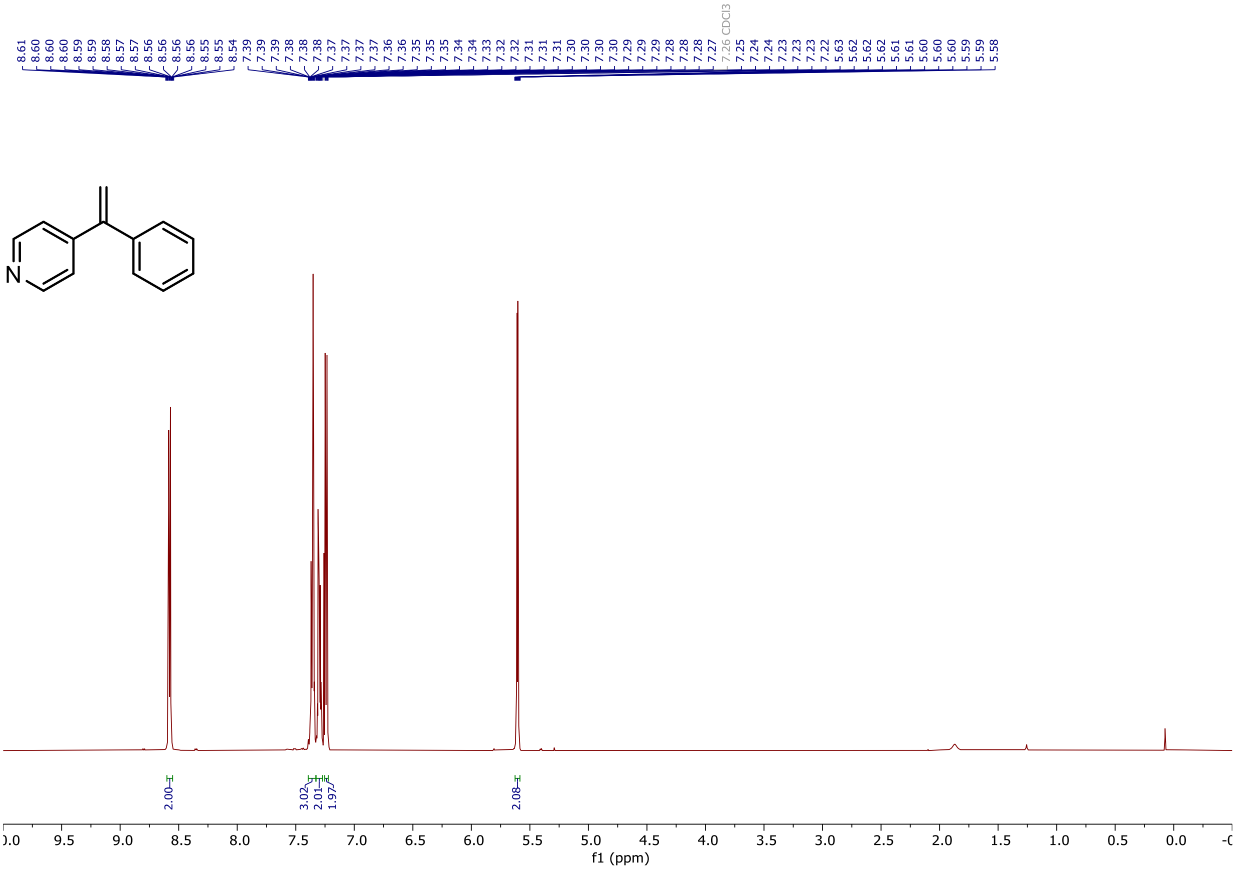

^{13}C NMR (101 MHz, $CDCl_3$) of 4-(1-phenylvinyl)pyridine

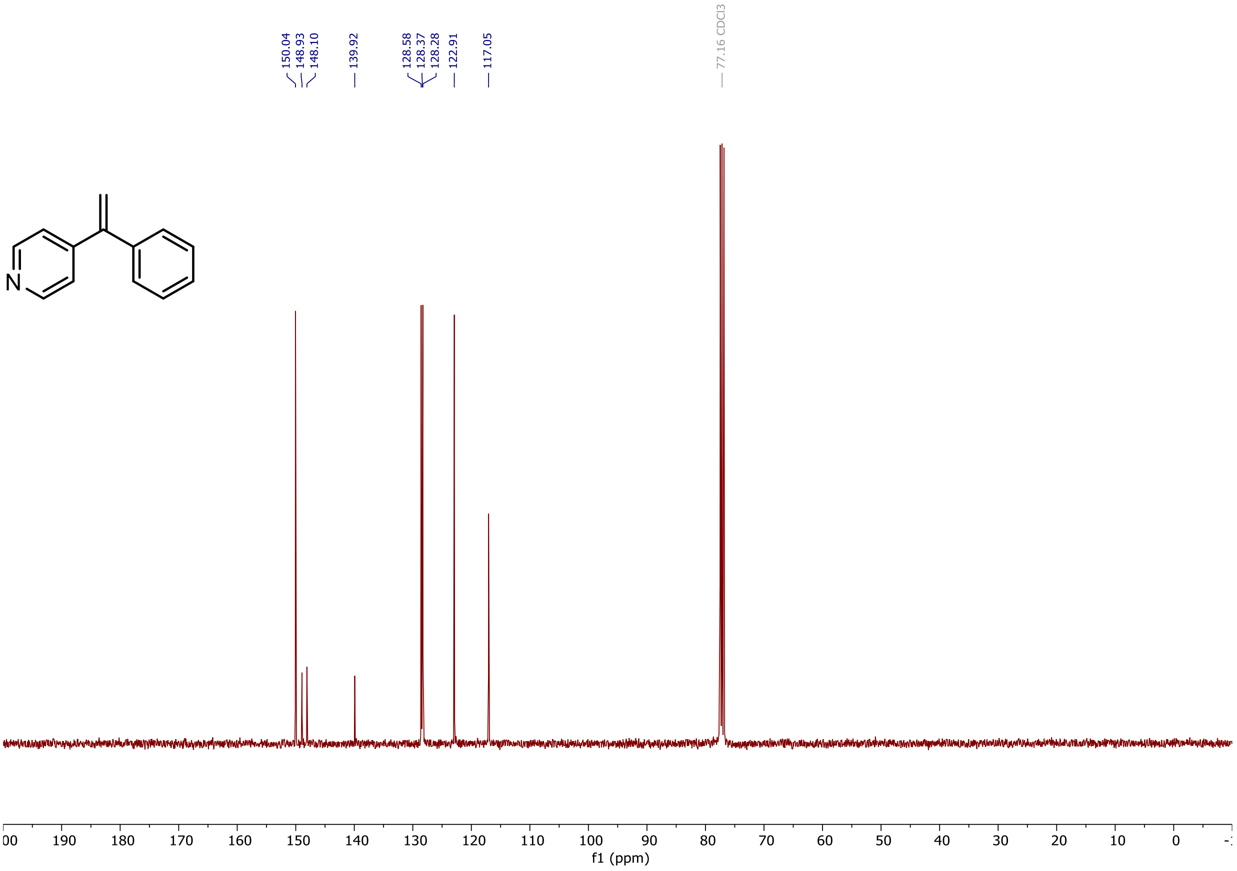

^{1}H NMR (400 MHz, $CDCl_3$) of **S1** (*see procedure*)

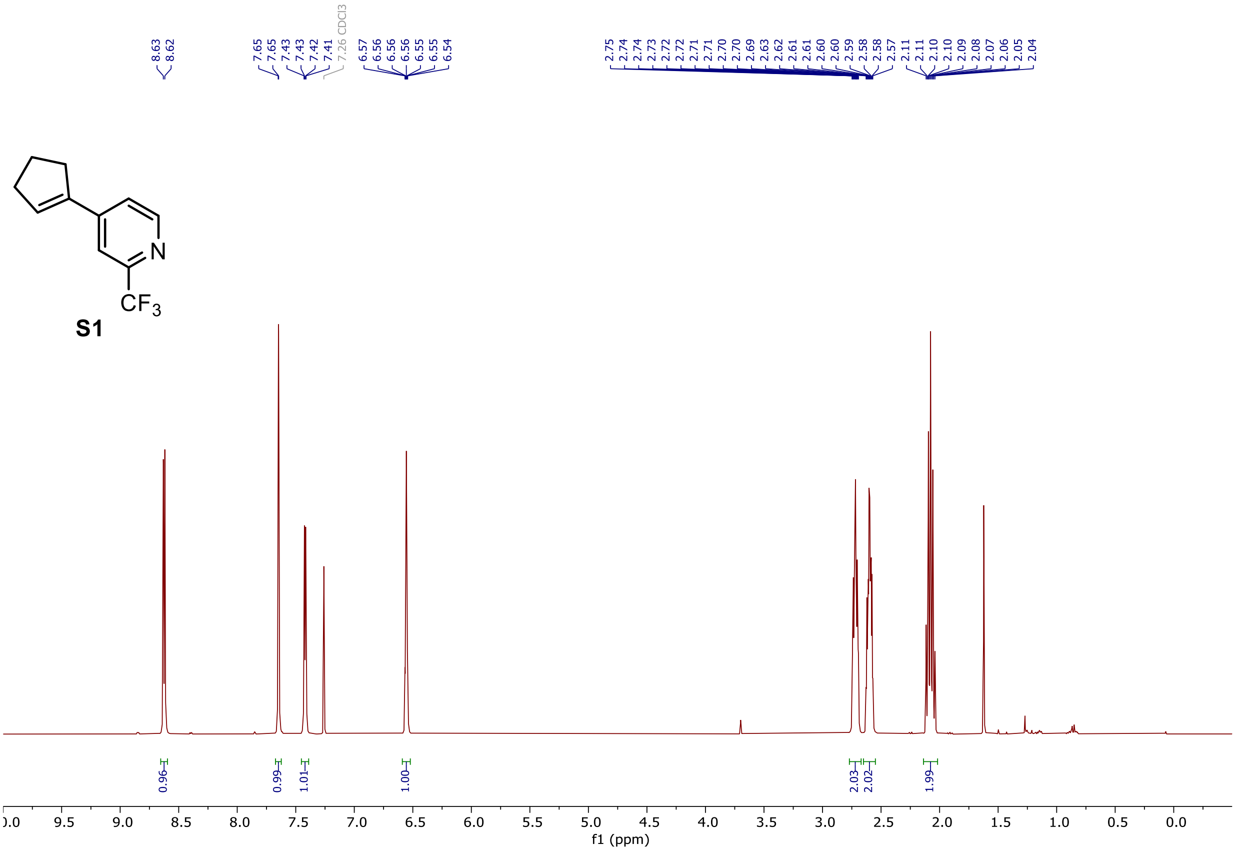

^{13}C NMR (101 MHz, $CDCl_3$) of **S1**

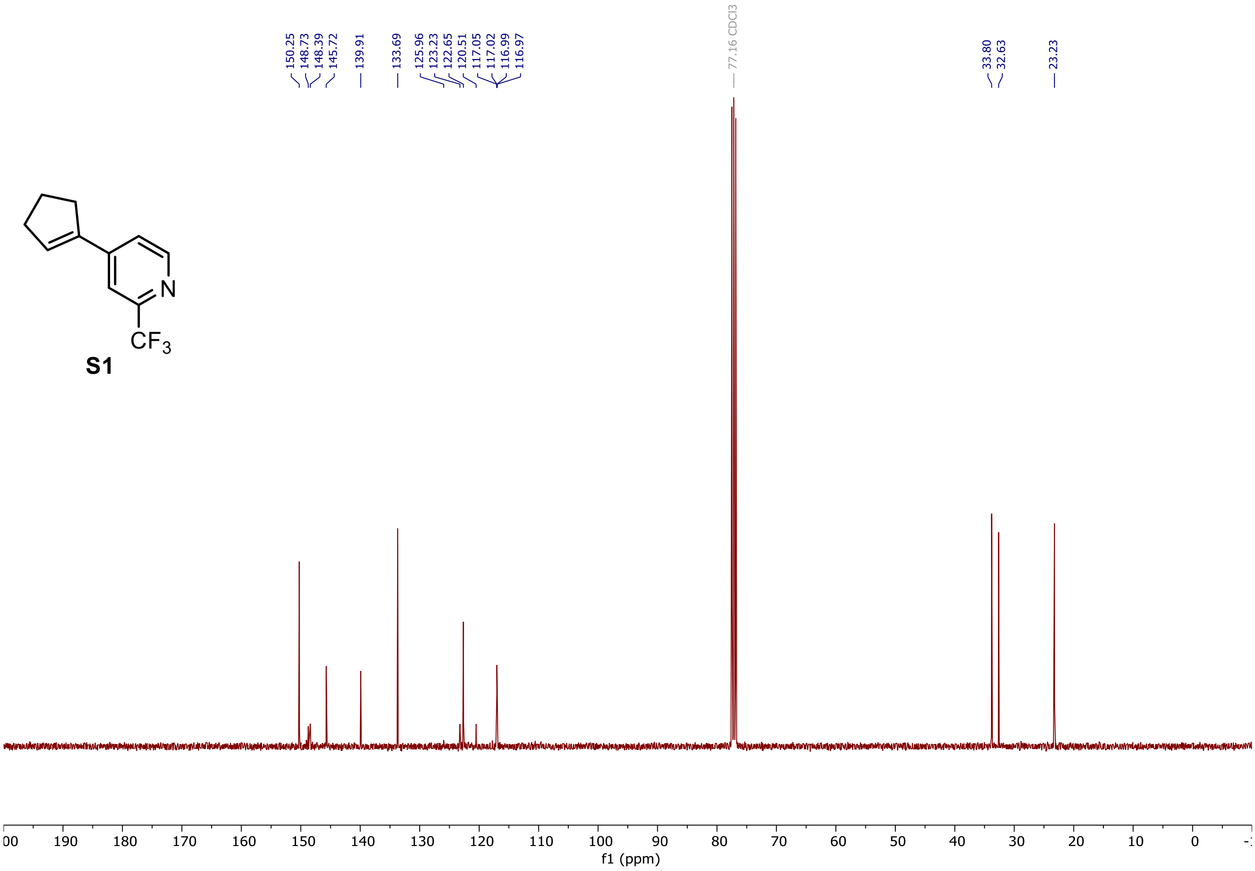

^{19}F NMR (377 MHz, $CDCl_3$) of **S1**

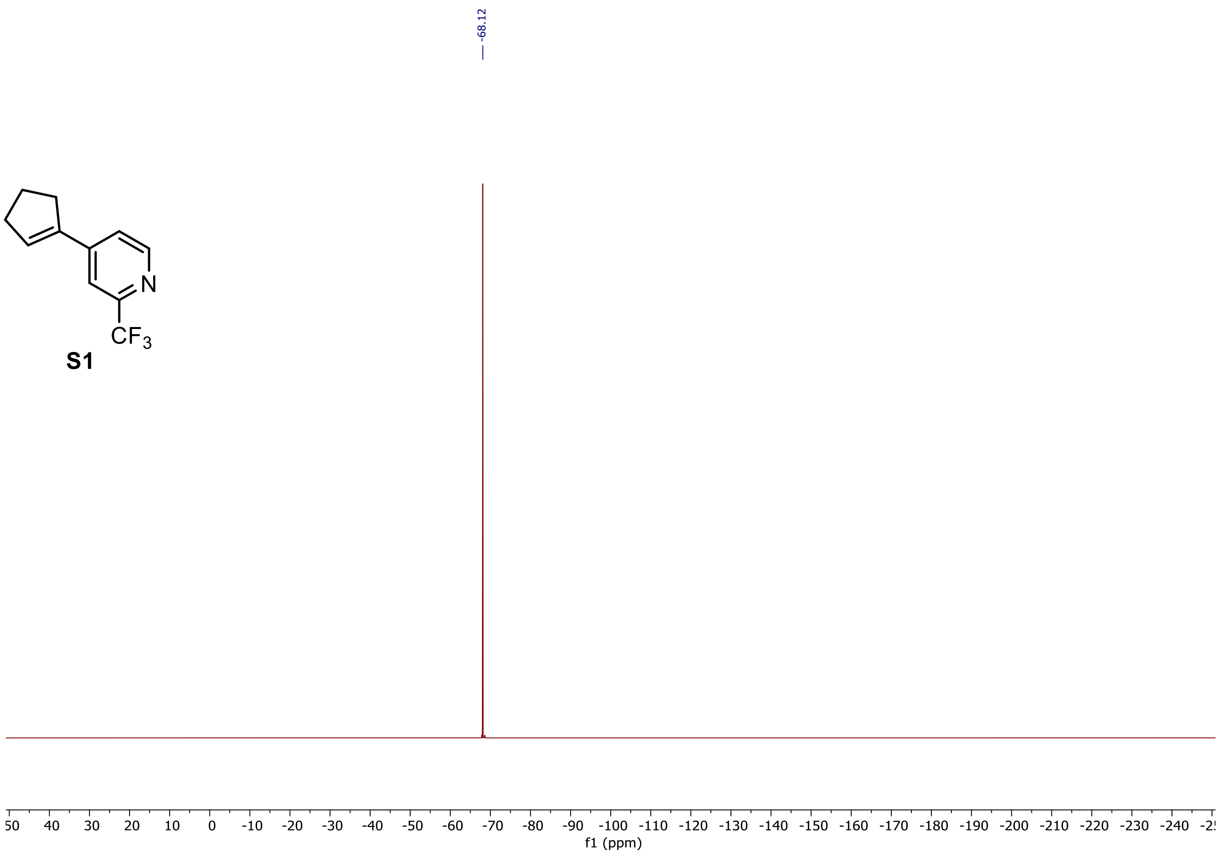

^{1}H NMR (400 MHz, $CDCl_3$) of 2-vinylpyrimidine (see procedure)

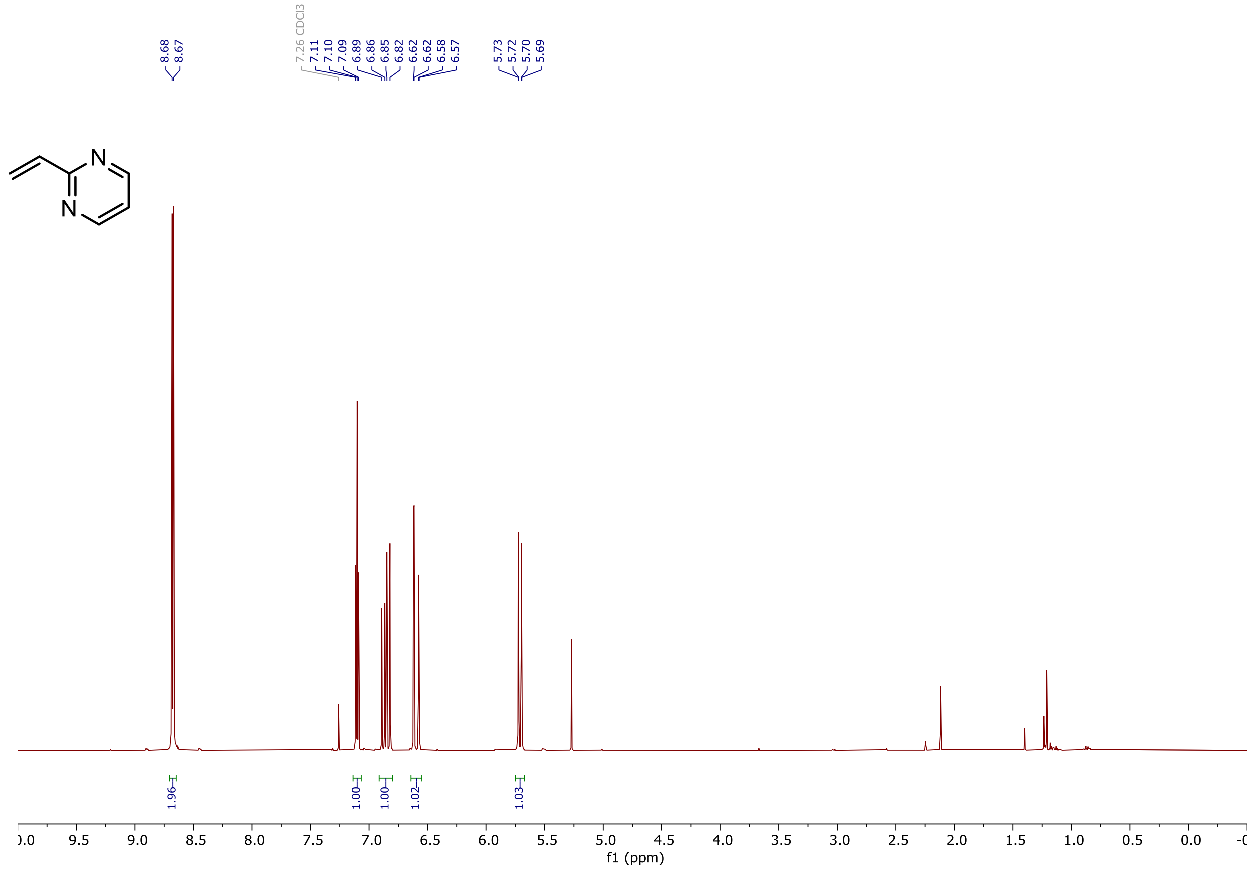

^{13}C NMR (101 MHz, $CDCl_3$) of 2-vinylpyrimidine

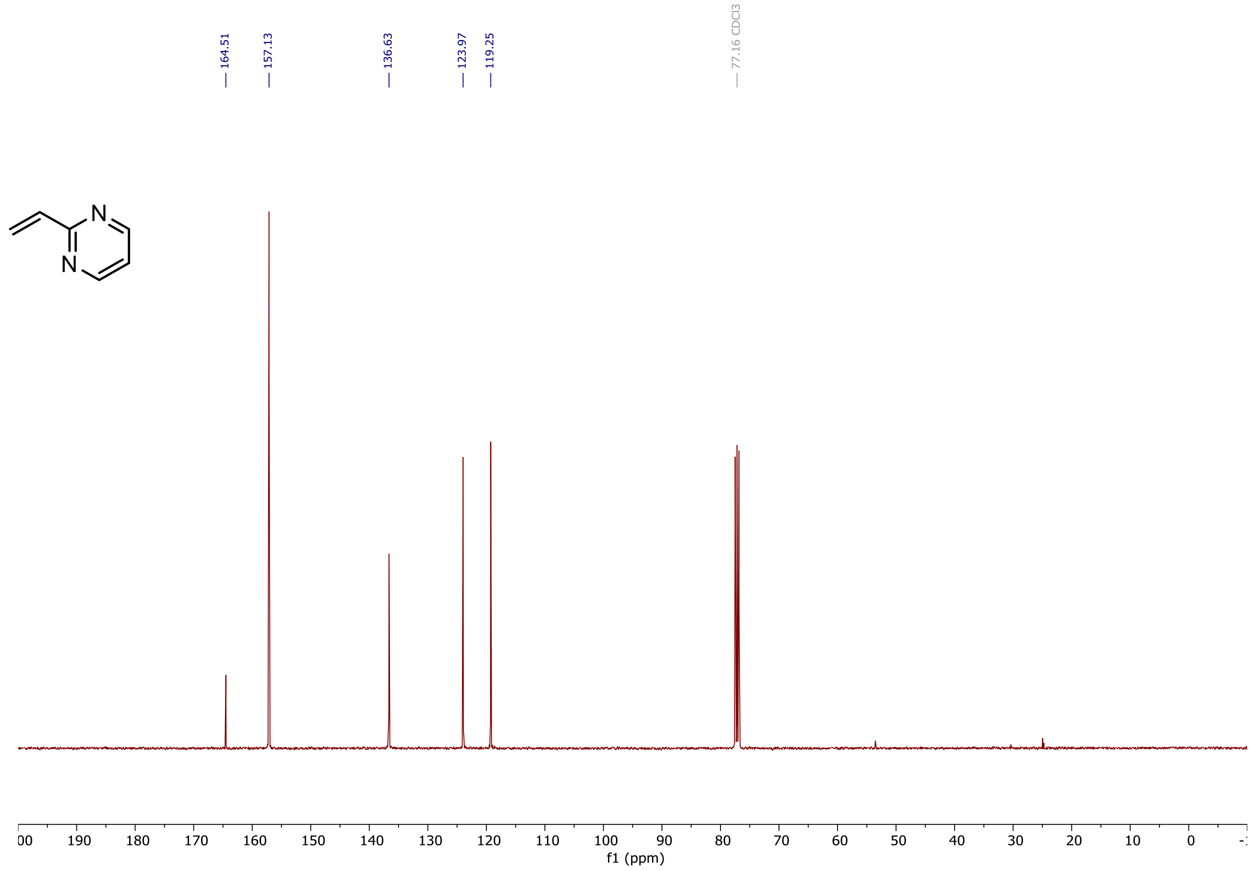

^{1}H NMR (400 MHz, $CDCl_3$) of 1-(methylsulfonyl)-4-vinylbenzene (see procedure)

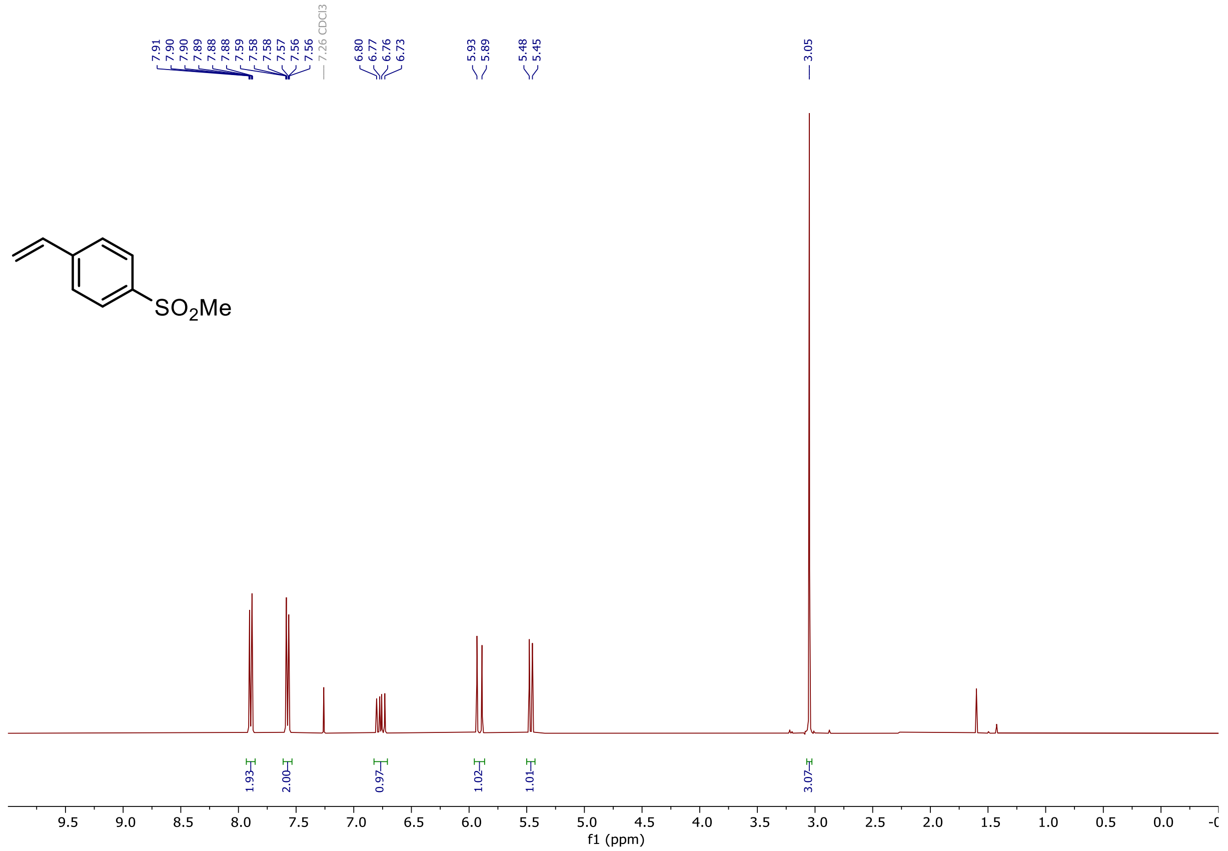

^{13}C NMR (101 MHz, $CDCl_3$) of 1-(methylsulfonyl)-4-vinylbenzene

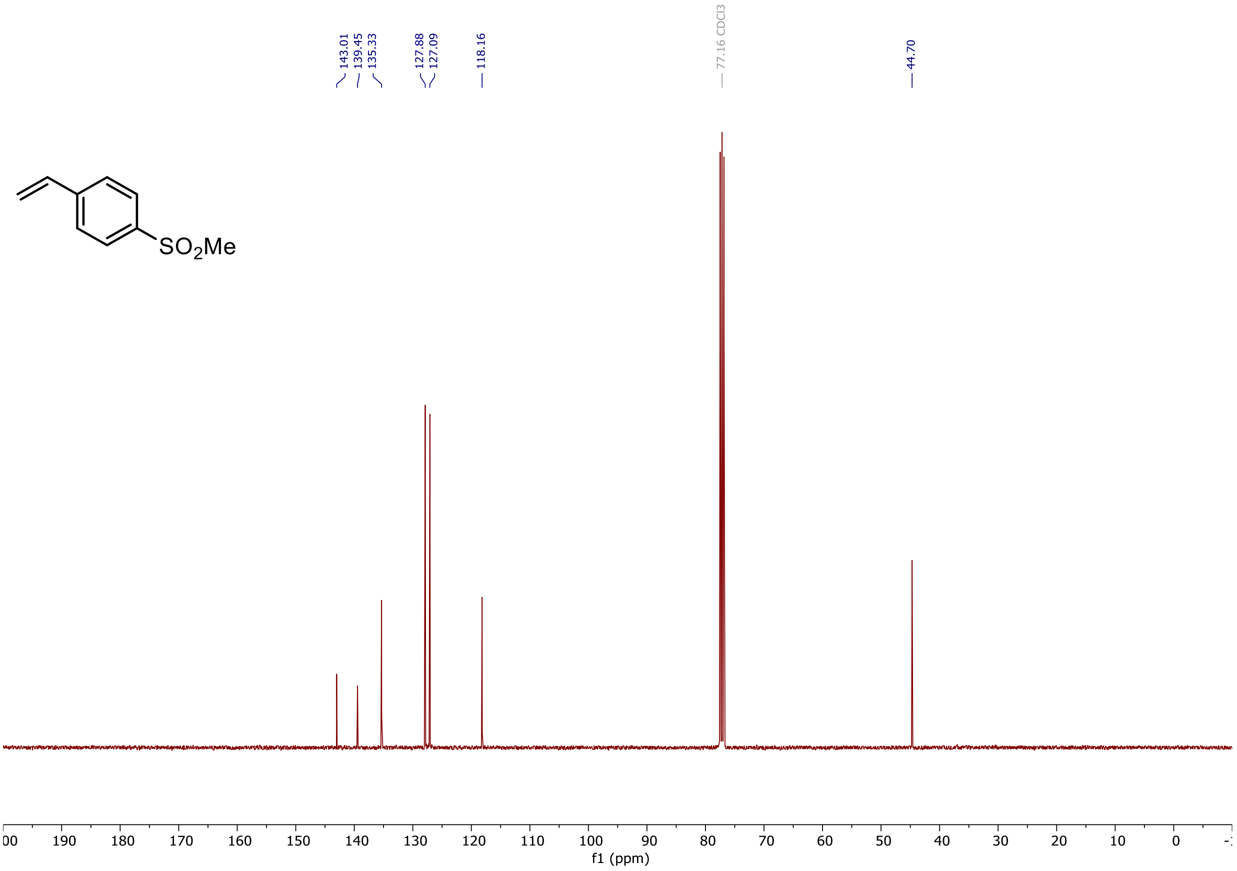

^{1}H NMR (400 MHz, $CDCl_3$) of *N*,*N*-dimethyl-4-vinylbenzenesulfonamide (*see procedure*)

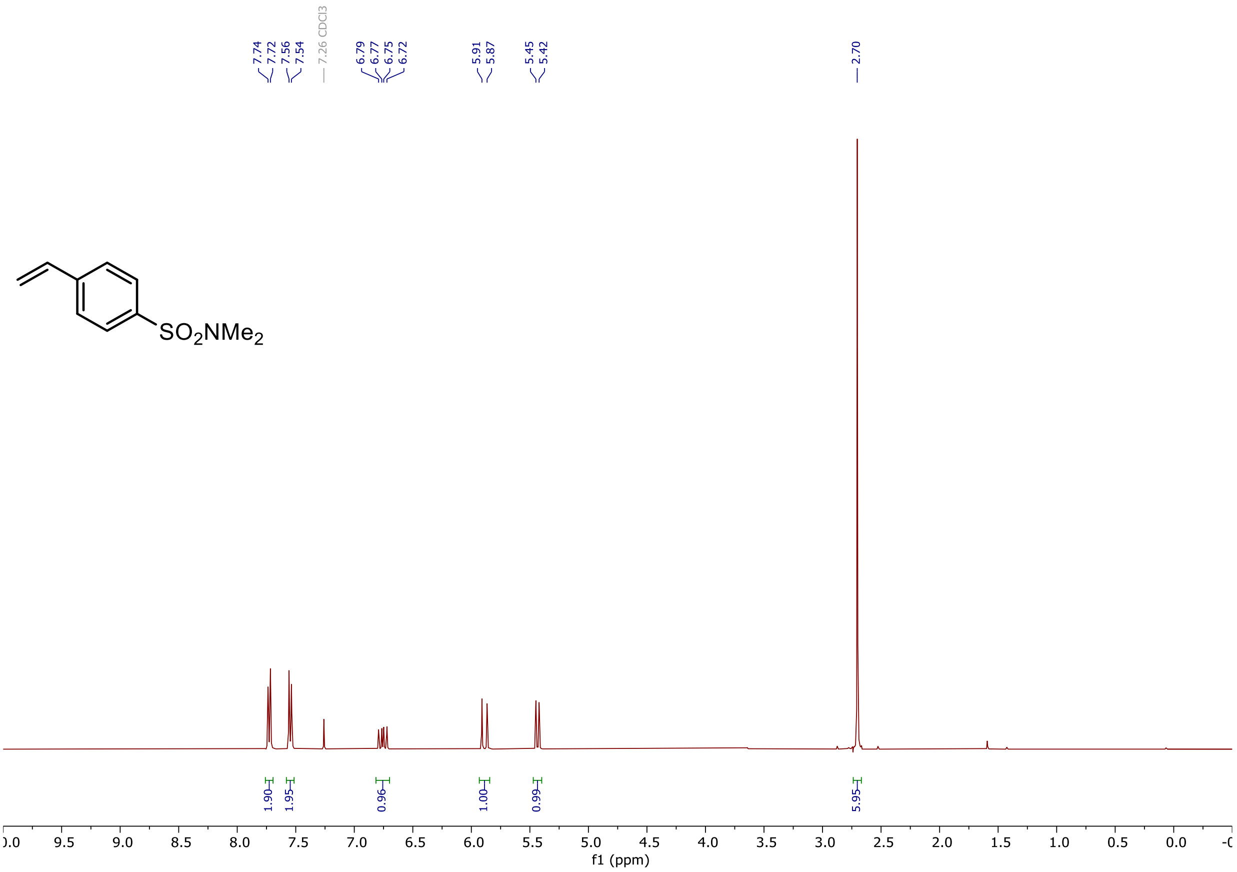

^{13}C NMR (101 MHz, $CDCl_3$) of *N*,*N*-dimethyl-4-vinylbenzenesulfonamide

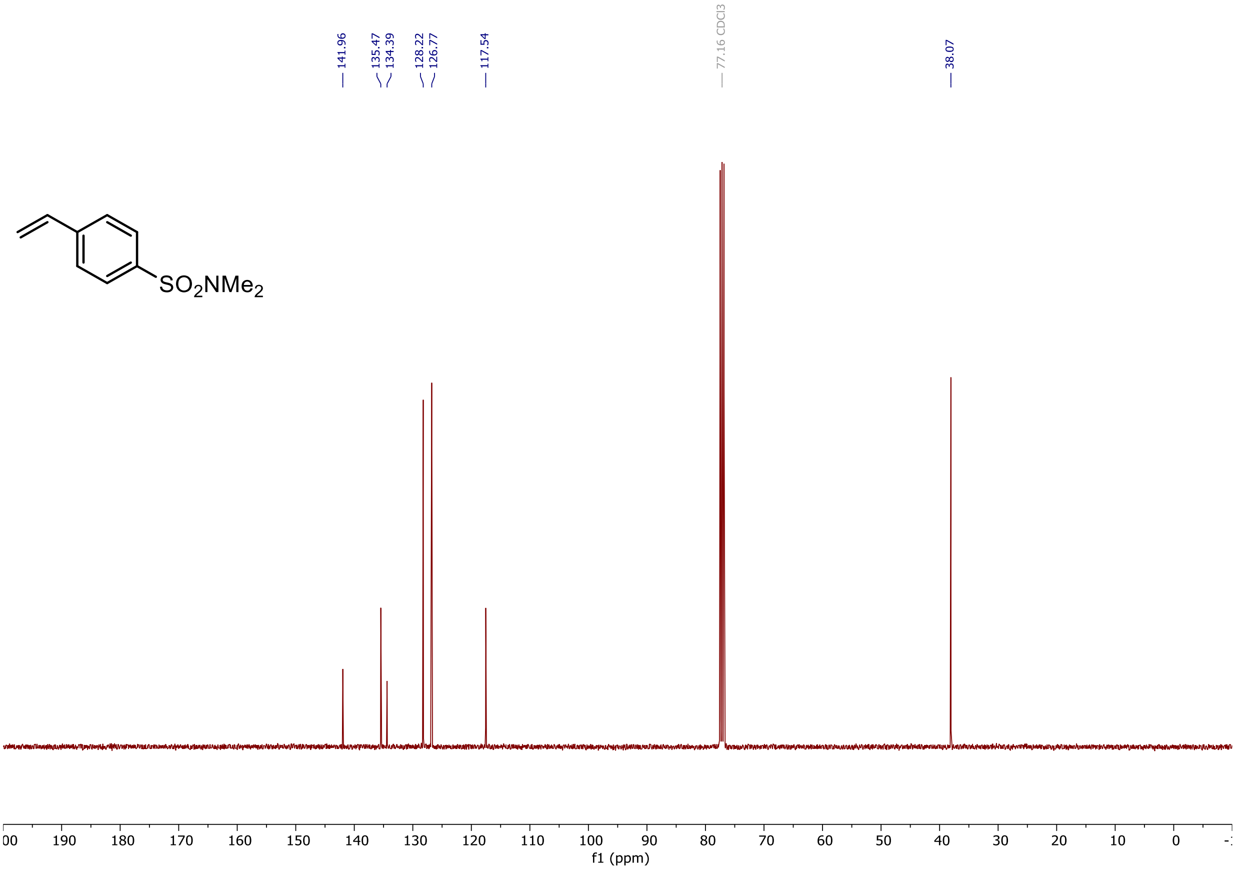

^{1}H NMR (400 MHz, $CDCl_3$) of *N*,2-diphenylacrylamide (*see procedure*)

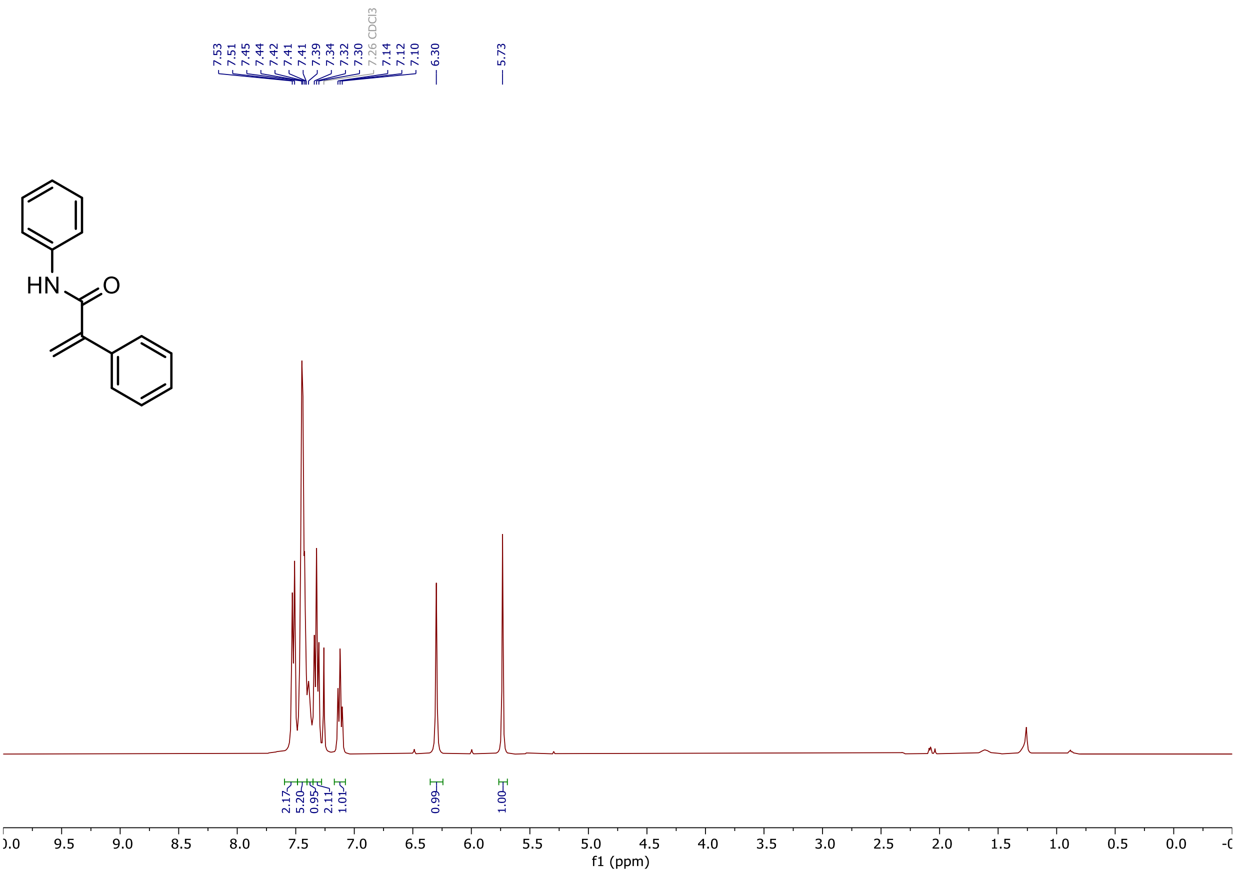

^{13}C NMR (101 MHz, $CDCl_3$) of *N*,2-diphenylacrylamide

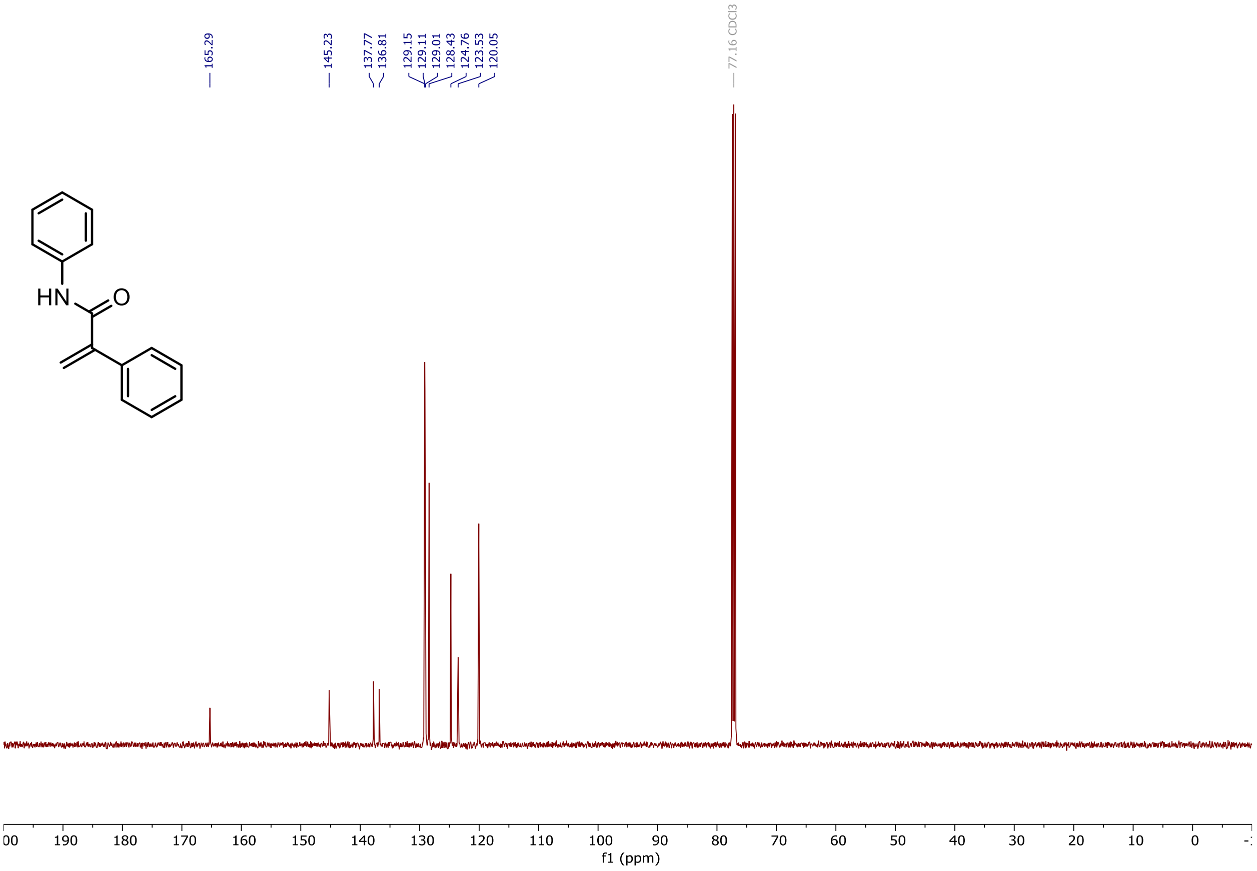

^{1}H NMR (400 MHz, $CDCl_3$) of **S2** (*see procedure*)

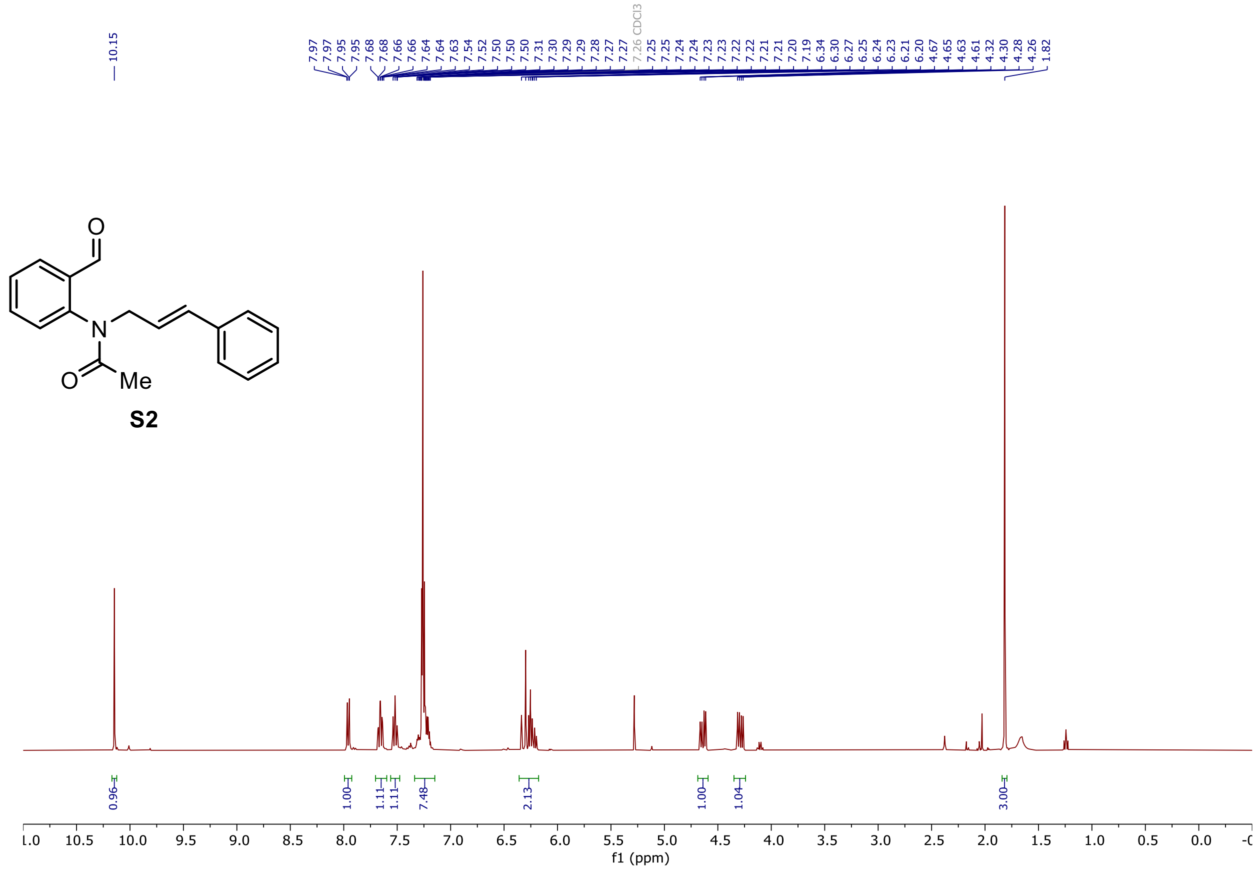

^{13}C NMR (101 MHz, $CDCl_3$) of **S2**

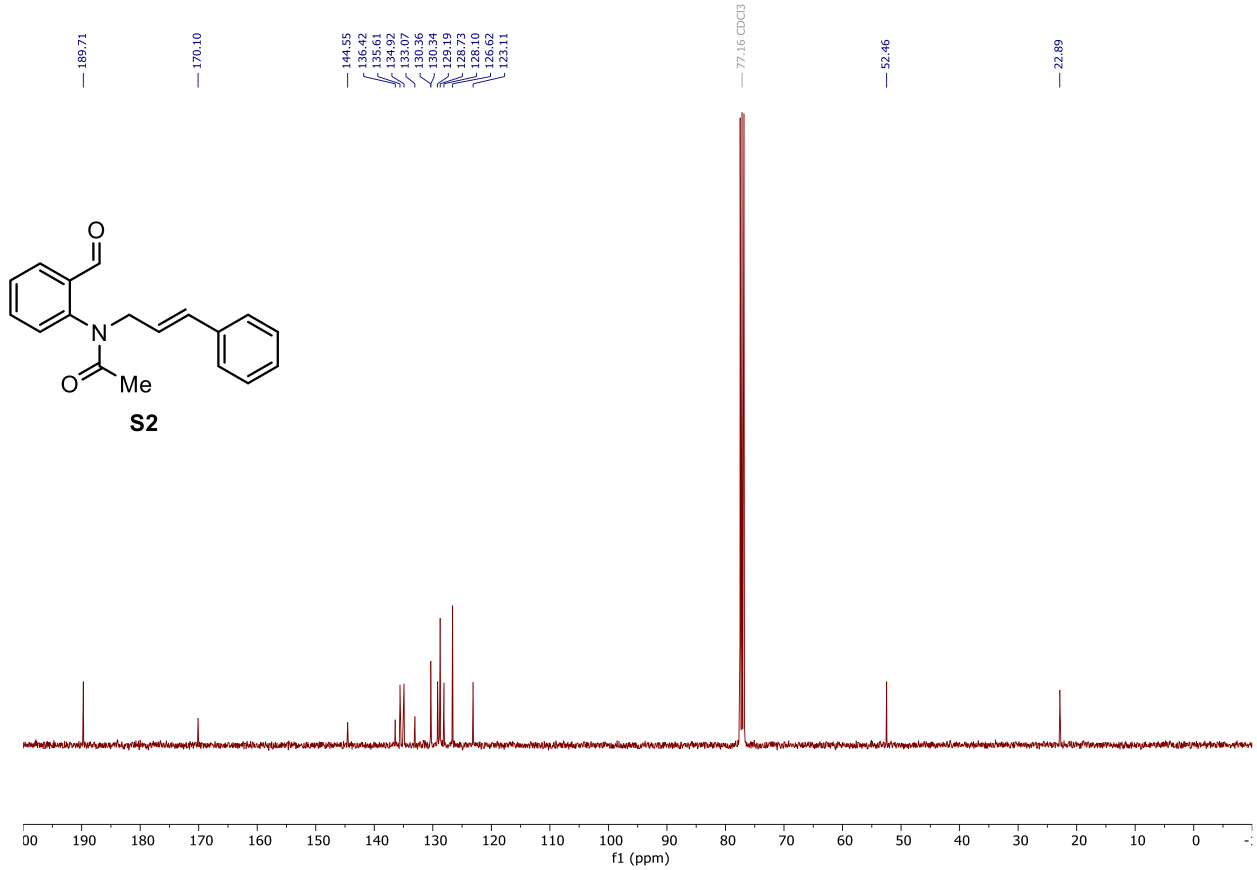

^{1}H NMR (400 MHz, $CDCl_3$) of **S3** (*see procedure*)

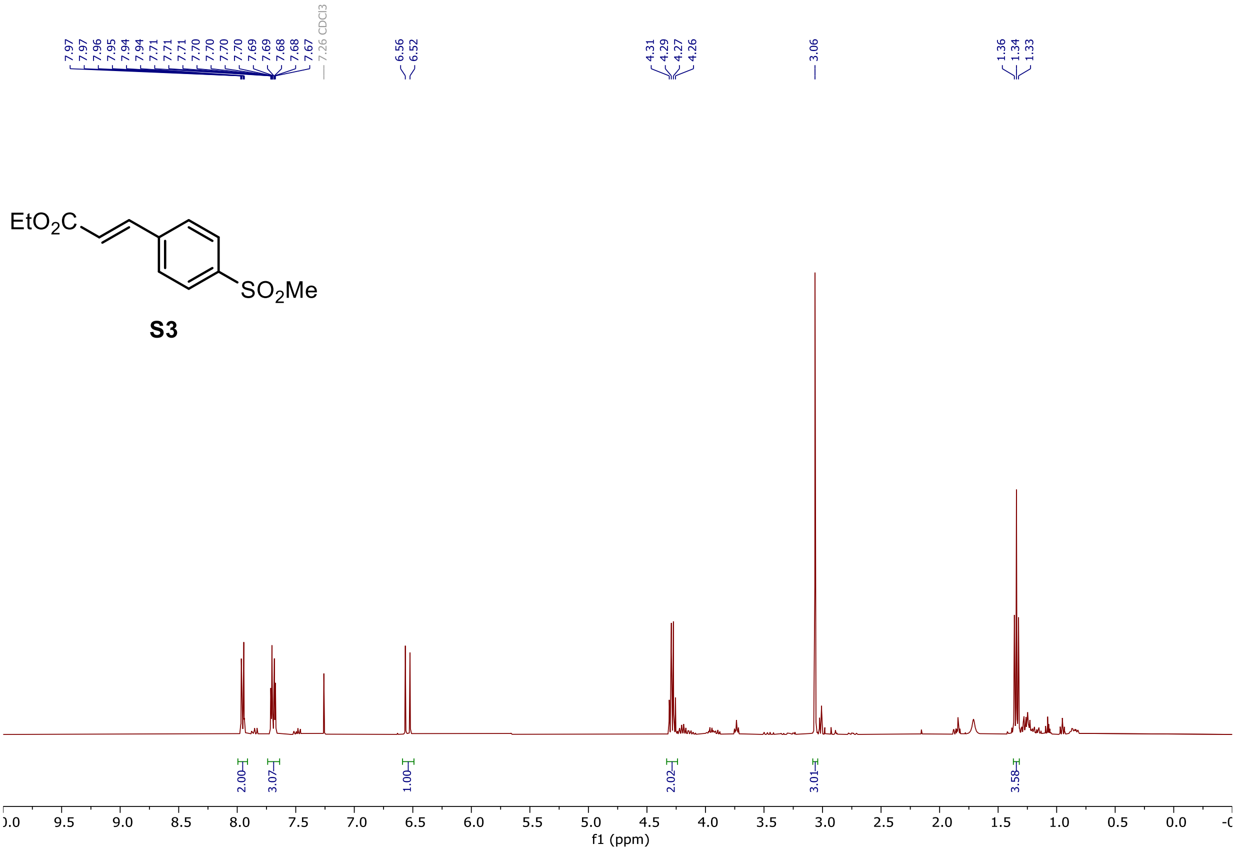

^{1}H NMR (400 MHz, $CDCl_3$) of **S4** (*see procedure*)

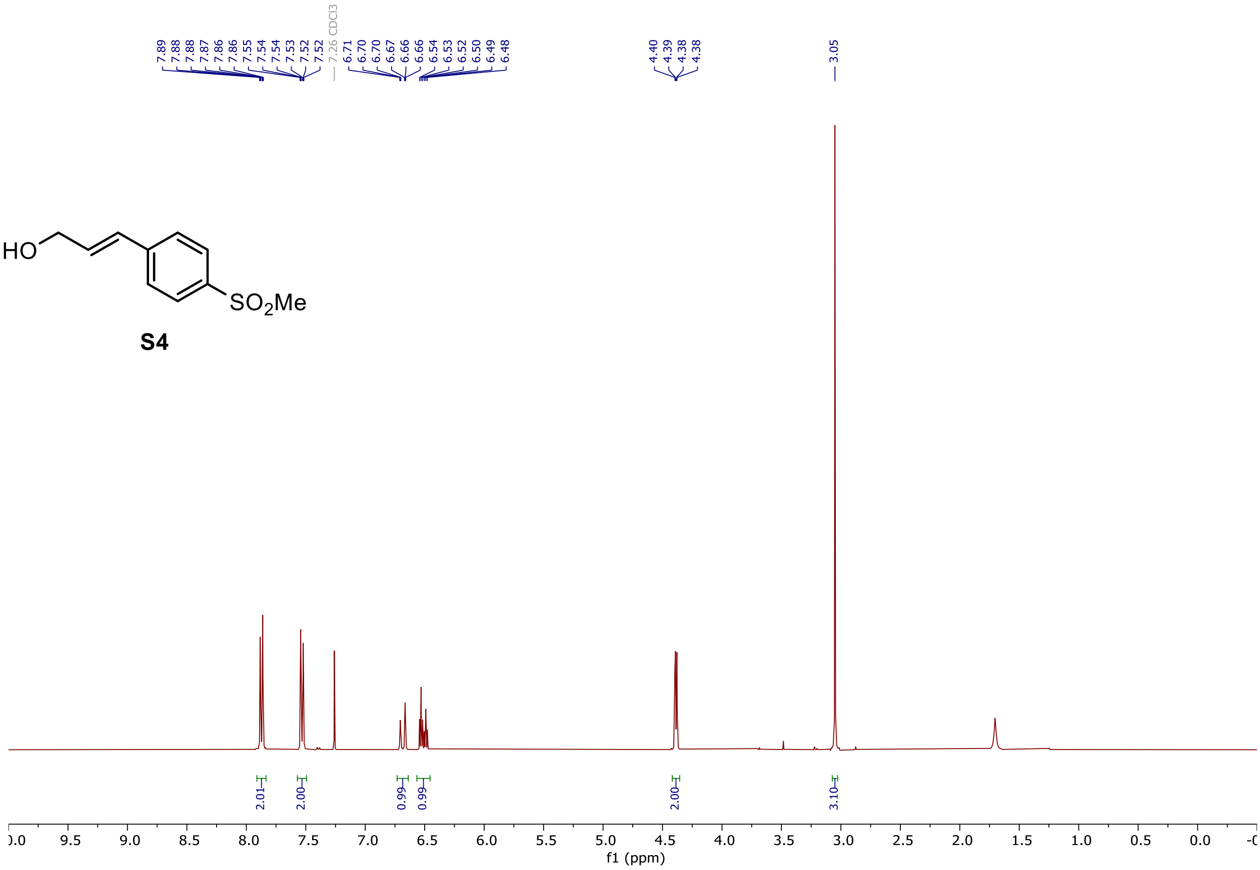

^{1}H NMR (400 MHz, $CDCl_3$) of **S5** (*see procedure*)

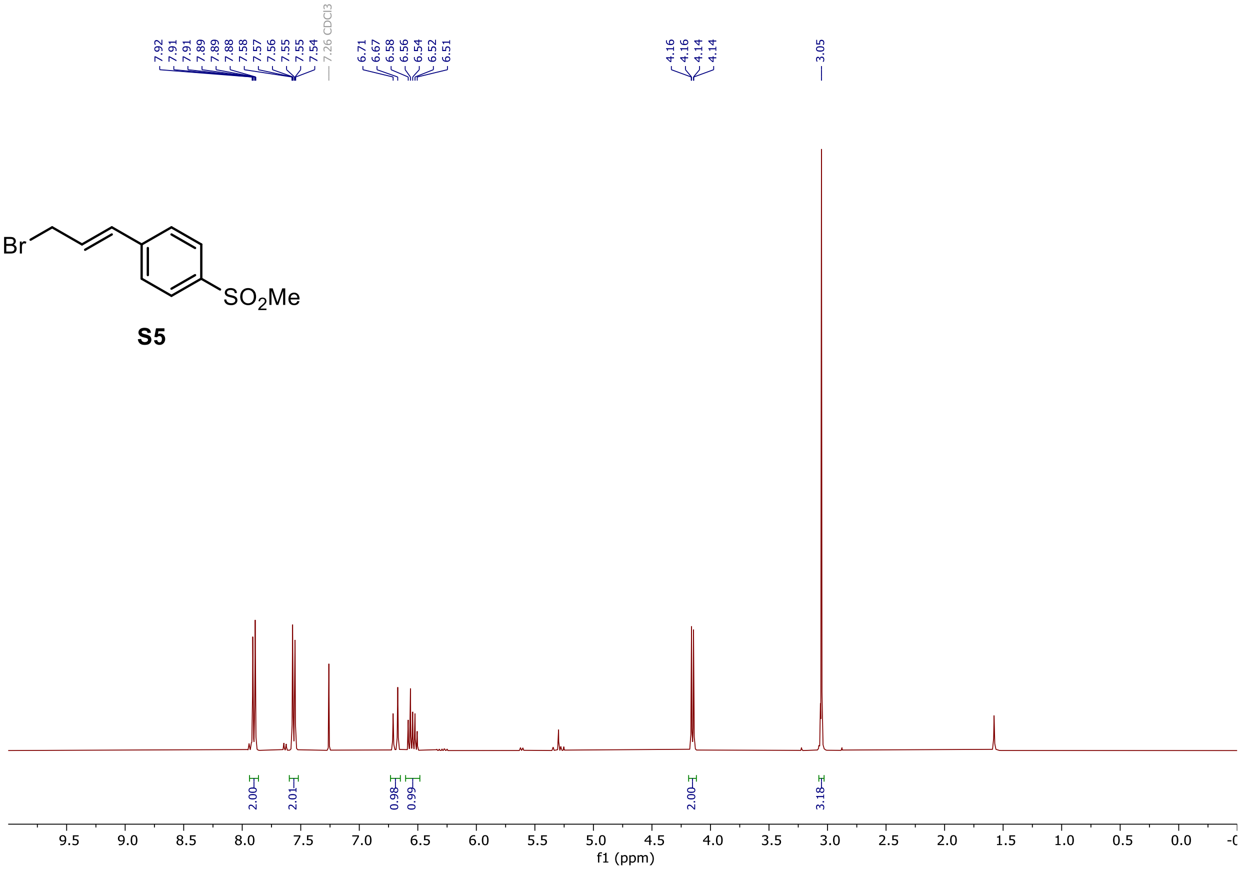

^{13}C NMR (101 MHz, $CDCl_3$) of **S5**

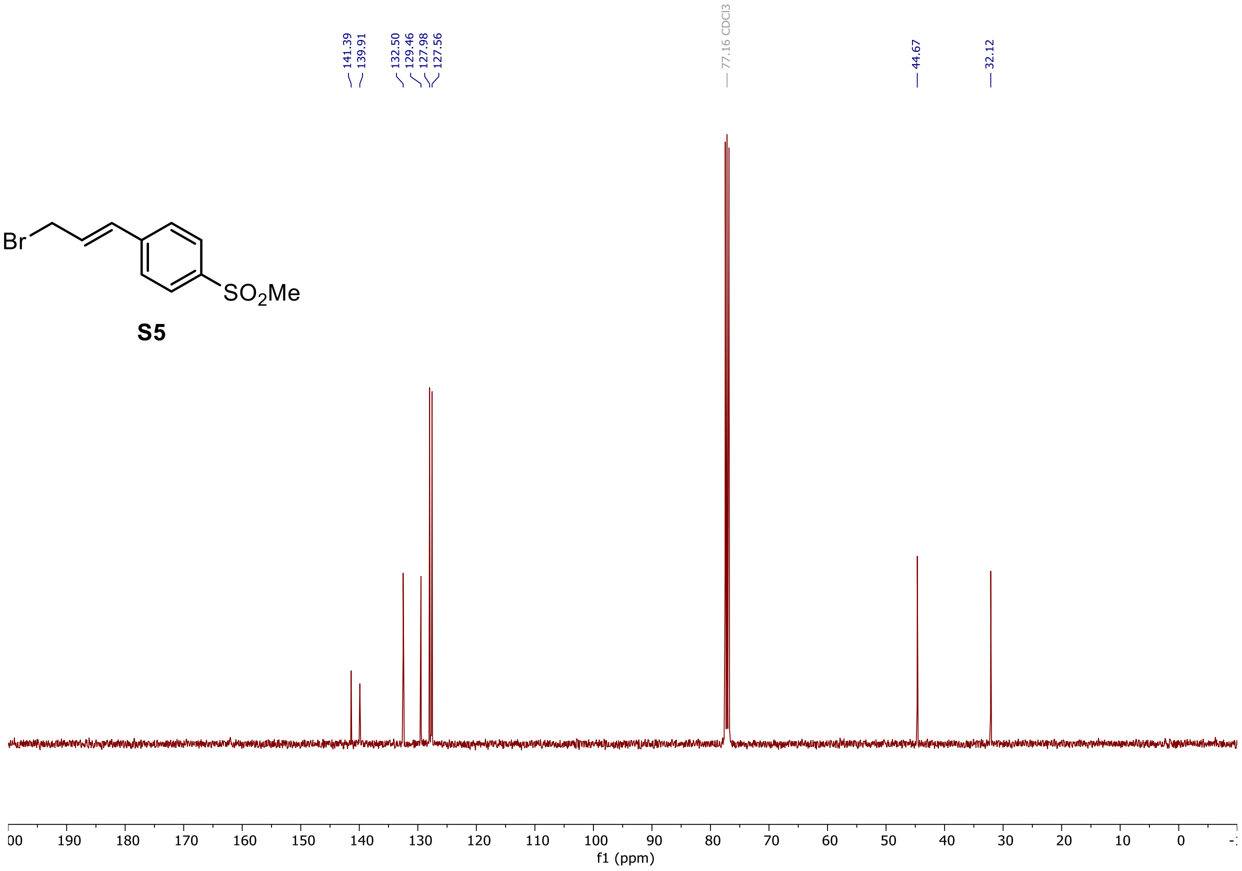

^{1}H NMR (400 MHz, $CDCl_3$) of **S6** (*see procedure*)

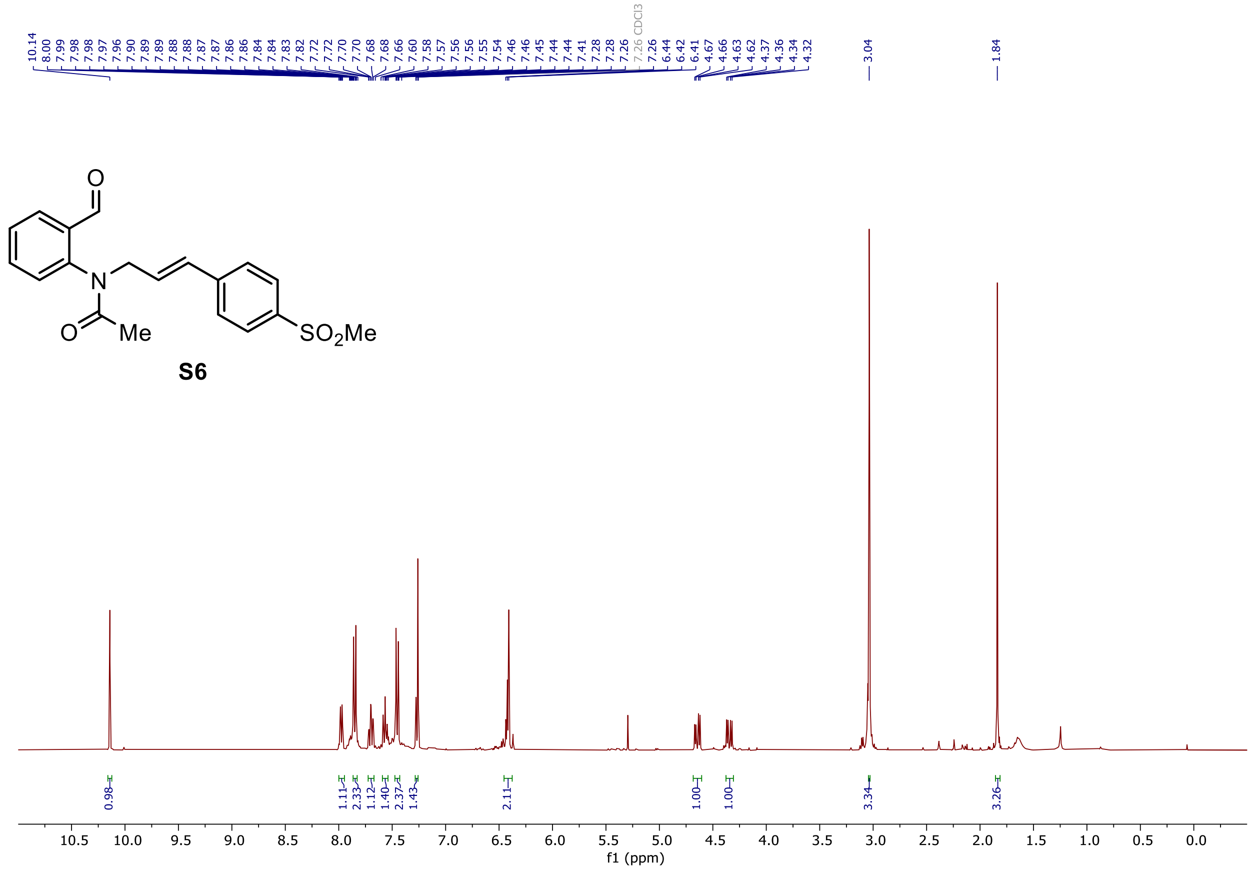

^{13}C NMR (101 MHz, $CDCl_3$) of **S6**

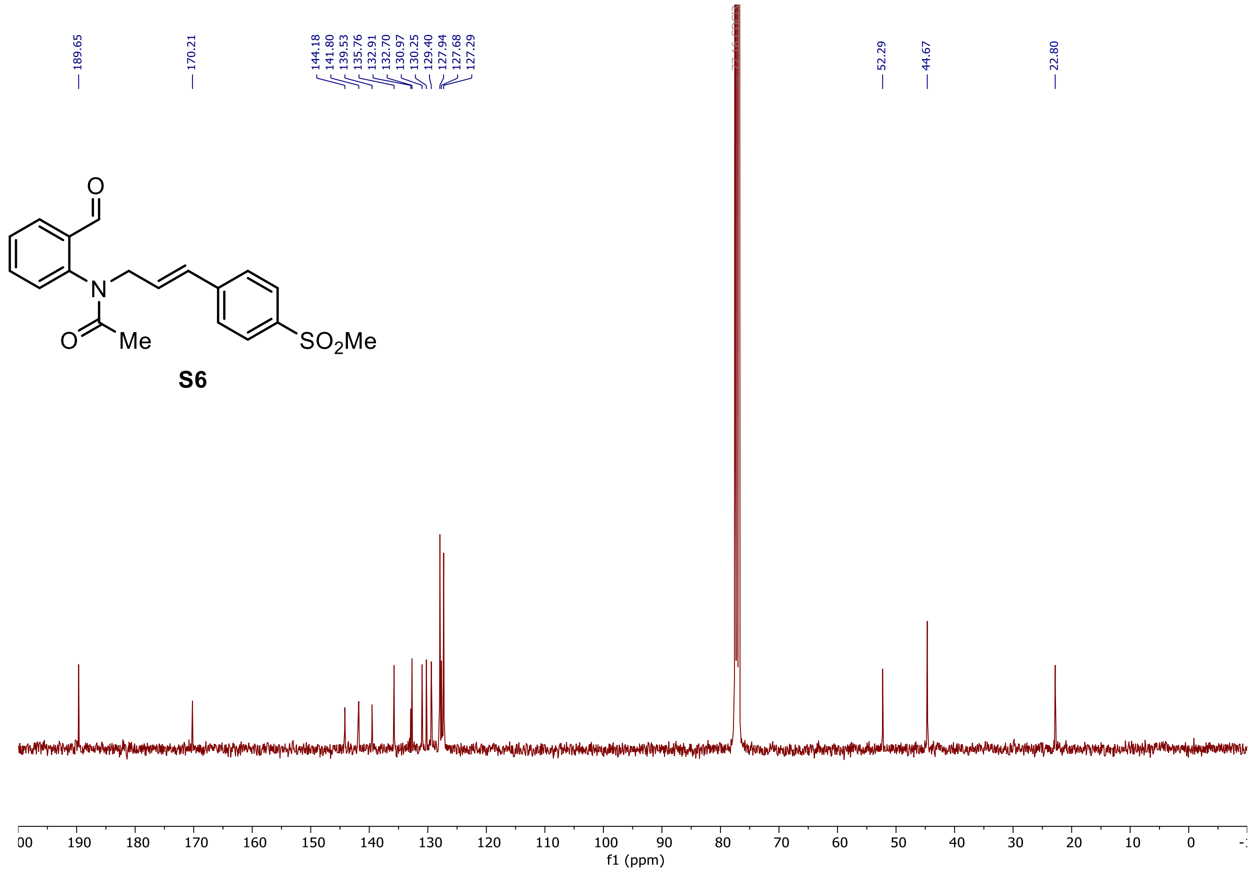

^{1}H NMR (400 MHz, $CDCl_3$) of **S7** (*see procedure*)

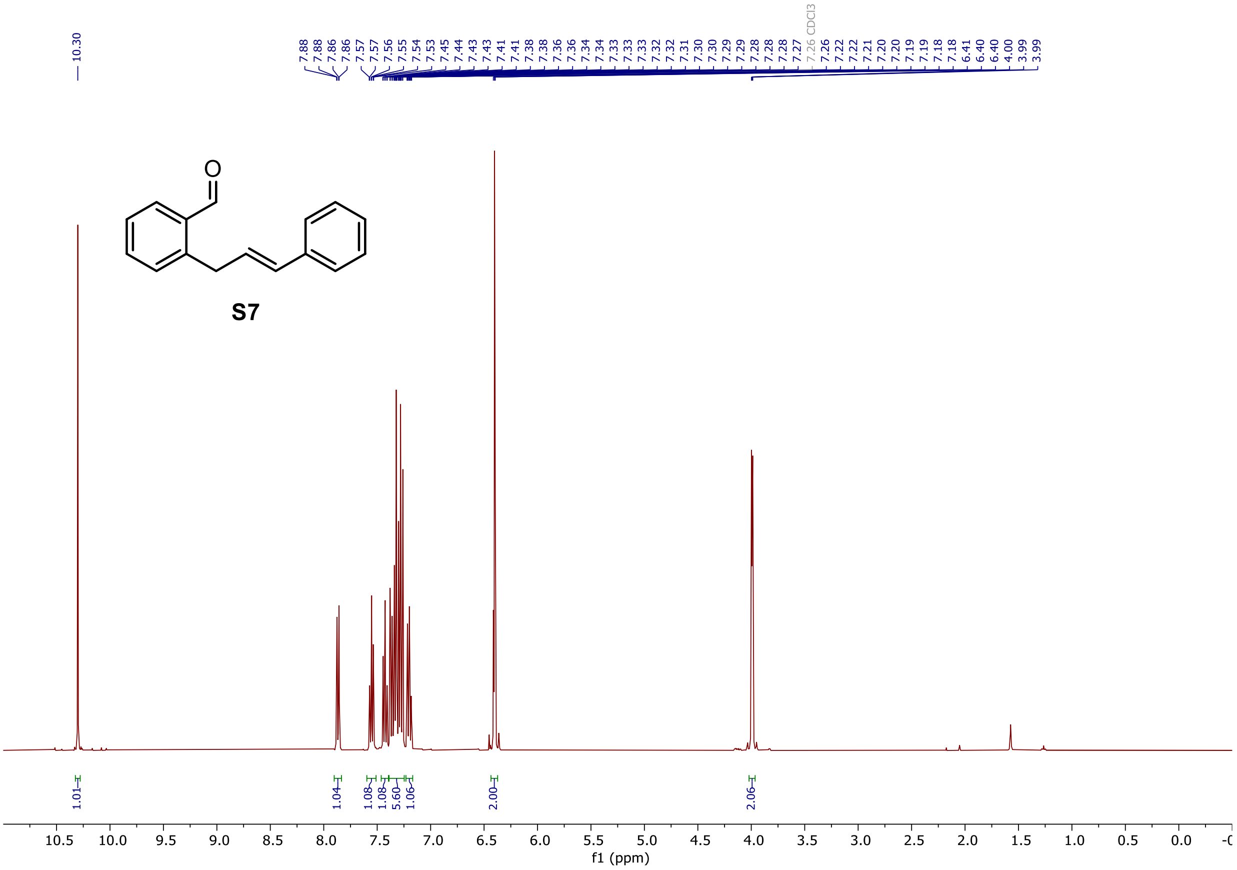

^{13}C NMR (101 MHz, $CDCl_3$) of **S7**

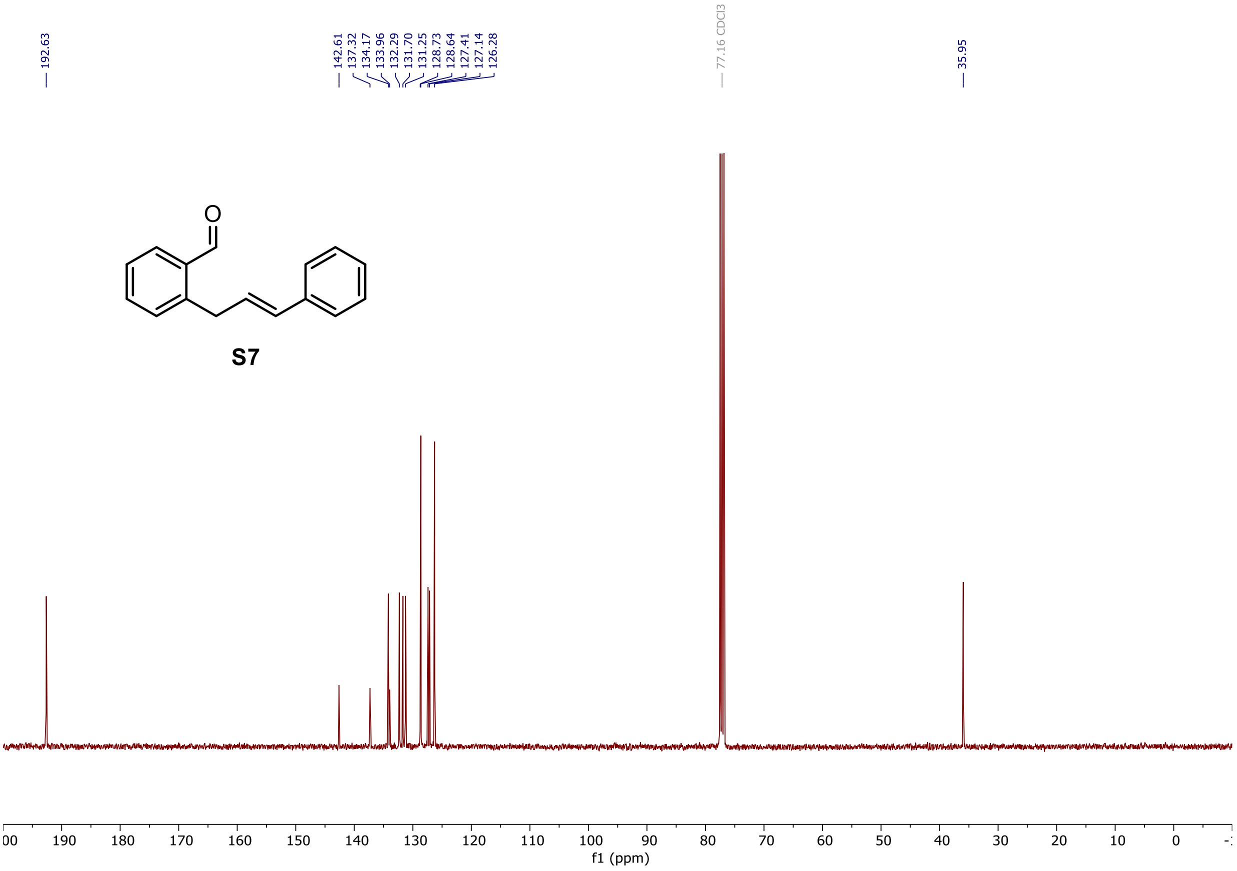

^{1}H NMR (400 MHz, $CDCl_3$) of **S8** (*see procedure*)

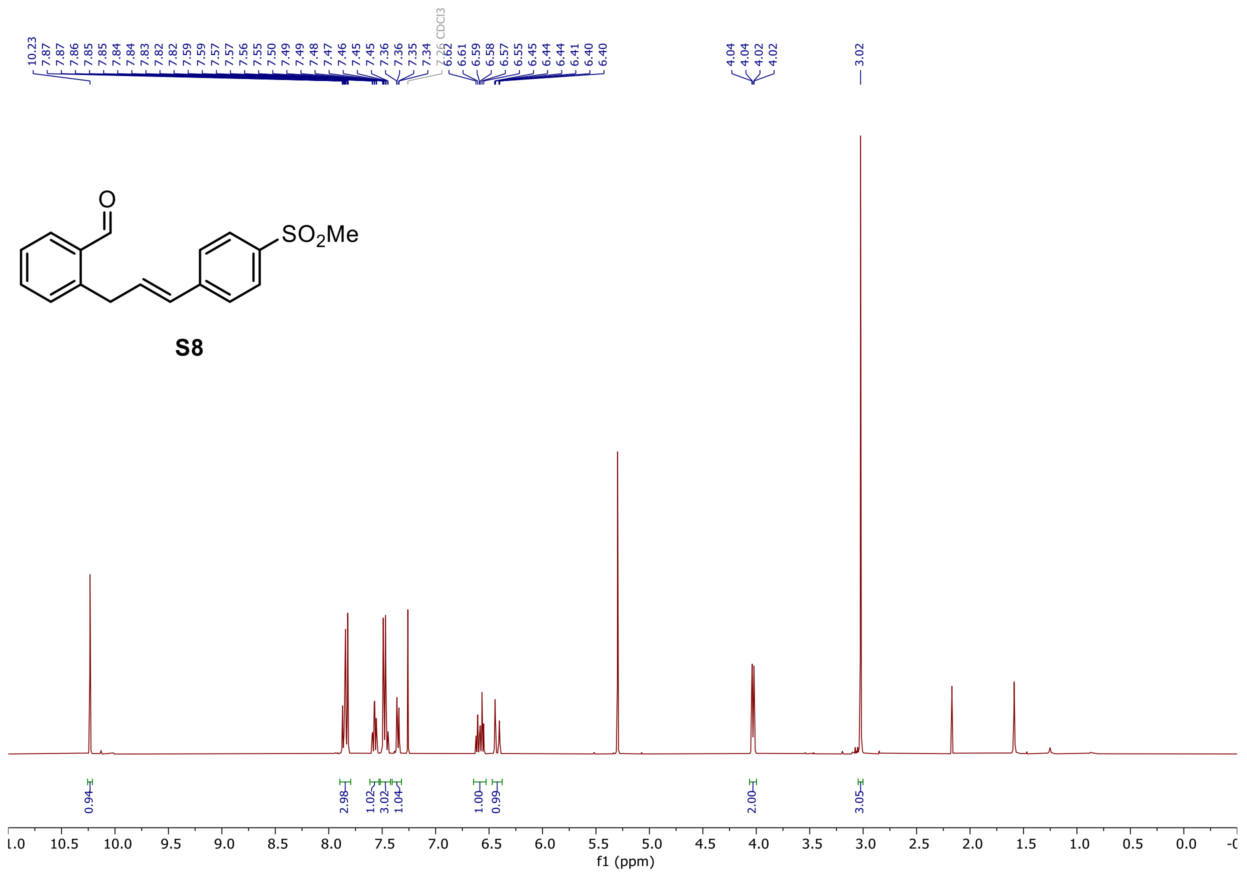

^{13}C NMR (101 MHz, $CDCl_3$) of **S8**

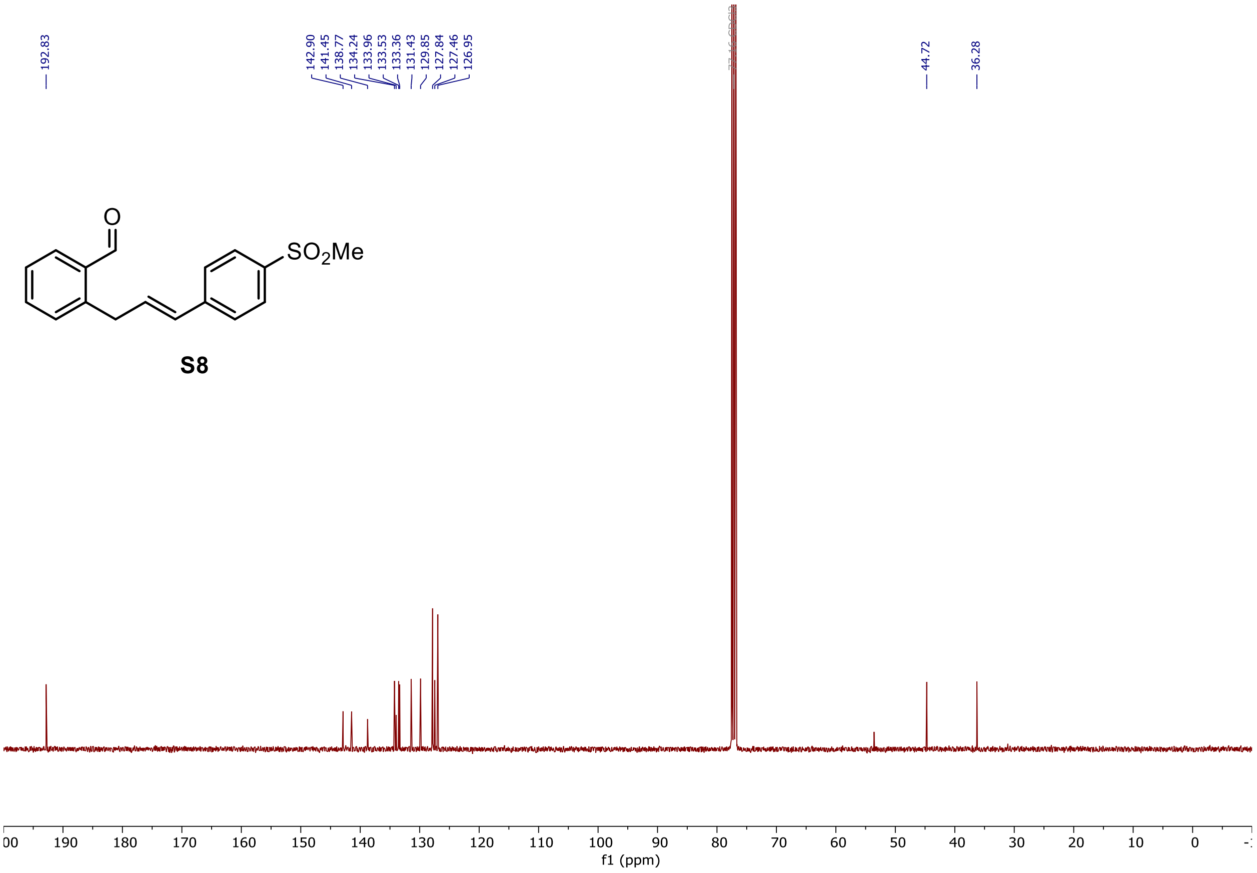

^1H NMR (400 MHz, $CDCl_3$) of **S10** (*see procedure*)

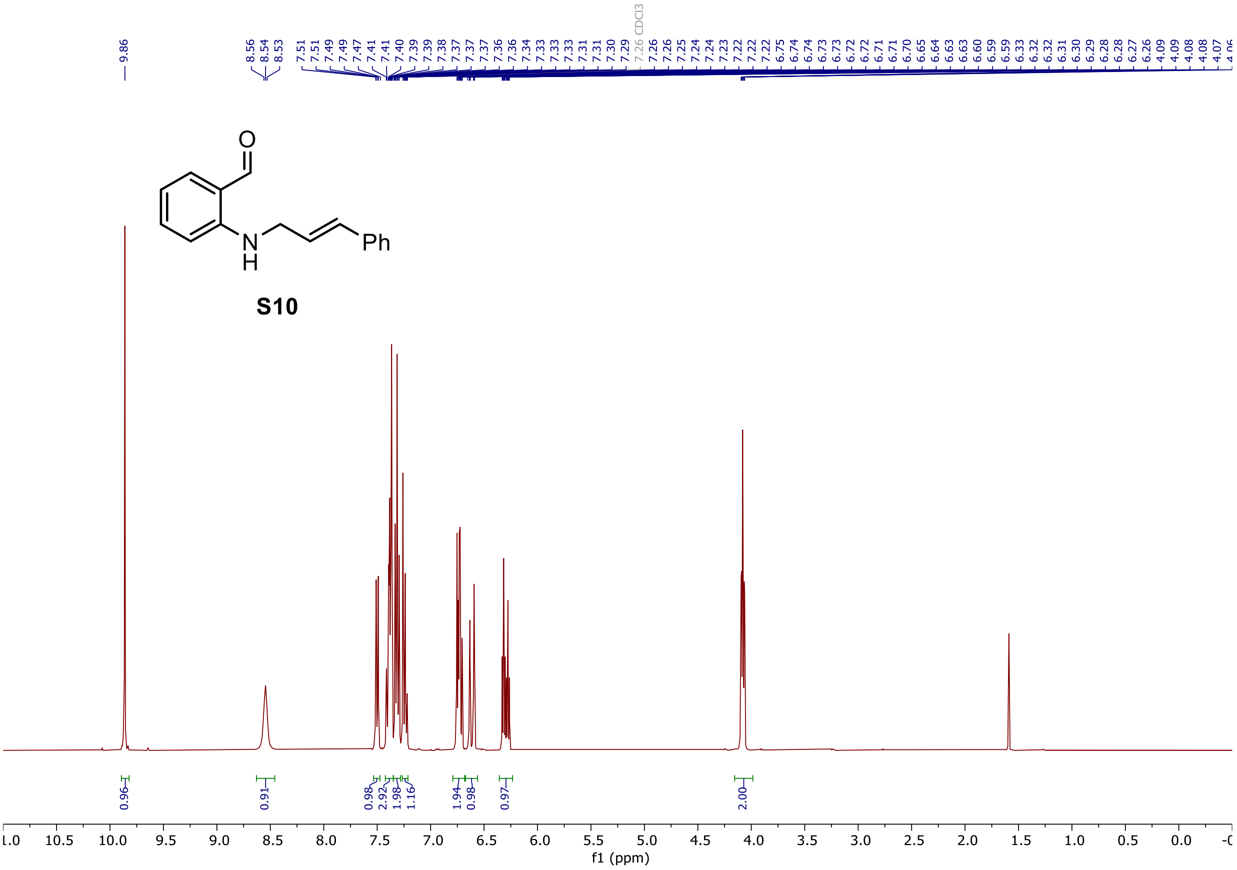

^{13}C NMR (101 MHz, $CDCl_3$) of **S10**

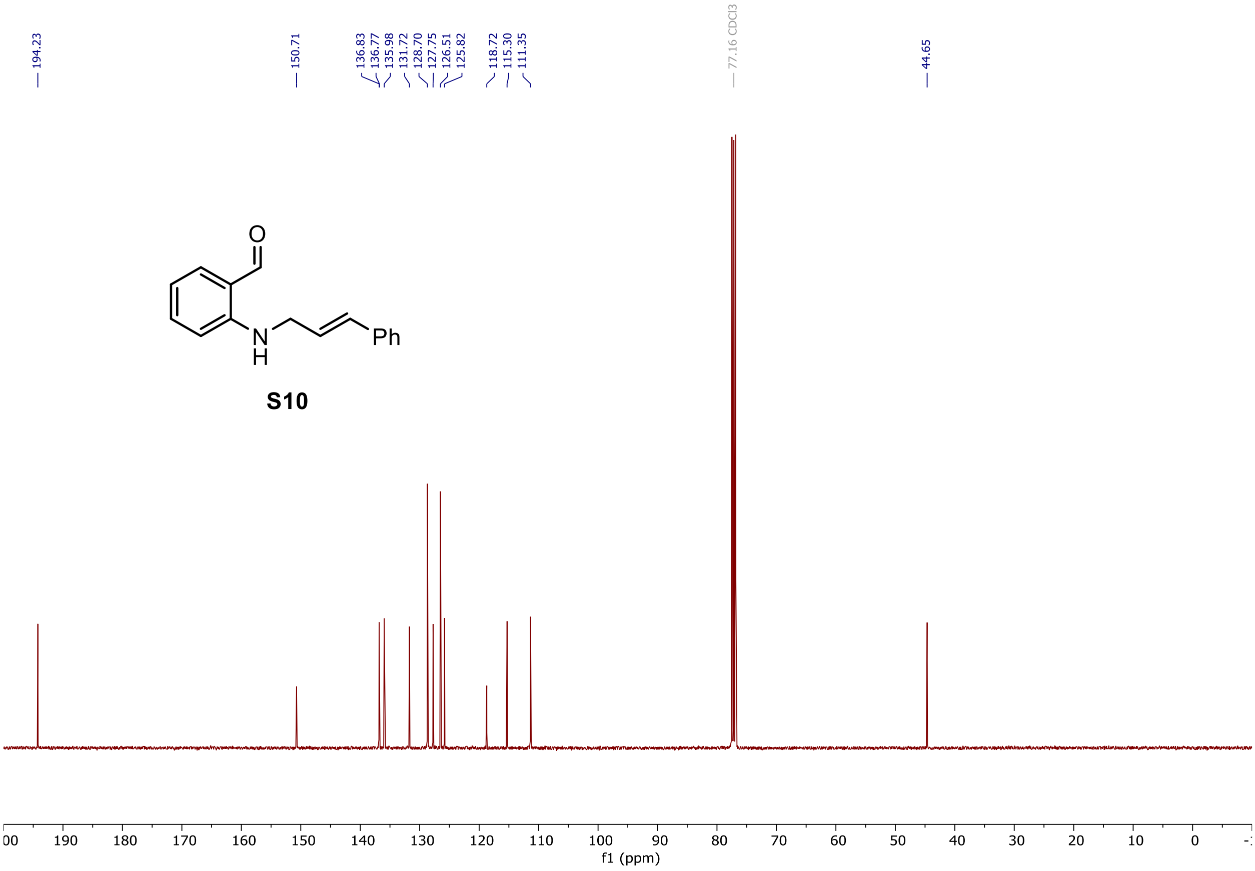

^{1}H NMR (400 MHz, $CDCl_3$) of **6a** (see procedure)

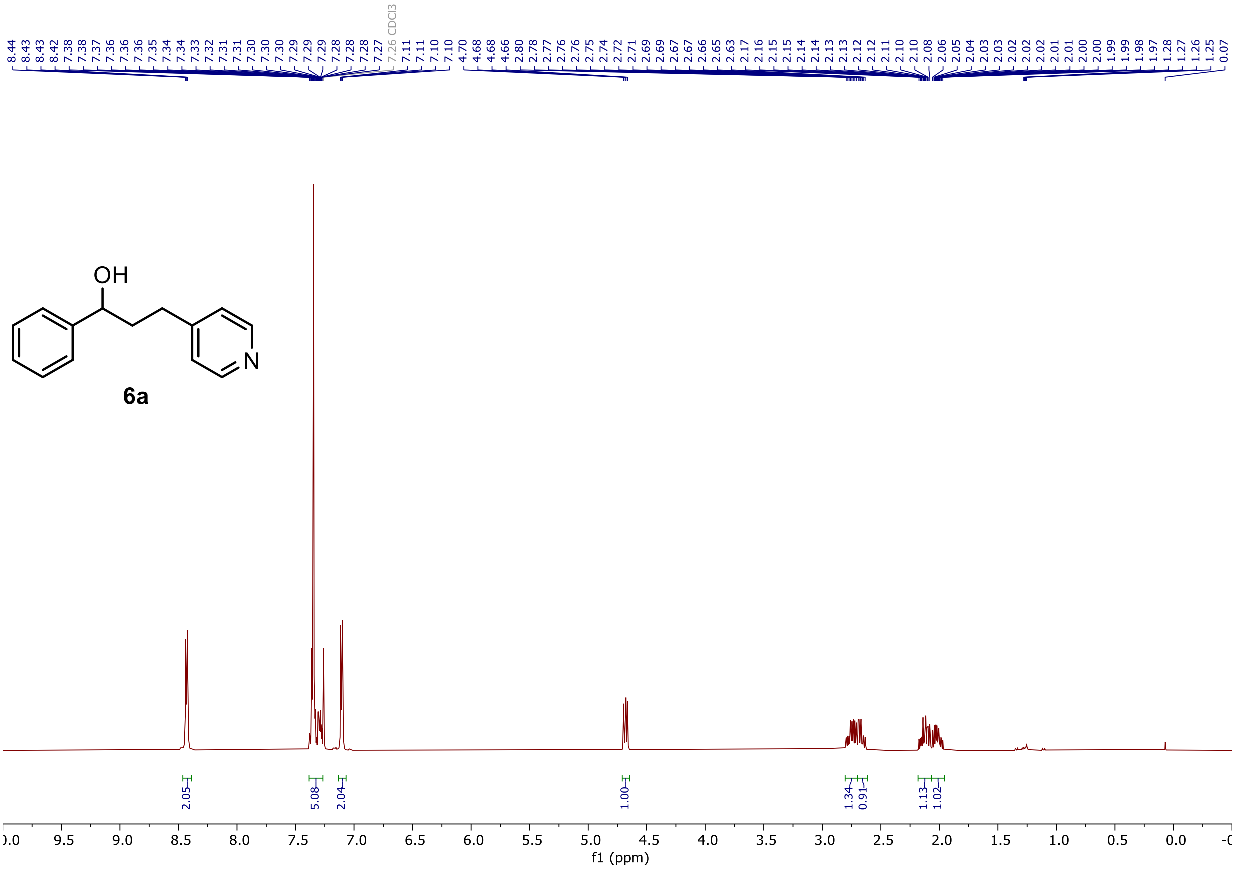

6a

^{13}C NMR (101 MHz, $CDCl_3$) of **6a**

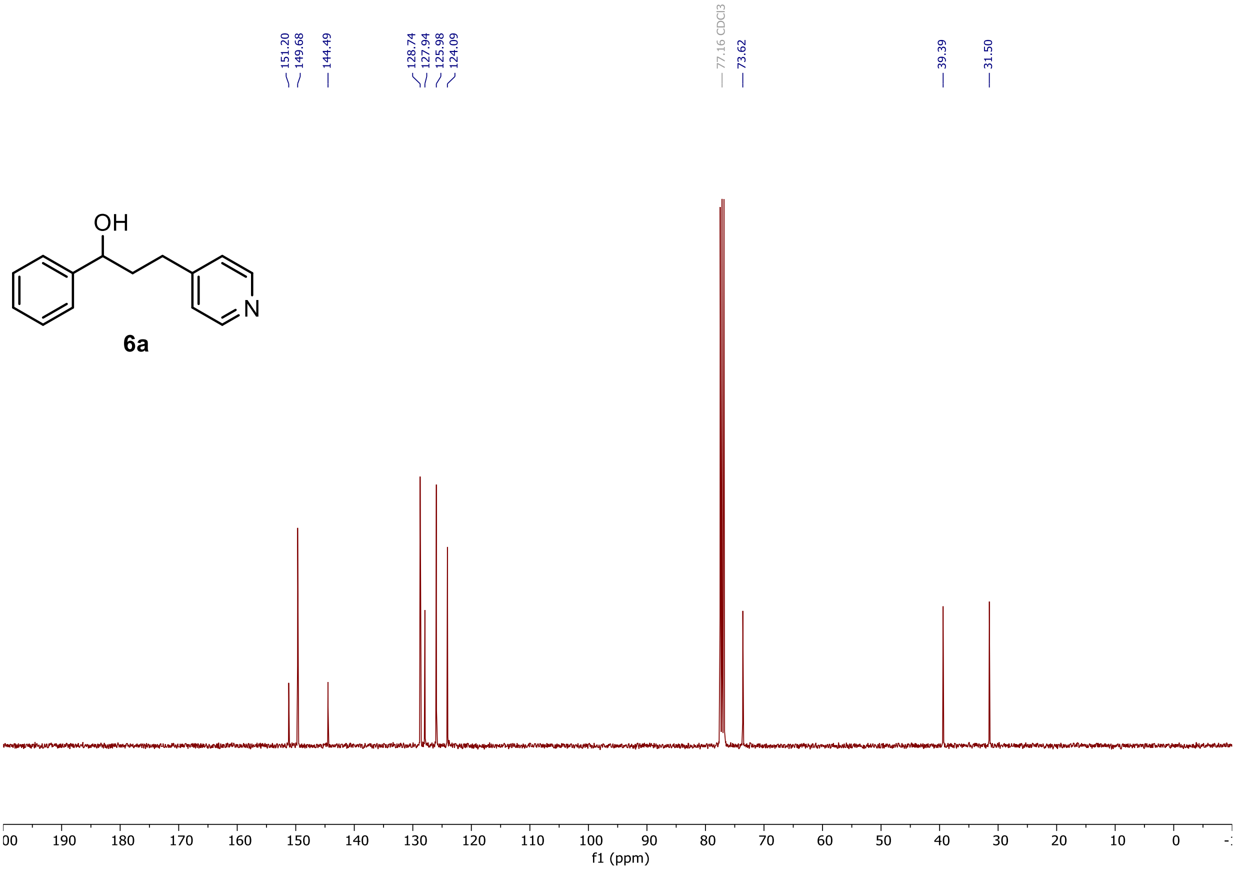

6a

^{1}H NMR (400 MHz, $CDCl_3$) of **7** (see procedure)

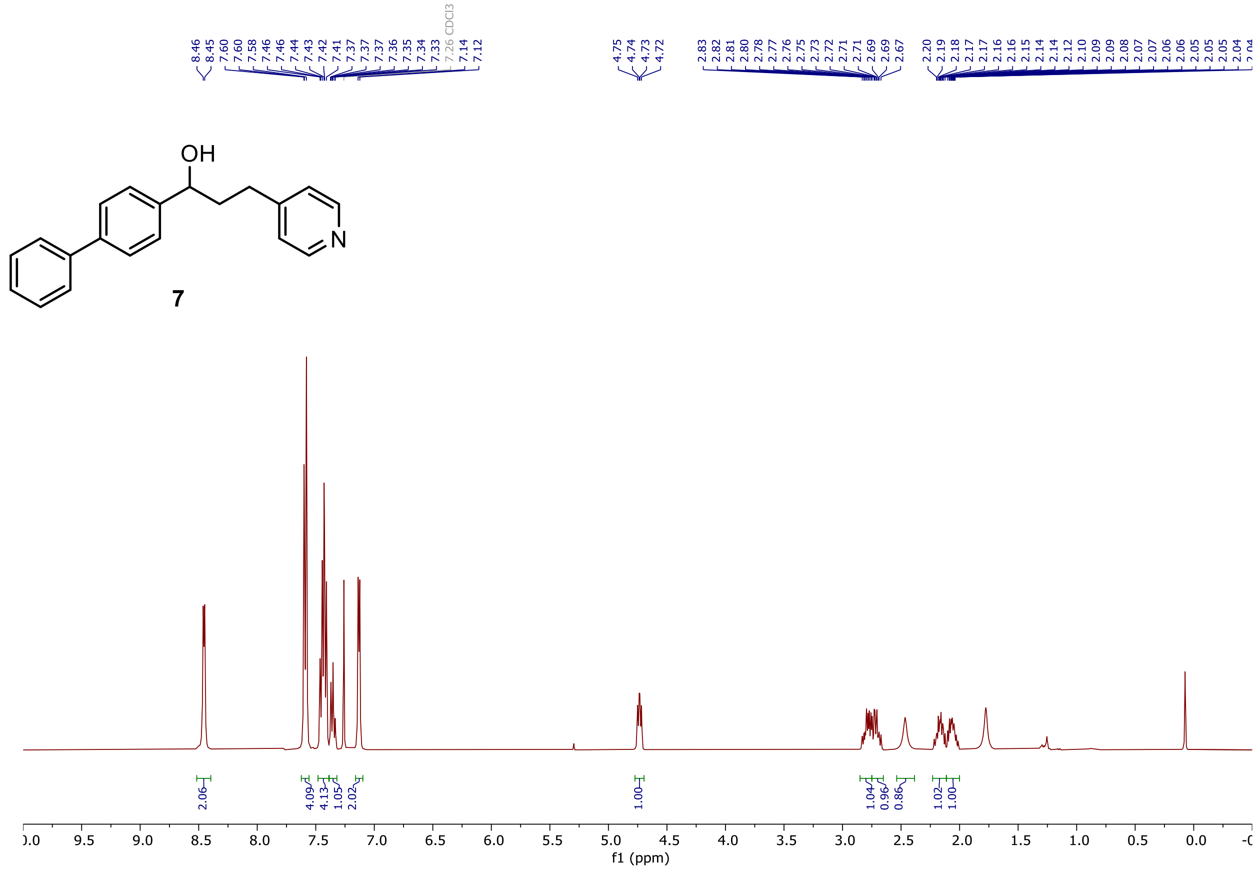

^{13}C NMR (101 MHz, $CDCl_3$) of **7**

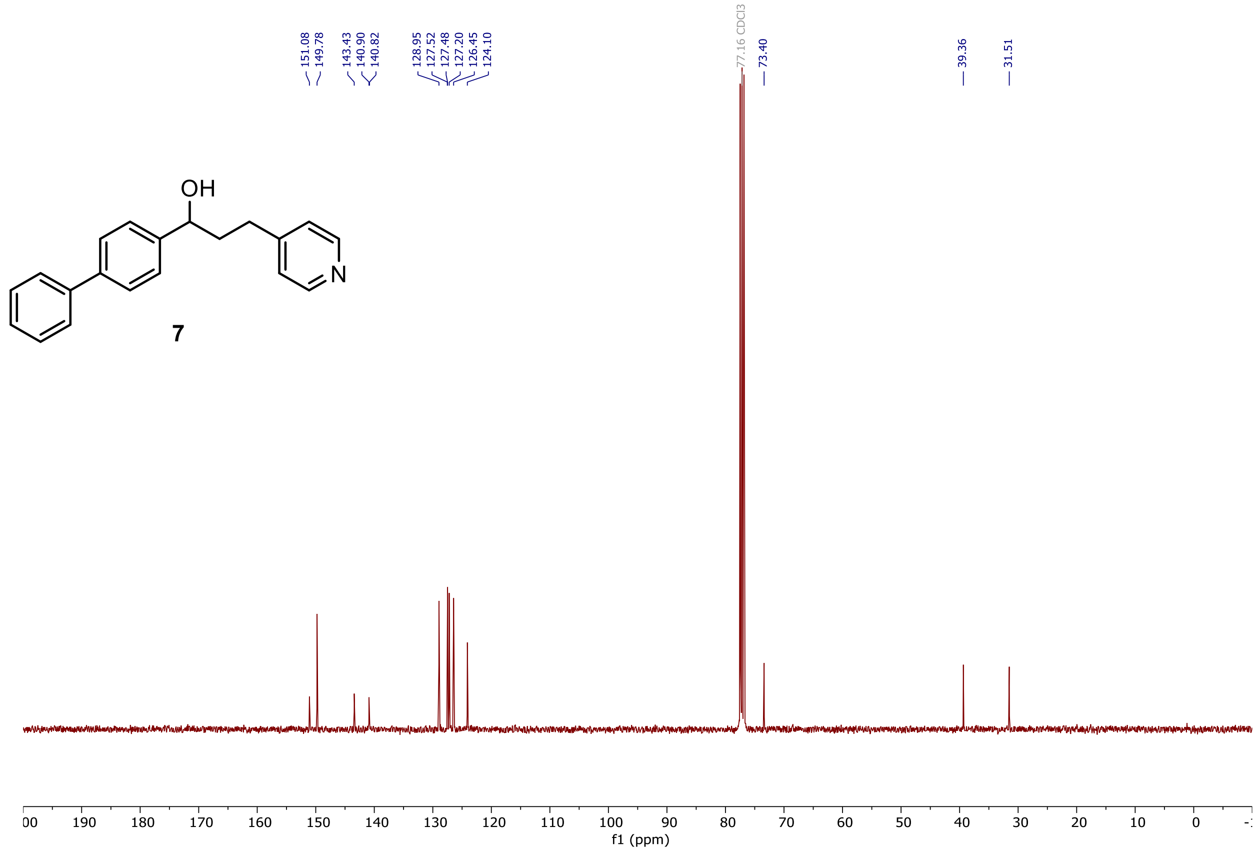

^{1}H NMR (500 MHz, $CDCl_3$) of **8** (*see procedure*)

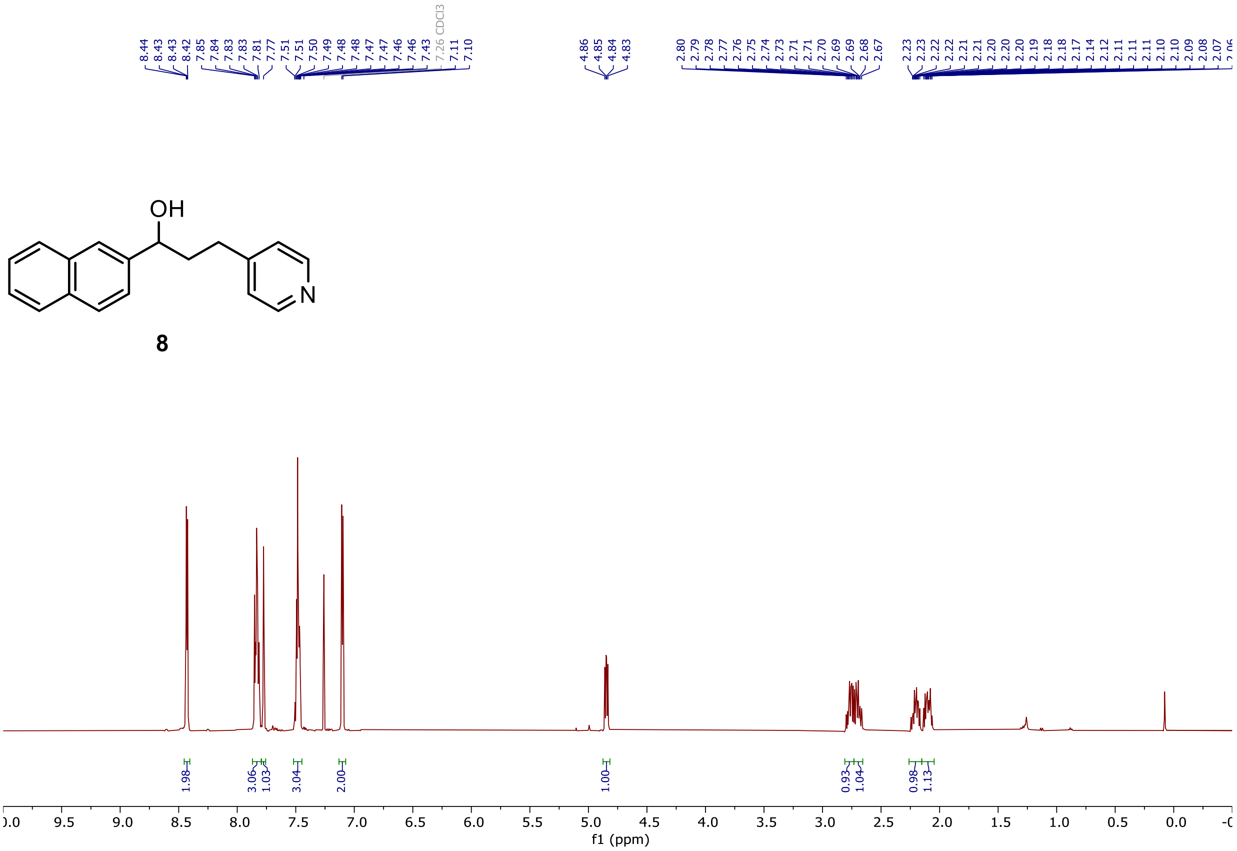

^{13}C NMR (126 MHz, $CDCl_3$) of **8**

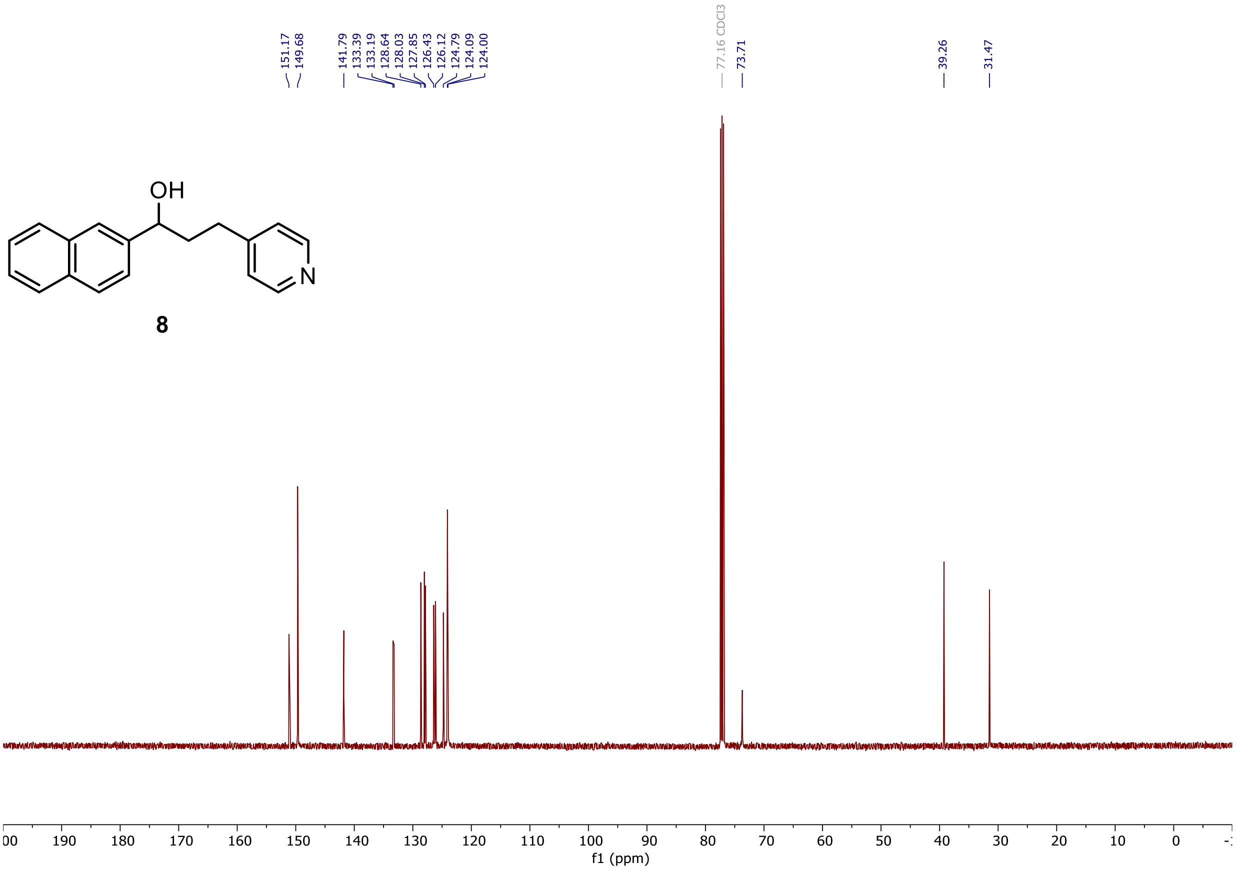

^{1}H NMR (400 MHz, $CDCl_3$) of **9** (<u>*see procedure*</u>)

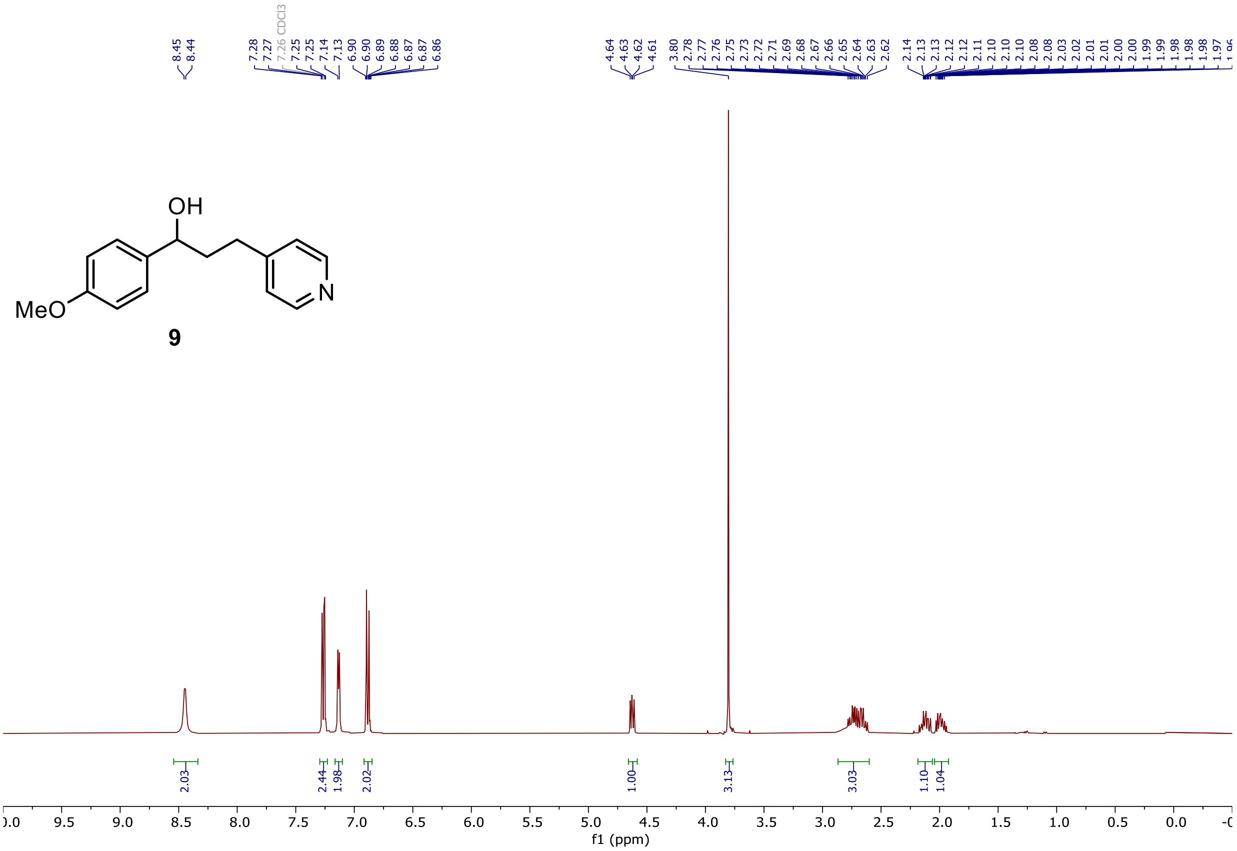

^{13}C NMR (101 MHz, $CDCl_3$) of **9**

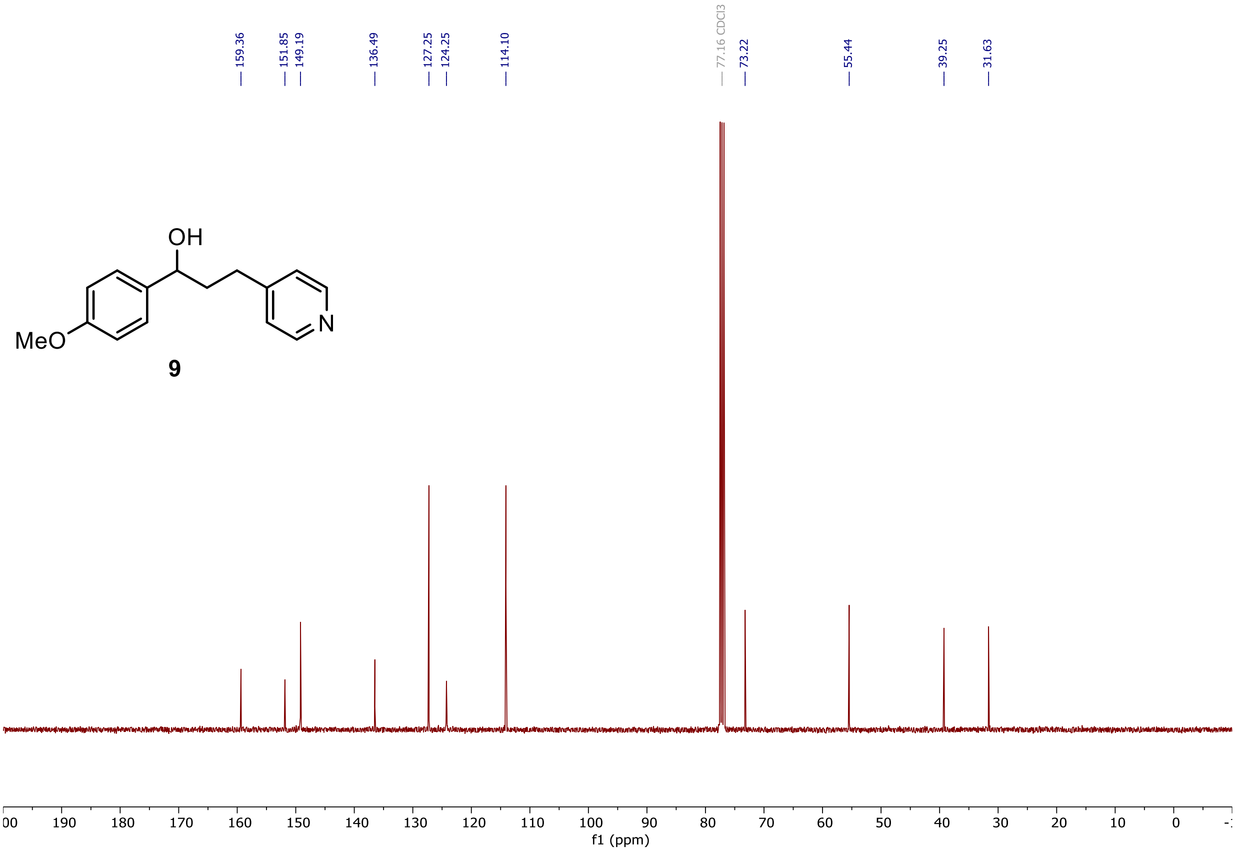

^{1}H NMR (400 MHz, $CDCl_3$) of **10** (see procedure)

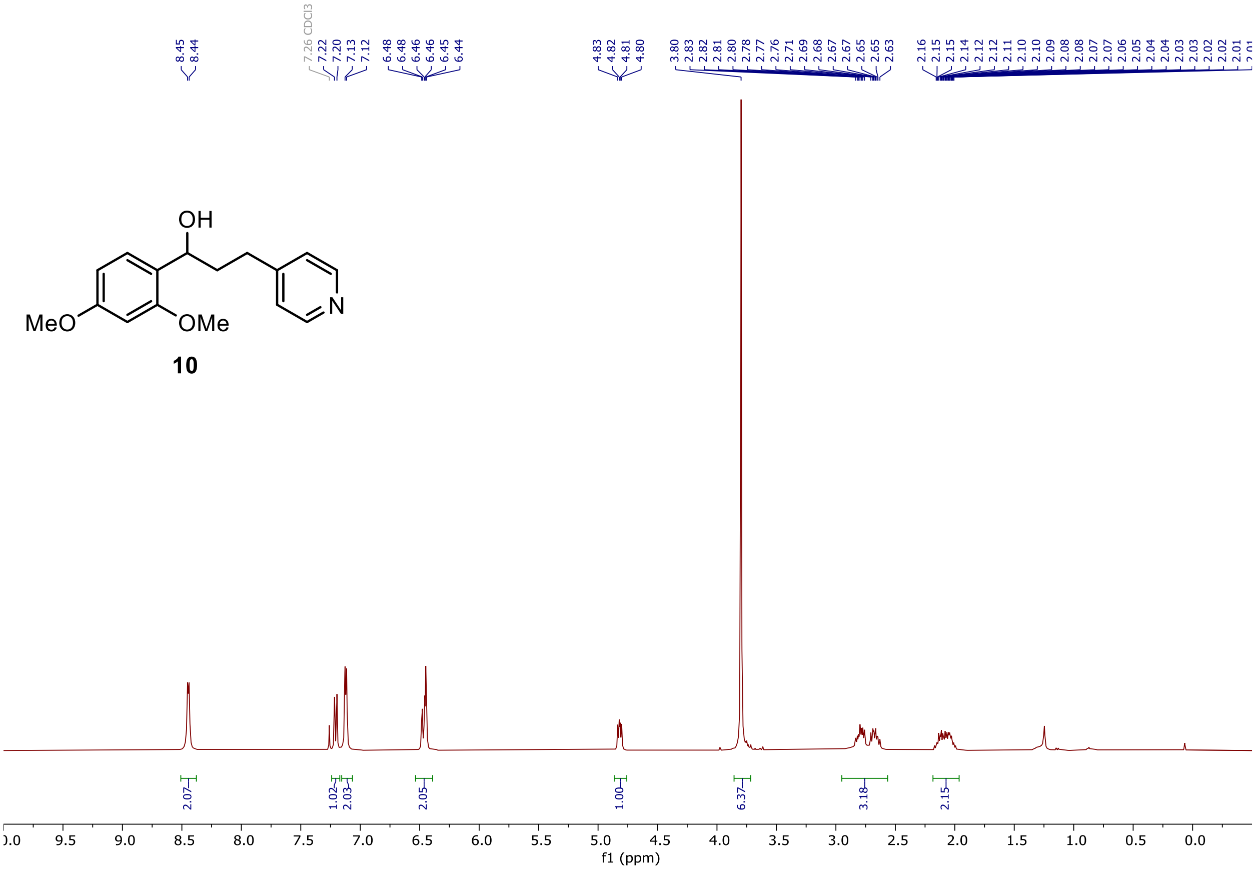

^{13}C NMR (101 MHz, $CDCl_3$) of **10**

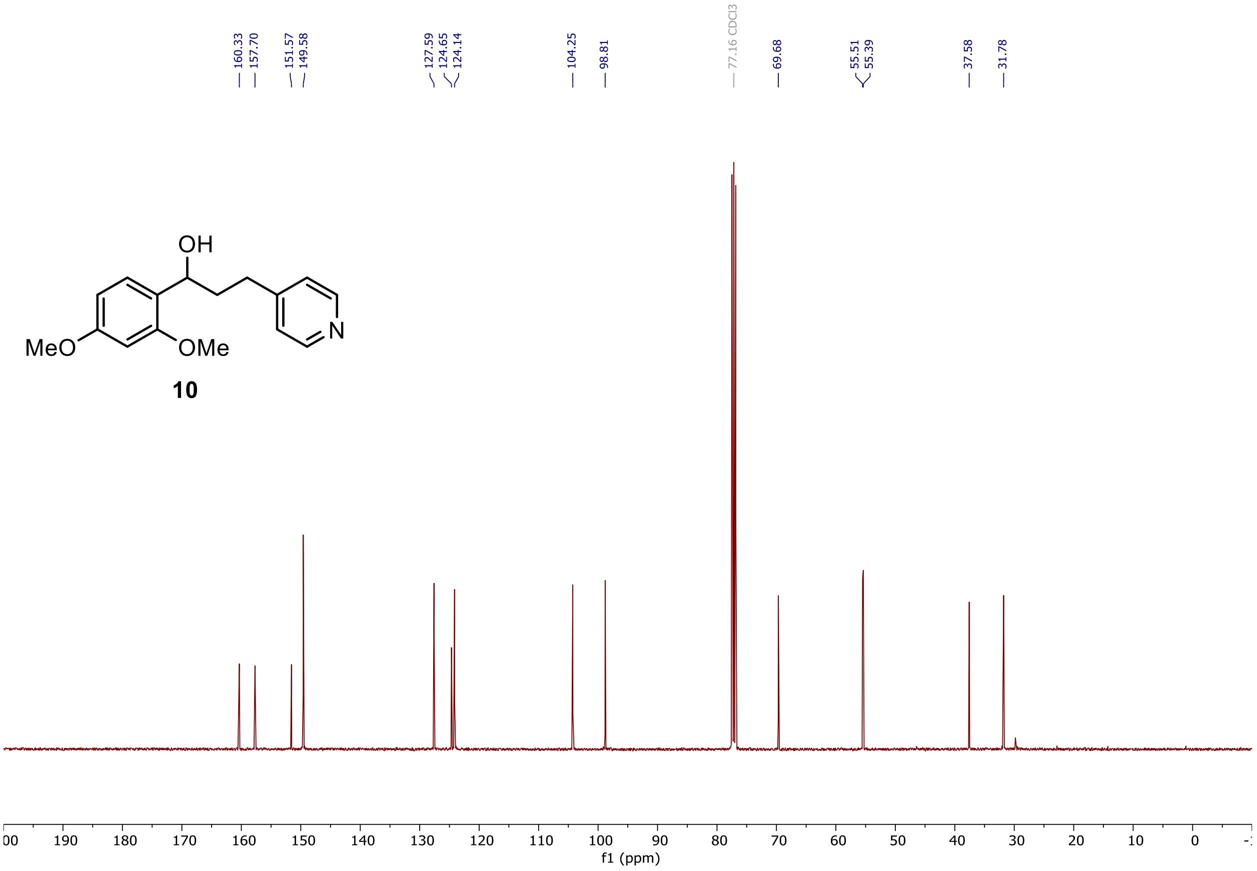

^{1}H NMR (400 MHz, $CDCl_3$) of **11** (*see procedure*)

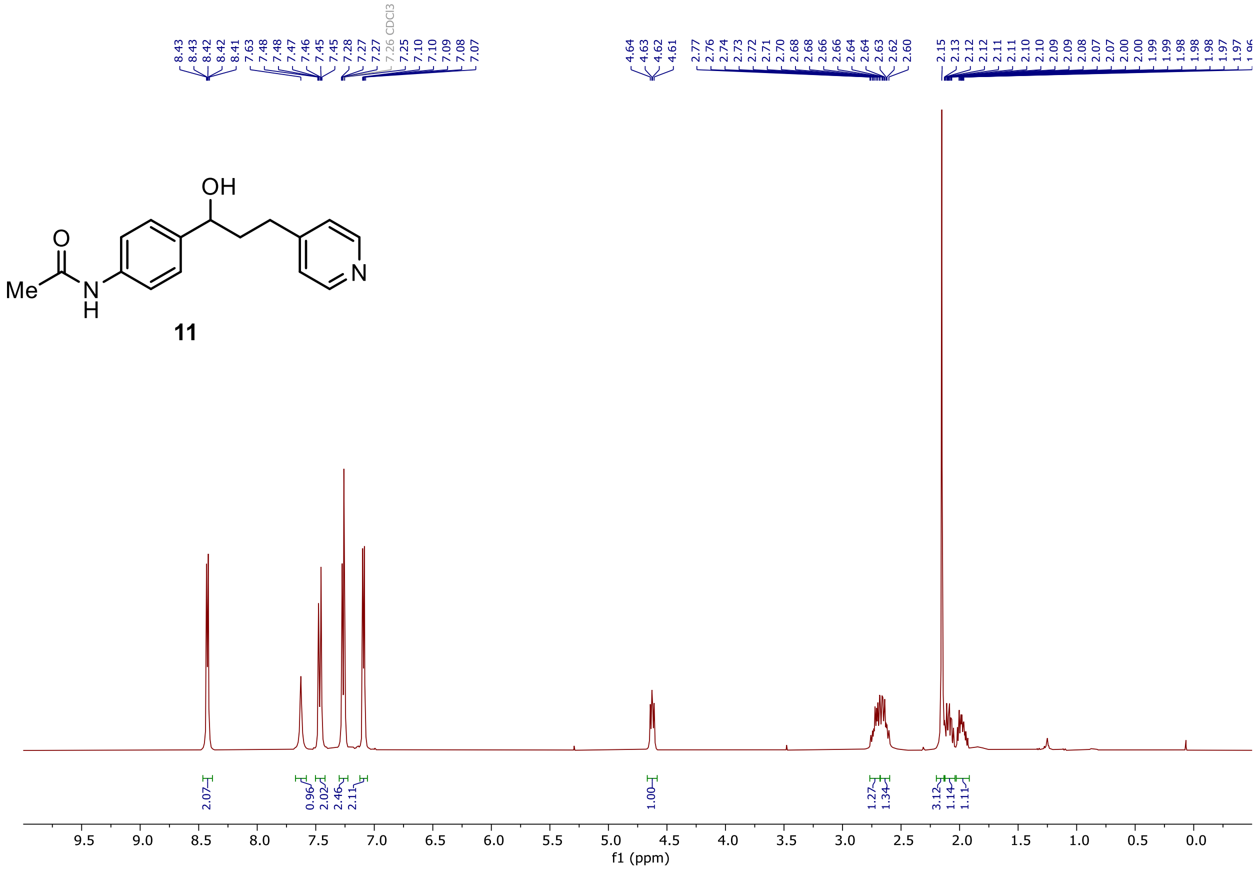

^{13}C NMR (101 MHz, $CDCl_3$) of **11**

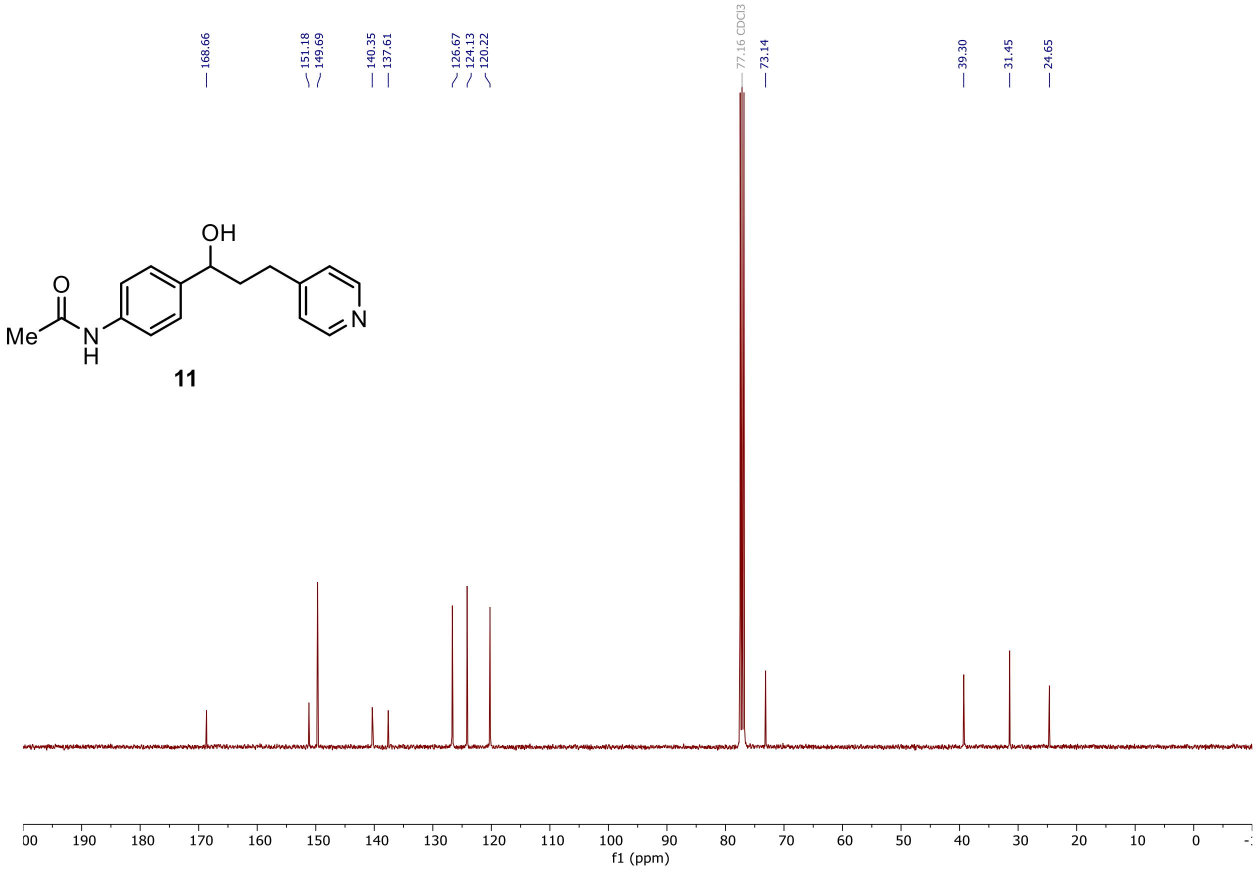

^{1}H NMR (400 MHz, $CDCl_3$) of **12** (see procedure)

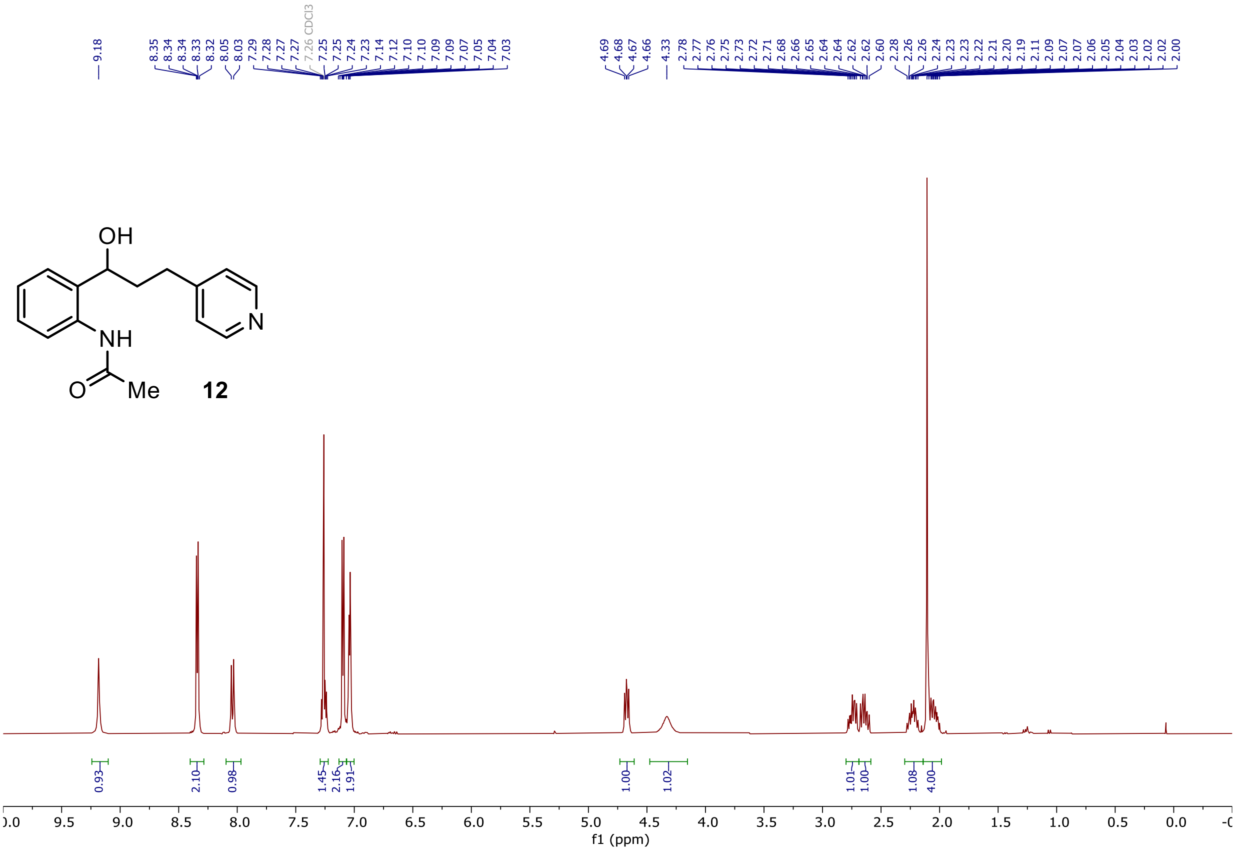

^{13}C NMR (126 MHz, $CDCl_3$) of **12**

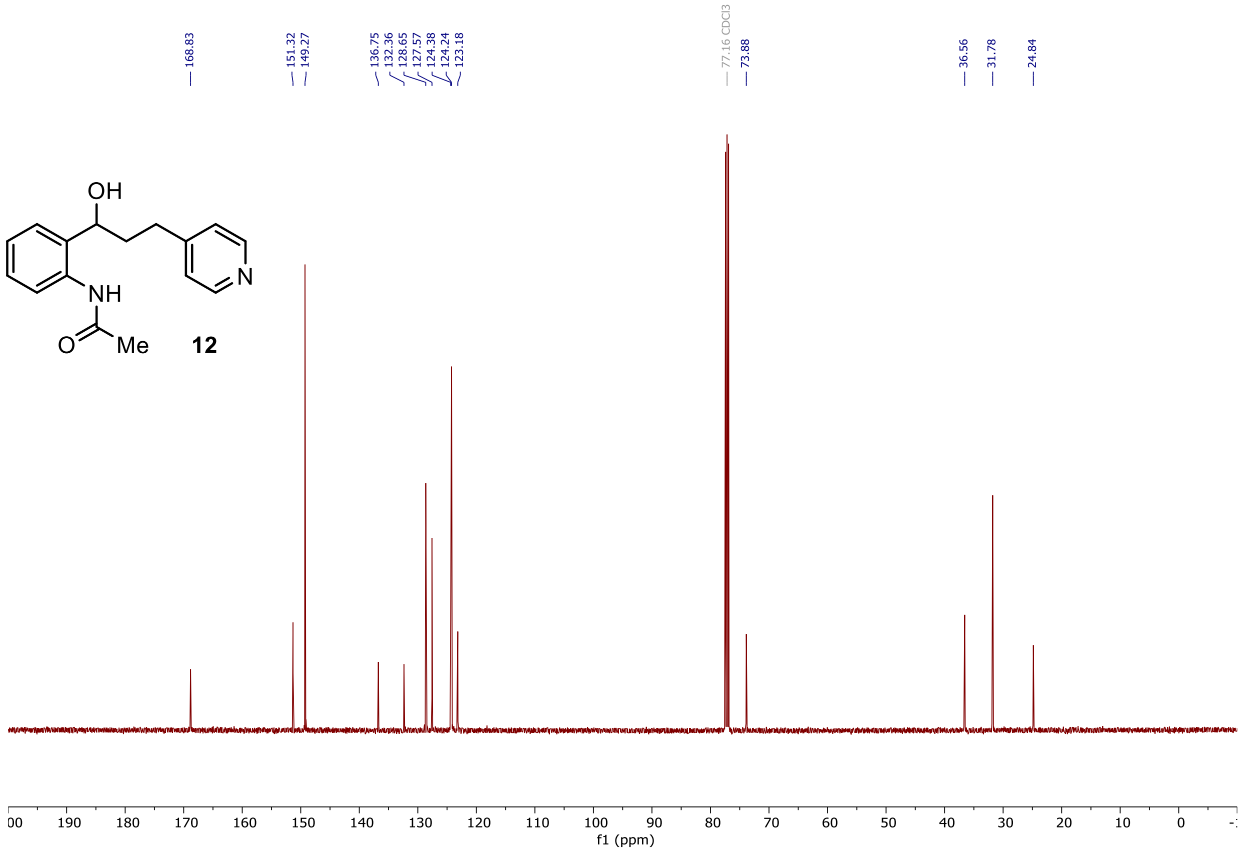

^{1}H NMR (400 MHz, $CDCl_3$) of **13** (see procedure)

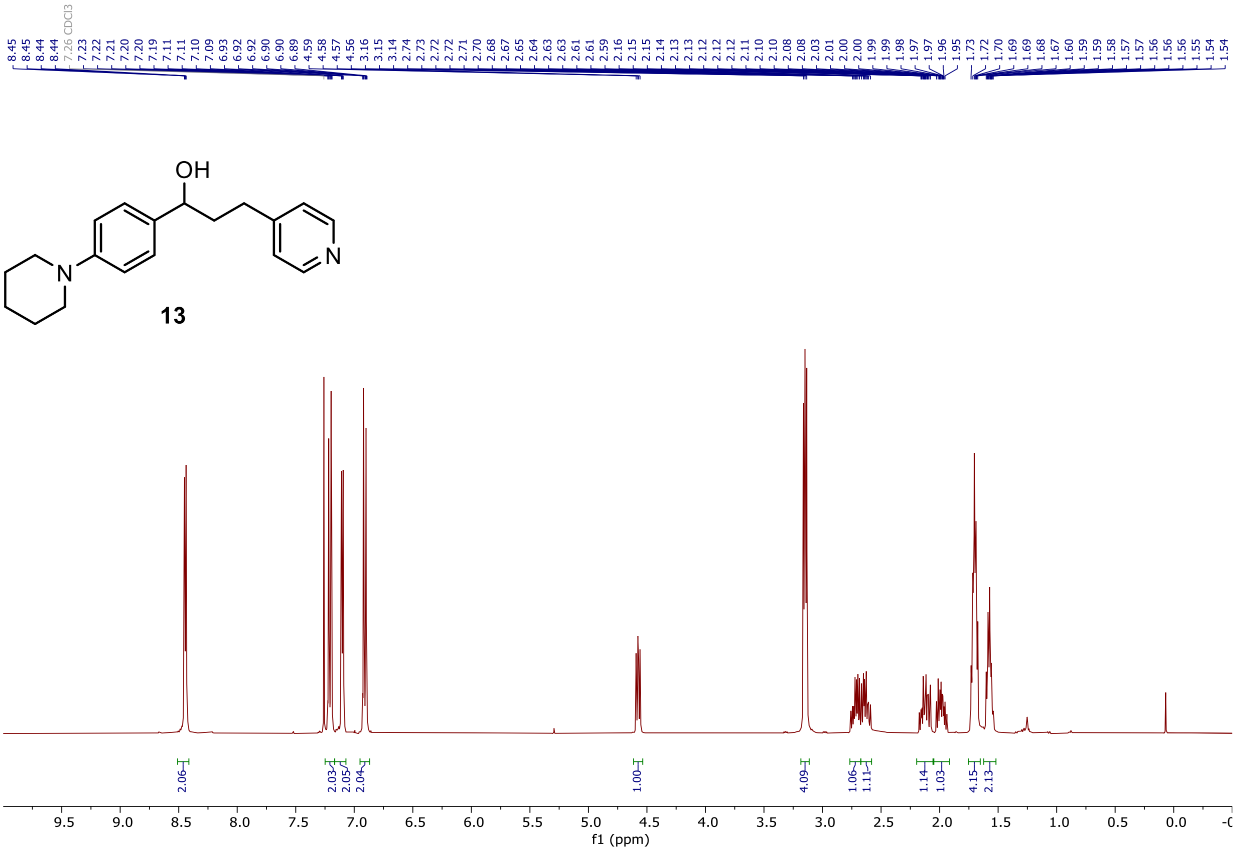

^{13}C NMR (101 MHz, $CDCl_3$) of **13**

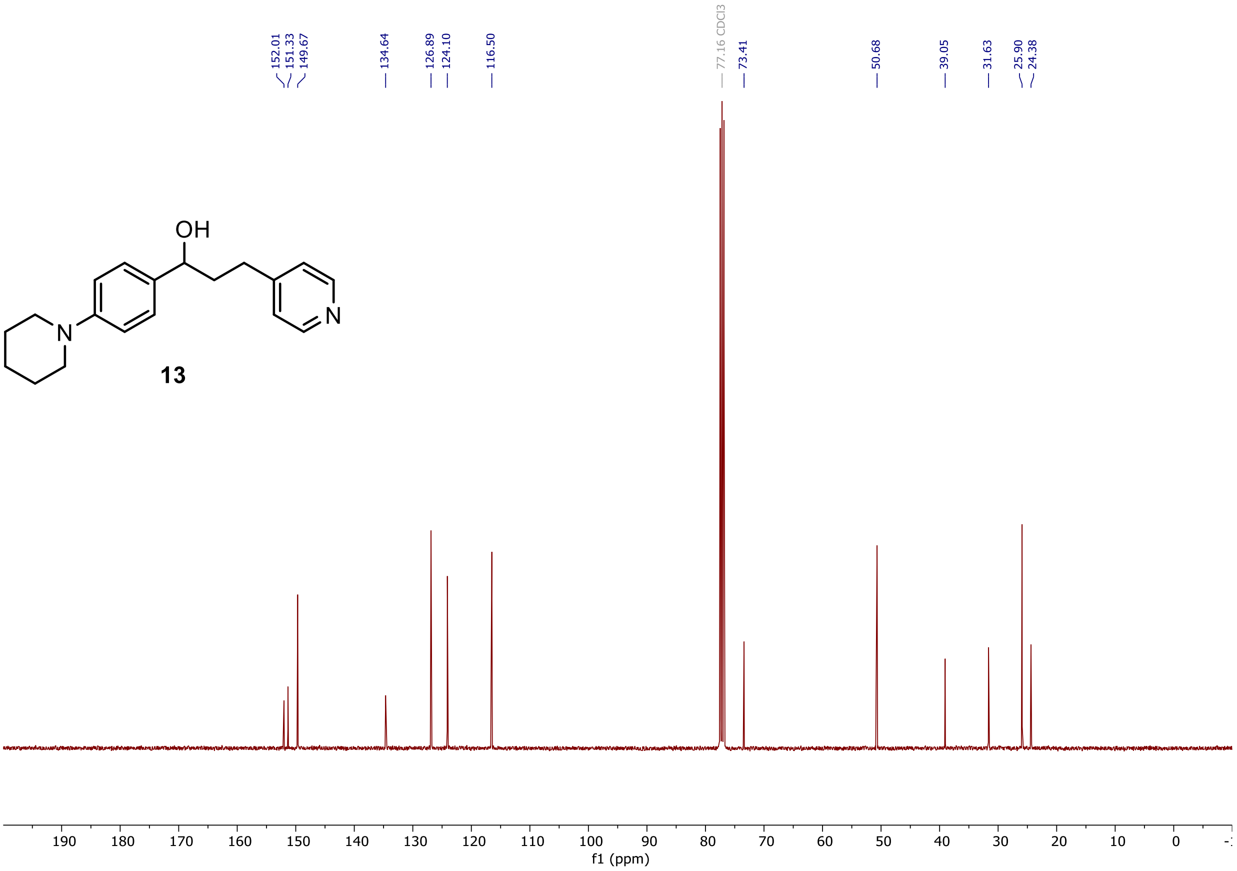

^{1}H NMR (400 MHz, $CDCl_3$) of **14** (*see procedure*)

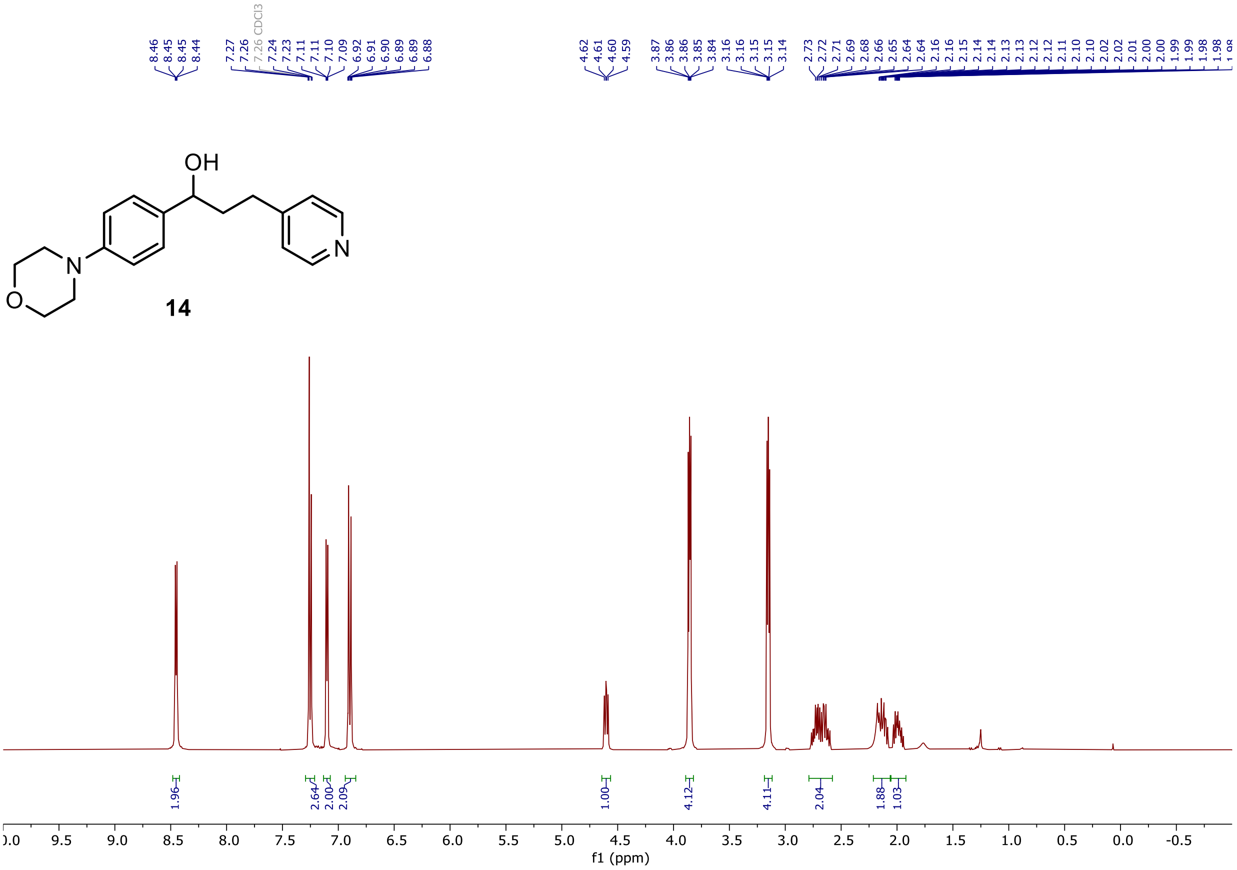

^{13}C NMR (101 MHz, $CDCl_3$) of **14**

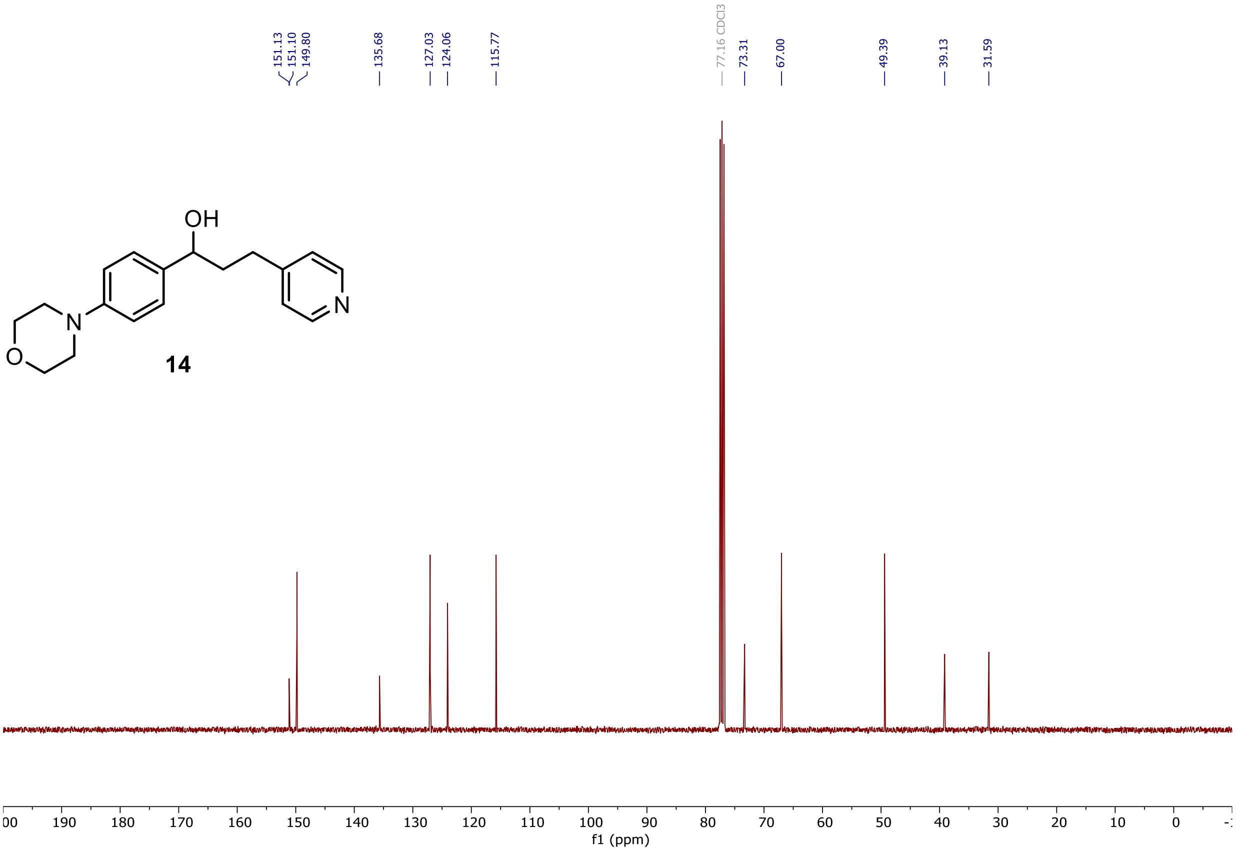

^{1}H NMR (500 MHz, $CDCl_3$) of **15** (*see procedure*)

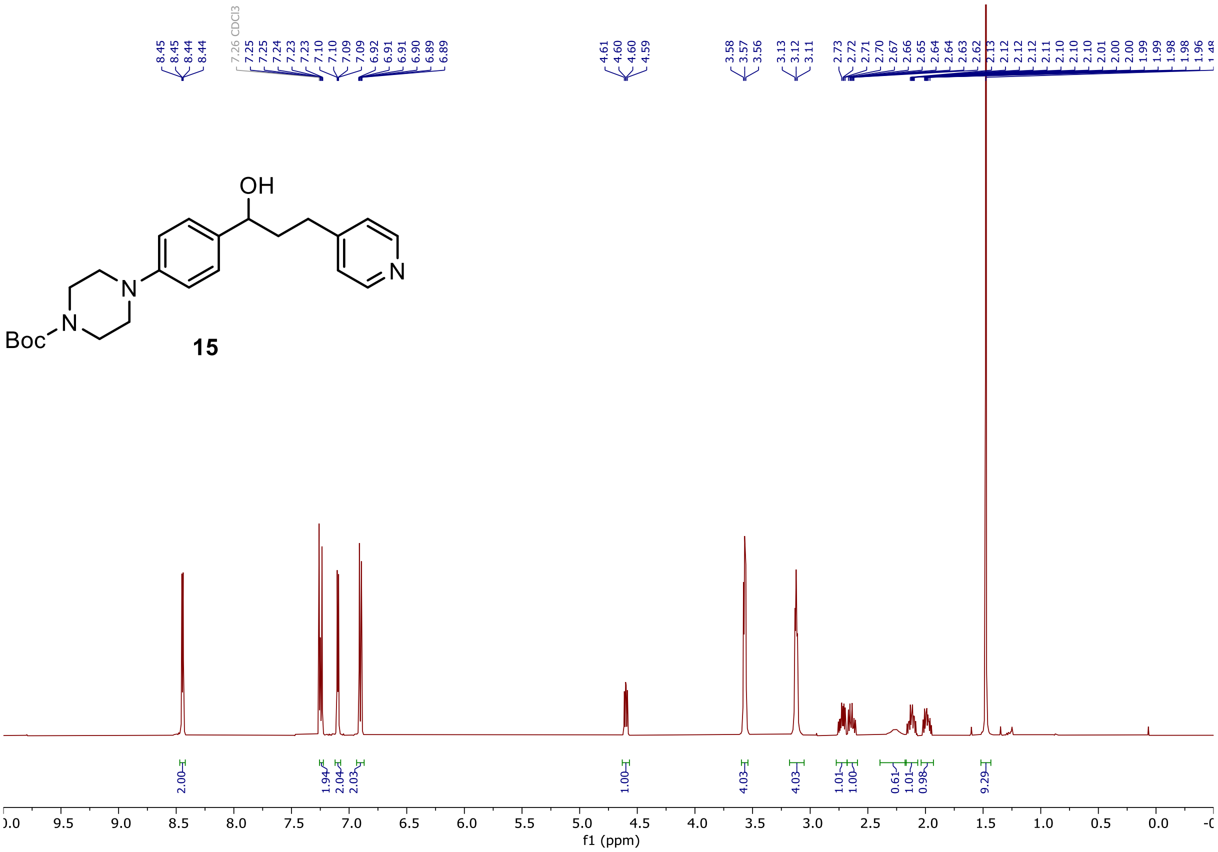

^{13}C NMR (126 MHz, $CDCl_3$) of **15**

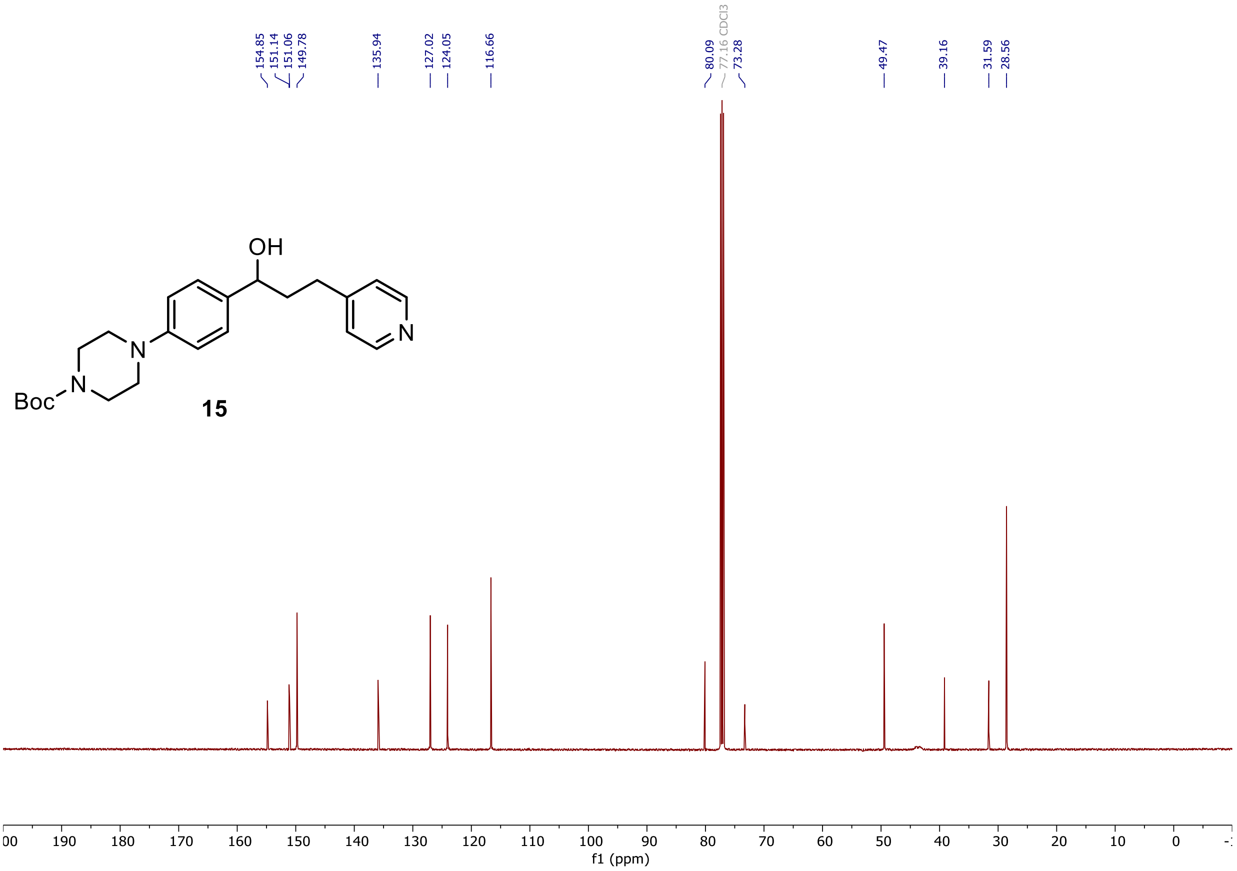

^{1}H NMR (400 MHz, $CDCl_3$) of **16** (*see procedure*)

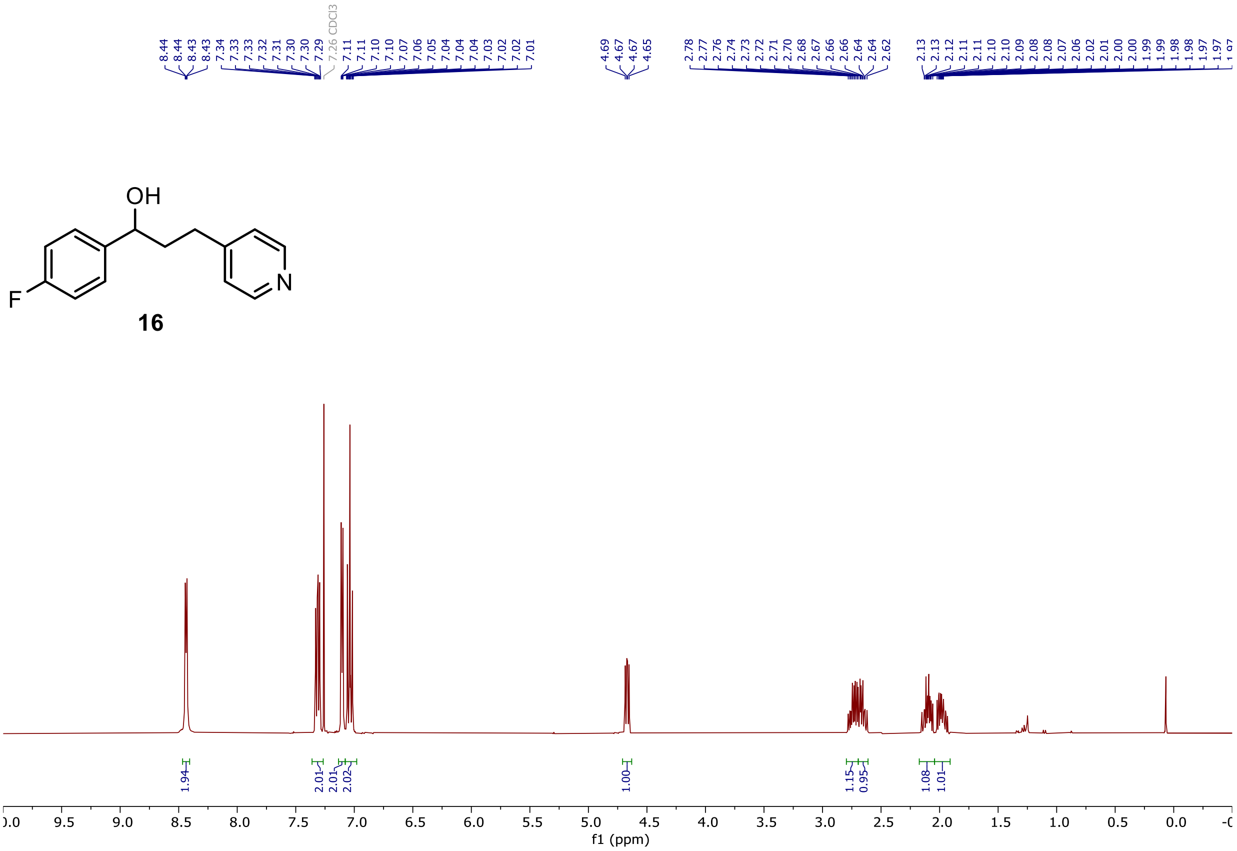

^{13}C NMR (101 MHz, $CDCl_3$) of **16**

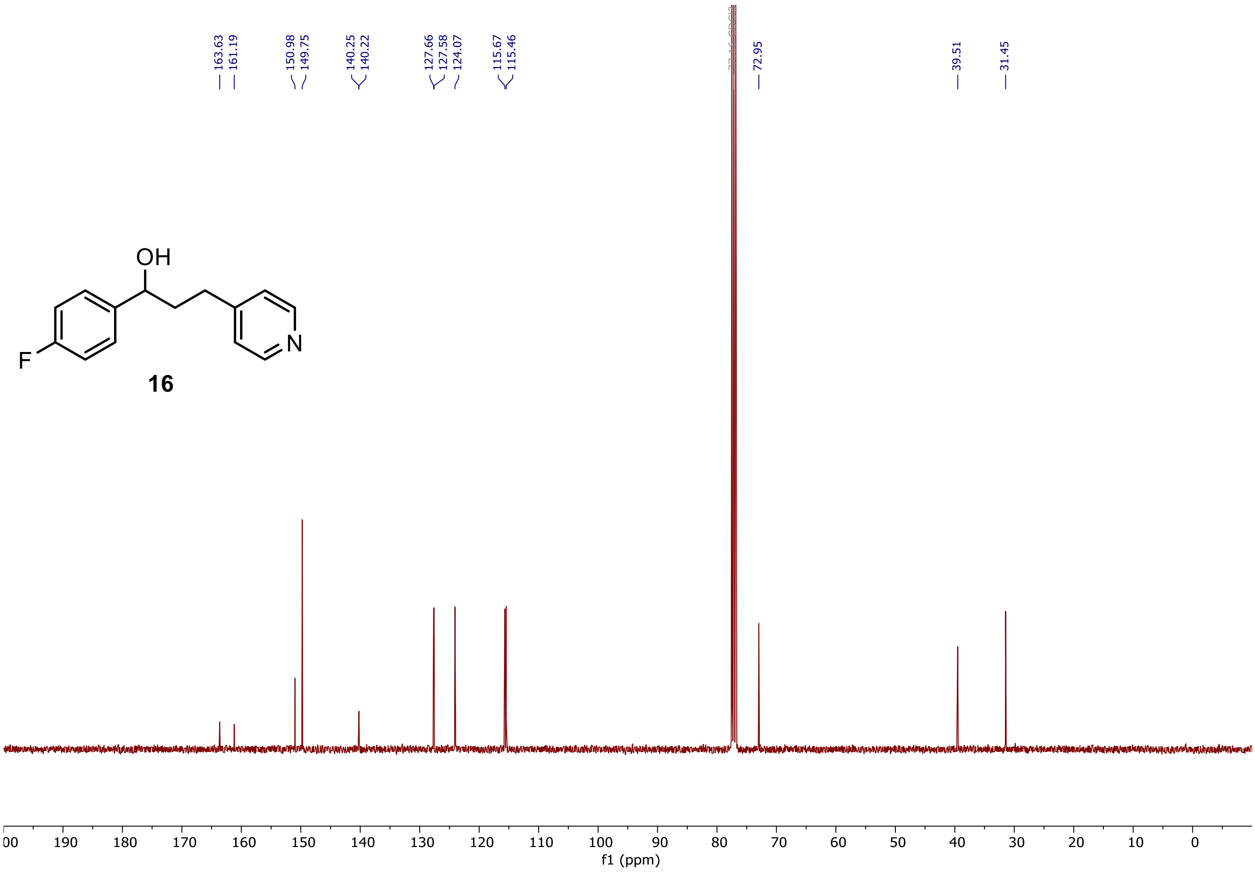

^{19}F NMR (377 MHz, $CDCl_3$) of **16**

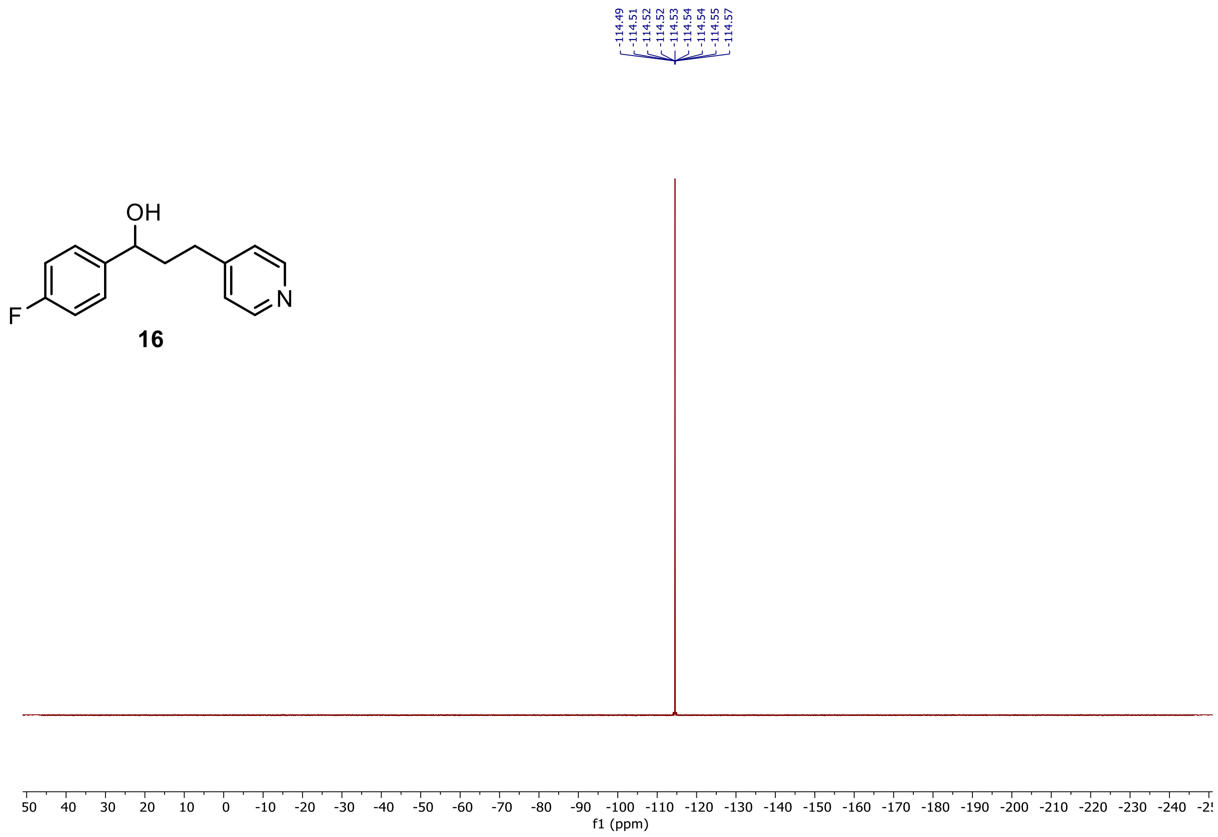

16

^{1}H NMR (400 MHz, $CDCl_3$) of **17** (*see procedure*)

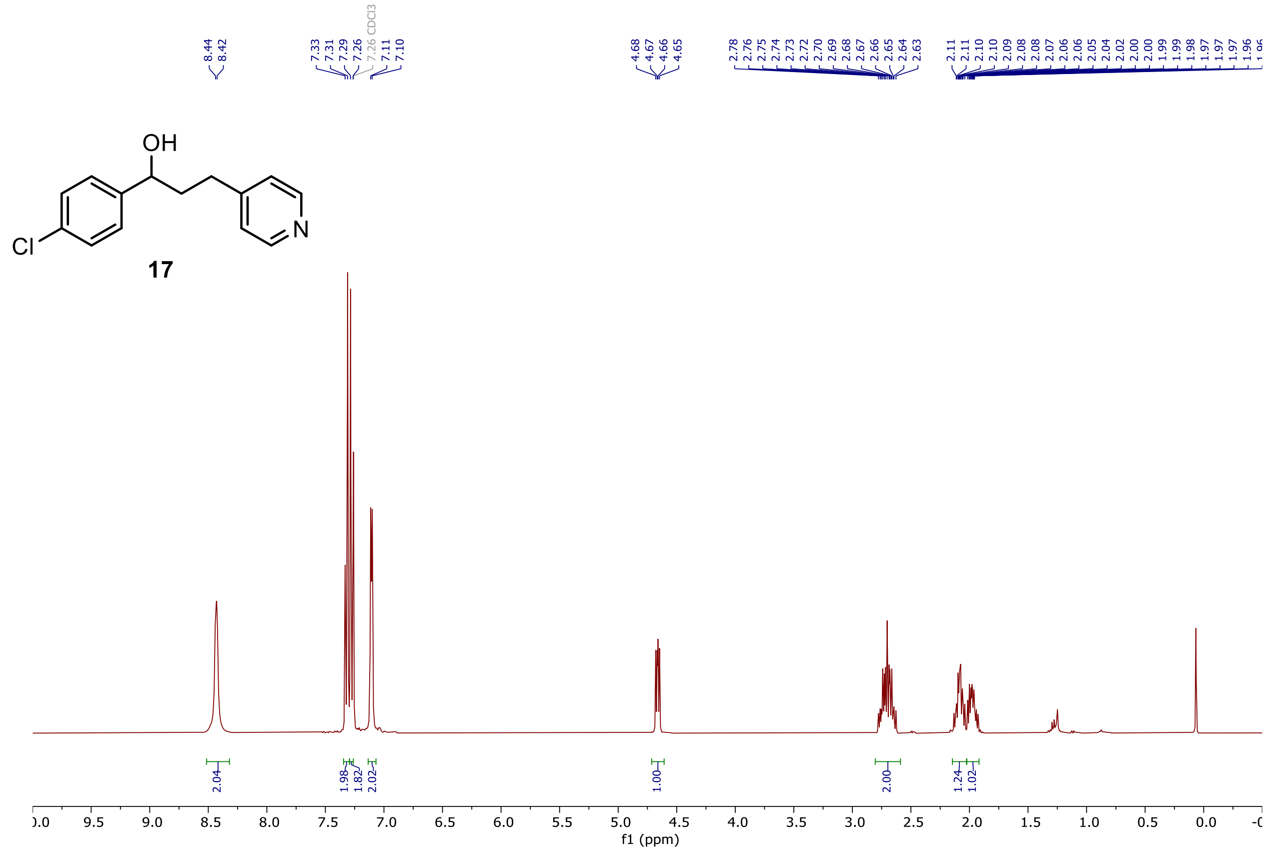

^{13}C NMR (101 MHz, $CDCl_3$) of **17**

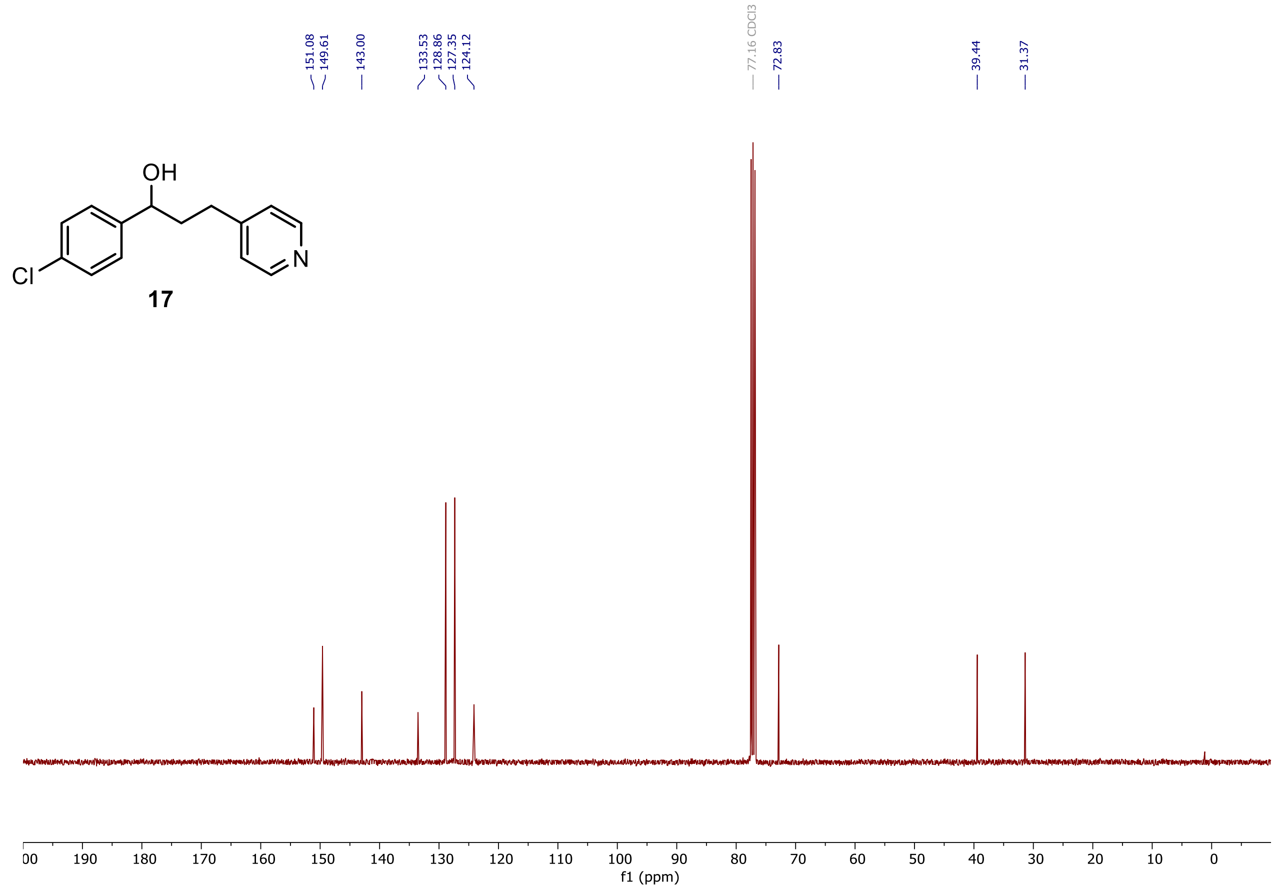

^{1}H NMR (400 MHz, $CDCl_3$) of **18** (see procedure)

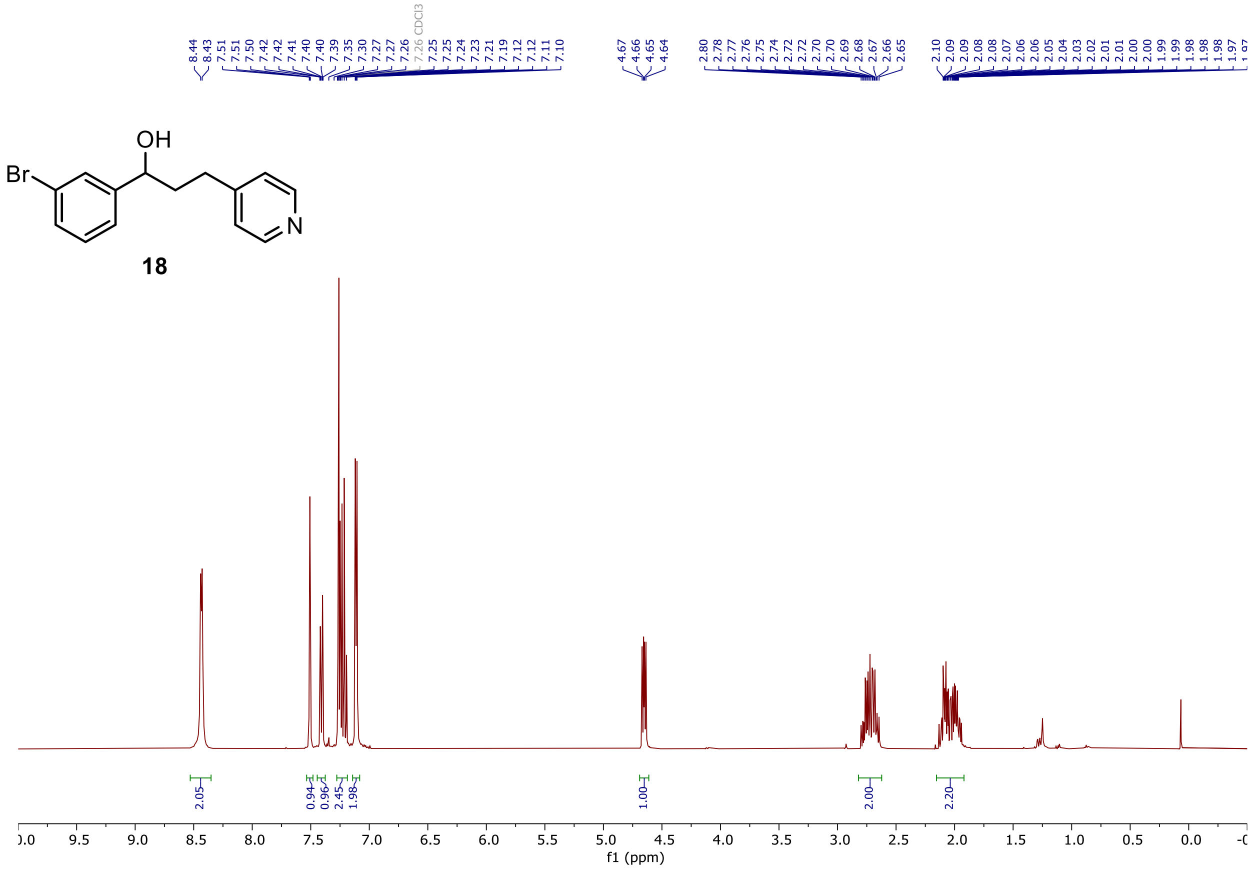

^{13}C NMR (101 MHz, $CDCl_3$) of **18**

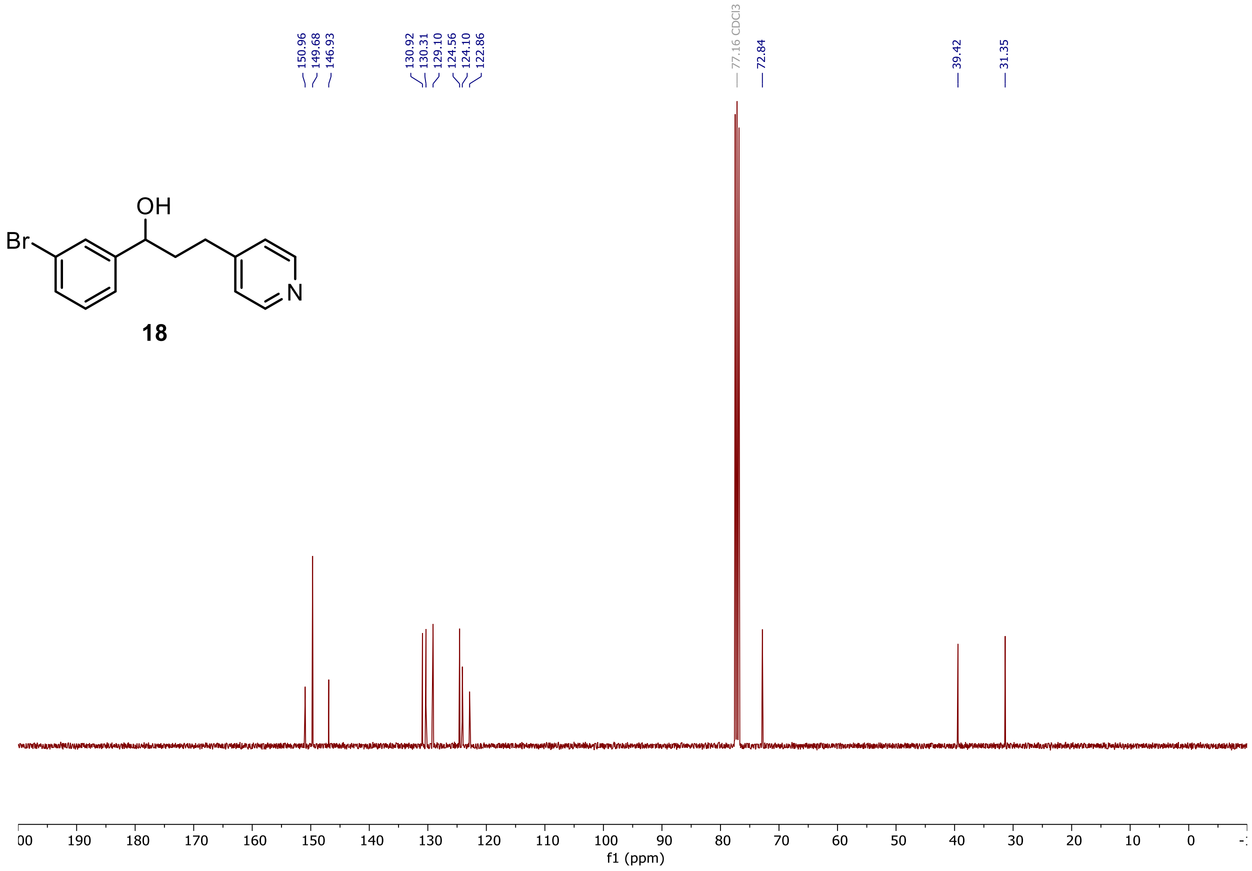

^{1}H NMR (400 MHz, $CDCl_3$) of **19** (*see procedure*)

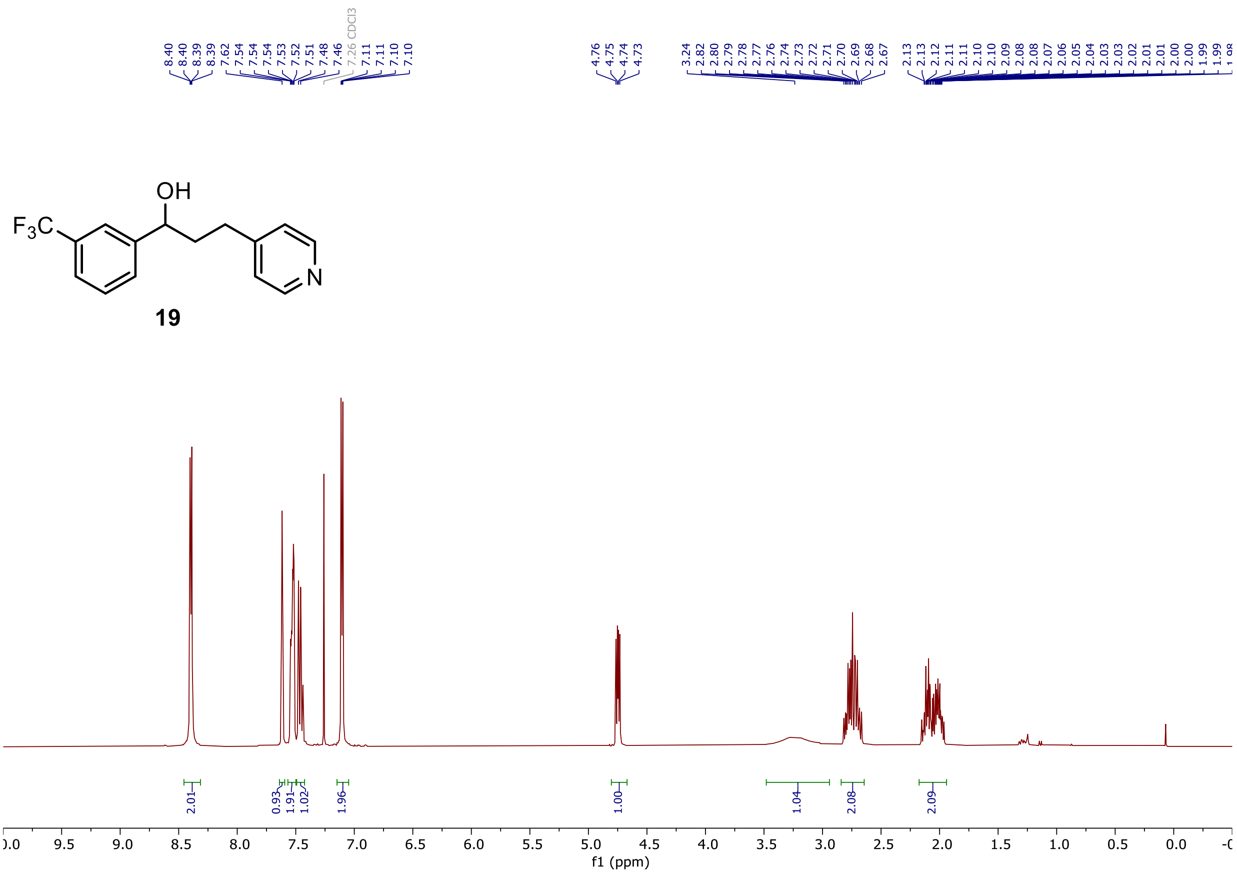

^{13}C NMR (101 MHz, $CDCl_3$) of **19**

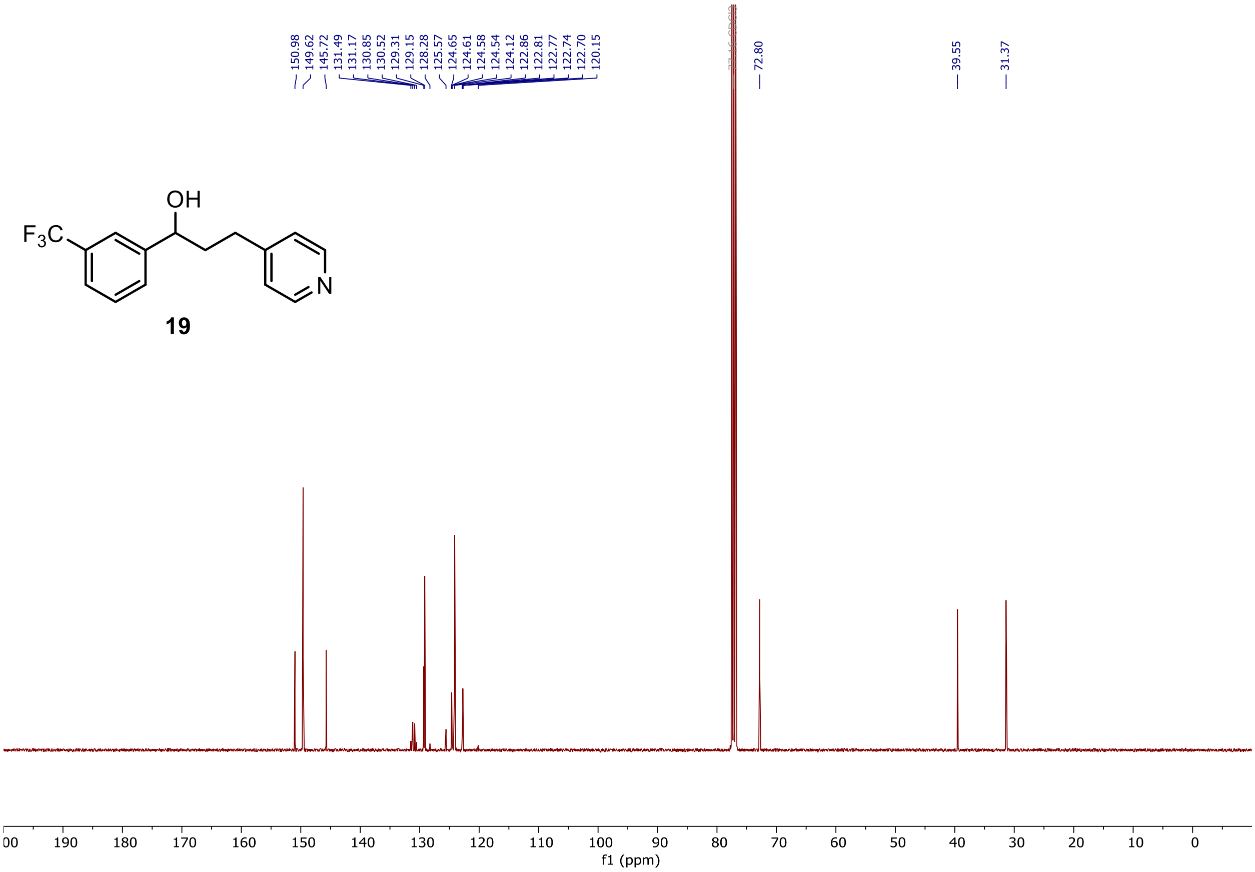

^{19}F NMR (377 MHz, $CDCl_3$) of **19**

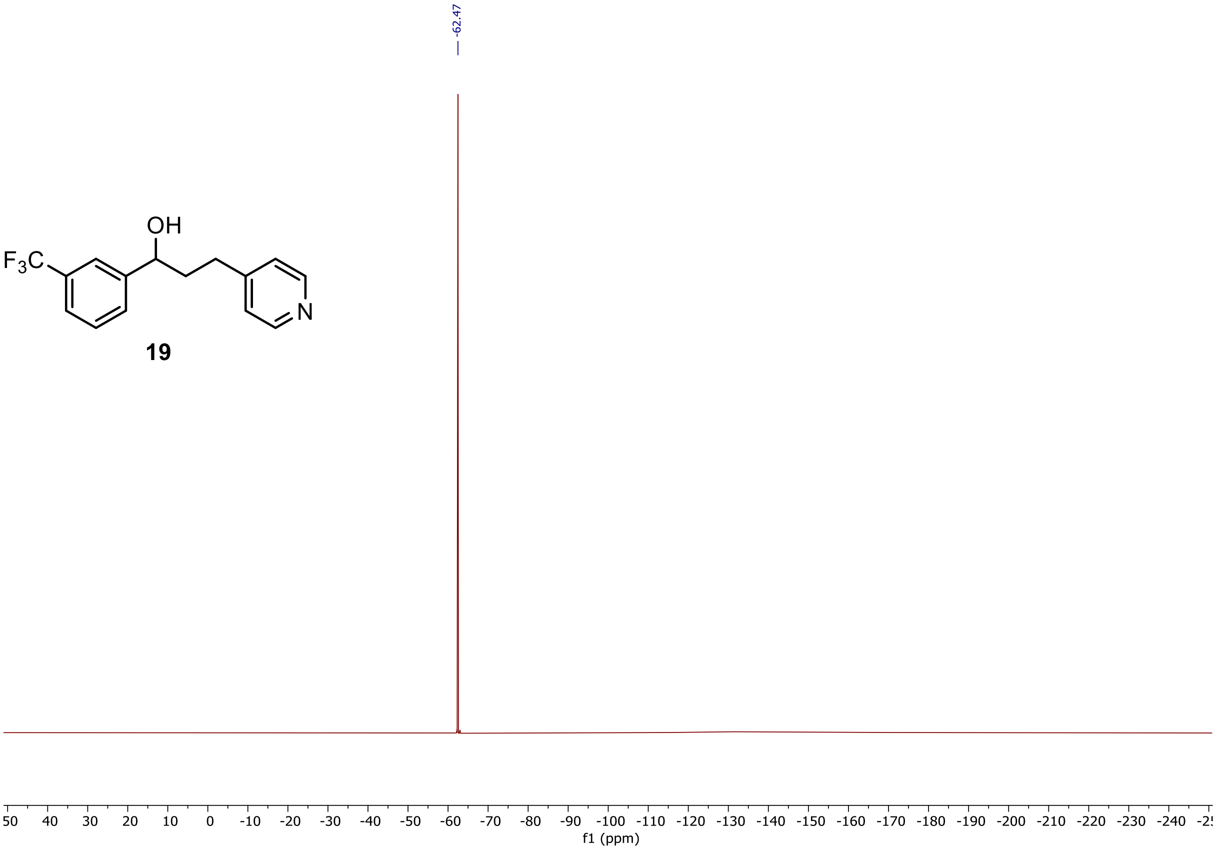

^{1}H NMR (400 MHz, $CDCl_3$) of **20** (*see procedure*)

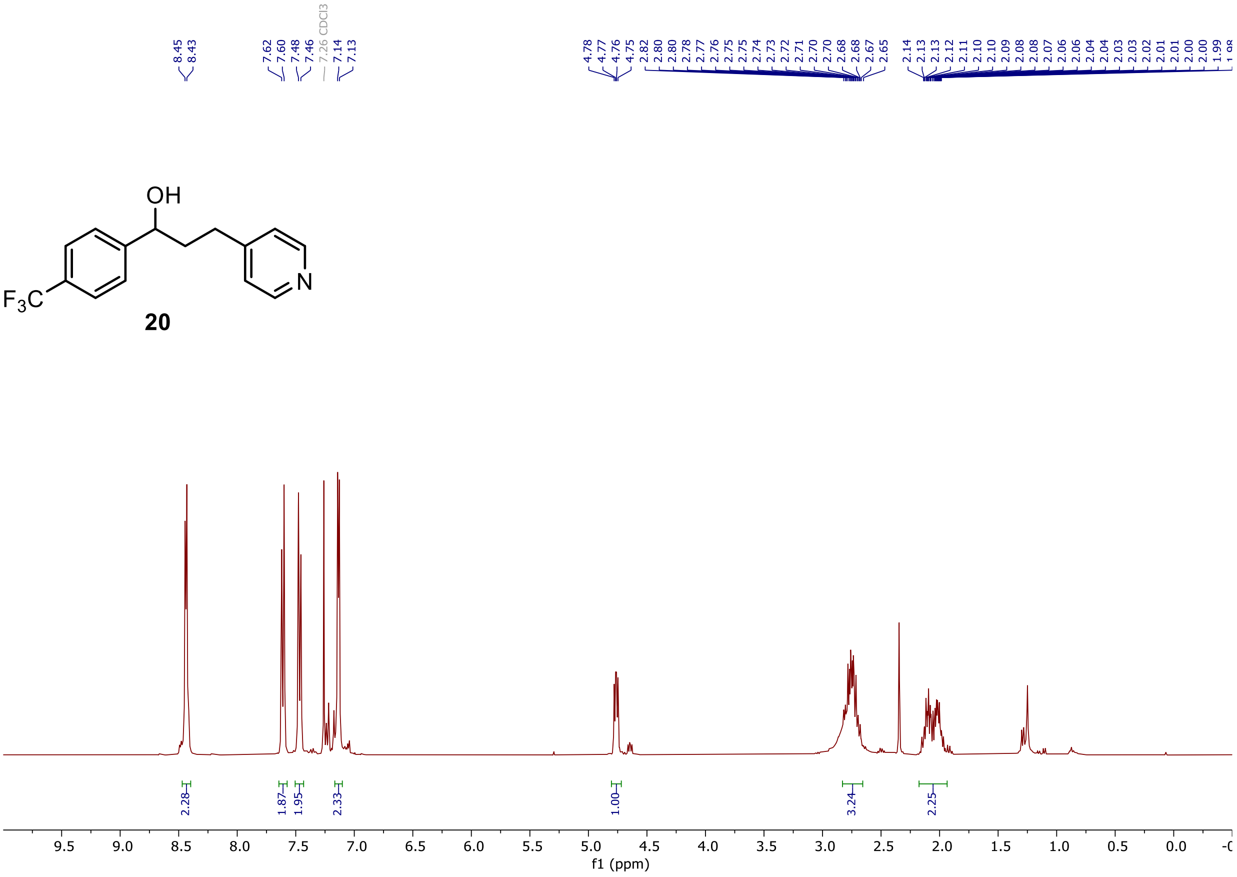

^{13}C NMR (101 MHz, $CDCl_3$) of **20**

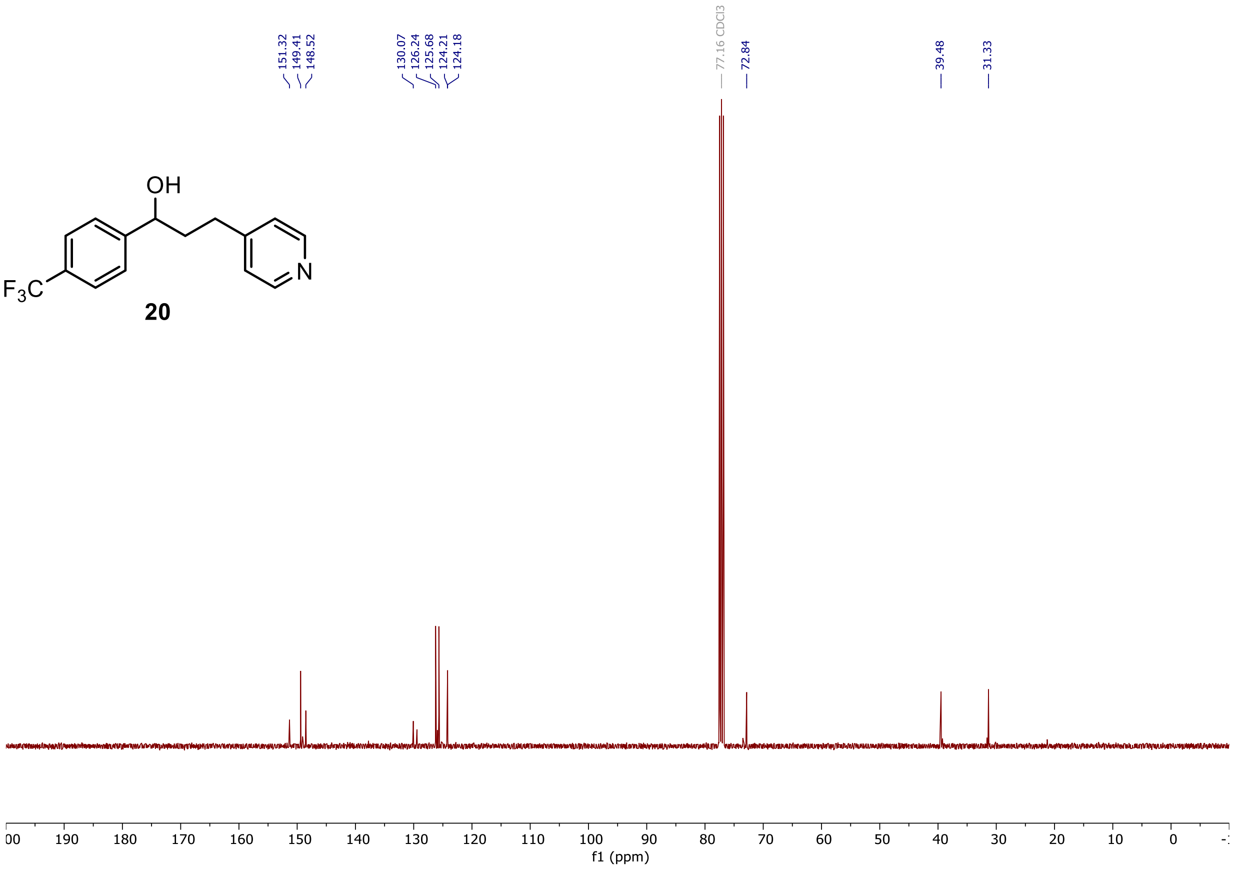

^{19}F NMR (377 MHz, $CDCl_3$) of **20**

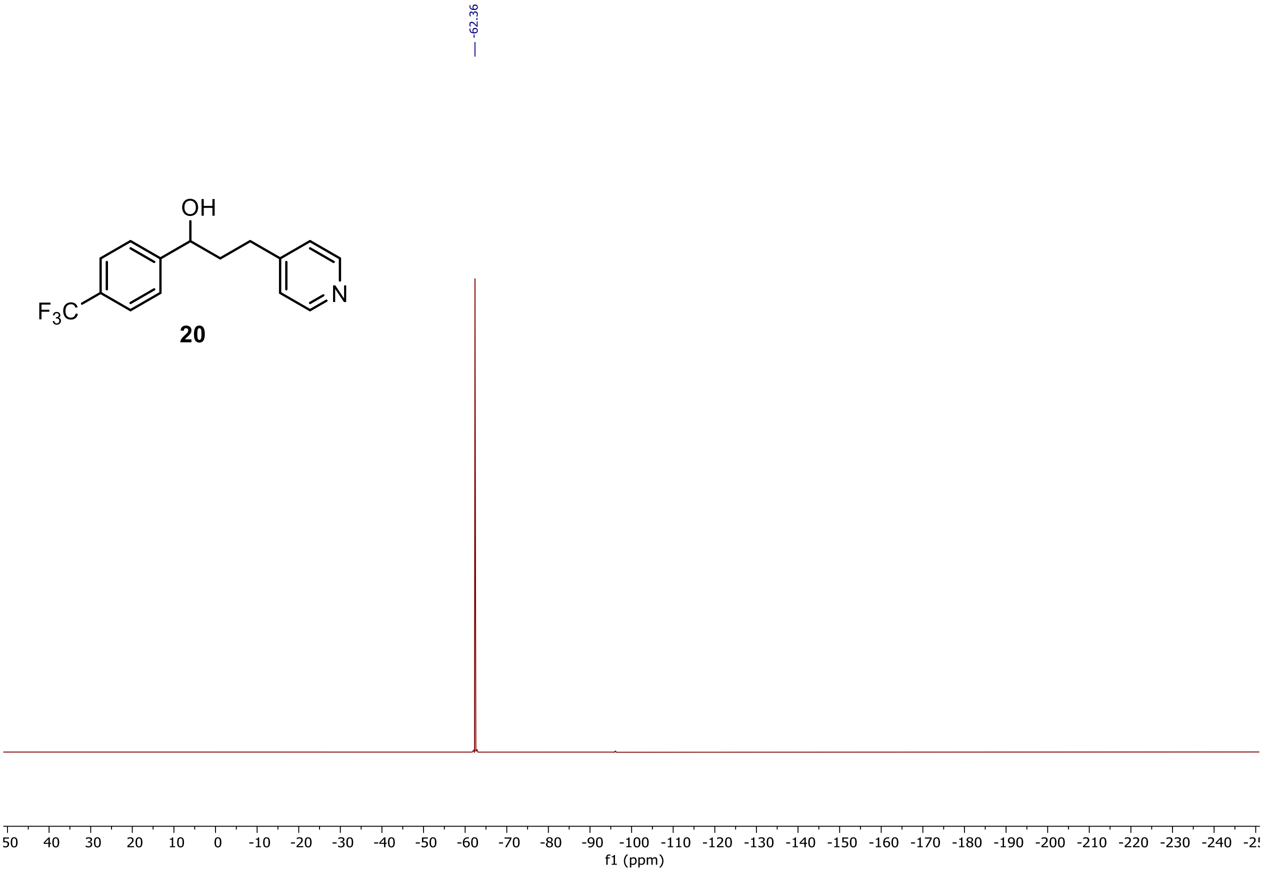

^{1}H NMR (400 MHz, CD_3OD) of **21** (*see procedure*)

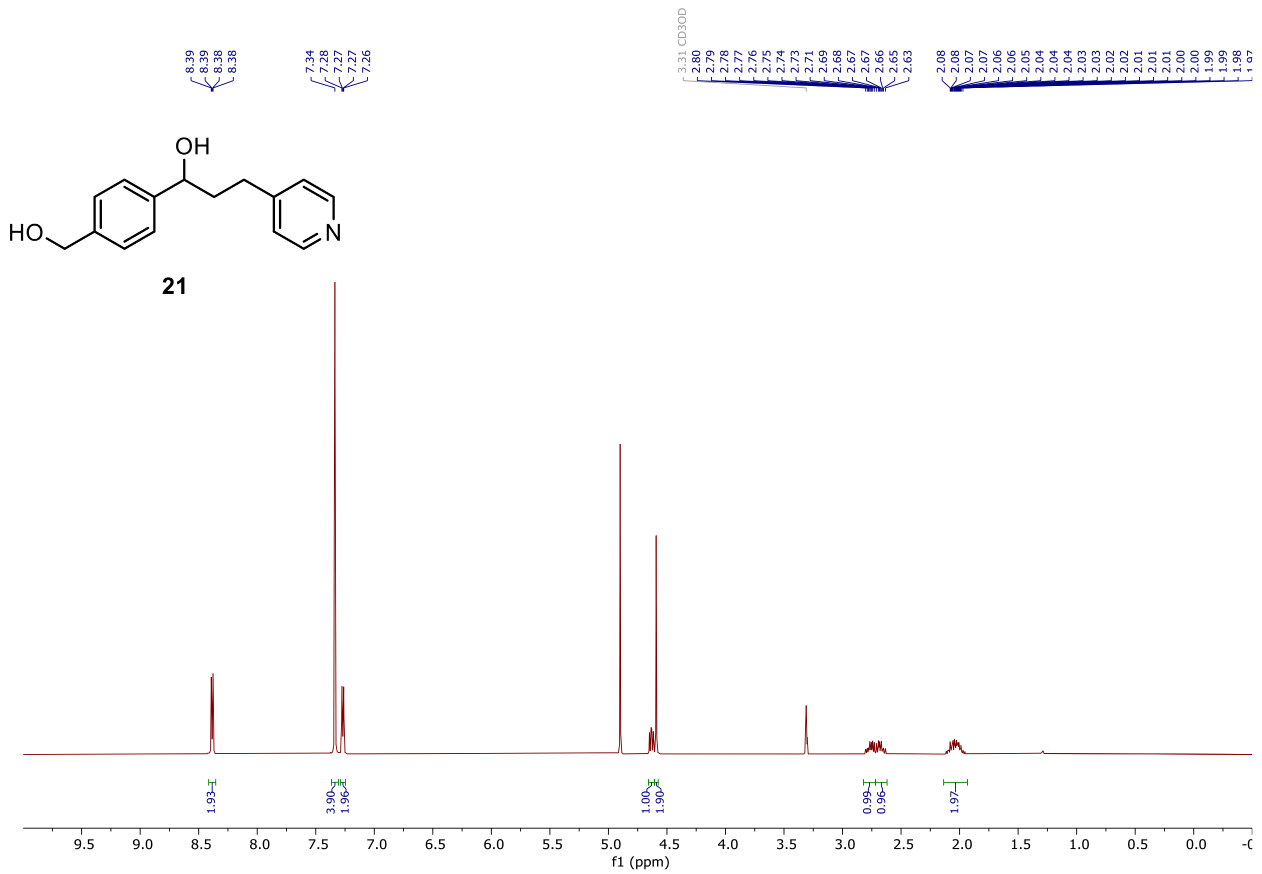

^{13}C NMR (101 MHz, CD_3OD) of **21**

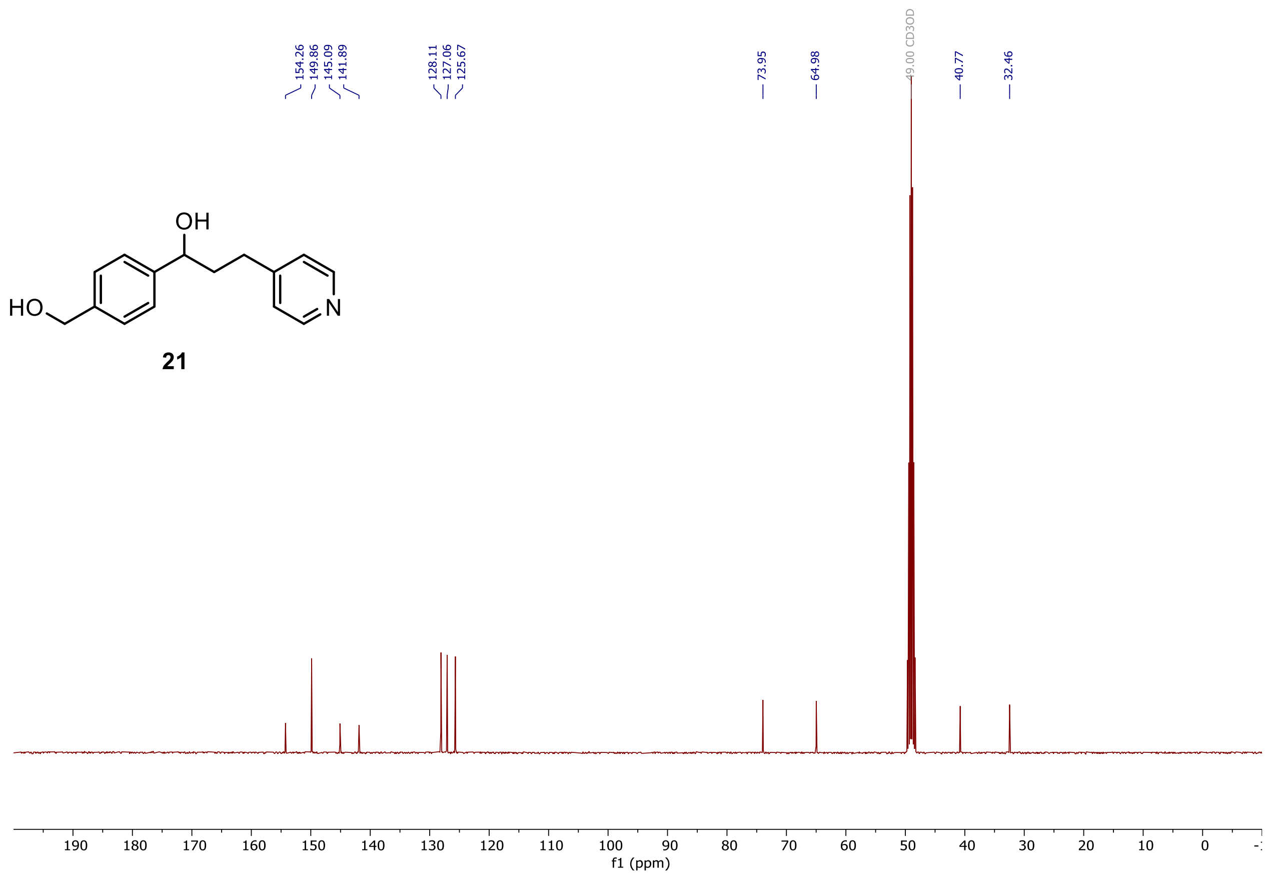

^{1}H NMR (400 MHz, CD_3OD) of **22** (*see procedure*)

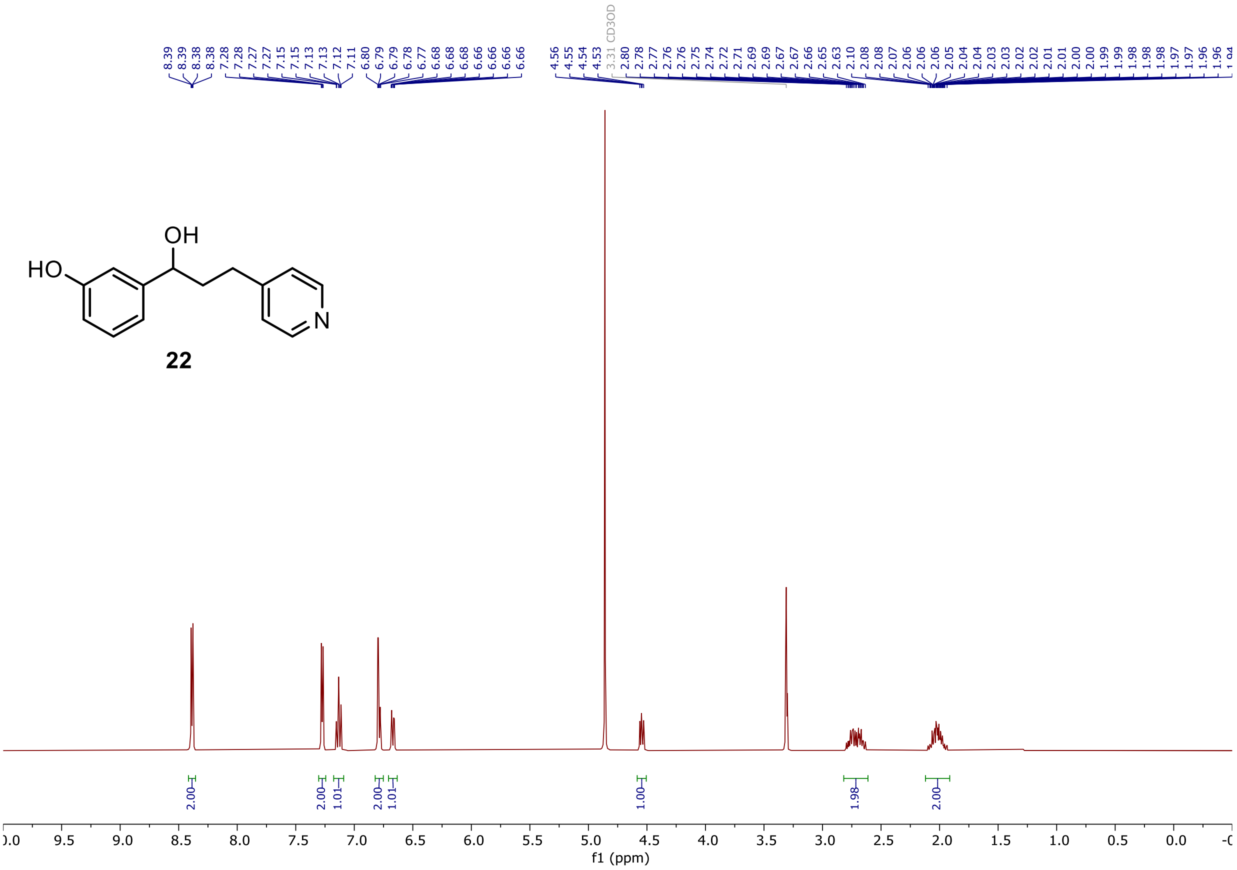

^{13}C NMR (101 MHz, CD_3OD) of **22**

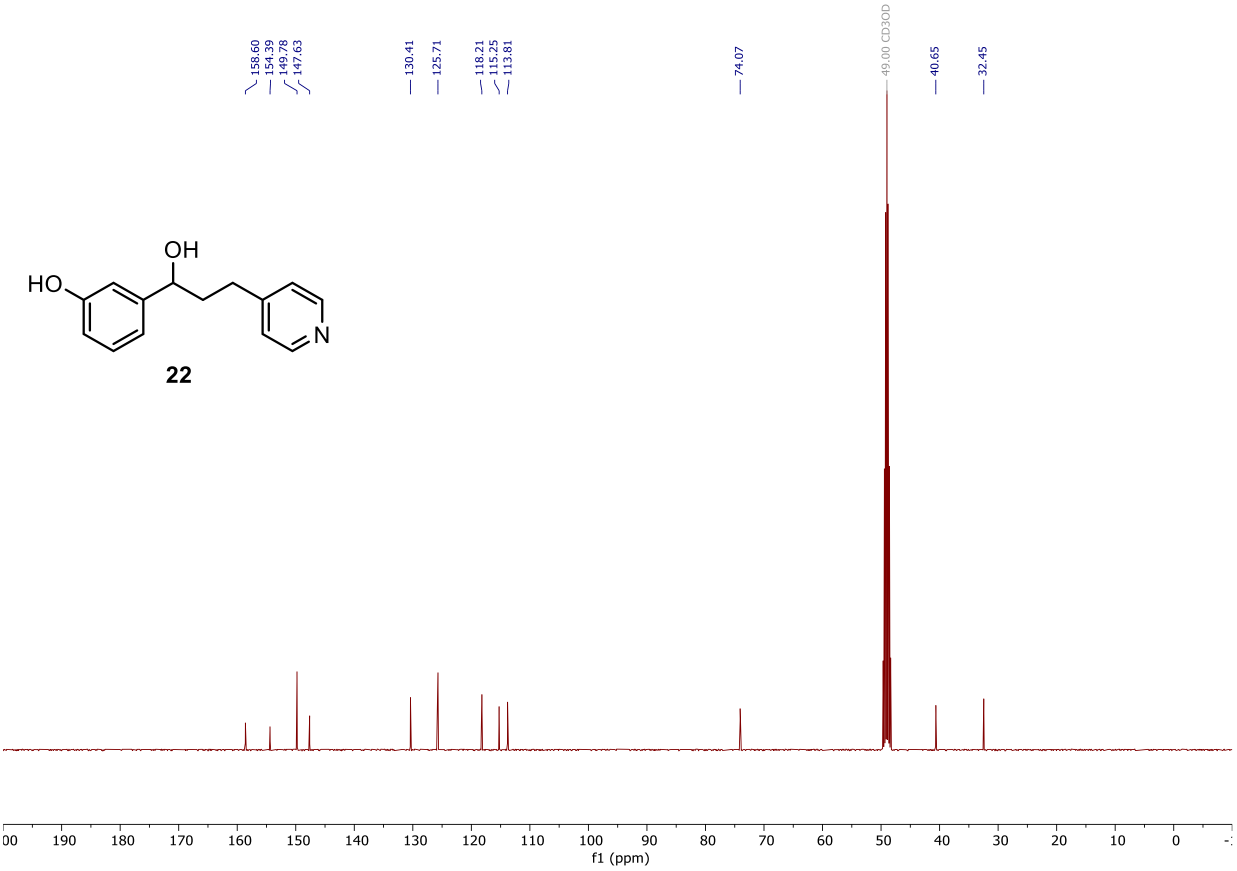

^{1}H NMR (500 MHz, CD_3OD) of **23** (see procedure)

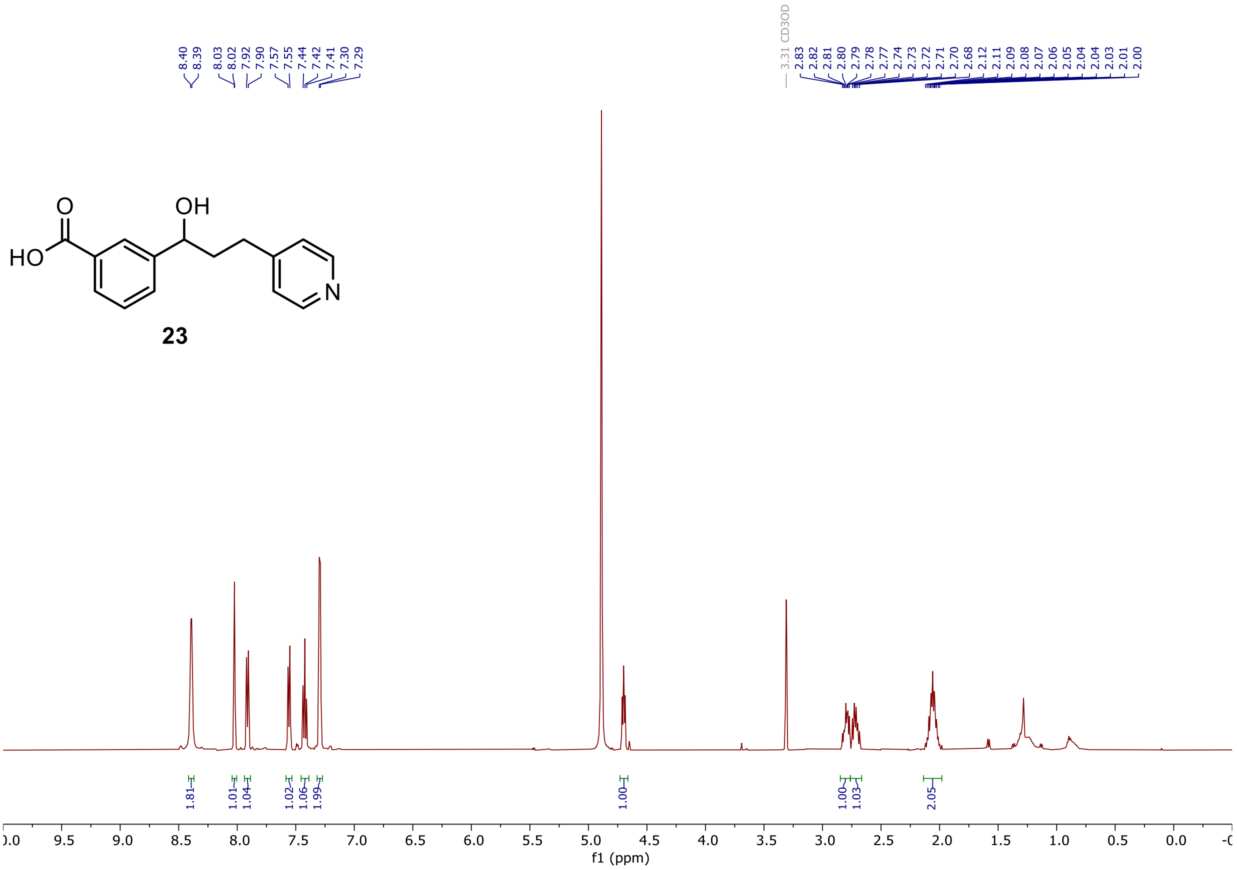

^{13}C NMR (126 MHz, CD_3OD) of **23**

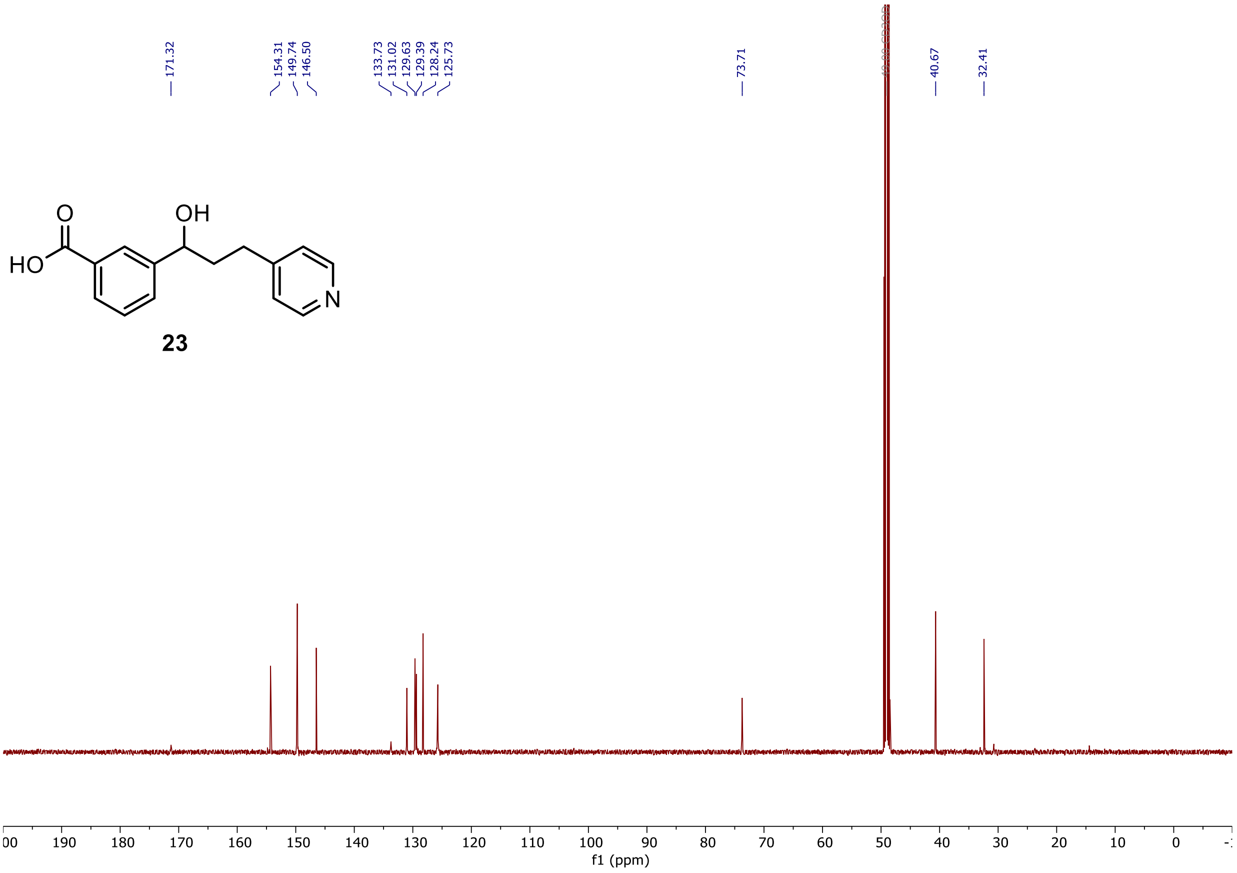

^{1}H NMR (400 MHz, $CDCl_3$) of **24** (*see procedure*)

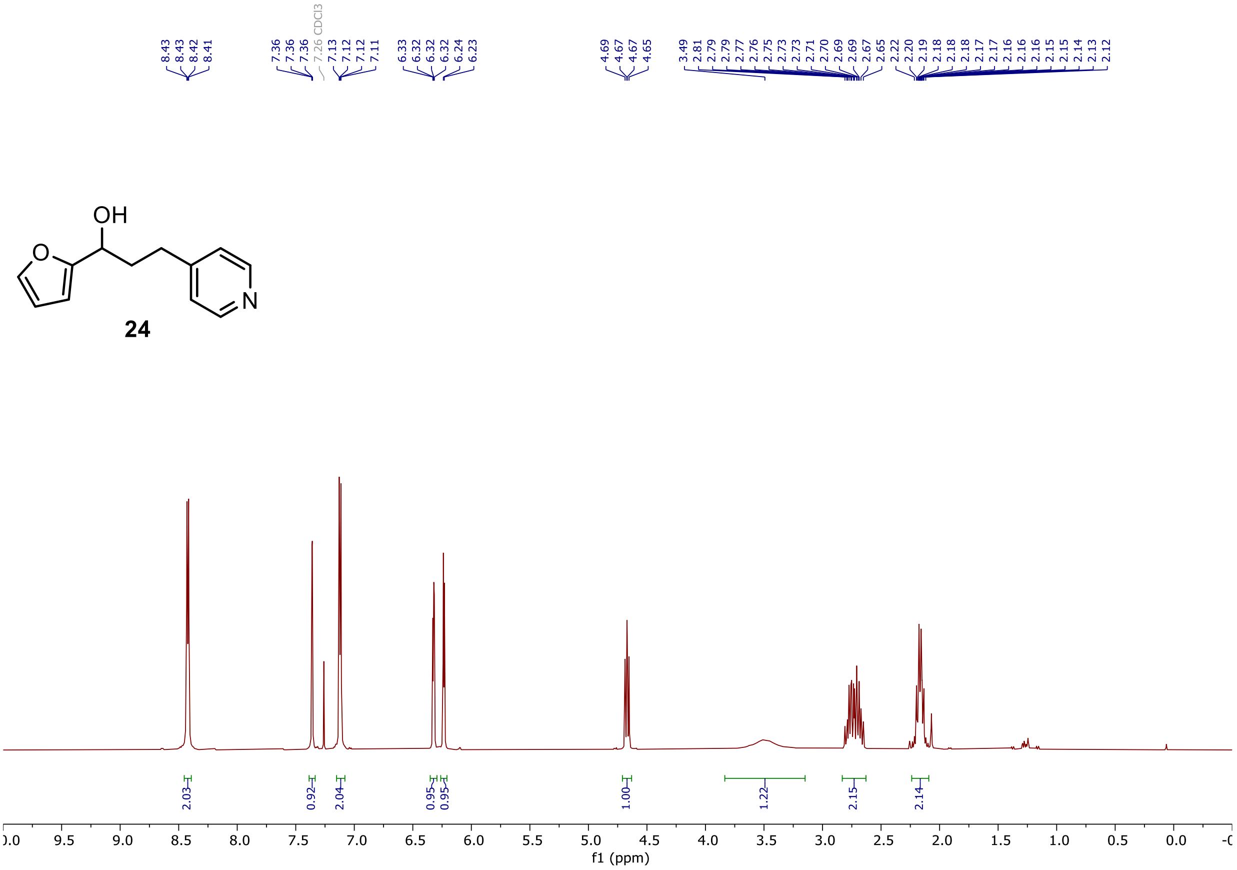

^{13}C NMR (101 MHz, $CDCl_3$) of **24**

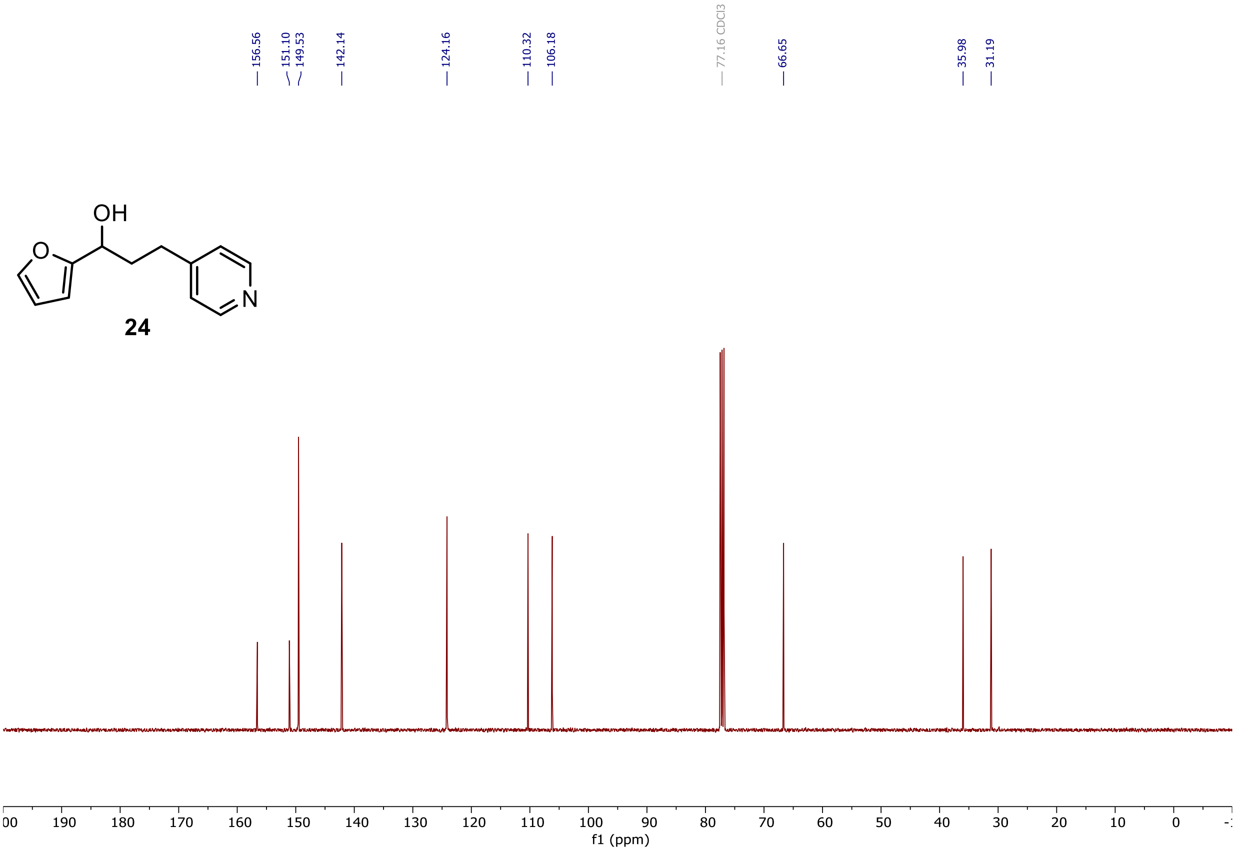

^{1}H NMR (400 MHz, $CDCl_3$) of **25** (*see procedure*)

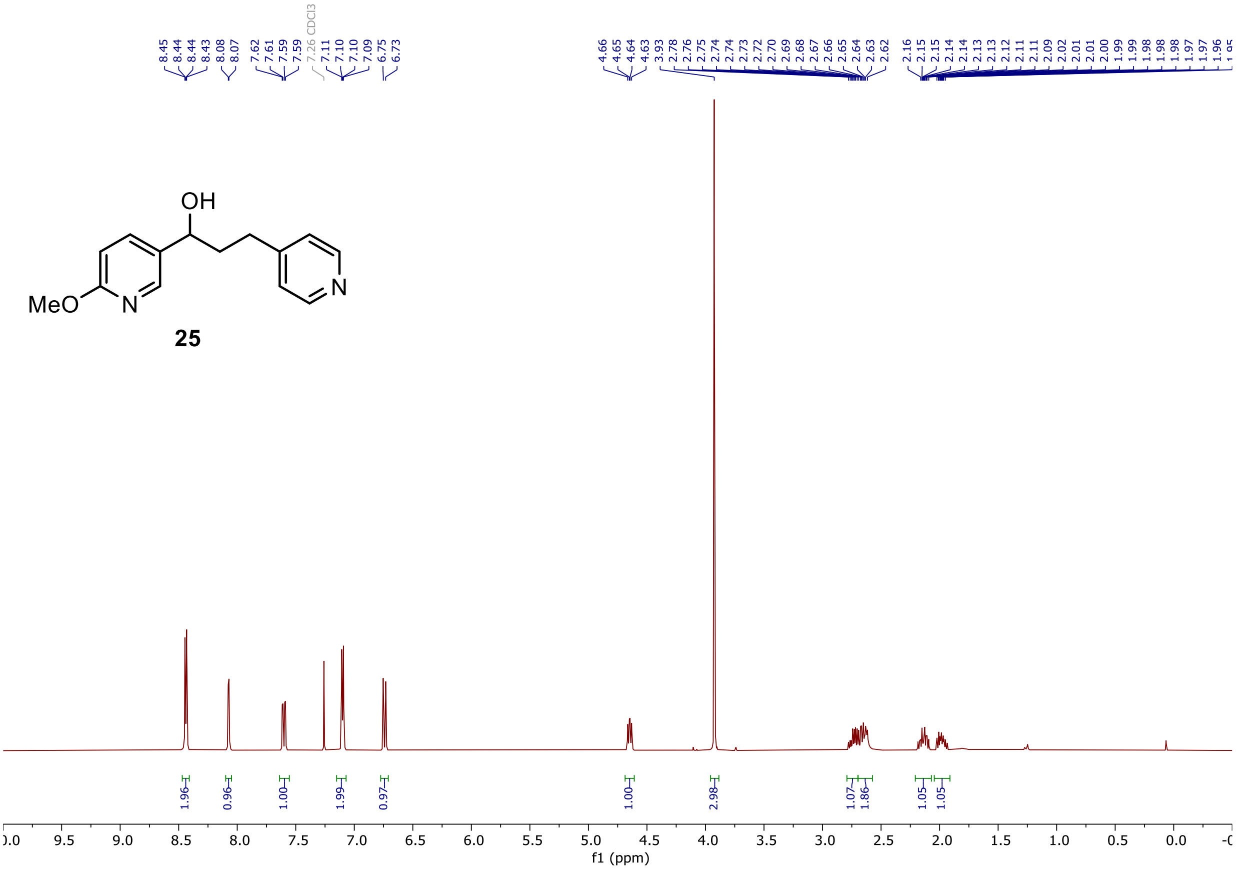

^{13}C NMR (101 MHz, $CDCl_3$) of **25**

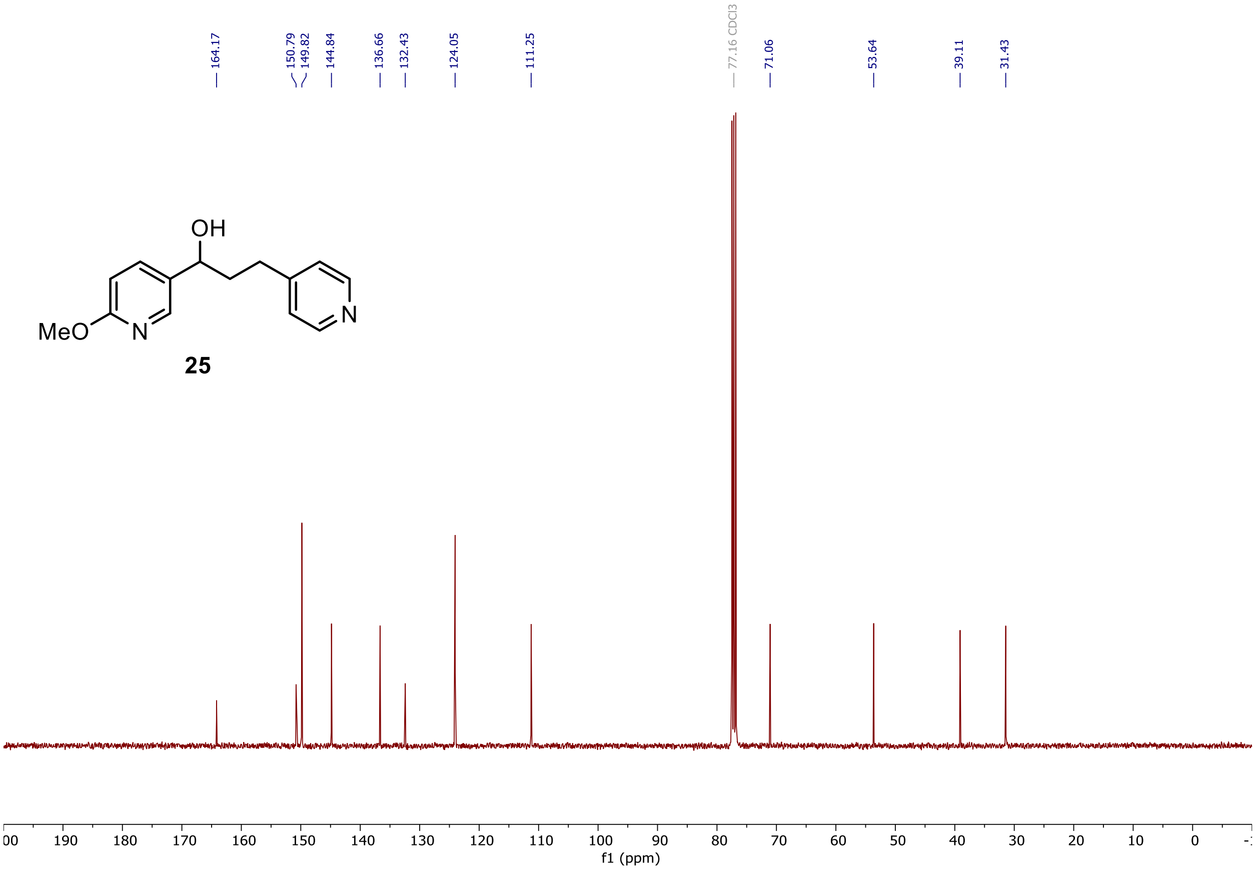

^{1}H NMR (500 MHz, $CDCl_3$) of **26** (*see procedure*)

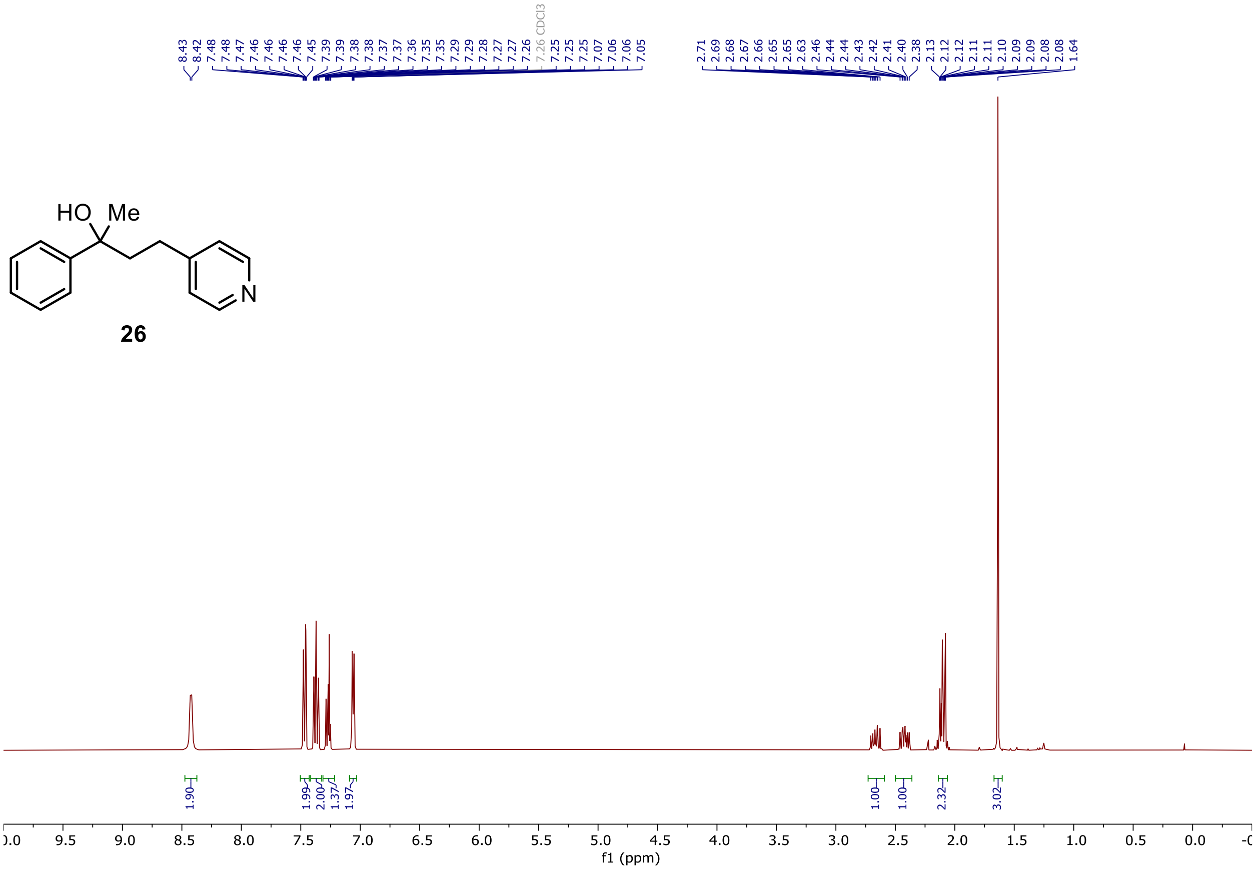

^{13}C NMR (126 MHz, $CDCl_3$) of **26**

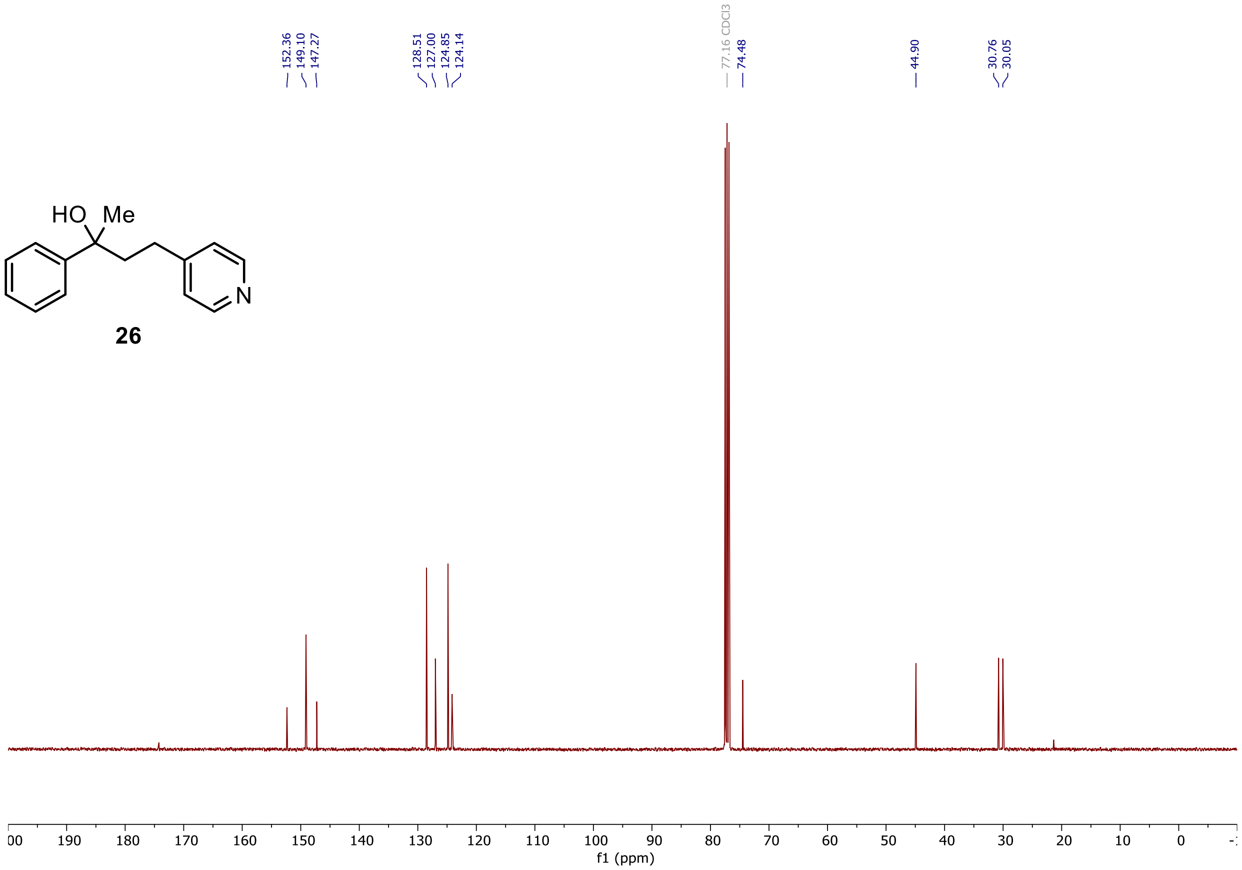

^{1}H NMR (400 MHz, $CDCl_3$) of **27** (*see procedure*)

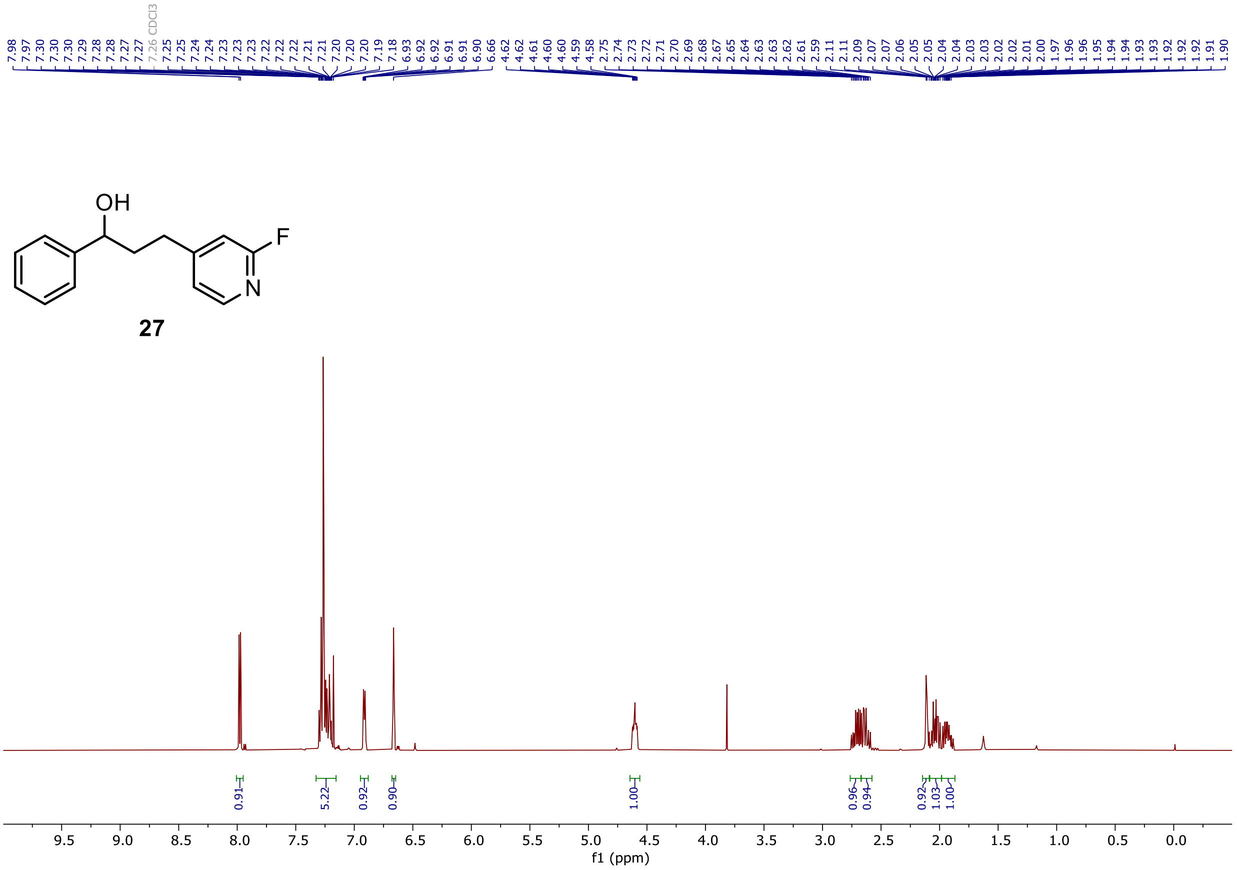

^{13}C NMR (101 MHz, $CDCl_3$) of **27**

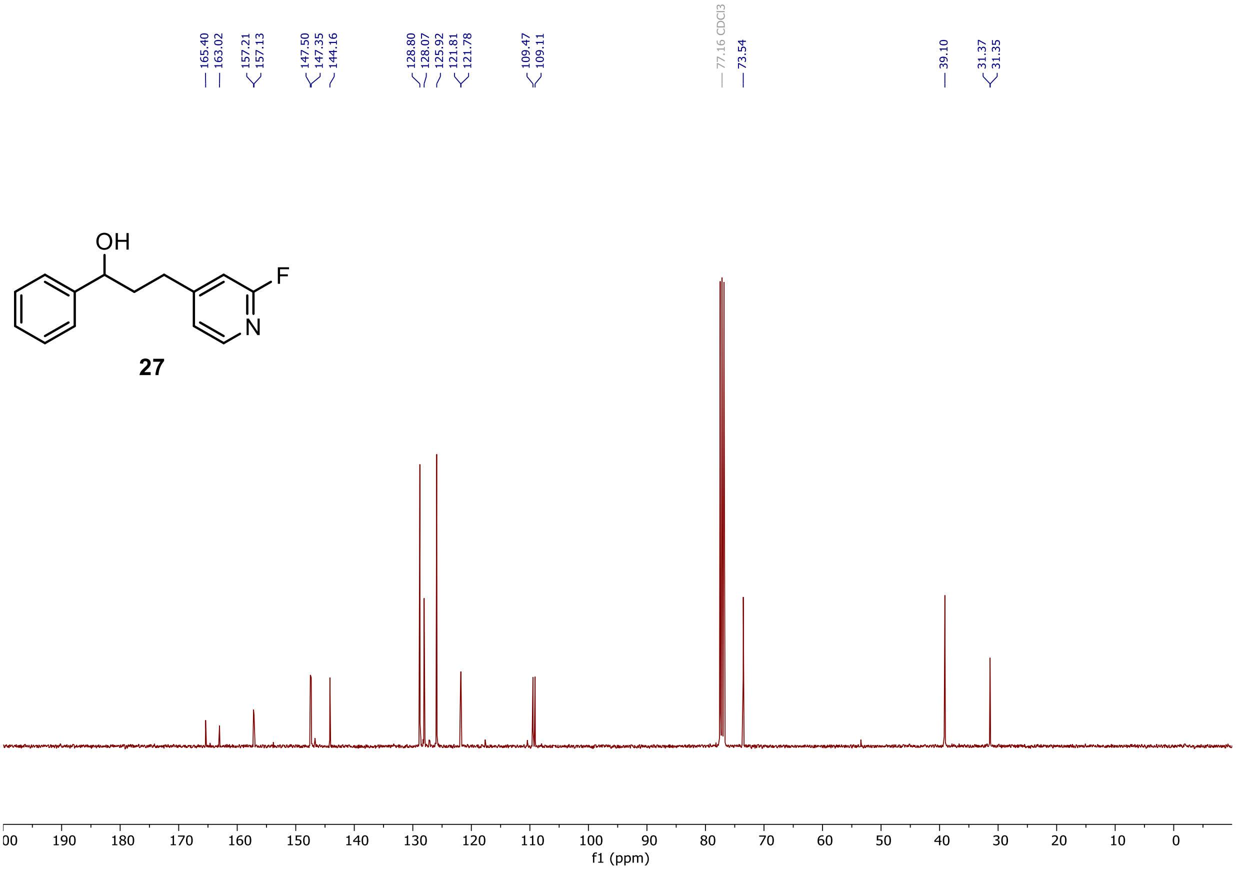

^{19}F NMR (377 MHz, $CDCl_3$) of **27**

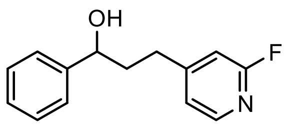

27

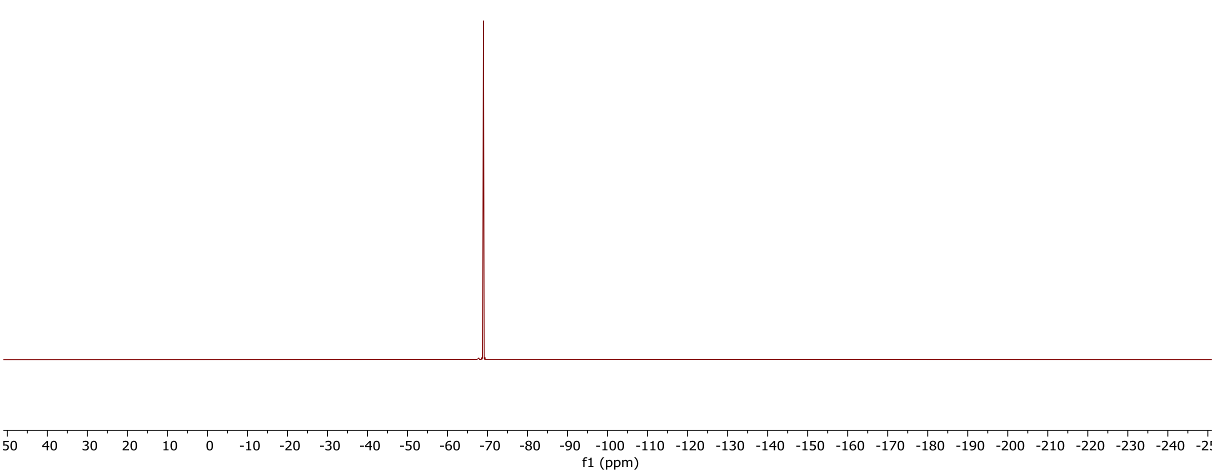

^{1}H NMR (400 MHz, CDCl₃) of **28** (*see procedure*)

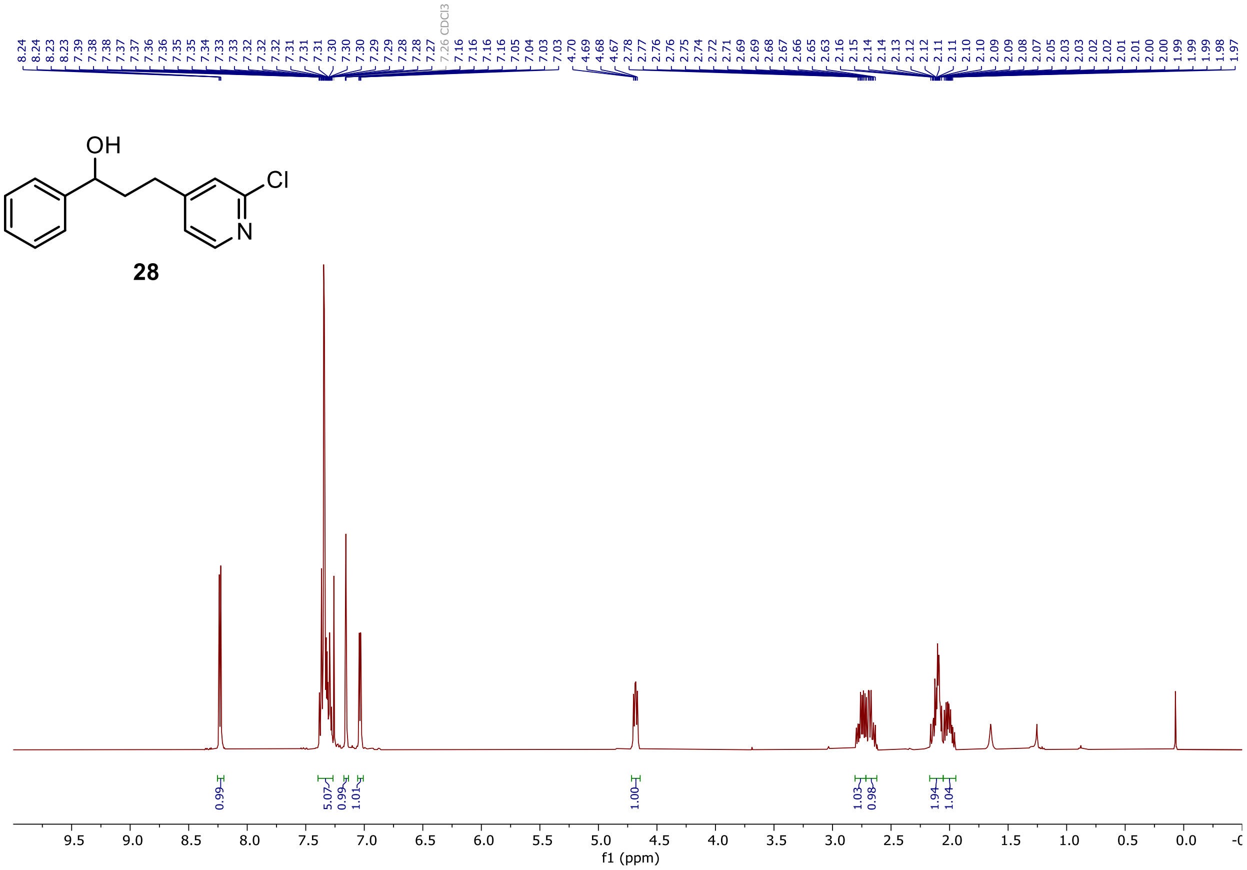

^{13}C NMR (101 MHz, CDCl₃) of **28**

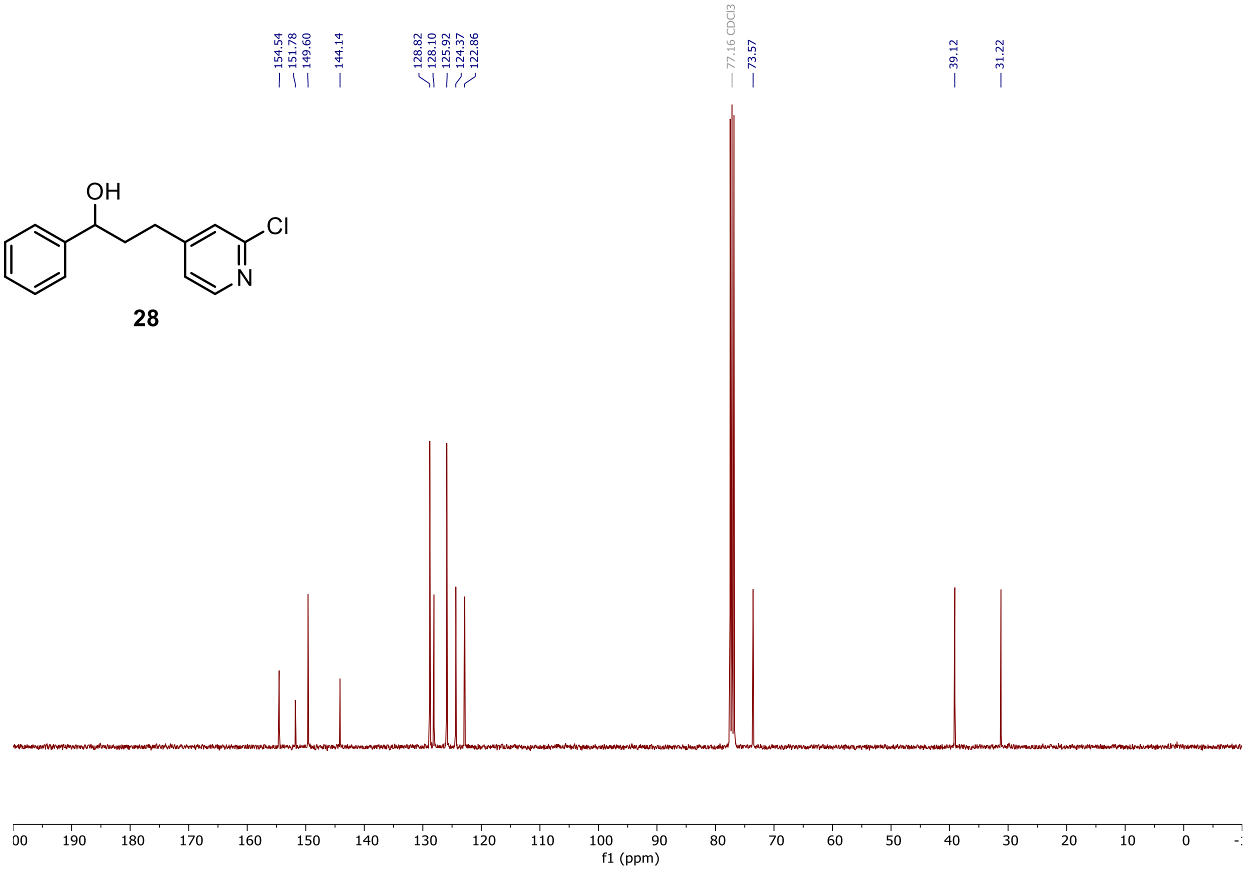

^{1}H NMR (400 MHz, $CDCl_3$) of **29** (*see procedure*)

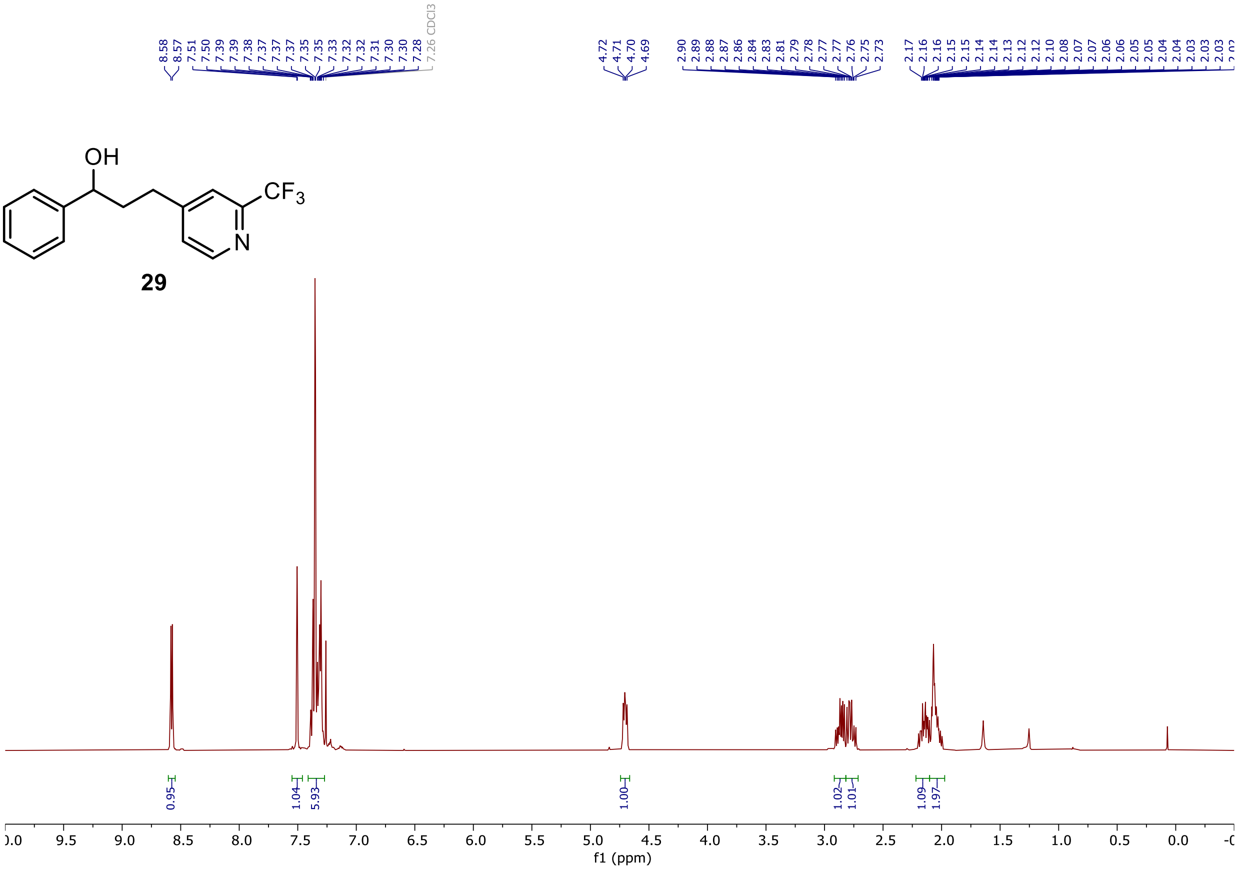

^{13}C NMR (101 MHz, $CDCl_3$) of **29**

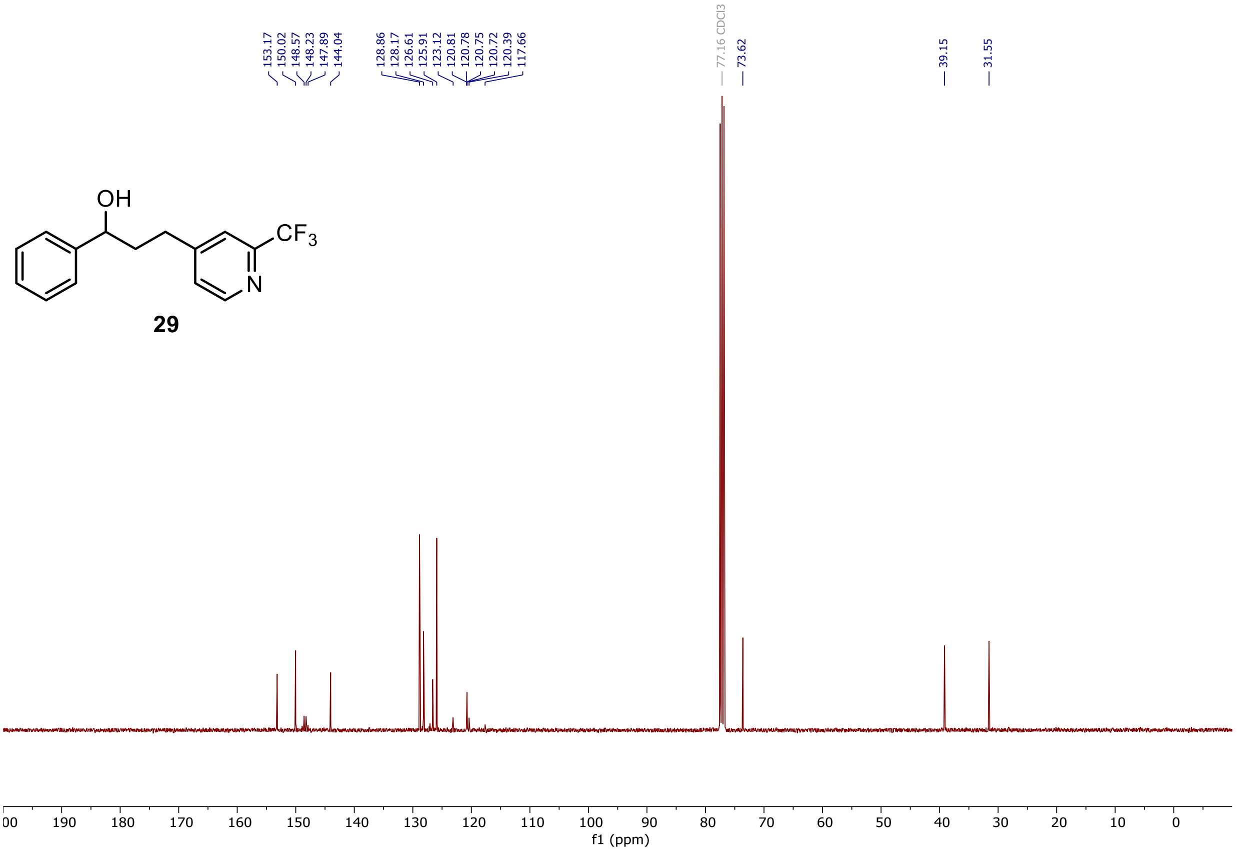

^{19}F NMR (377 MHz, $CDCl_3$) of **29**

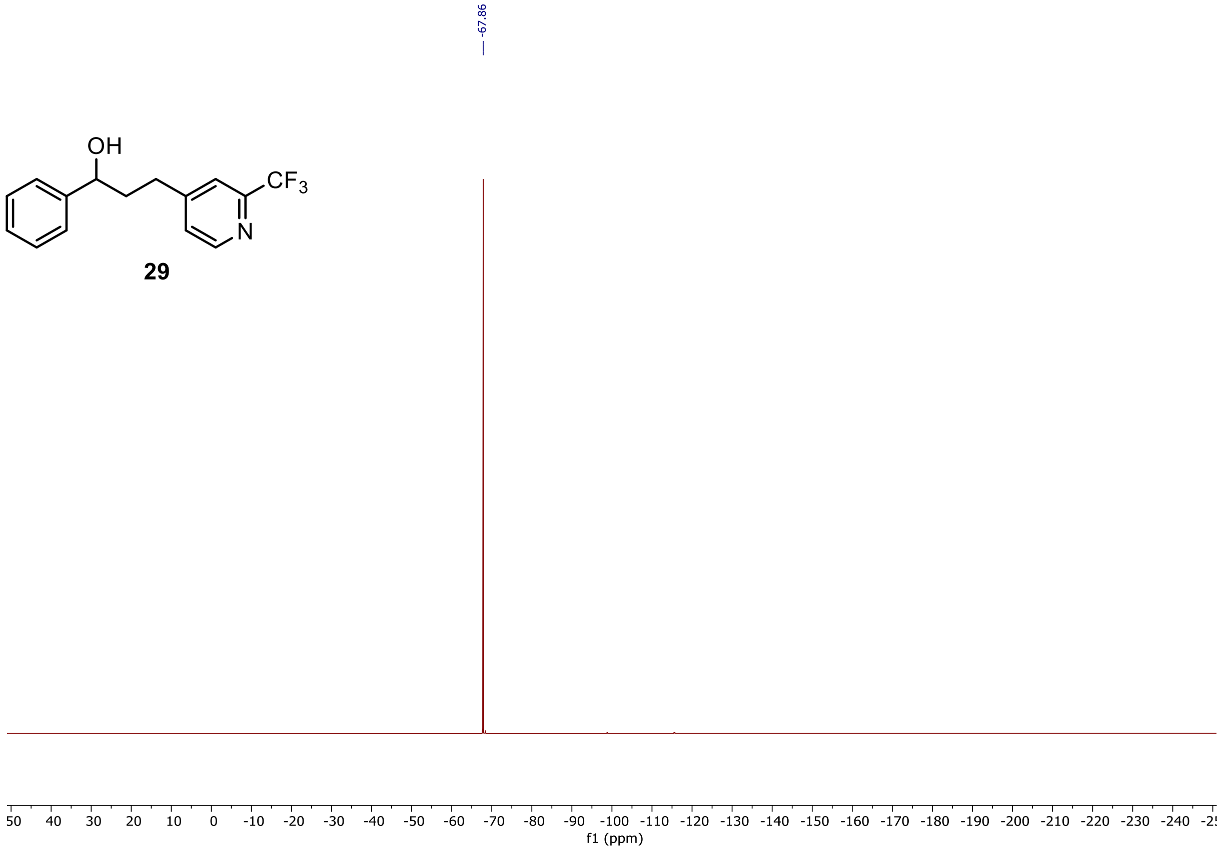

29

^{1}H NMR (400 MHz, $CDCl_3$) of **30** (*see procedure*)

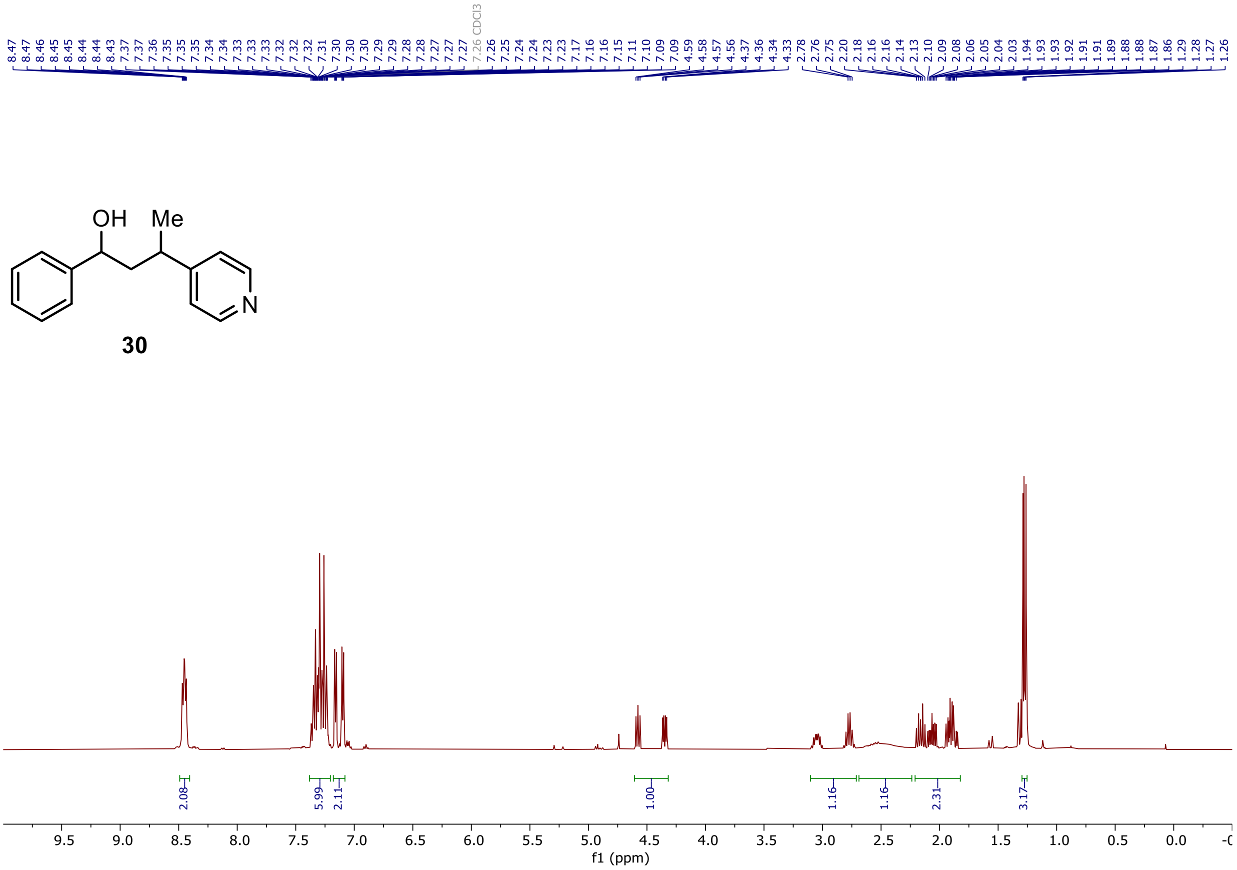

^{13}C NMR (101 MHz, $CDCl_3$) of **30**

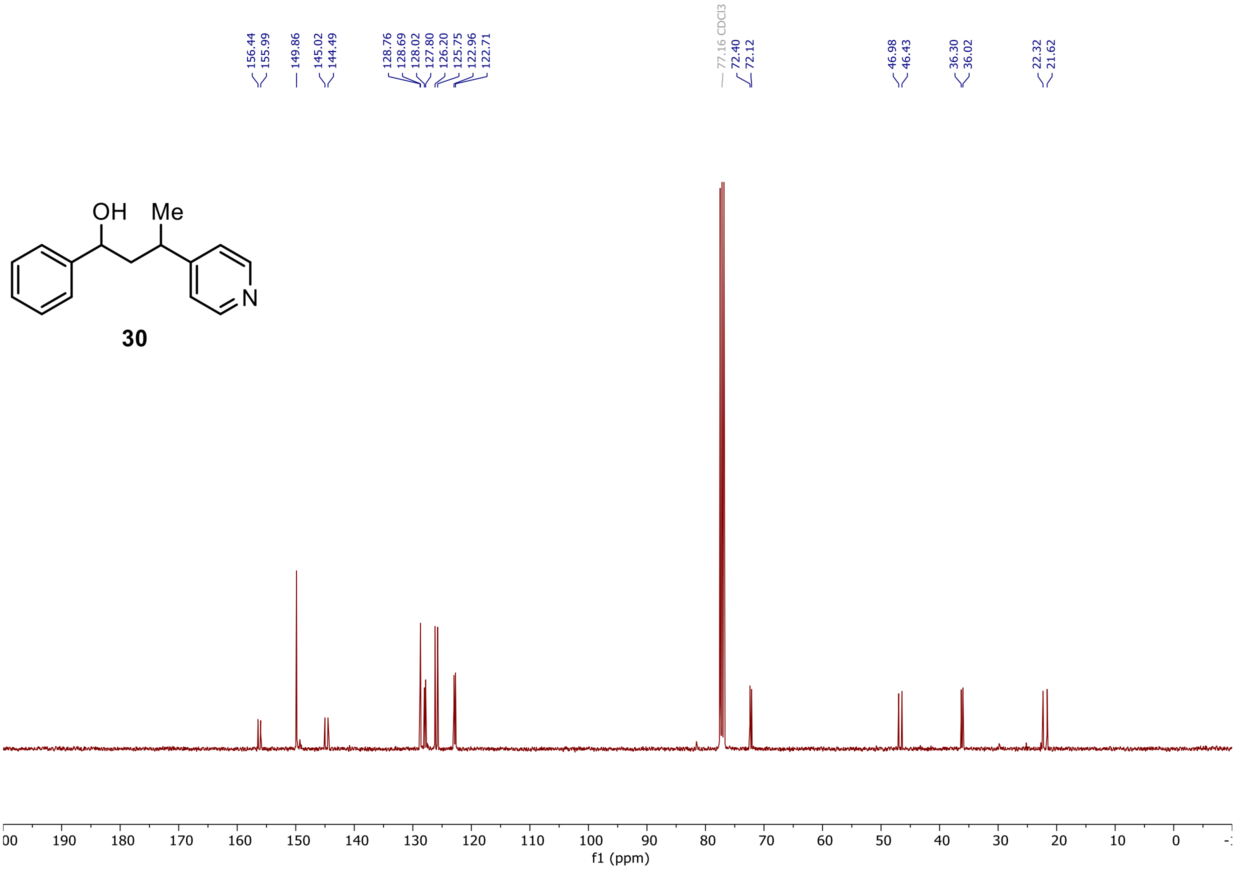

^{1}H NMR (400 MHz, $CDCl_3$) of **31** (*see procedure*)

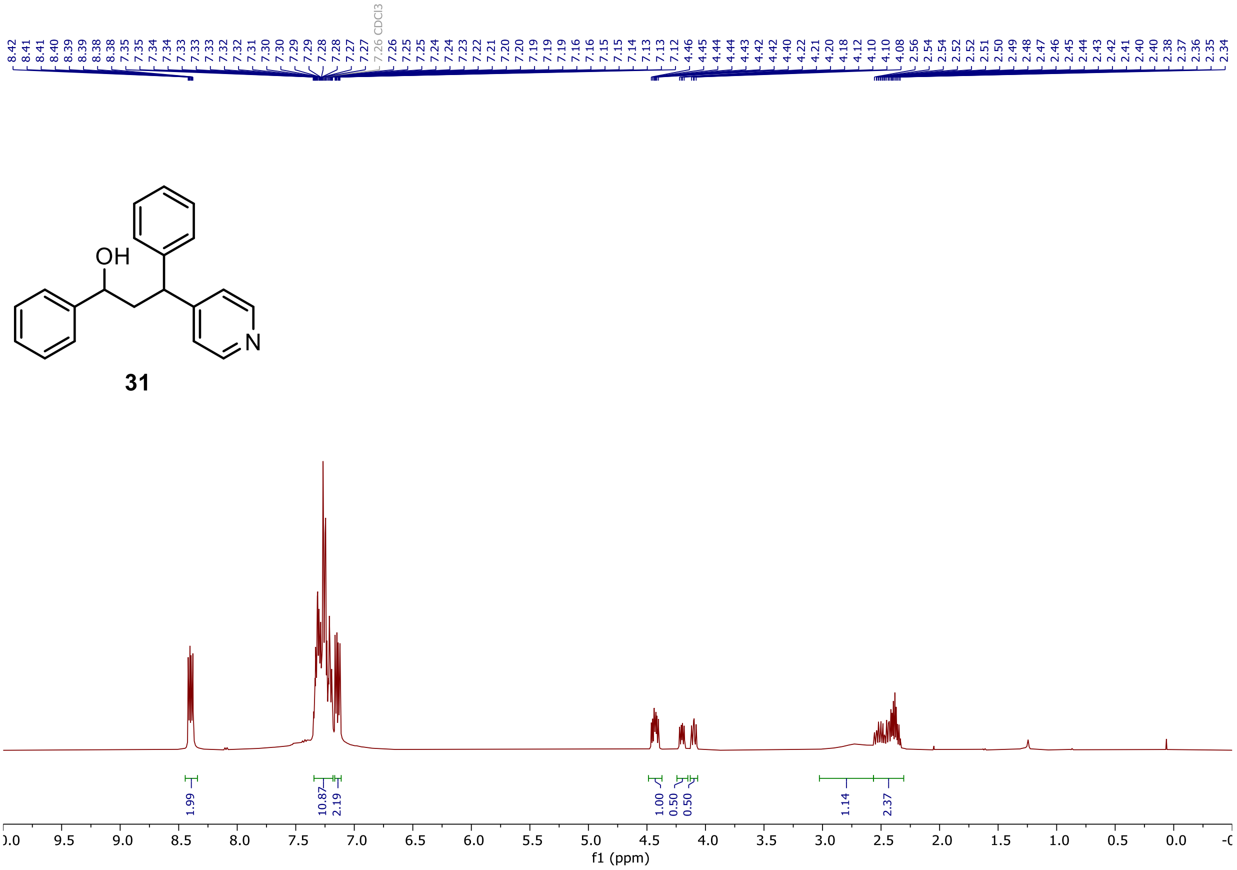

^{13}C NMR (101 MHz, $CDCl_3$) of **31**

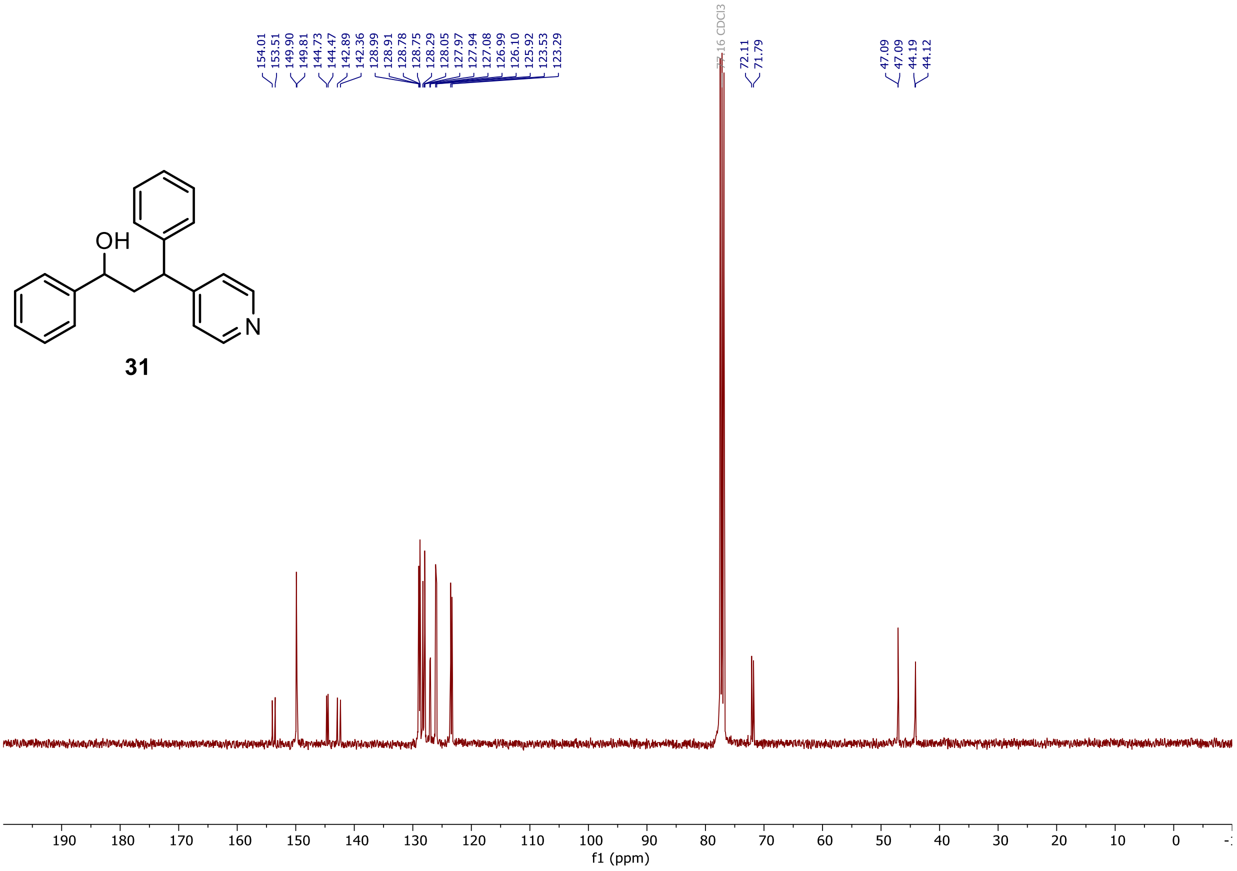

^{1}H NMR (500 MHz, $CDCl_3$) of **32** (*see procedure*)

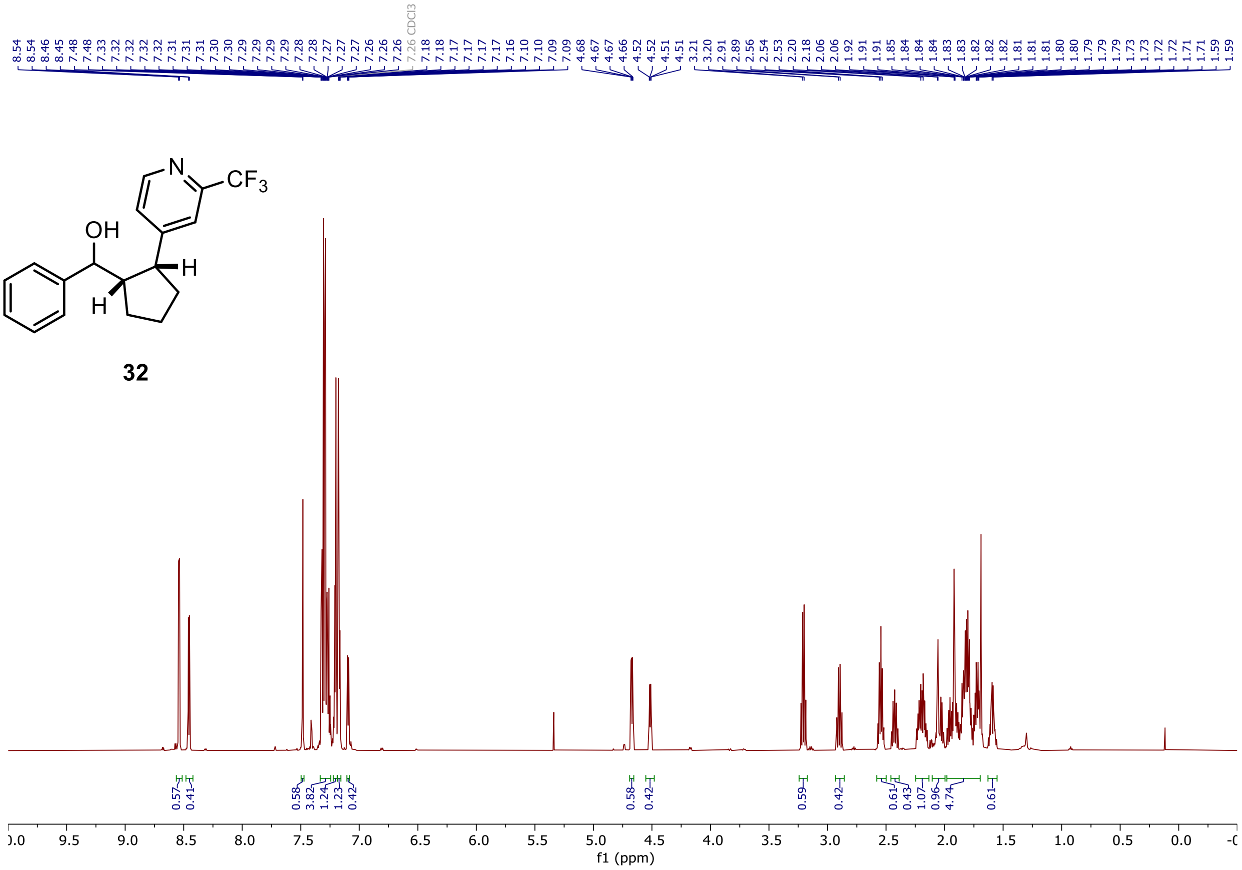

NOESY of **32**

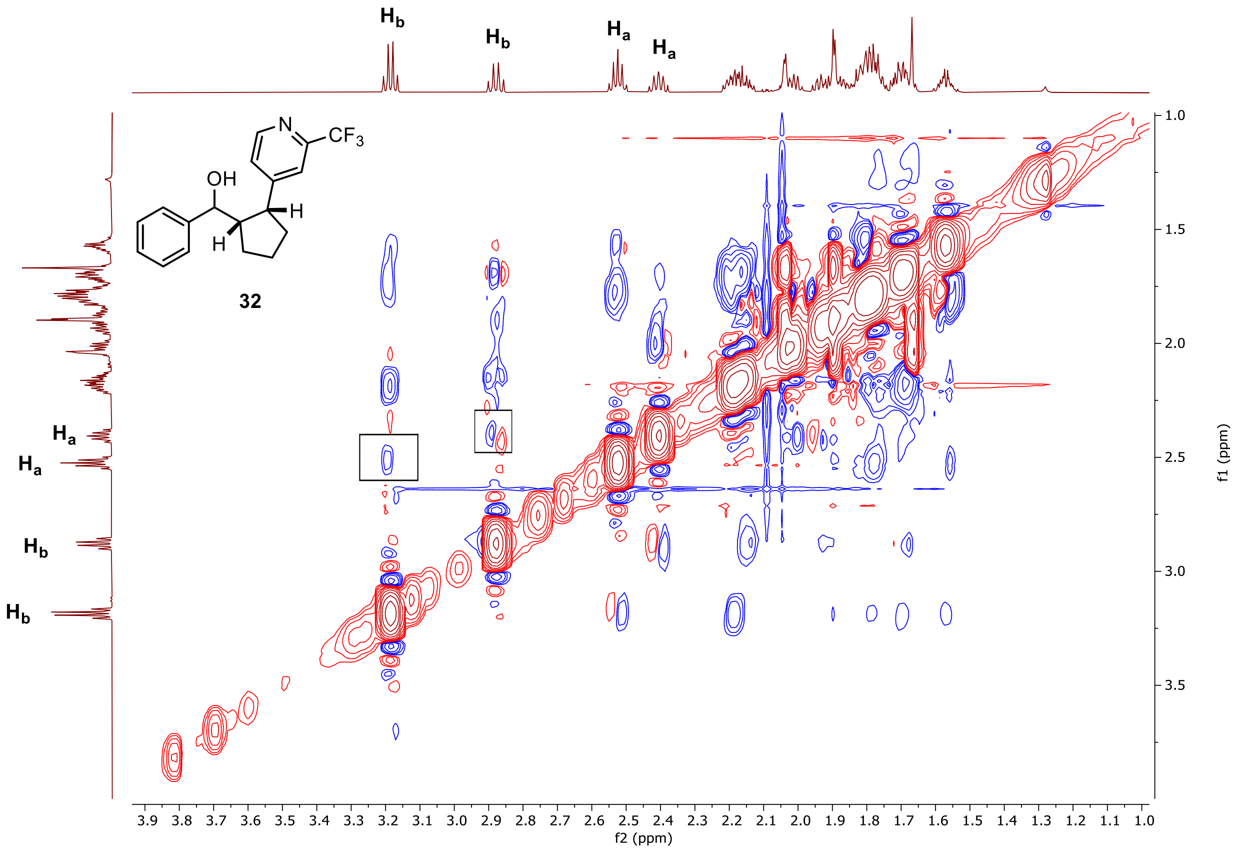

^{13}C NMR (126 MHz, $CDCl_3$) of **32**

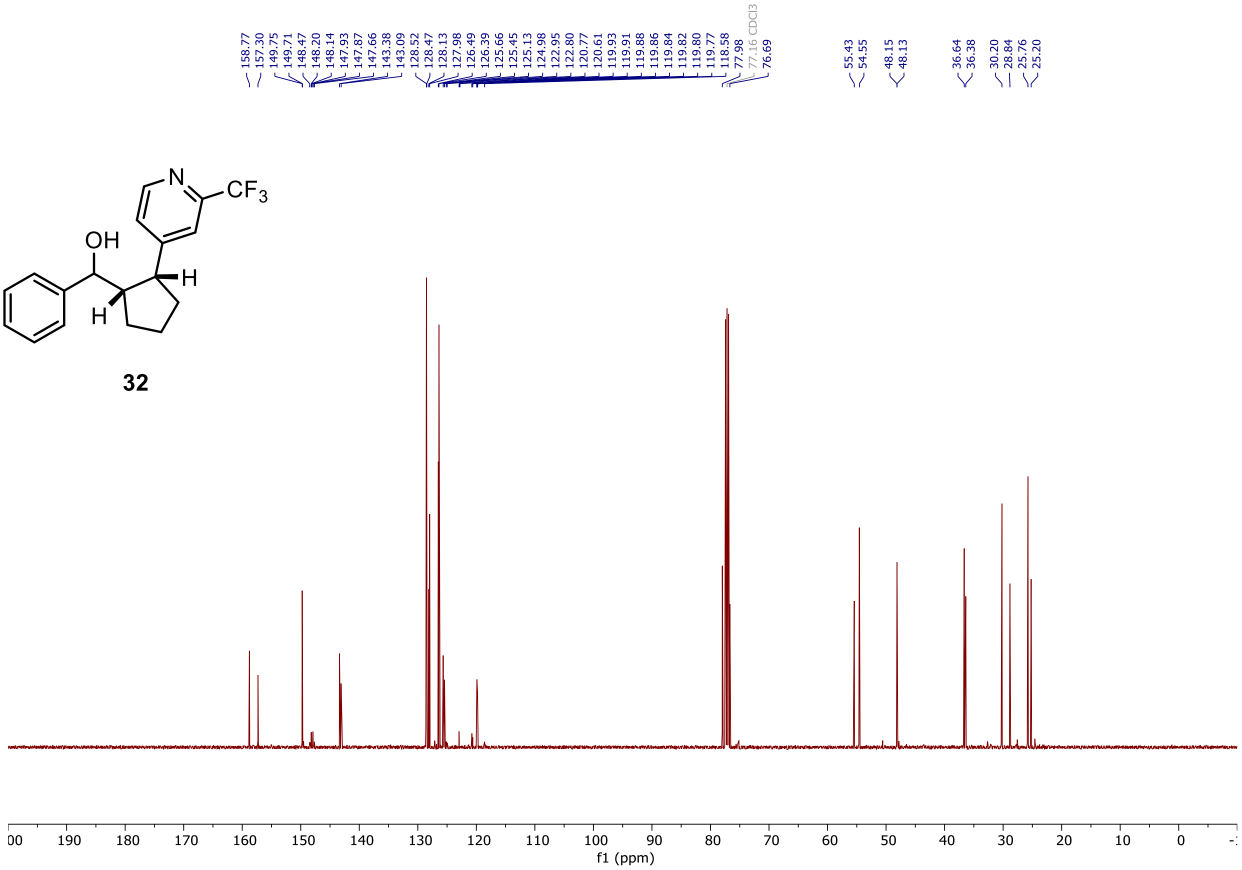

^{19}F NMR (377 MHz, $CDCl_3$) of **32**

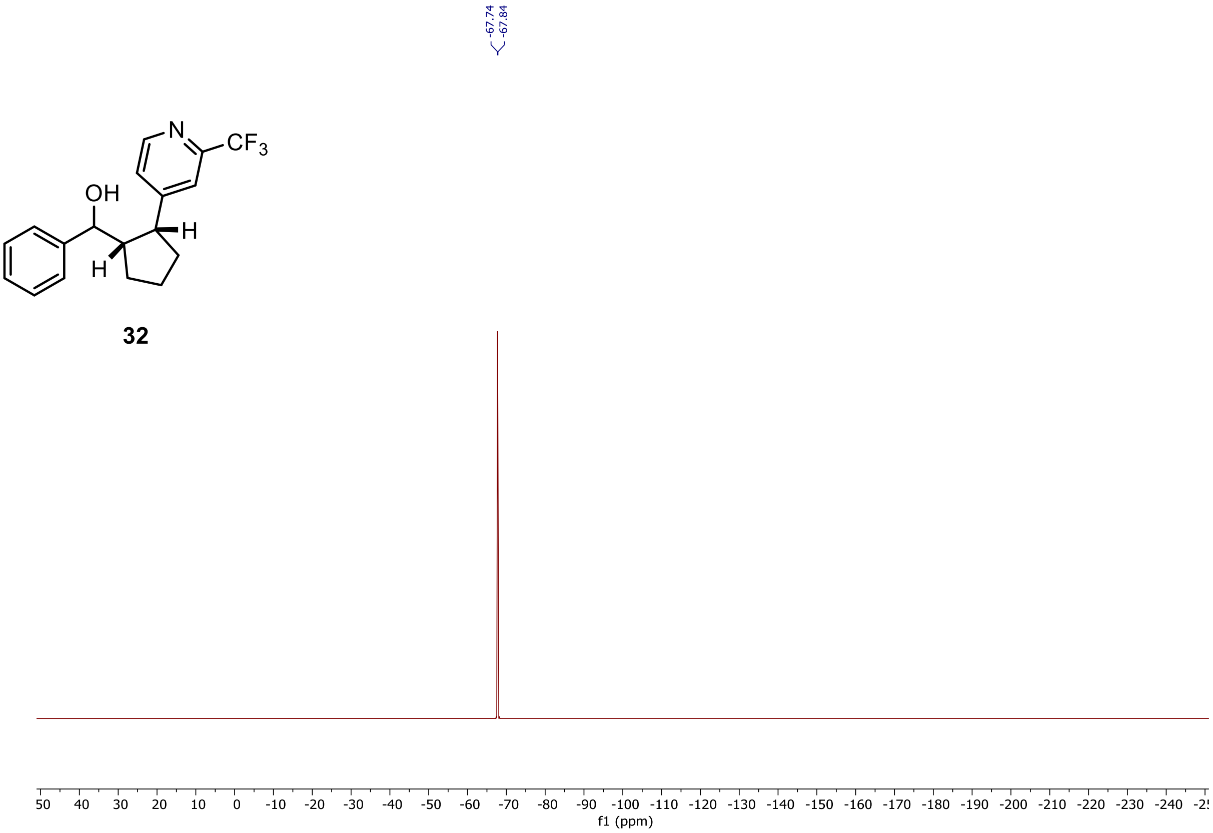

^{1}H NMR (400 MHz, $CDCl_3$) of **33** (*see procedure*)

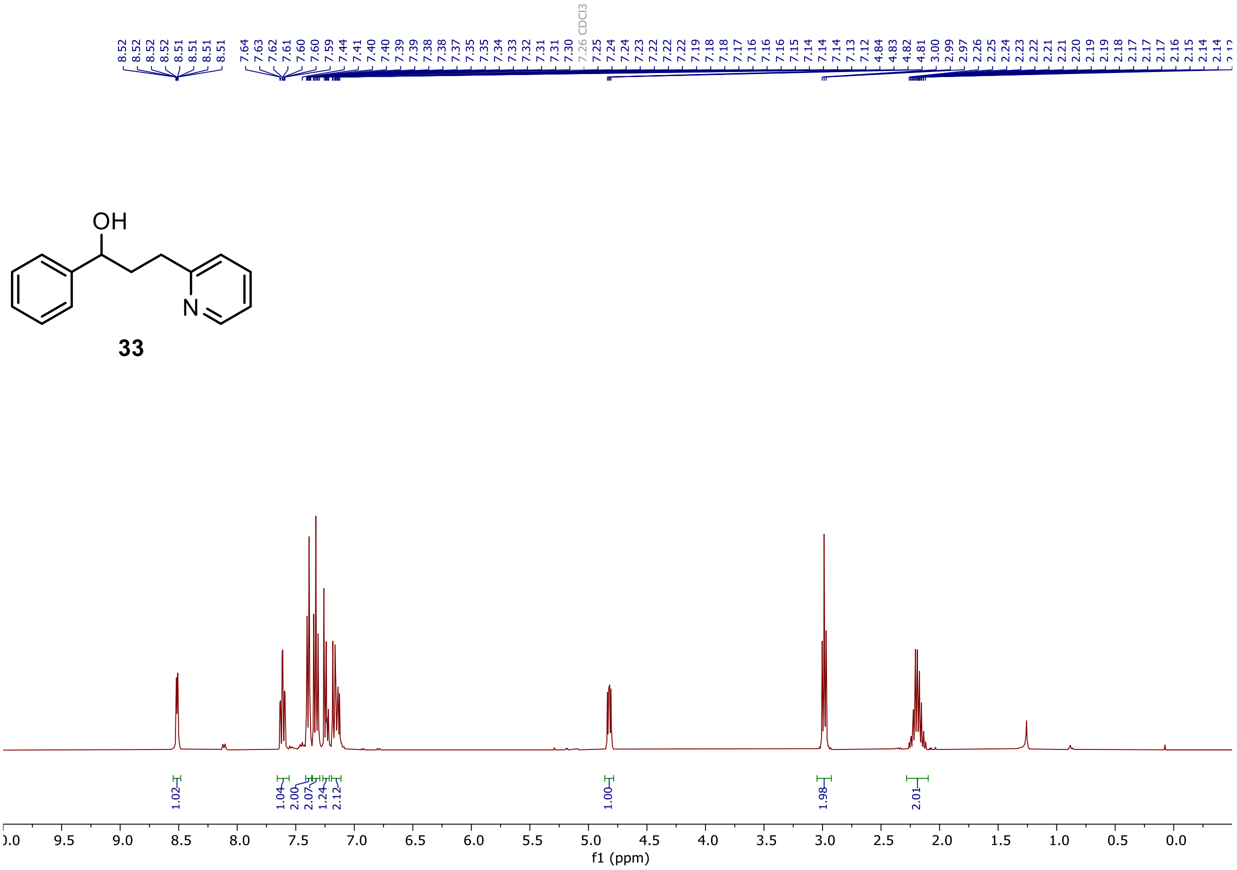

^{13}C NMR (101 MHz, $CDCl_3$) of **33**

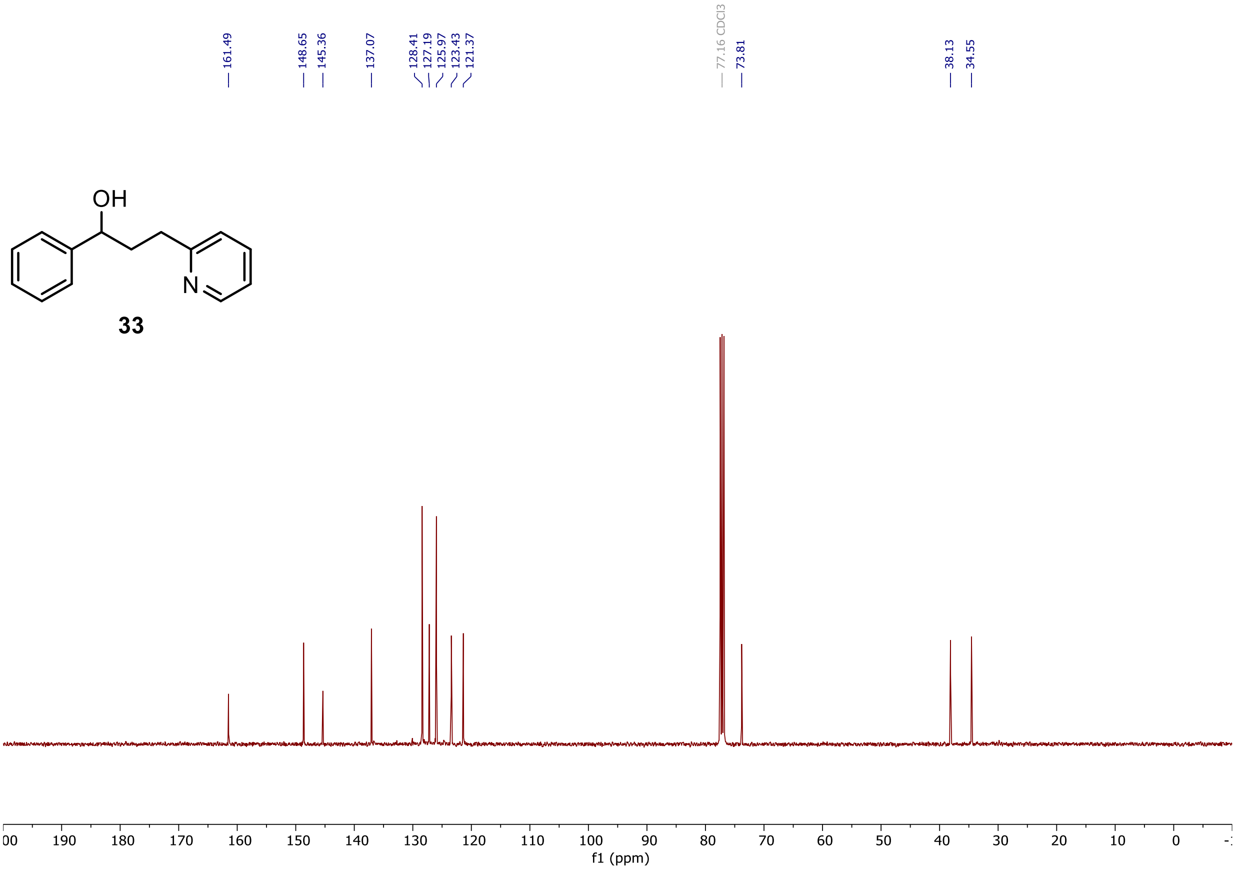

^{1}H NMR (400 MHz, $CDCl_3$) of **34** (*see procedure*)

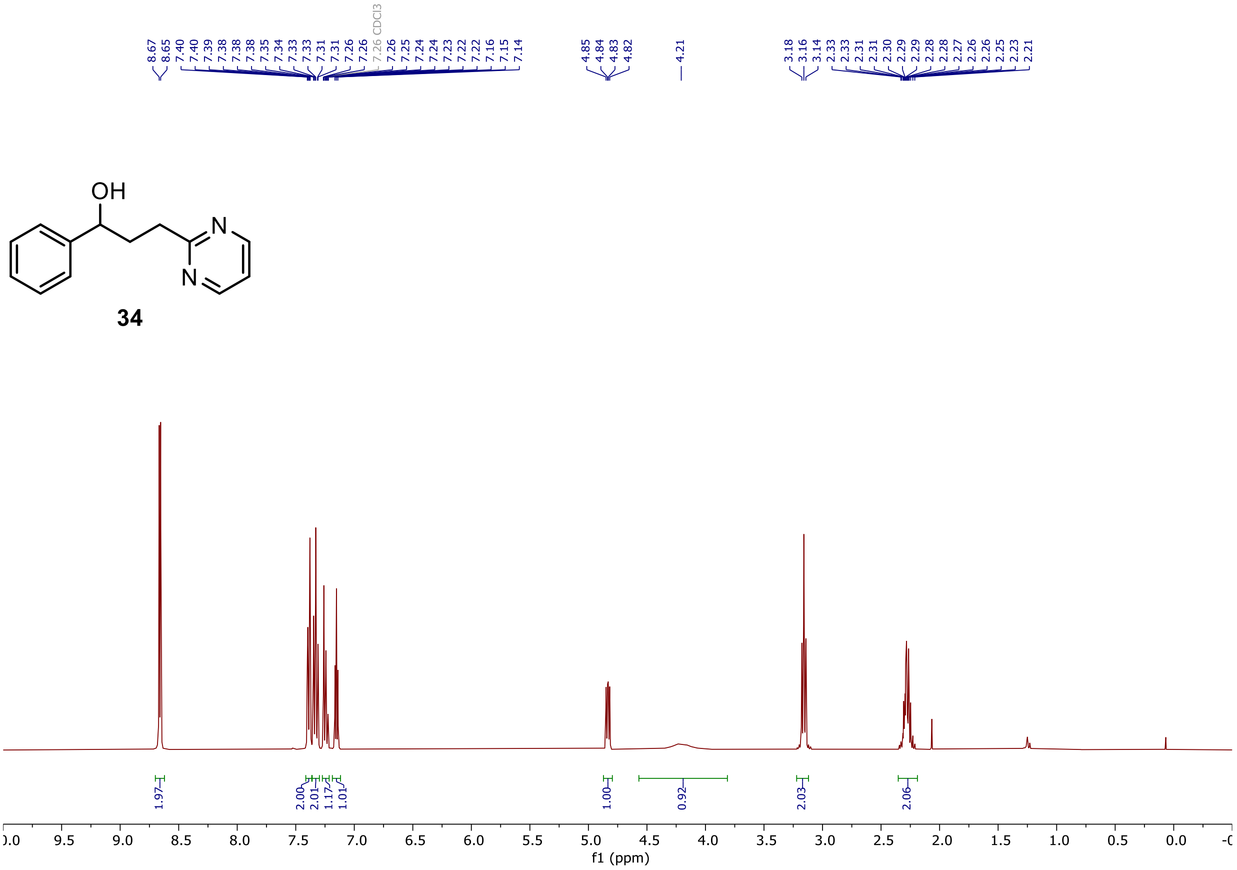

^{13}C NMR (101 MHz, $CDCl_3$) of **34**

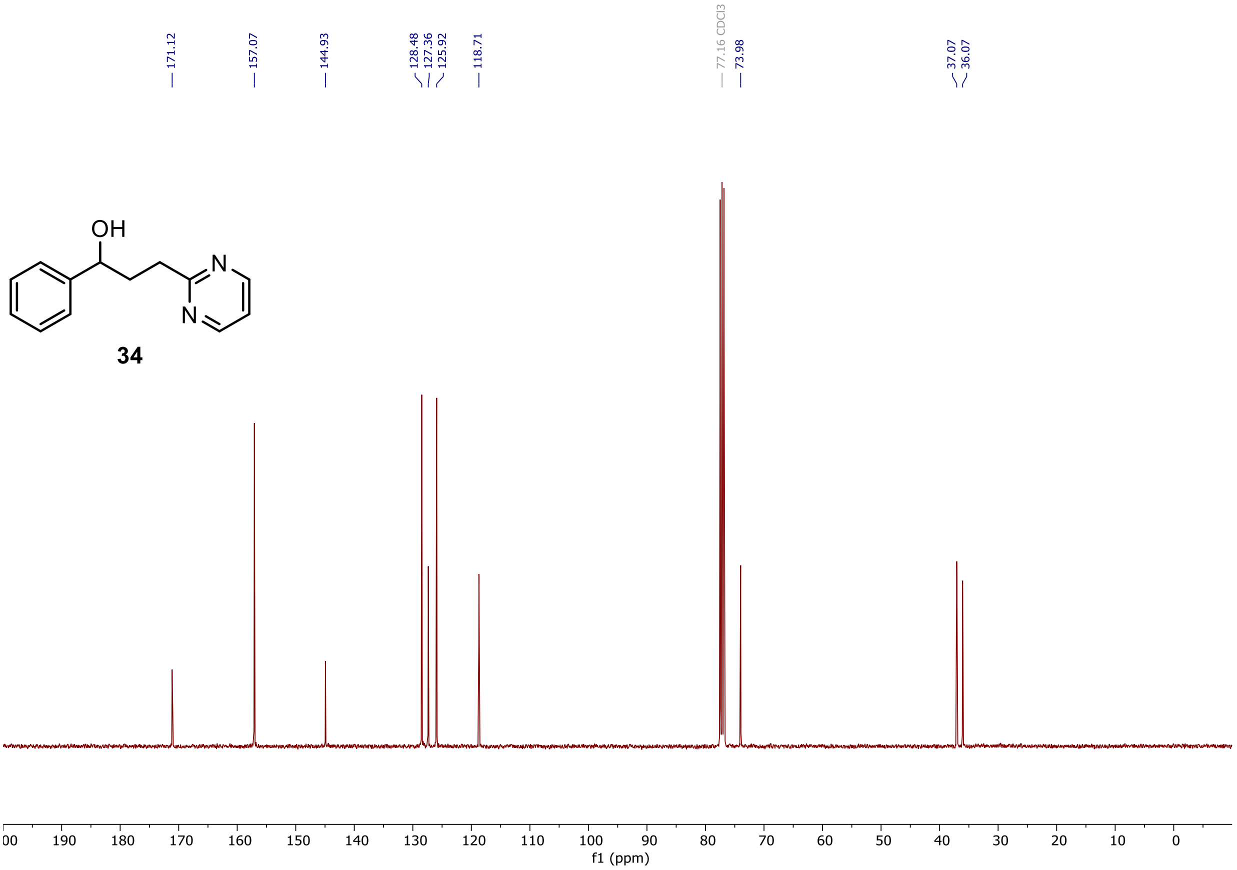

^{1}H NMR (400 MHz, $CDCl_3$) of **35** (*see procedure*)

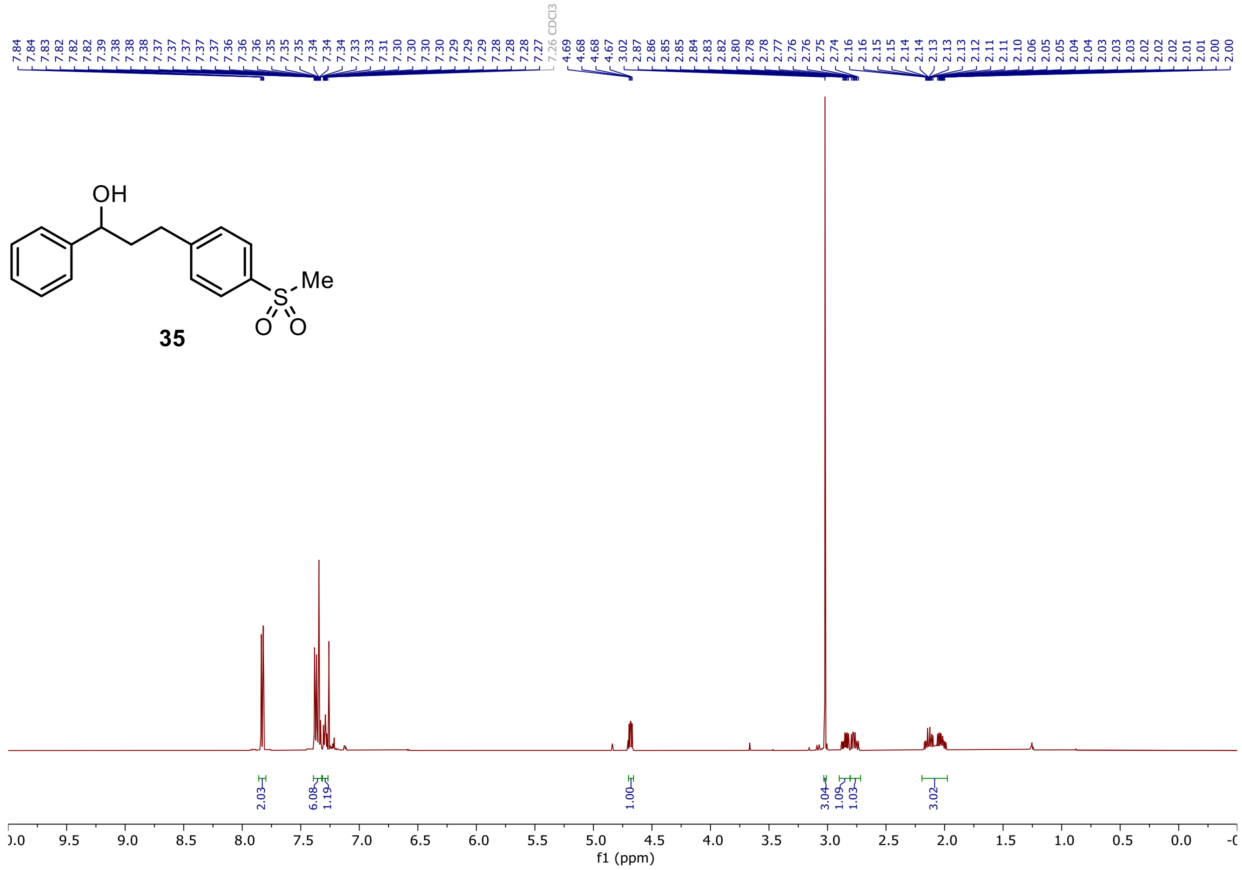

^{13}C NMR (101 MHz, $CDCl_3$) of **35**

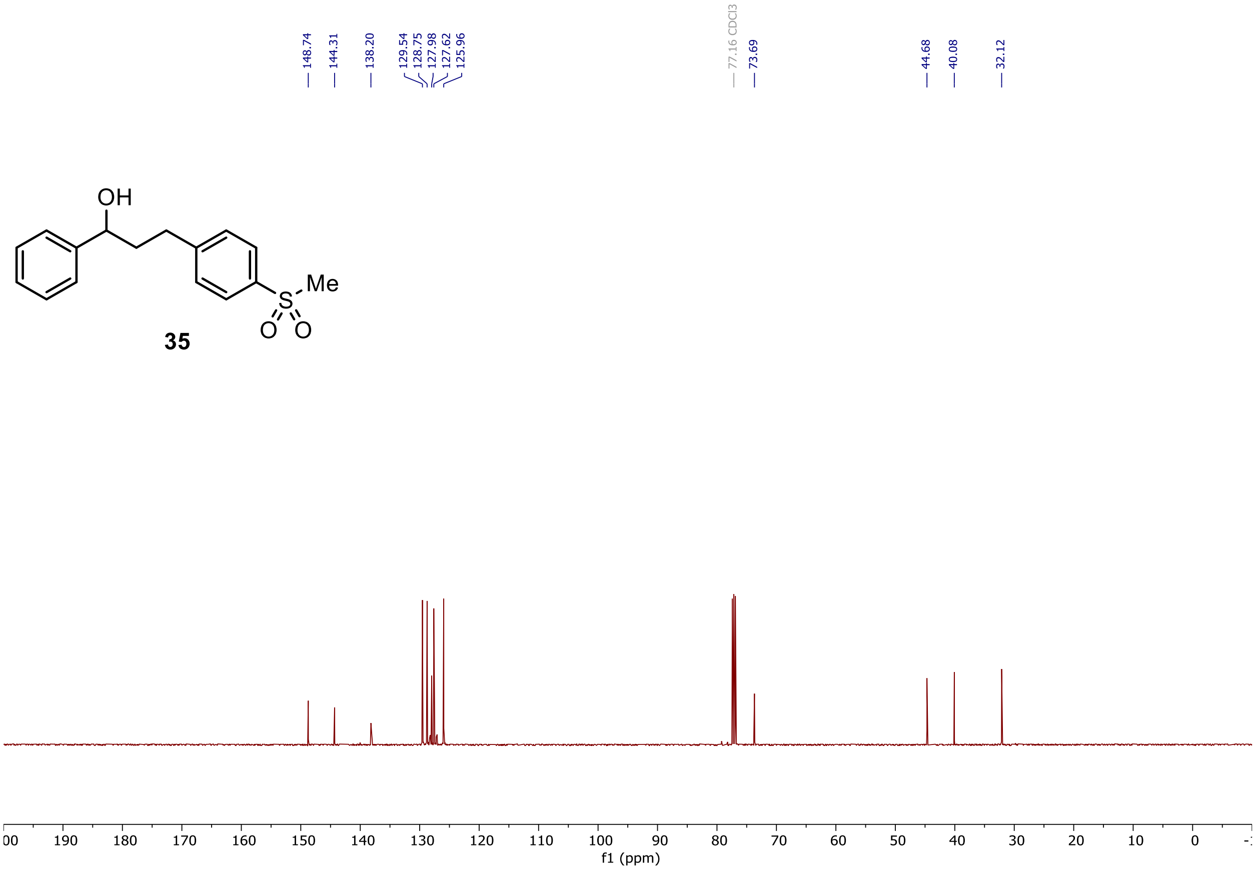

^{1}H NMR (400 MHz, $CDCl_3$) of **36** (*see procedure*)

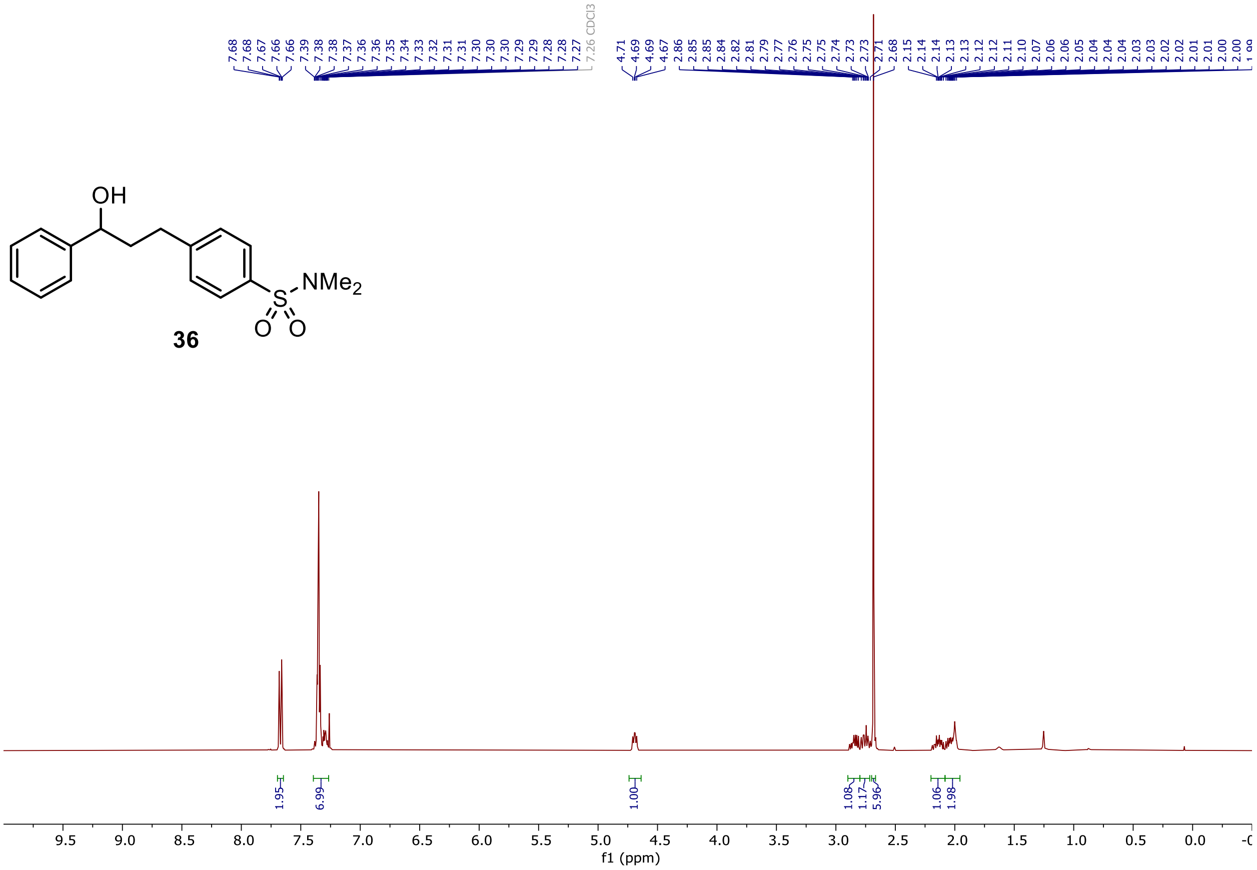

^{13}C NMR (126 MHz, $CDCl_3$) of **36**

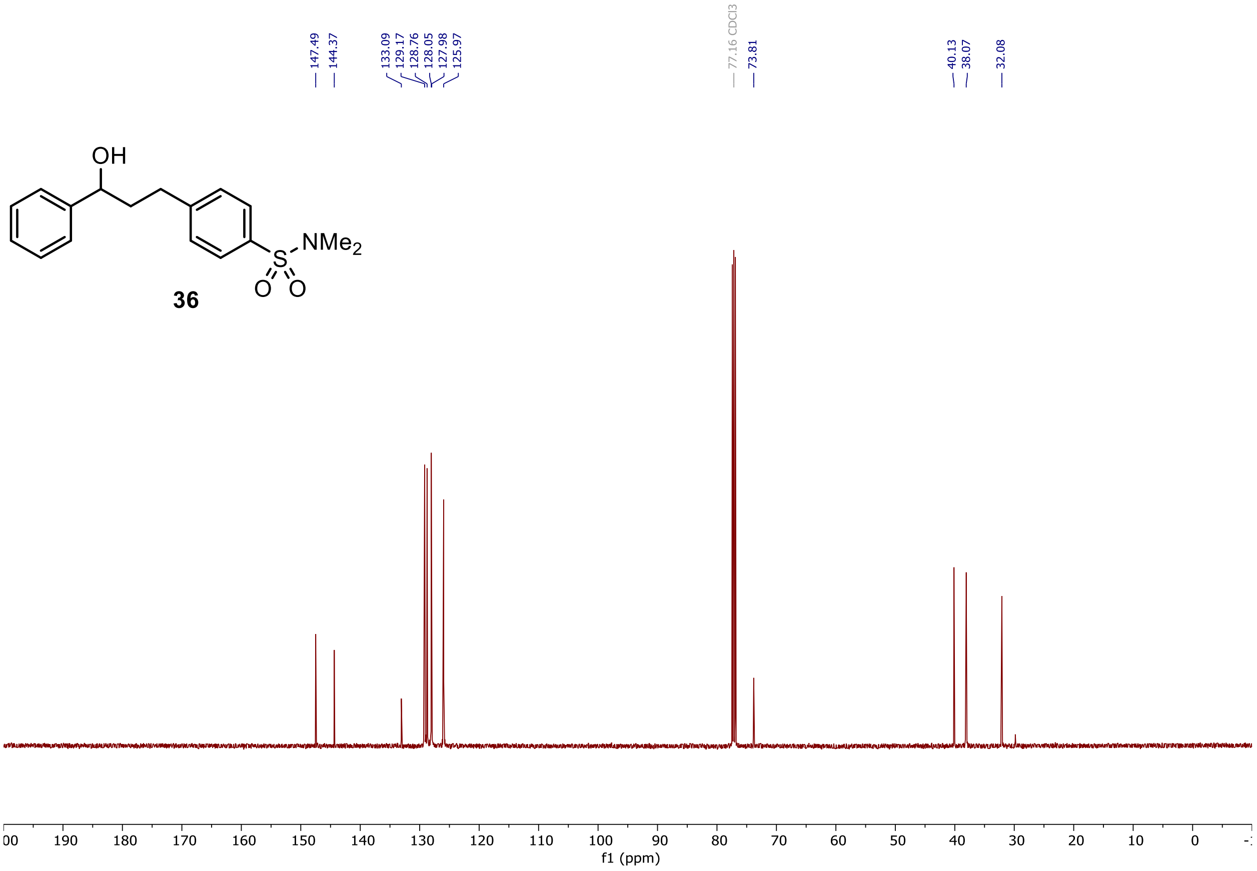

^{1}H NMR (400 MHz, $CDCl_3$) of **37** (*see procedure*)

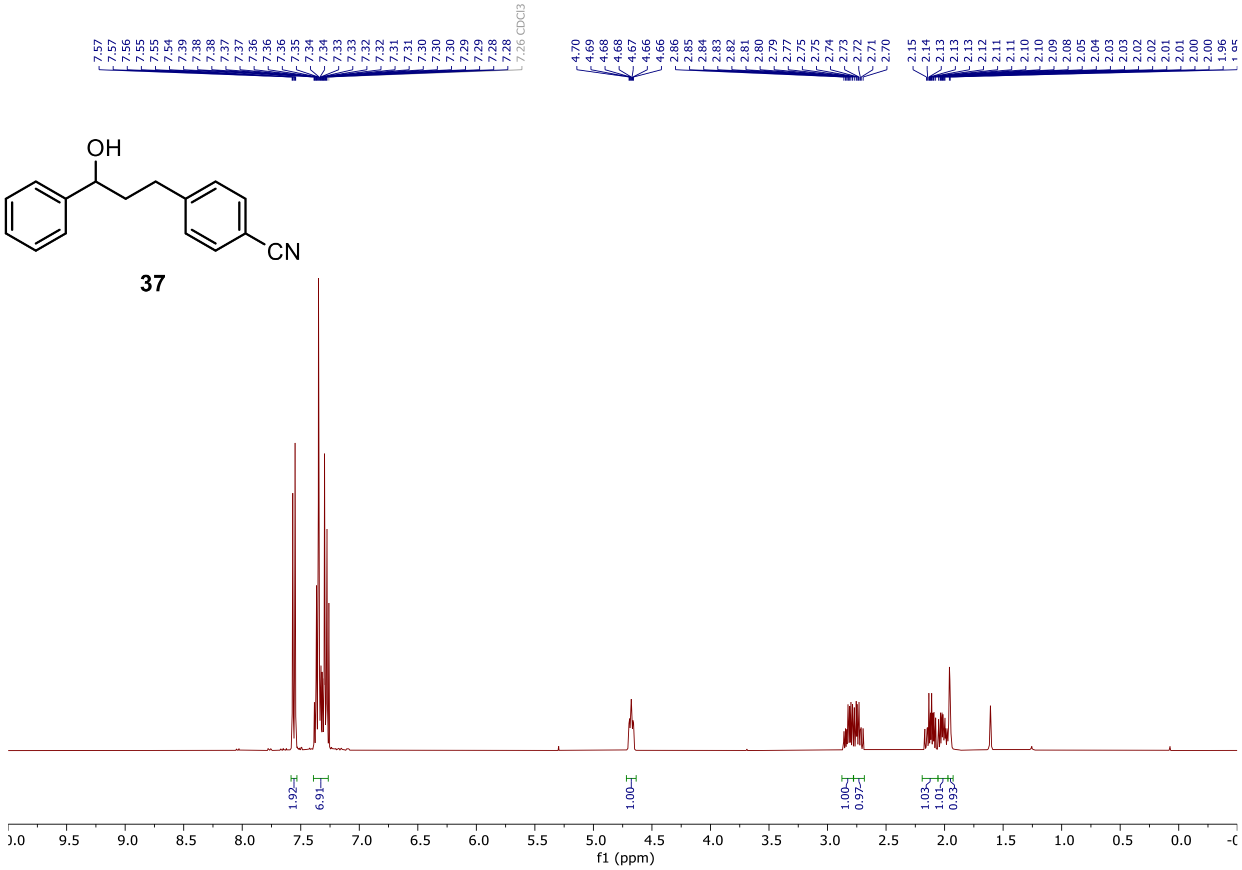

^{13}C NMR (101 MHz, $CDCl_3$) of **37**

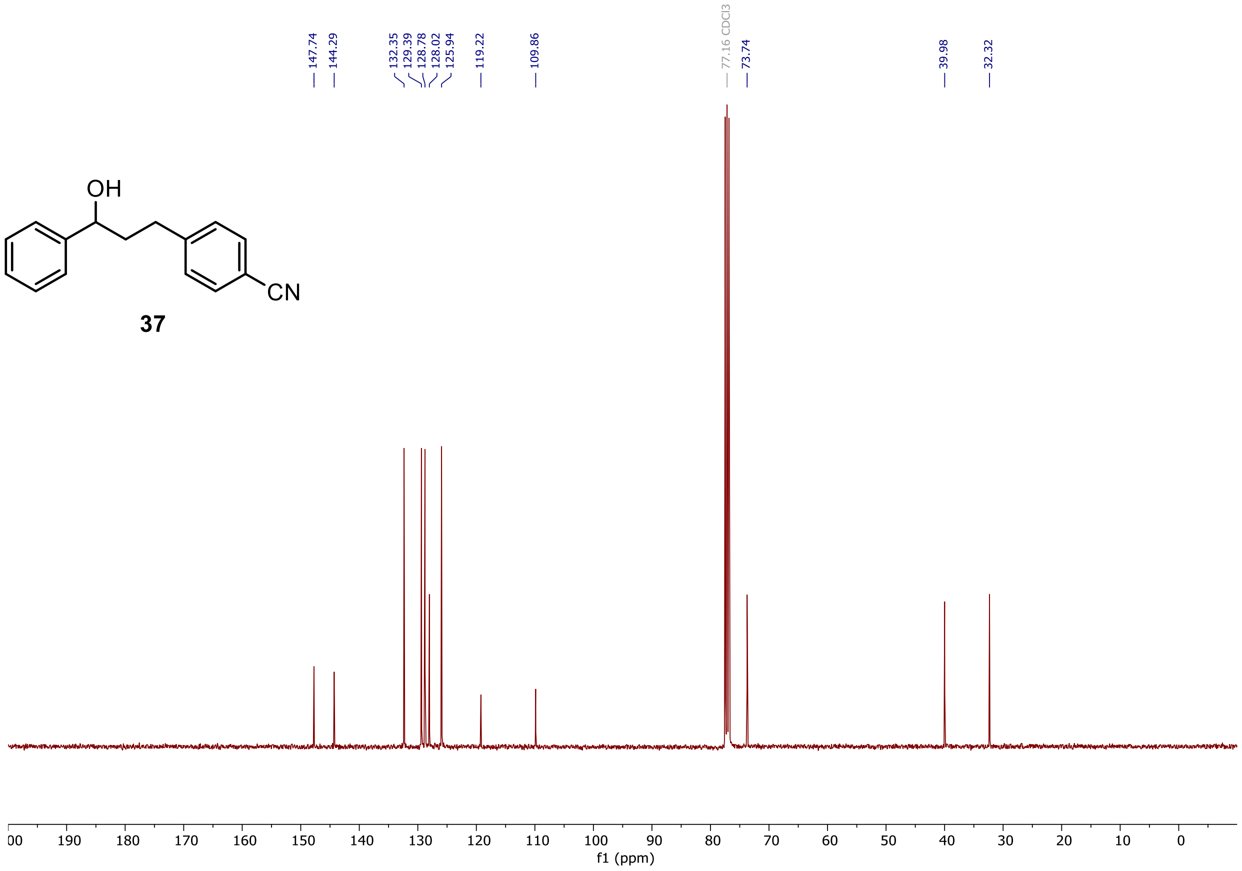

^{1}H NMR (400 MHz, $CDCl_3$) of **39** (*see procedure*)

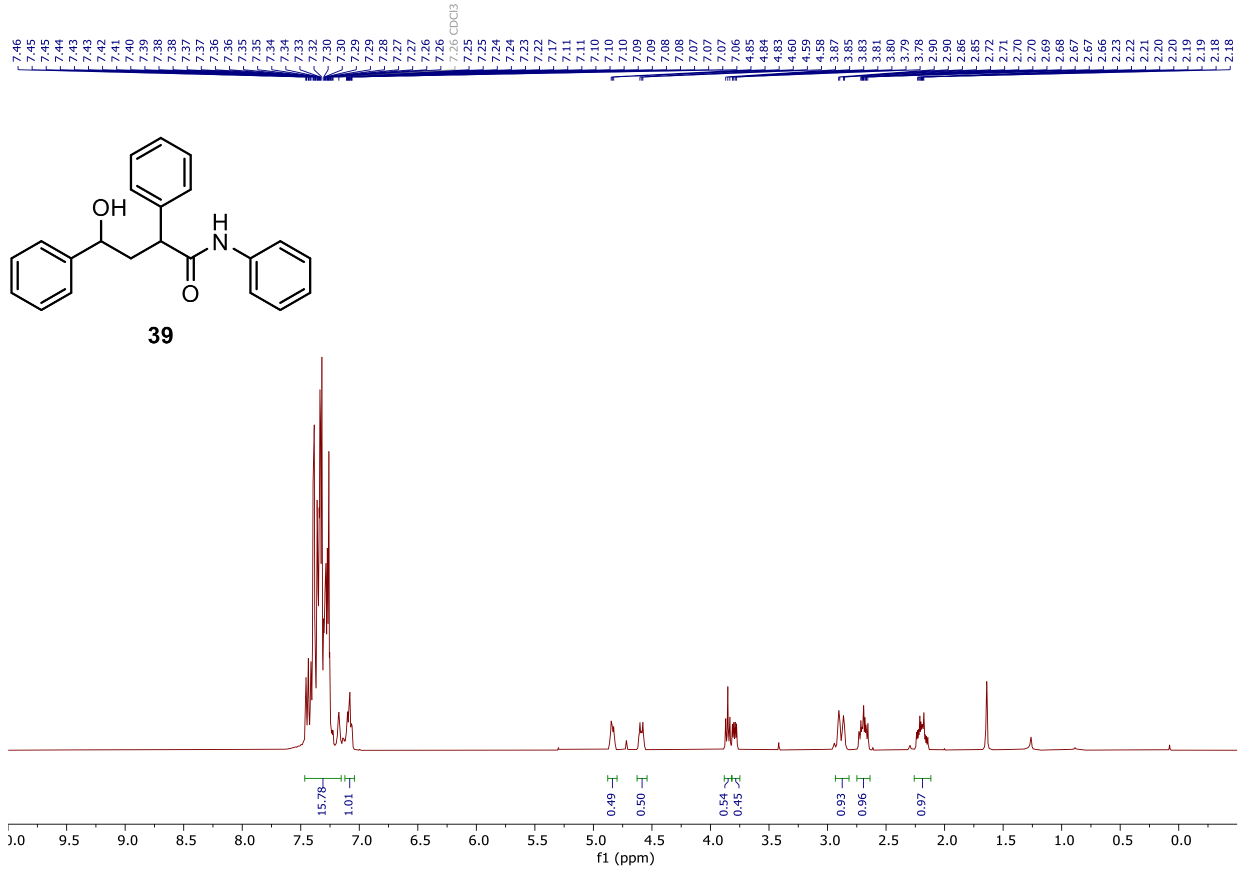

^{13}C NMR (126 MHz, $CDCl_3$) of **39**

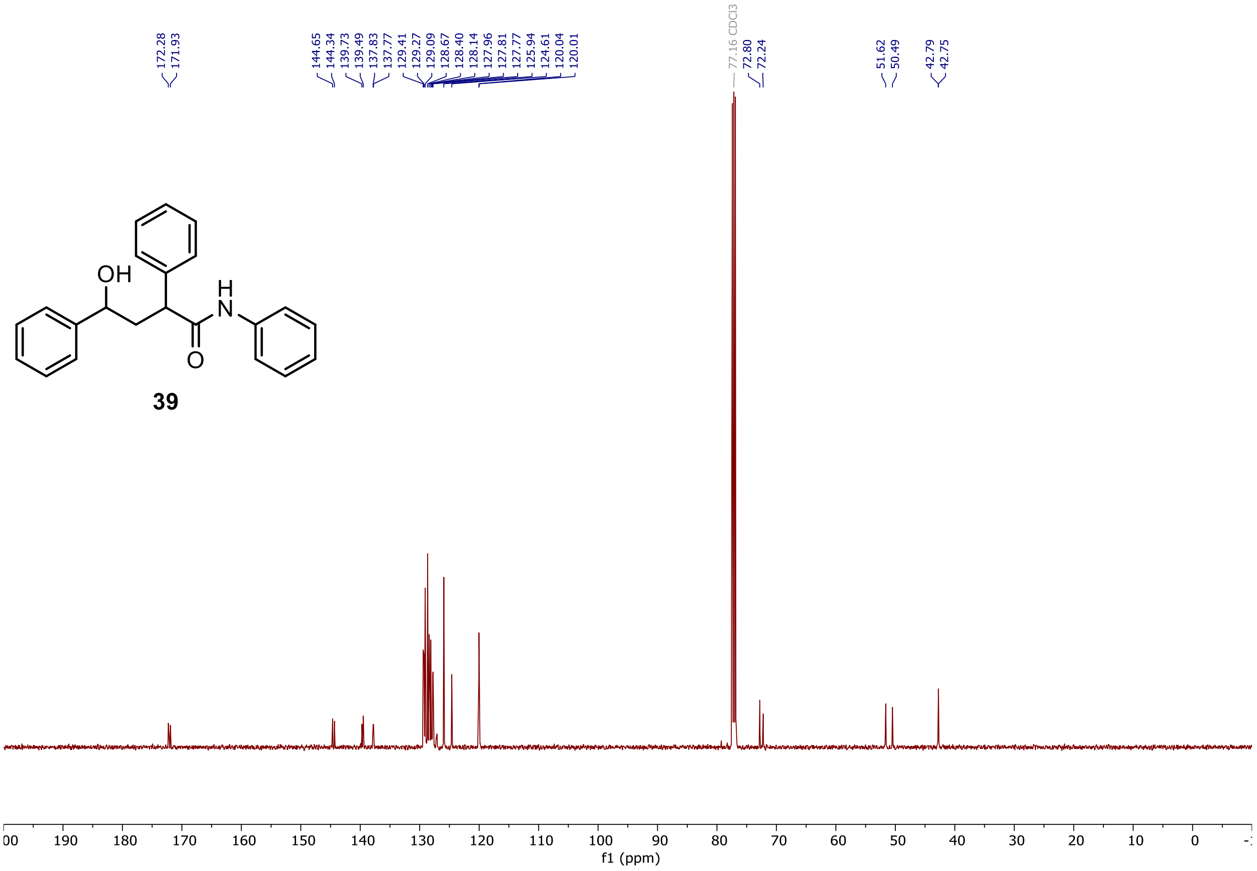

^{1}H NMR (400 MHz, $CDCl_3$) of **40** (*see procedure*)

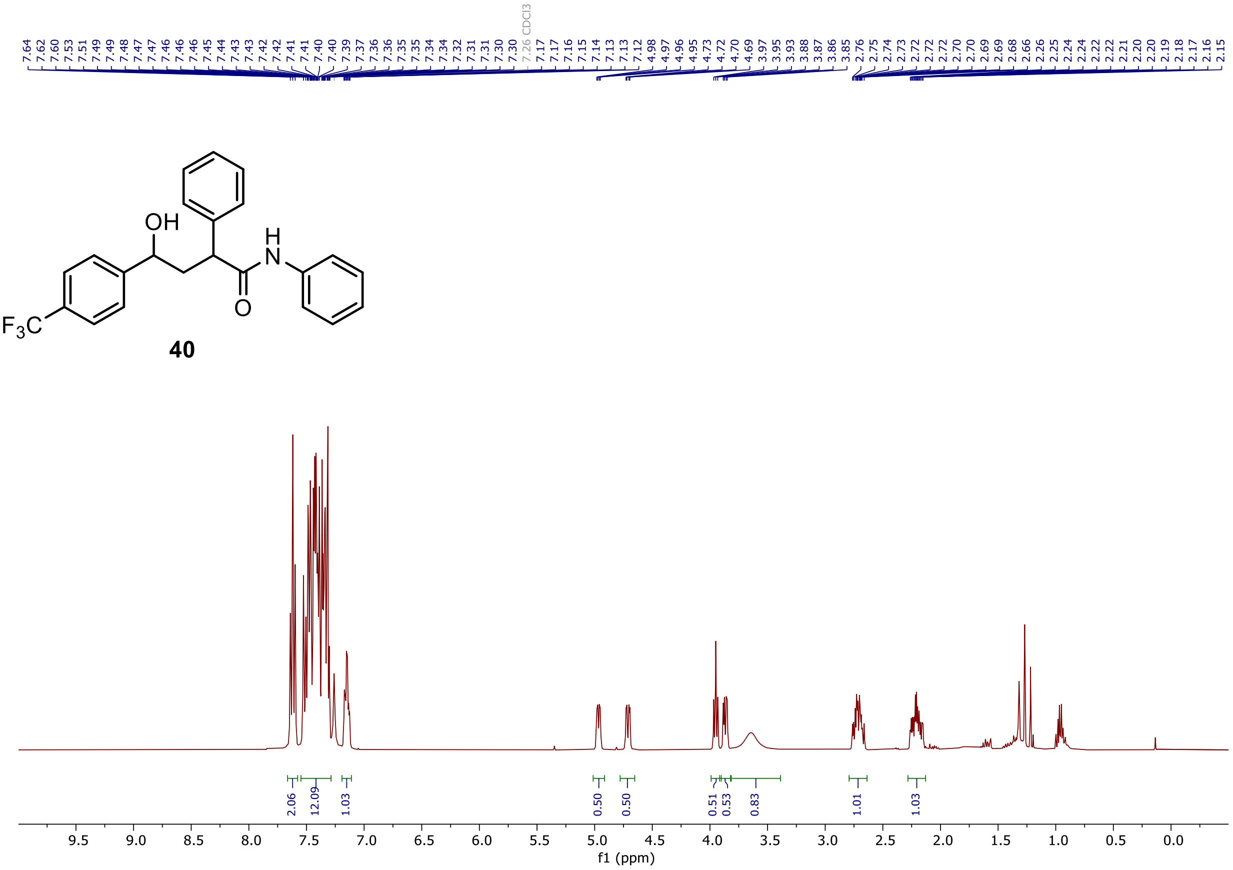

^{13}C NMR (126 MHz, $CDCl_3$) of **40**

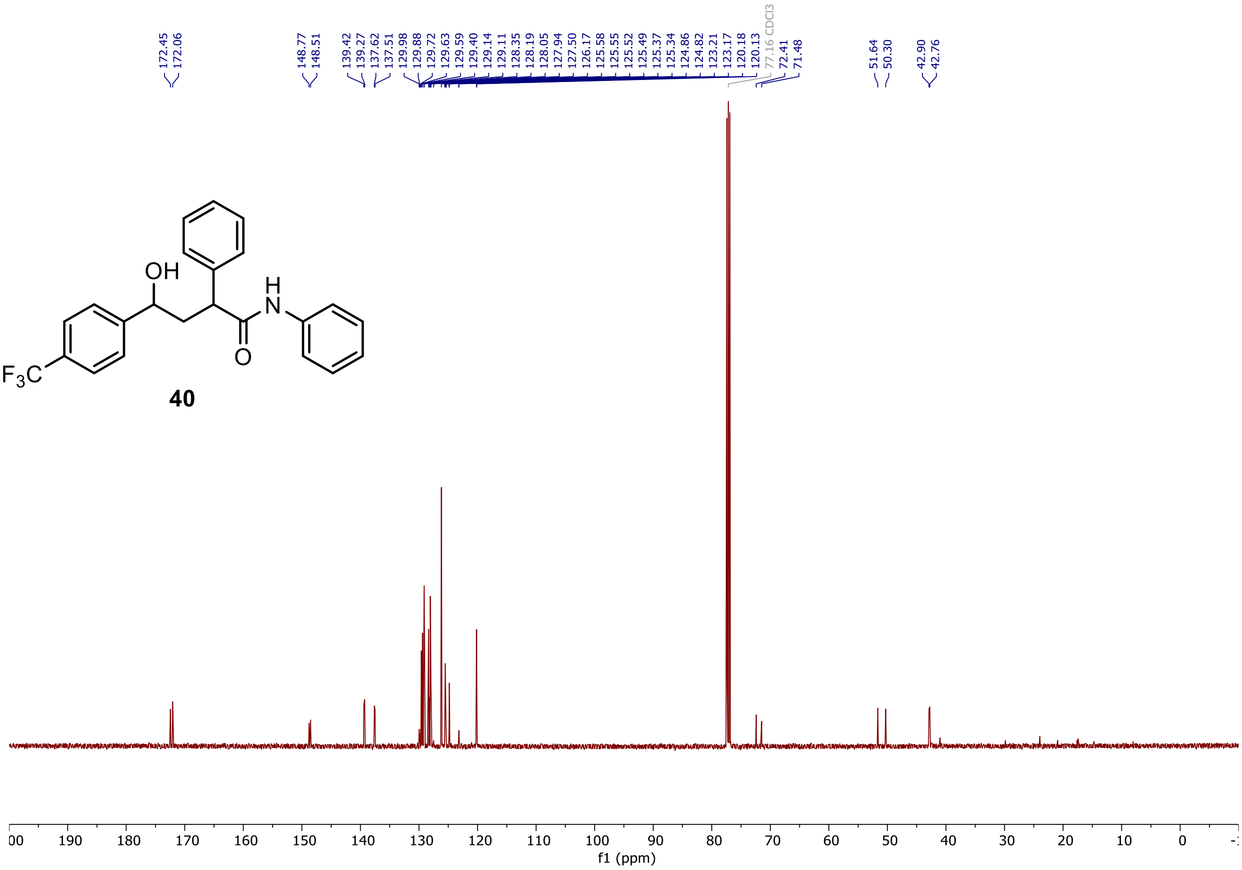

^{19}F NMR (377 MHz, $CDCl_3$) of **40**

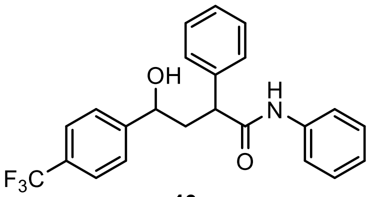

40

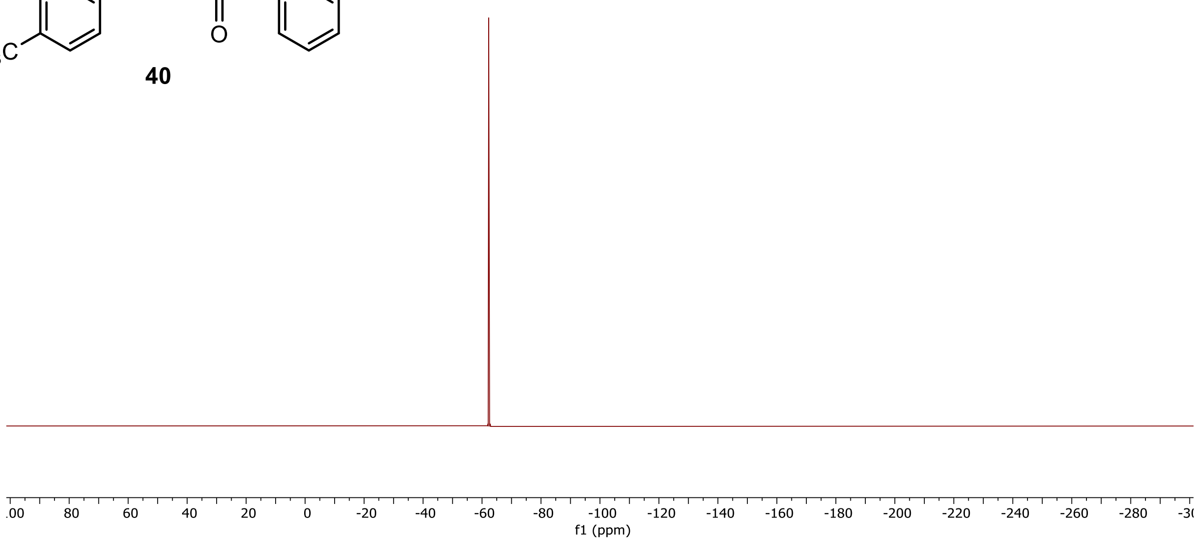

^{1}H NMR (400 MHz, $CDCl_3$) of **41** (*see procedure*)

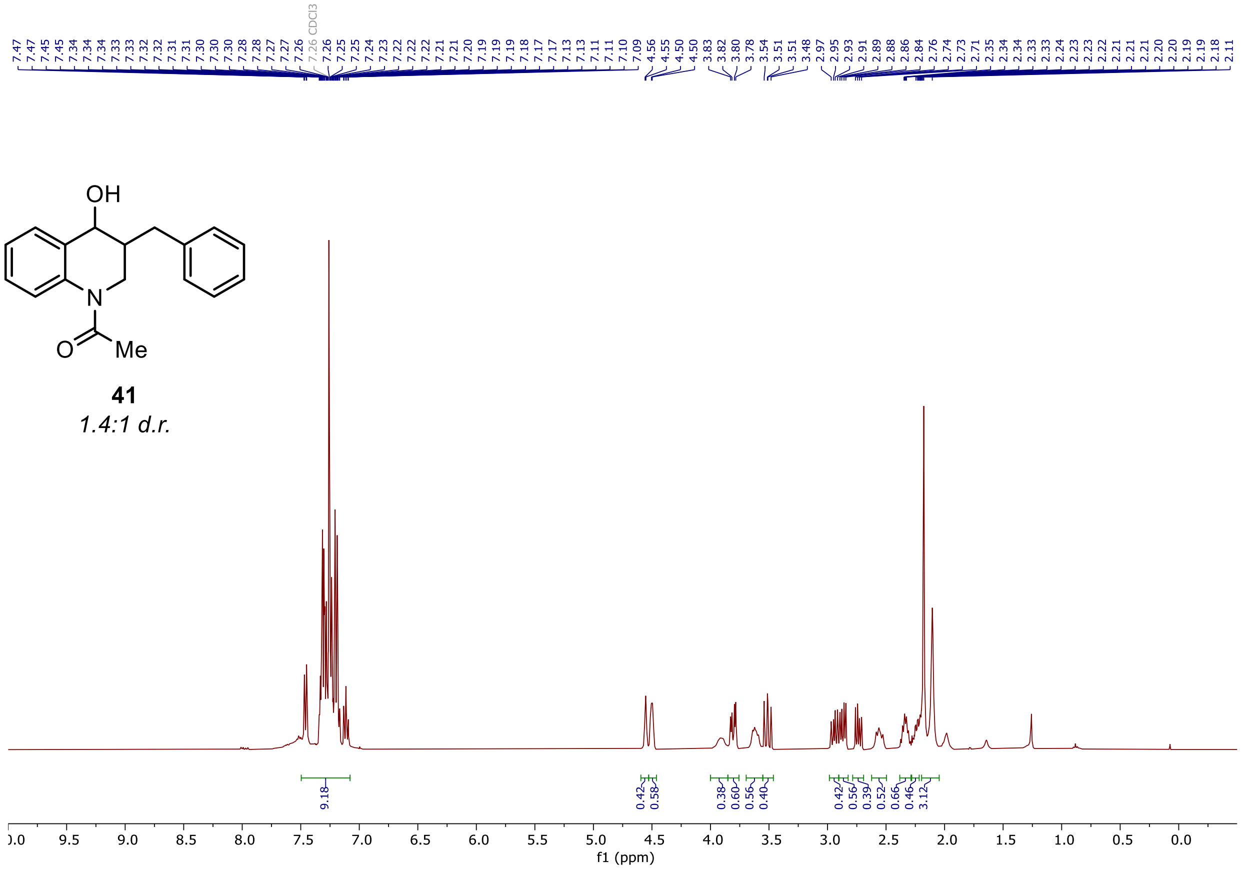

^{13}C NMR (101 MHz, $CDCl_3$) of **41**

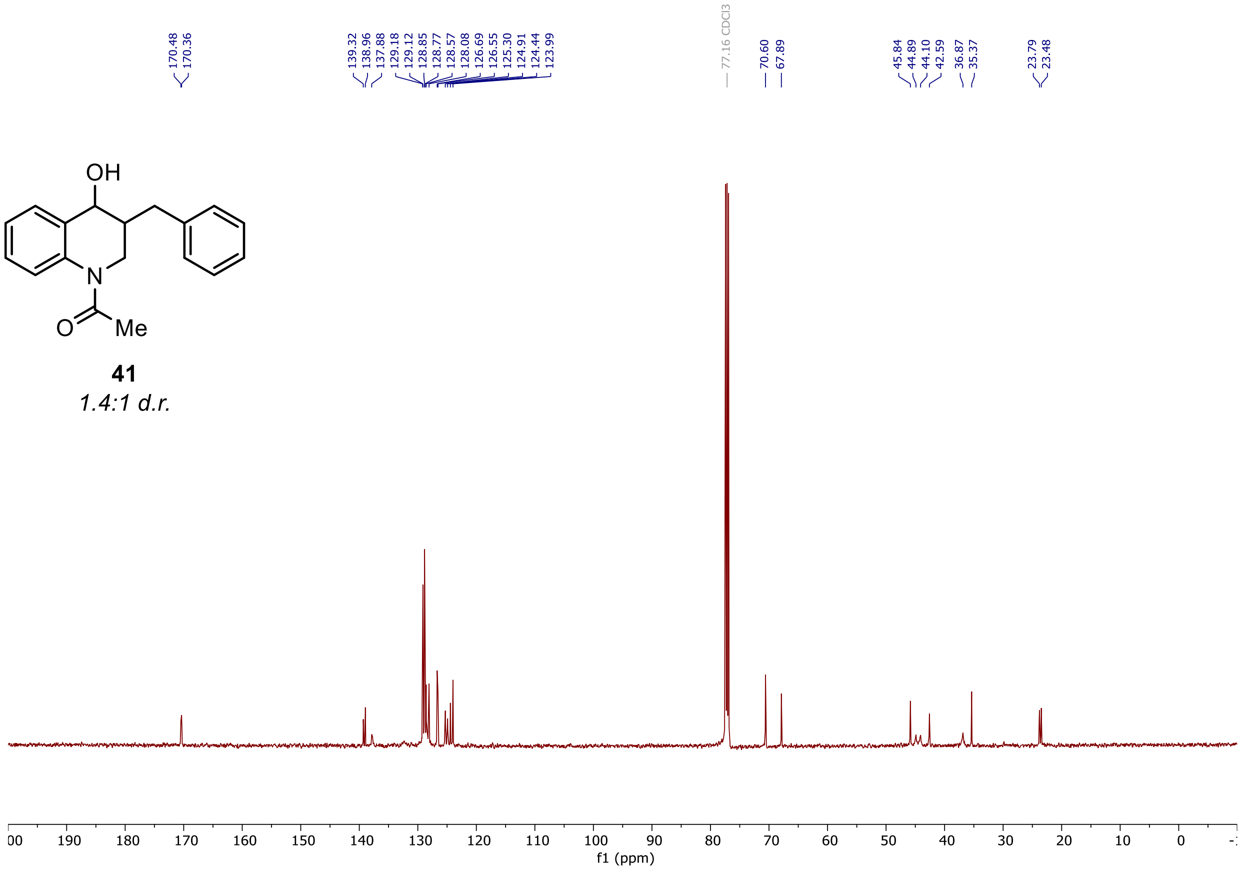

^{1}H NMR (400 MHz, $CDCl_3$) of **42** (see procedure)

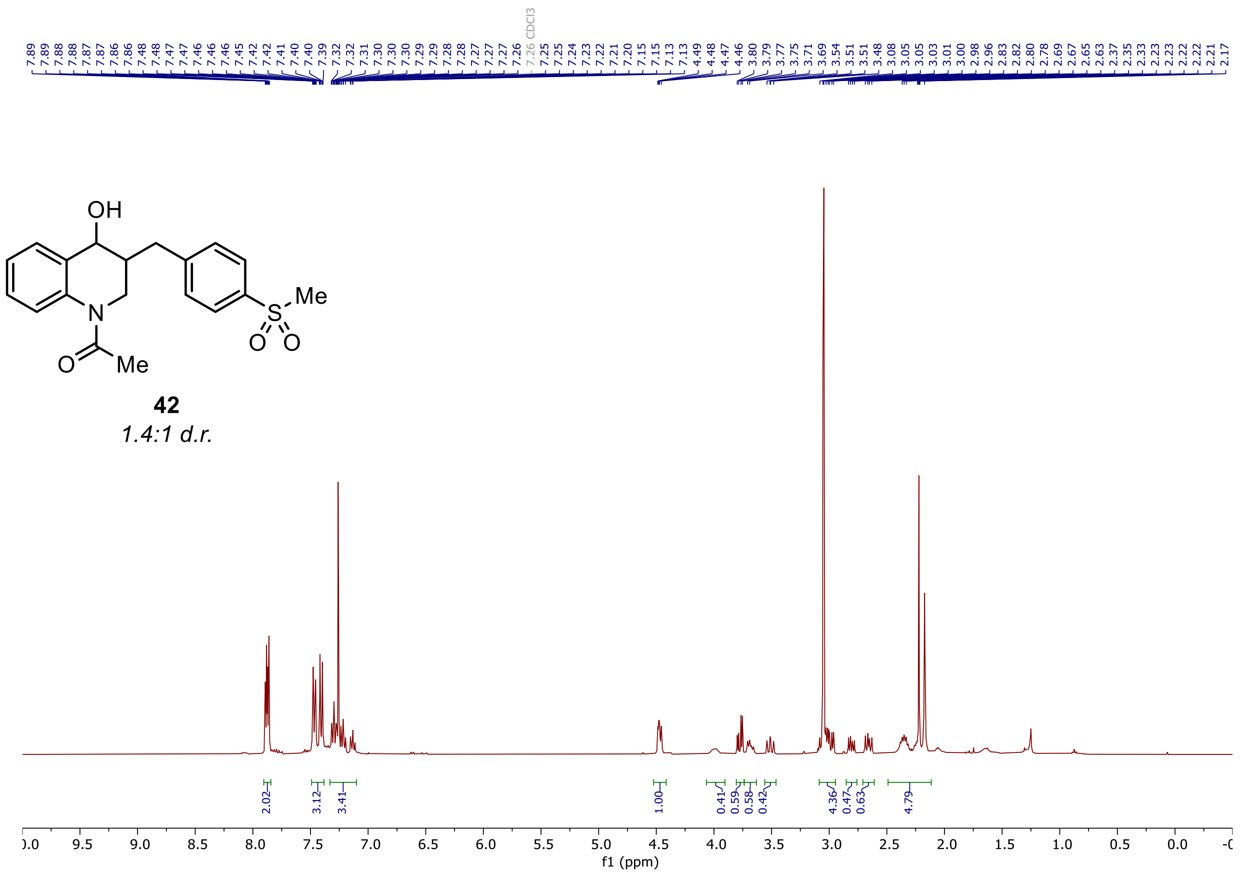

^{13}C NMR (101 MHz, $CDCl_3$) of **42**

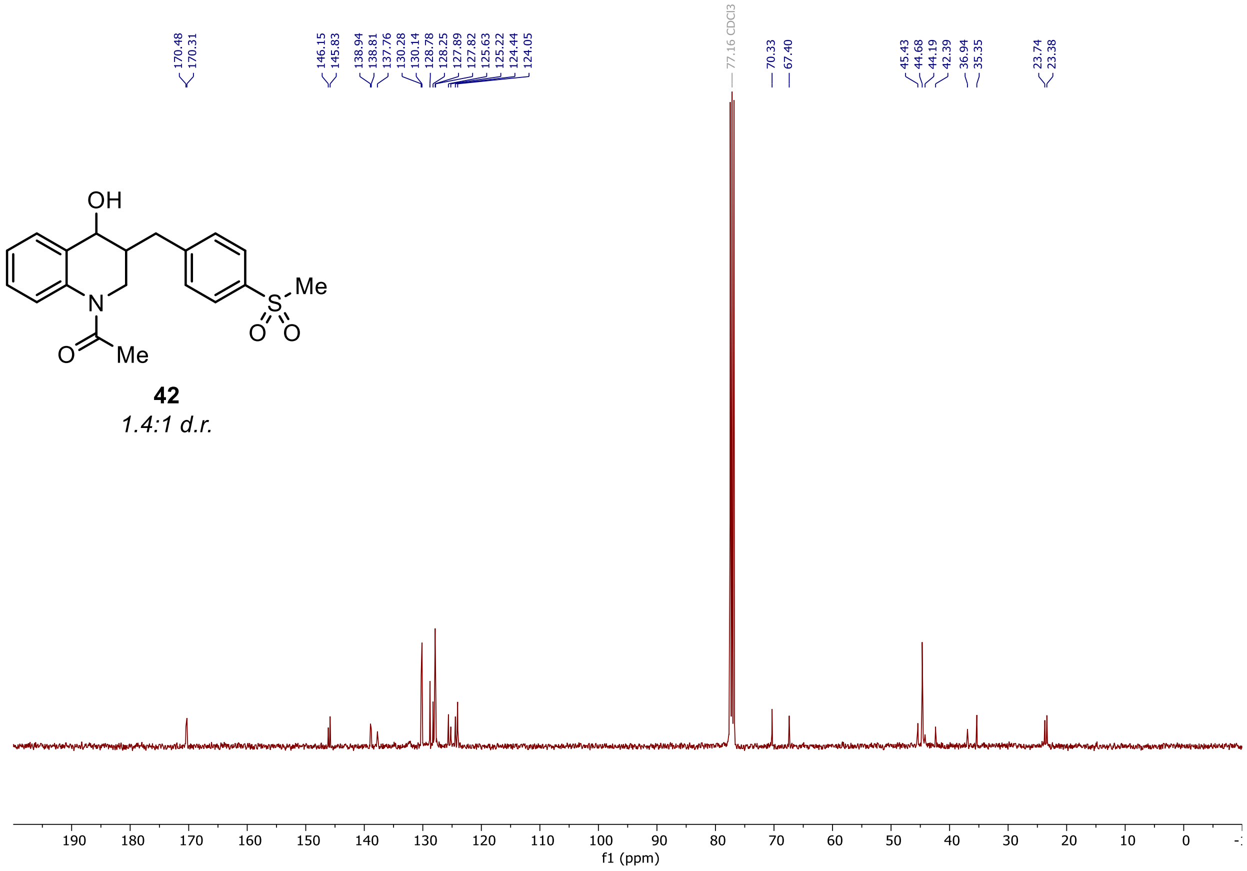

^{1}H NMR (400 MHz, $CDCl_3$) of **43** (*see procedure*)

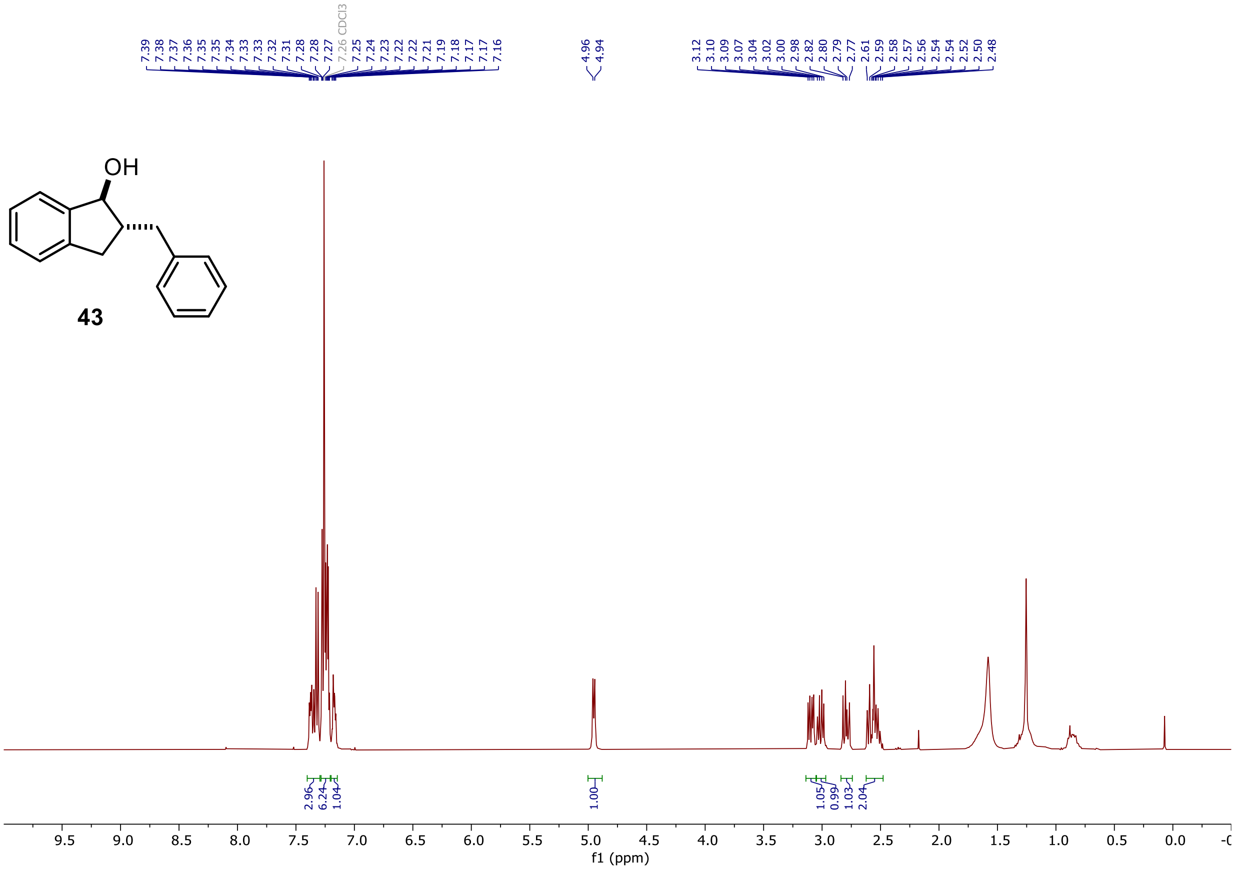

^{13}C NMR (101 MHz, $CDCl_3$) of **43**

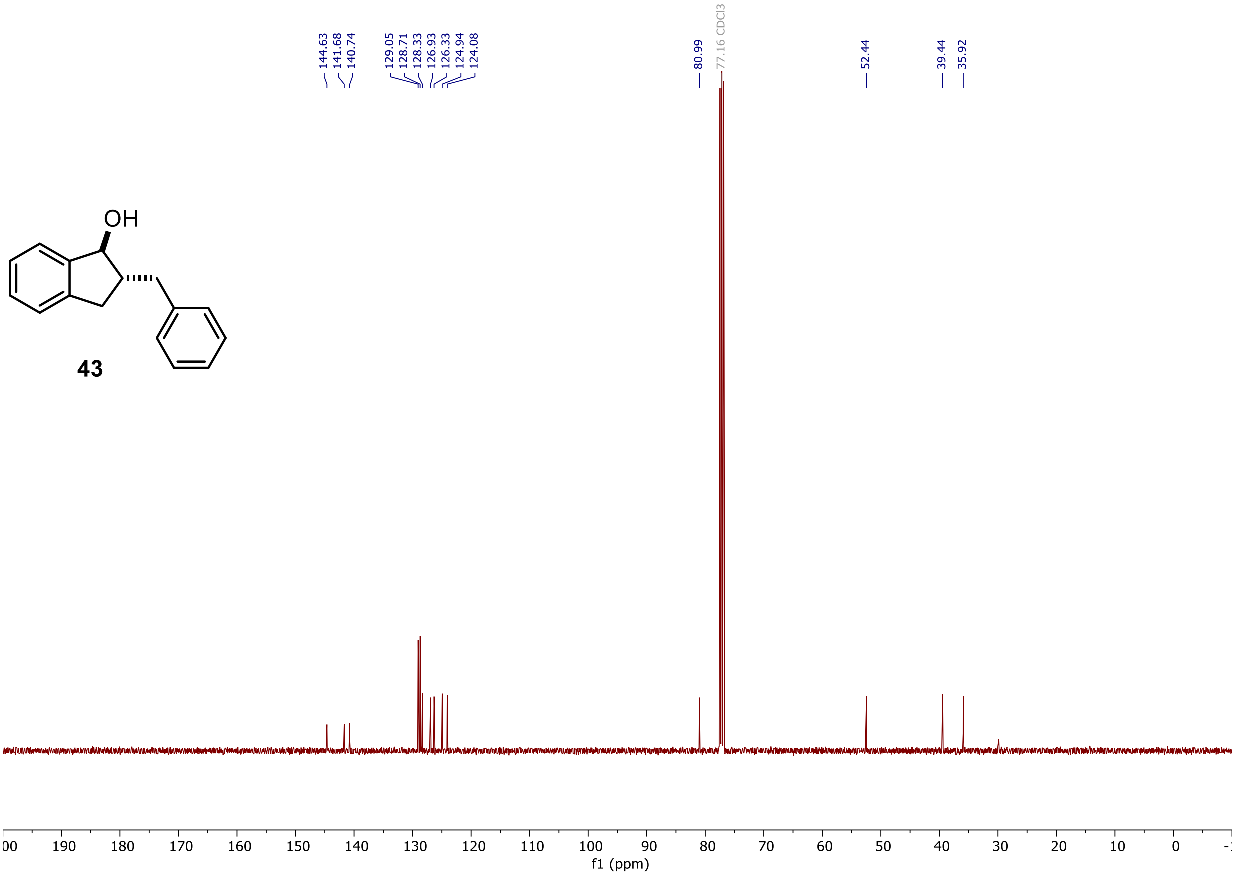

^{1}H NMR (400 MHz, CDCl$_3$) of **44** (see procedure)

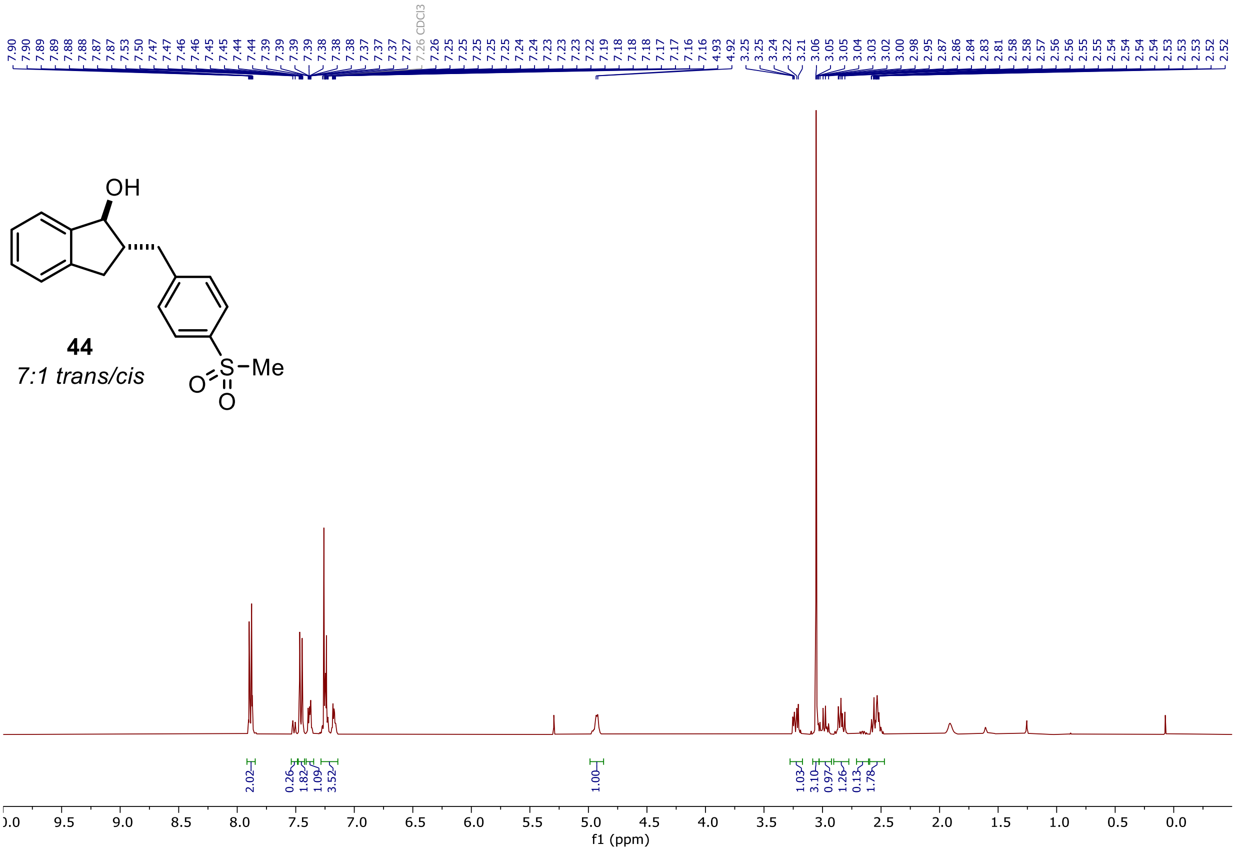

^{13}C NMR (101 MHz, CDCl$_3$) of **44**

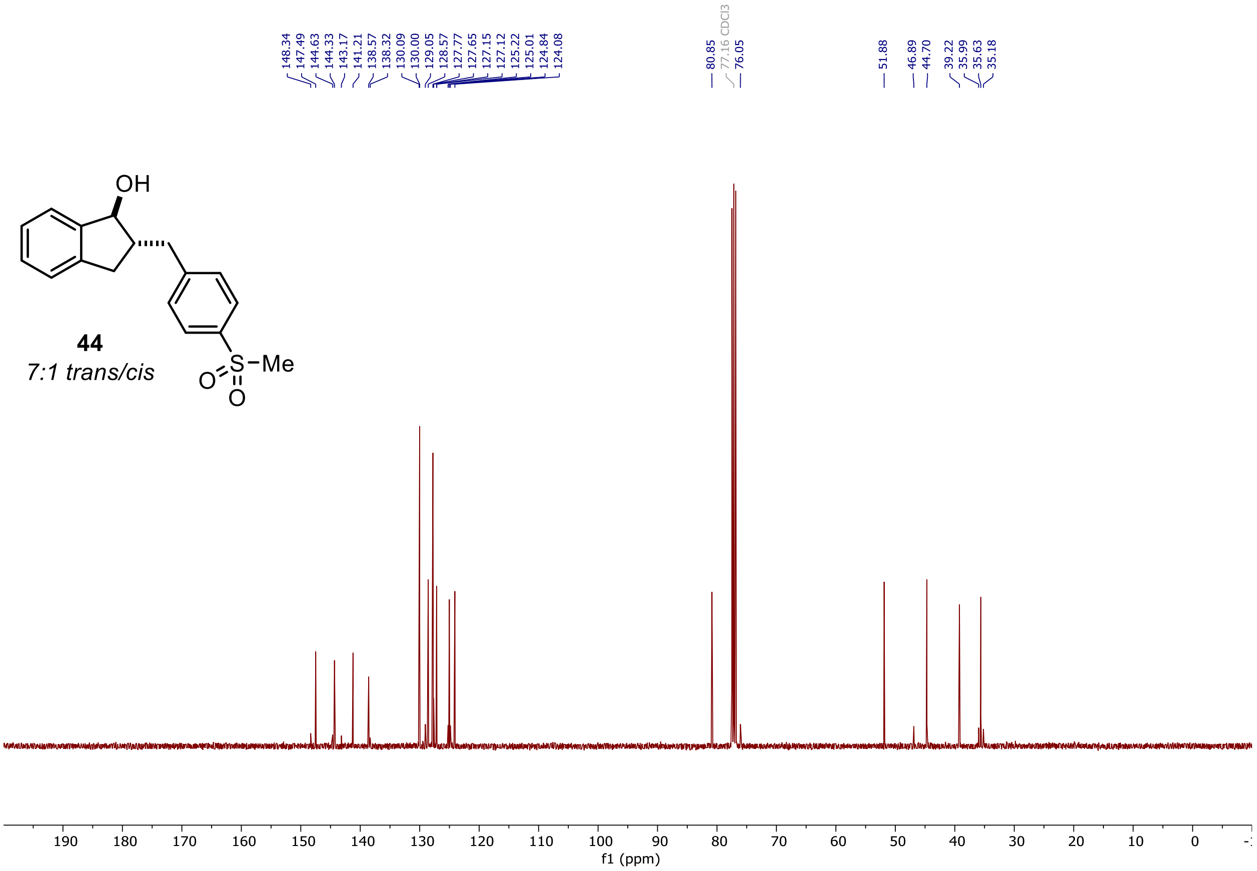

^{1}H NMR (400 MHz, $CDCl_3$) of **46** (see procedure)

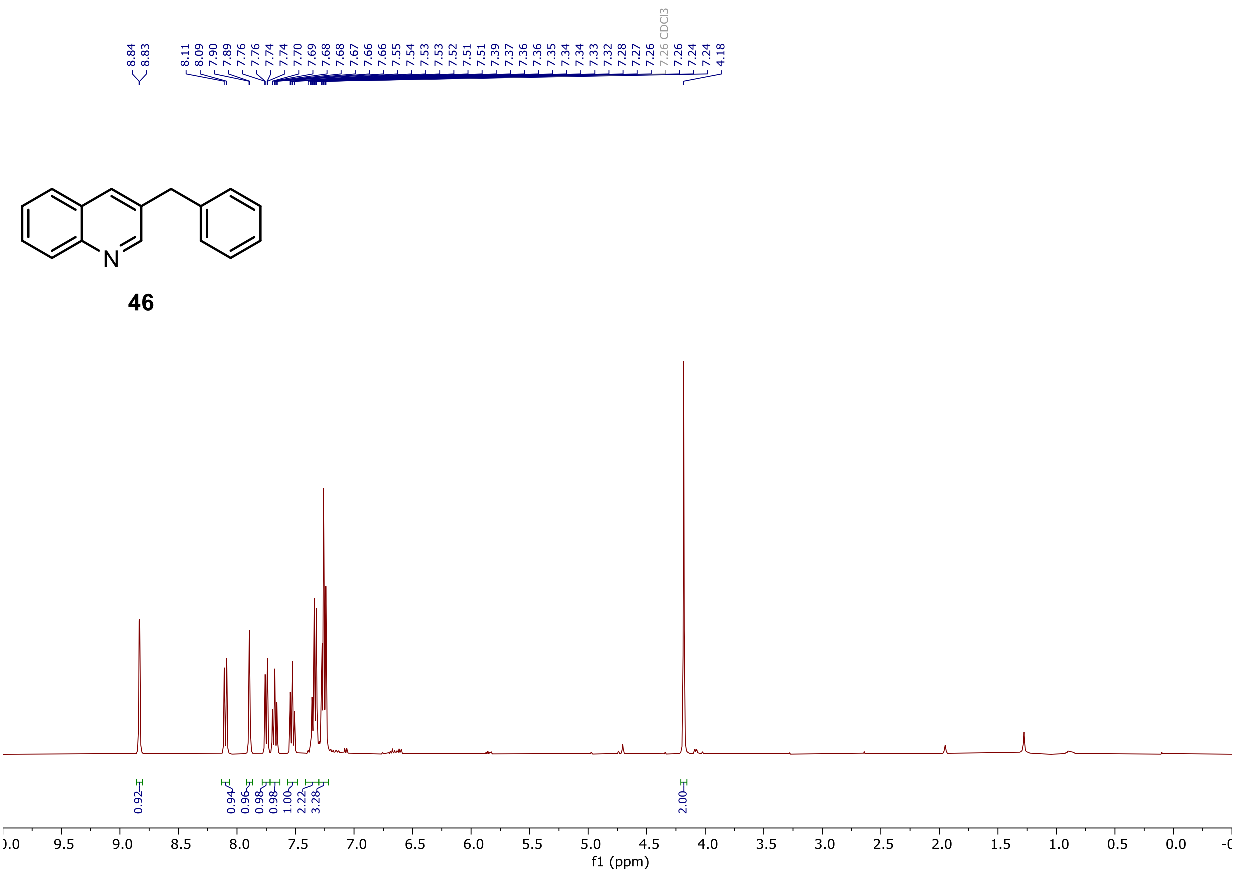

^{13}C NMR (101 MHz, $CDCl_3$) of **46**

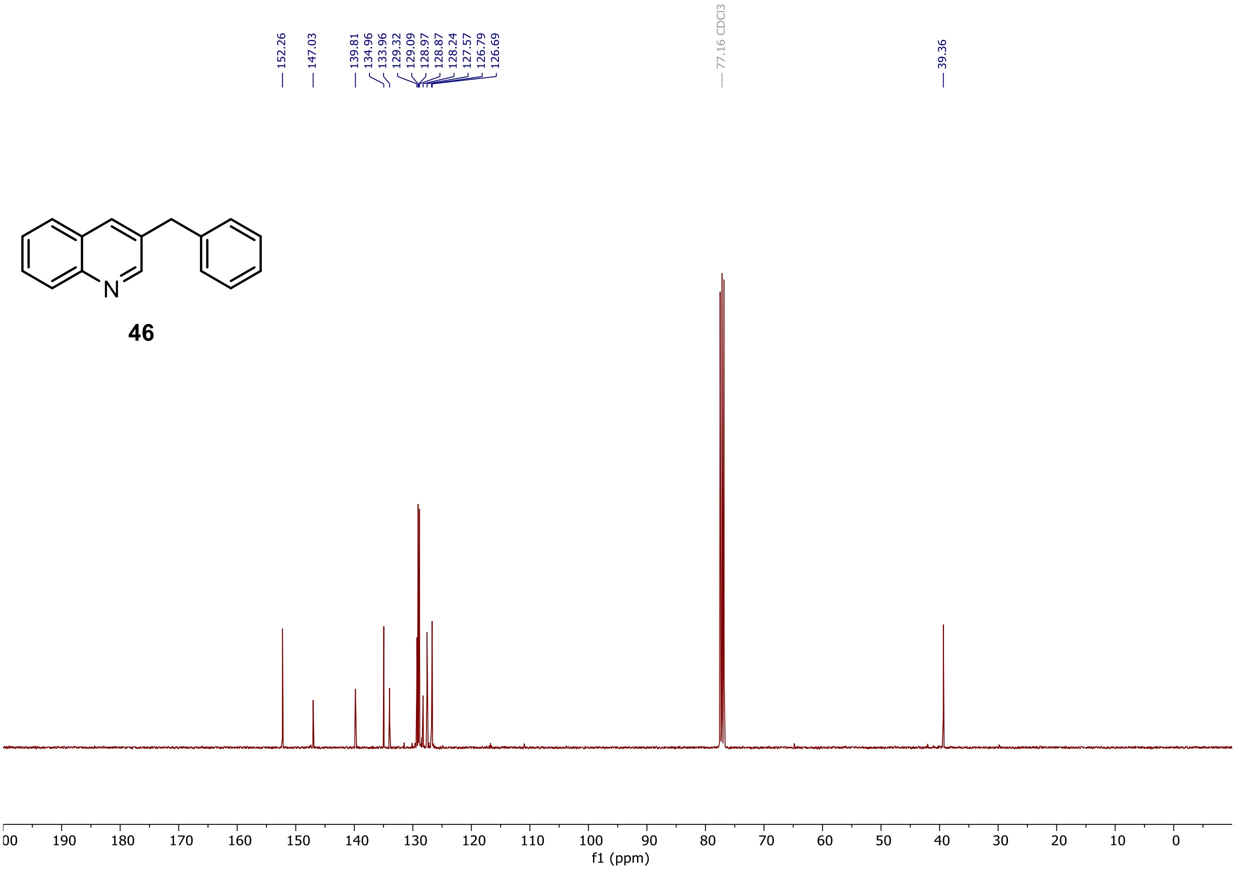

5. REFERENCES

(1) Constantin, T.; Zanini, M.; Regni, A.; Sheikh, N. S.; Juliá, F.; Leonori, D. Aminoalkyl Radicals as Halogen-Atom Transfer Agents for Activation of Alkyl and Aryl Halides. *Science* **2020**, *367*, 1021–1026.

(2) Mulliez, M.; Naudy, C. Synthèse d'α-Hydroxysulfinates. *Tetrahedron* **1993**, *49*, 2469–2476. https://doi.org/10.1016/S0040-4020(01)86325-0.

(3) Castaing, M.; Wason, S. L.; Estepa, B.; Hooper, J. F.; Willis, M. C. 2-Aminobenzaldehydes as Versatile Substrates for Rhodium-Catalyzed Alkyne Hydroacylation: Application to Dihydroquinolone Synthesis. *Angew. Chem. Int. Ed.* **2013**, *52*, 13280–13283.

(4) Shi, J.; Wang, R. A.; Wu, W.; Song, J. R.; Chi, Q.; Pan, W. D.; Ren, H. Copper-Catalyzed Aerobic Selective Oxidation of Tetrahydrocarbolines. *Org. Lett.* **2022**, *24*, 3358–3362.

(5) Maisonial, A.; Kuhnast, B.; Papon, J.; Boisgard, R.; Bayle, M.; Vidal, A.; Auzeloux, P.; Rbah, L.; Bonnet-Duquennoy, M.; Miot-Noirault, E.; Galmier, M. J.; Borel, M.; Askienazy, S.; Dollé, F.; Tavitian, B.; Madelmont, J. C.; Moins, N.; Chezal, J. M. Single Photon Emission Computed Tomography/Positron Emission Tomography Imaging and Targeted Radionuclide Therapy of Melanoma: New Multimodal Fluorinated and Iodinated Radiotracers. *J. Med. Chem.* **2011**, *54*, 2745–2766.

(6) Yang, C. T.; Han, J.; Liu, J.; Li, Y.; Zhang, F.; Yu, H. Z.; Hu, S.; Wang, X. Pd-Catalyzed Vinylation of Aryl Halides with Inexpensive Organosilicon Reagents Under Mild Conditions. *Chem. Eur. J.* **2018**, *24*, 10324–10328.

(7) Su, M.; Huang, X.; Lei, C.; Jin, J. Nickel-Catalyzed Reductive Cross-Coupling of Aryl Bromides with Vinyl Acetate in Dimethyl Isosorbide as a Sustainable Solvent. *Org. Lett.* **2022**, *24*, 354–358.

(8) Nuñez, A.; Abarca, B.; Cuadro, A. M.; Alvarez-Builla, J.; Vaquero, J. J. Ring-Closing Metathesis Reactions on Azinium Salts: Straightforward Access to Quinolizinium Cations and Their Dihydro Derivatives. *J. Org. Chem.* **2009**, *74*, 4166–4176.

(9) Stanton, M. P.; Hoover, J. M. Copper-Catalyzed Decarboxylative Elimination of Carboxylic Acids to Styrenes. *J. Org. Chem.* **2023**, *88*, 1713–1719.

(10) Nakano, Y.; Black, M. J.; Meichan, A. J.; Sandoval, B. A.; Chung, M. M.; Biegasiewicz, K. F.; Zhu, T.; Hyster, T. K. Photoenzymatic Hydrogenation of Heteroaromatic Olefins Using 'Ene'-Reductases with Photoredox Catalysts. *Angew. Chem. Int. Ed.* **2020**, *59*, 10484–10488.

(11) Zhang, Y. L.; Wang, G. H.; Wu, Y.; Zhu, C. Y.; Wang, P. Construction of α-Amino Azines via Thianthrenation-Enabled Photocatalyzed Hydroarylation of Azine-Substituted Enamides with Arenes. *Org. Lett.* **2021**, *23*, 8522–8526.

(12) Le Saux, E.; Georgiou, E.; Dmitriev, I. A.; Hartley, W. C.; Melchiorre, P. Photochemical Organocatalytic Functionalization of Pyridines via Pyridinyl Radicals. *J. Am. Chem. Soc.* **2023**, *145*, 47–52.

(13) Yu, Z.; Liu, Q.; Li, Q.; Huang, Z.; Yang, Y.; You, J. Remote Editing of Stacked Aromatic Assemblies for Heteroannular C−H Functionalization by a Palladium Switch between Aromatic Rings. *Angew. Chem.*

Int. Ed. **2022**, *61*, e202212079.

(14) Scheidt, F.; Schäfer, M.; Sarie, J. C.; Daniliuc, C. G.; Molloy, J. J.; Gilmour, R. Enantioselective, Catalytic Vicinal Difluorination of Alkenes. *Angew. Chem. Int. Ed.* **2018**, *57*, 16431–16435.

(15) Scheidt, F.; Neufeld, J.; Schäfer, M.; Thiehoff, C.; Gilmour, R. Catalytic Geminal Difluorination of Styrenes for the Construction of Fluorine-Rich Bioisosteres. *Org. Lett.* **2018**, *20*, 8073–8076.

(16) Wei, S.; Mao, Y.; Shi, S. L. Nickel-Catalyzed Ligand-Free Hiyama Coupling of Aryl Bromides and Vinyltrimethoxysilane. *Synlett* **2021**, *32*, 1670–1674.

(17) Wu, H.; Yang, B.; Zhu, L.; Lu, R.; Li, G.; Lu, H. High-Valent Palladium-Promoted Formal Wagner-Meerwein Rearrangement. *Org. Lett.* **2016**, *18*, 5804–5807.

(18) Shi, Z.; Ji, X.; Shen, C.; Dong, K. Pd-Catalyzed Enantioselective Hydroamidocarbonylation of α-Substituted Acrylamides to Chiral Succinimides. *J. Org. Chem.* **2023**, *88*, 5036–5043.

(19) Zhang, Y.; Sim, J. H.; Macmillan, S. N.; Lambert, T. H. Synthesis of 1,2-Dihydroquinolines via Hydrazine-Catalyzed Ring-Closing Carbonyl-Olefin Metathesis. *Org. Lett.* **2020**, *22*, 6026–6030.

(20) Jang, J.; Bae, Y.; Shin, S. Enantioselective Sulfonium-Claisen Rearrangement with Cinnamyl Thioethers. *Org. Lett.* **2023**, *25*, 3881–3885.

(21) Jagdale, A. R.; Park, J. H.; Youn, S. W. Cyclization Reaction for the Synthesis of Polysubstituted Naphthalenes in the Presence of Au(I) Precatalysts. *J. Org. Chem.* **2011**, *76*, 7204–7215.

(22) Venditto, N. J.; Liang, Y. S.; El Mokadem, R. K.; Nicewicz, D. A. Ketone-Olefin Coupling of Aliphatic and Aromatic Carbonyls Catalyzed by Excited-State Acridine Radicals. *J. Am. Chem. Soc.* **2022**, *144*, 11888–11896.

(23) Bala, K.; Hailes, H. C. Nitrile Oxide 1,3-Dipolar Cycloadditions in Water: Novel Isoxazoline and Cyclophane Synthesis. *Synthesis* **2005**, 3423–3427.

(24) Cao, K.; Tan, S. M.; Lee, R.; Yang, S.; Jia, H.; Zhao, X.; Qiao, B.; Jiang, Z. Catalytic Enantioselective Addition of Prochiral Radicals to Vinylpyridines. *J. Am. Chem. Soc.* **2019**, *141*, 5437–5443.

(25) Wang, H.; Qu, J.-P.; Kang, Y.-B. CBZ6 as a Recyclable Organic Photoreductant for Pinacol Coupling. *Org. Lett.* **2021**, *23*, 2900–2903.

(26) Demidoff, F. C.; Caleffi, G. S.; Figueiredo, M.; Costa, P. R. R. Ru(II)-Catalyzed Asymmetric Transfer Hydrogenation of Chalcones in Water: Application to the Enantioselective Synthesis of Flavans BW683C and Tephrowatsin E. *J. Org. Chem.* **2022**, *87*, 14208–14222.

(27) Lee, K. N.; Lei, Z.; Ngai, M. Y. β-Selective Reductive Coupling of Alkenylpyridines with Aldehydes and Imines via Synergistic Lewis Acid/Photoredox Catalysis. *J. Am. Chem. Soc.* **2017**, *139*, 5003–5006.

(28) Zhang, M.; Xie, J.; Zhu, C. A General Deoxygenation Approach for Synthesis of Ketones from Aromatic Carboxylic Acids and Alkenes. *Nat. Commun.* **2018**, *9*, 3517.

(29) Fava, E.; Nakajima, M.; Nguyen, A. L. P.; Rueping, M. Photoredox-Catalyzed Ketyl-Olefin Coupling for the Synthesis of Substituted Chromanols. *J. Org. Chem.* **2016**, *81*, 6959–6964.

(30) McKnight, J.; Shavnya, A.; Sach, N. W.; Blakemore, D. C.; Moses, I. B.; Willis, M. C. Reductant-Free Cross-Electrophile Synthesis of Di(Hetero)Arylmethanes by Palladium-Catalyzed Desulfinative C−C Coupling. *Angew. Chem. Int. Ed.* **2022**, *61*, e202116775.

(31) Pavlishchuk, V. V.; Addison, A. W. Conversion Constants for Redox Potentials Measured versus Different Reference Electrodes in Acetonitrile Solutions at 25°C. *Inorganica Chim. Acta* **2000**, *298*, 97–102.
